# Environmental chamber studies of eye and respiratory irritation from use of a peracetic acid–based hospital surface disinfectant

**DOI:** 10.1017/ash.2023.138

**Published:** 2023-04-11

**Authors:** Pamela H. Dalton, Christopher Maute, Jeffrey B. Hicks, Heather N. Watson, Anne E. Loccisano, Brent D. Kerger

**Affiliations:** 1 Monell Chemical Senses Center, Philadelphia Pennsylvania; 2 Exponent Inc., Oakland California; 3 Exponent Inc., Alexandria Virginia; 4 Exponent Inc., Irvine, California

## Abstract

**Objective:**

To characterize personal exposures and measures of eye and respiratory tract irritation in controlled environmental chamber studies of 44 healthy adult volunteers simulating upper-bound use of peracetic acid (PAA)–based surface disinfectant for terminal cleaning of hospital patient rooms.

**Design:**

Experimental, within-subject, double-blinded cross-over design.

**Methods:**

Objective and subjective exposure effects were assessed for PAA and its components: acetic acid (AA) and hydrogen peroxide (HP). Deionized water was included as a control. Breathing-zone concentrations of PAA, AA, and HP were assessed for 8 female multiday volunteers (5 consecutive days) and 36 single-day volunteers (32 females and 4 males). Wetted cloths were used to wipe high-touch surfaces for 20 minutes per trial. Also, 15 objective measures of tissue injury or inflammation and 4 subjective odor or irritation scores were assessed.

**Results:**

Disinfectant trials showed 95th percentile breathing zone concentrations of 101 ppb PAA, 500 ppb AA, and 667 ppb HP. None of the volunteers observed over 75 test days exhibited significant increases in IgE or objective measures of eye and respiratory tract inflammation. Subjective ratings for disinfectant and AA-only trials showed similar increases for odor intensity and nose irritation, with lower ratings for eye and throat irritation. Females were 2.5-fold more likely than males to assign moderate + irritation ratings.

**Conclusions:**

Simulated upper-bound hospital use of PAA-based disinfectant led to no significant increases in objective markers of tissue injury, inflammation, or allergic sensitization, and no frank signs of eye or respiratory tract irritation.

Hospital-acquired infections, also known as healthcare-associated infections (HAIs), are nosocomial infections that are not present at the time of admission.^1^ Due to the potential for such infections to be transmitted widely with serious consequences, hospitals have established infection tracking and surveillance systems in place, along with robust prevention strategies to reduce the rate of hospital-acquired infections.^2–4^ Beginning in 2013, peracetic acid (PAA)–based disinfectants were introduced into a number of hospitals because of their superior ability to eliminate *Clostridium difficile* and other dangerous pathogens. Confirmation of this efficacy was recently demonstrated in a study showing that hospital-wide surface hygiene protocols with a PAA-based surface disinfectant containing active ingredients PAA (0.13%) and hydrogen peroxide (HP, 0.64%) reduced hospital-acquired *Clostridioides difficile* infections by 50% on average in a study across 8 hospitals over 3 years.^5^ In 2022, Carling et al^5^ noted that such use of PAA-based disinfectant reduced a variety of other hospital-acquired infections: *Staphylococcus aureus* (MRSA), *Enterococci* (VRE), *norovirus*, *Klebsiella pneumoniae*, *Candida auris*, and other multidrug-resistant organisms (MDROs).^6–14^ Despite the demonstrated efficacy of a PAA-based disinfectant in the control of hospital-acquired infection transmission, concerns have been raised regarding the sensory and health impacts of exposure to PAA disinfectants on environmental service workers.

National Institute for Occupational Safety and Health (NIOSH) investigations of 2 US hospitals using PAA-based surface disinfectant evaluated self-reported health complaints relating to skin, eye, and respiratory tract irritation among staff.^15–17^ Subjective responses of “difficulty breathing” in the 2018 study were most strongly associated with “sensitizer and irritant use” or “total stress.” In the 2019 study, participants frequently attributed their symptoms as work-related regardless of their frequency or magnitude of PAA-based disinfectant exposure. Work-shift average exposures were assessed in both studies, showing 95^th^-percentile PAA exposures up to 48 ppb, well below the 160-ppb level that is considered to be generally well tolerated.^18^ The final evaluations in both the 2018 and 2019 NIOSH reports did not find a robust statistical relationship between the self-reported health effects and work-shift concentrations of active ingredients PAA and HP. However, in 2017, Hawley et al^15^ concluded that their findings suggested that PAA and HP “contributed to eye and respiratory symptoms reported by hospital cleaning staff at low levels of measured exposure.”^15^

Many disinfectant cleansers, particularly those used in hospital settings, have strong pungent odors that can cause worker complaints and discomfort, particularly upon initial use.^19^ However, there is substantial variability in the acceptance of PAA-based cleaner. For example, staff members in a South Australian hospital using a chlorine-based disinfectant reported adverse respiratory reactions to the cleaner. Replacing the chlorine-based disinfectant with a hydrogen peroxide disinfectant eliminated respiratory complaints but resulted in an increase of slip incidents and the need for additional cleaning due to a build-up of product residue on surfaces. A 1-year trial of a buffered PAA-based disinfectant, however, resolved the residue problem and, despite the vinegar odor, resulted in no respiratory issues reported by staff, including those originally affected by the chlorine-based cleaner.^20^

Thus, in present study, we evaluated the irritant and inflammatory potential in the eyes and respiratory tract from a PAA-based disinfectant under conditions of simulated upper-bound use in a hospital setting. Although allergic hypersensitivity (ie, immunoglobulin E (IgE)-mediated immediate or delayed-type hypersensitivity) to PAA has not been reported in the scientific literature to date, we also sought to evaluate whether 5 consecutive days of exposure to a PAA-based disinfectant increased serum IgE levels. Auxiliary studies characterizing disinfectant product dispenser calibration and environmental service-worker use patterns for hospital patient room cleaning and mass transfer characteristics of surface application and breathing zone PAA concentrations when applying a high-end disinfectant concentration will be reported separately.

## Methods

### Participants

Two subgroups of volunteers participated: (1) single-day volunteers (32 females and 4 males; mean age, 40.7; SEM, 0.59; median age, 40.8 years) to assess the range of individual exposures and irritant responses, and (2) multiday volunteers (8 females; mean age, 36.3; SEM, 0.31; median age, 34.6 years) whose exposures and irritant responses were assessed over 5 consecutive-use days.

The study was approved by the Advarra Institutional Review Board and full consent was obtained from each of the healthy volunteers. Study participants were recruited by Monell Chemical Senses Center and were compensated for their participation. Predominantly female participants were included because they are significantly more prone to report odor-related irritation compared to male participants,^21–24^ and they exhibit irritant receptor-triggered cough reflex at lower average airborne-irritant concentrations compared to males (ie, with capsaicin exposures).^25,26^ None of the participants reported a history of any respiratory conditions, including asthma, and none reported any prior exposure to any PAA-based disinfectant.

### Procedure

**Fig. 1.**
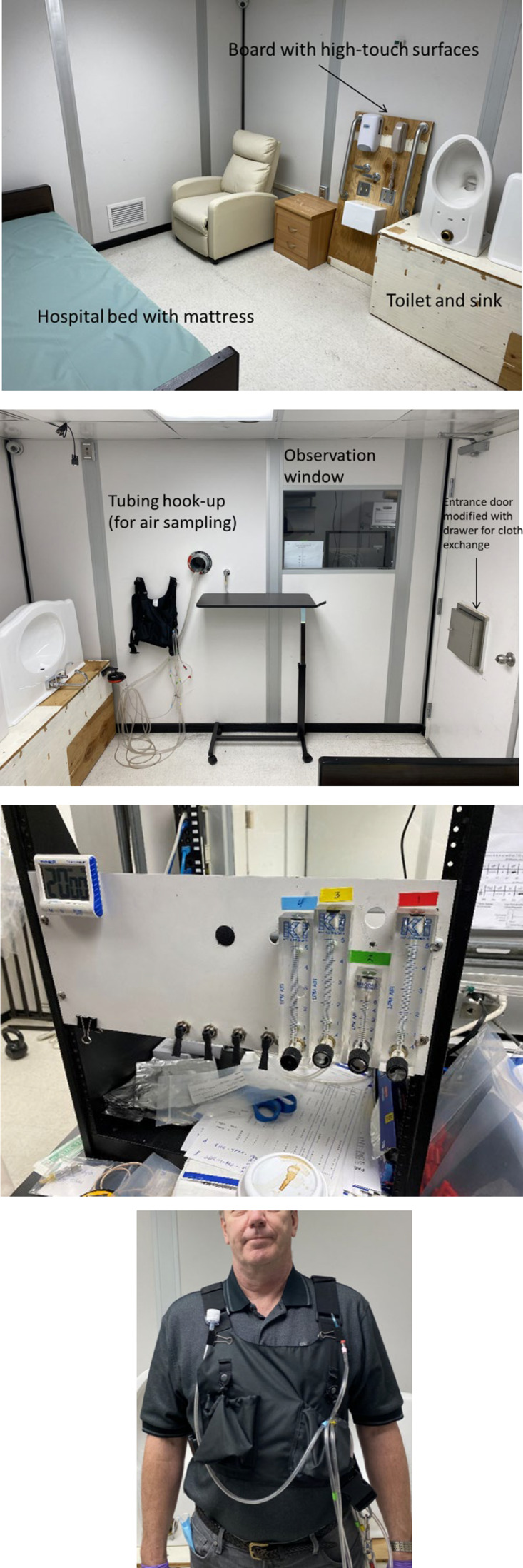
Chamber configuration and equipment at the Monell Chemical Senses Center. Top 2 photos: Environmental chamber configuration with furniture and high-touch surfaces, sample tubing hook-up, and chamber entrance door, which was modified with a drawer for cloth exchange. Third from top: Sample collection manifold constructed with 4 key instrument rotameters and a Gast DOA P707-AA vacuum pump. Bottom: Customized sampling vest, with Tygon tubing from the 4-channel sample collection manifold to the breathing zone of the volunteer and connected to the sample cartridges.

In this study, we utilized an experimental, within-subject, double-blind, crossover design for evaluating eye and respiratory irritation responses in healthy human volunteers to a PAA-based disinfectant and its components in an environment chamber, which was set up to simulate a hospital room (Fig. 1). The environmental chamber had dimensions of 2.9 m wide × 3.6 m long × 2.2 m height. It was equipped to allow for control and monitoring of supply air, exhaust air (set at 5.4 air changes per hour for this study based on typical hospital rooms), and temperature (21°C) using an air-control system (Siemens, Berlin, Germany). Temperature and airflow were recorded at 1-minute intervals (data shown in Supplementary Appendix 1).

**Fig. 2.**
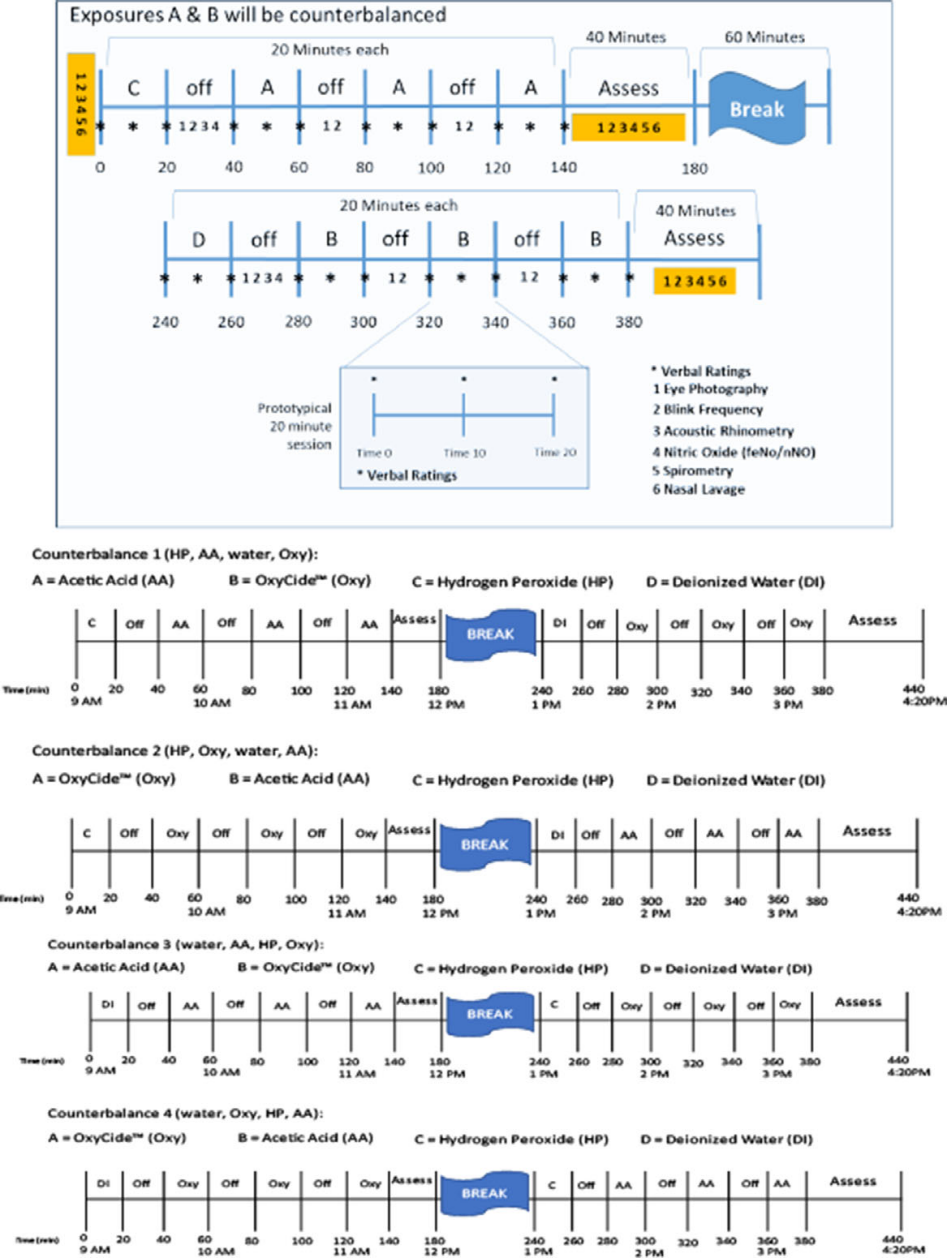
Summary of the timing of exposures and endpoints evaluated on each test day and the 4 counterbalance sequences that were varied for each Monell volunteer.

Participants were exposed during each of 4 conditions during a single day: (1) PAA-based disinfectant (positive odor or irritant), (2) acetic acid (AA) only (positive odor or irritant), (3) HP only (negative odor or irritant), and (4) water only (negative odor and negative irritant control) (Fig. 2). Exposures were ordered in 1 of 4 counterbalanced sequences, which were randomized by participant (Supplementary Appendix 3). Figure 2 also summarizes the timing of the exposure trials and the end points evaluated during each test day.

The PAA-based disinfectant use solution (3 oz OxyCide™ concentrate per gallon of water; Ecolab, Minneapolis, MN) contained 0.13% PAA, 0.16% AA, and 0.64% HP (Supplementary Appendix 1). The AA-only solution (0.38%) and the HP-only solution (0.63%) were utilized to generate similar airborne exposures as observed with the disinfectant mixture. Solutions were made fresh daily and were evenly distributed over a designated number of microfiber cloths (dimensions 38 cm × 40 cm) at a saturation level of 125 mL per cloth that could be used for surface cleaning without dripping, in accordance with the disinfectant manufacturer’s training materials for use (Supplementary Appendix 2).

Exposures to PAA, AA, and HP were measured in the volunteer’s breathing zone during each trial, and pre-wetted microfiber cloths were used to wipe for 20 minutes continuously to disinfect a selected set of high-touch nonporous surfaces found in patient room or bathroom spaces (8.54 m^2^ surface area) (Fig. 1). Volunteers were instructed to use the wetted cloths to wipe on a heavy film and to continue repeating the cleaning cycle of high-touch surfaces in accordance with the disinfectant manufacturer’s training materials for use until the 20-minute trial was completed. Study participants donned a sampling vest and wore nitrile gloves; 1 of 4 wetted cloths was provided via a pass-through compartment at 0-, 5-, 10-, and 15-minute intervals; spent cloths were exchanged and removed. Breathing zone samples for PAA, AA, and HP during each trial were collected at 4 L per minute and shipped under chain-of-custody records to an EPA-certified laboratory for extraction and quantitative analysis based on OSHA method PV2321 for PAA, NIOSH method 1603 for AA, and OSHA method 1019 for HP. Further details on the exposure characterization methods and results are reported in Supplementary Appendix 1.

The following objective measures were applied: eye imaging for measurement of eye-blink frequency and hyperemia (eye redness, vascularity); nasal assessment of right and left sinus volume by nasal rhinometry; nasal nitric oxide; nasal mucus collection for measuring selected cytokines of respiratory tract inflammatory response (interleukin-8, calcitonin gene-related peptide, substance P, and tumor necrosis factor α); and respiratory assessment of exhaled breath nitric oxide and spirometry (FEV1, FVC, and FEV/FVC). Multiday volunteers were also assessed for allergic sensitization by analysis of blood immunoglobulin E before the first exposure trial and after the last trial on day 5. Each trial was video-recorded, and participants were observed for any frank signs of eye or respiratory irritation (ie, lacrimation, sneezing, coughing, or nasal congestion or dripping). Further details on the irritation metric methods and data collected are reported in Supplementary Appendix 3.

**Fig. 3.**
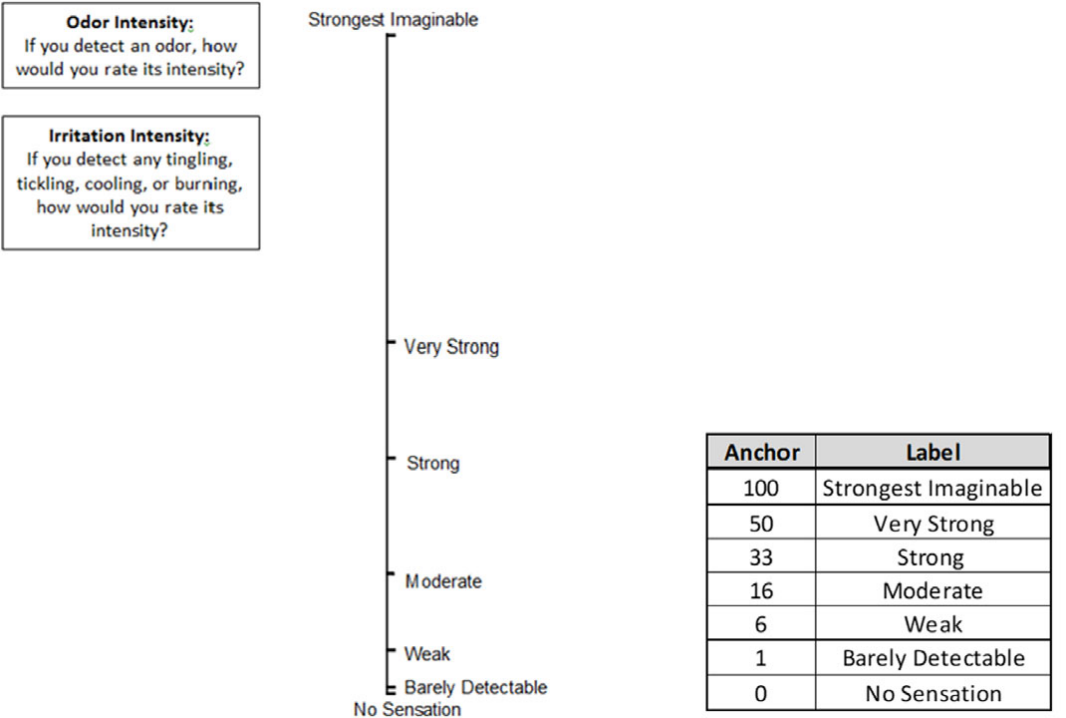
Labeled magnitude scale and corresponding anchors used for analysis of subjective symptoms.

Eye and respiratory irritation were measured during each trial using objective measures and subjective ratings of irritation intensity (odor, eye, nose, and throat). The general labeled magnitude scale,^27,28^ a log scale for sensation intensity, was used by each volunteer to assign ratings anchored by corresponding magnitude descriptors (Fig. 3).

### Statistical analysis

For the statistical analysis, summary statistics were calculated by solution for each exposure and irritation metric. For the exposure characterization, analysis of variance models (ANOVAs) were implemented using the Tukey multiple comparison test to identify significantly different group means. All analyses were performed in SAS version 9.4 software (SAS Institute, Cary, NC).

For the subjective irritation scores, ANOVAs were used with conditions (A–D) and time (before or after and hour) as factors to understand the effects of OxyCide™ exposure. Any interactions revealed by analysis were explored using a Tukey HSD post hoc analysis, with Bonferroni corrections as appropriate. All analyses were performed in Statistica software (TIBCO Software, Palo Alto, CA).

## Results

Mean ± standard deviation personal exposures for the disinfectant trials were 66 ± 23 ppb PAA, 287 ± 121 ppb HP, and 387 ± 148 ppb AA; the relevant component exposures were comparable in the AA-only and HP-only trials (Table 1). Similar personal exposure levels were identified for the 36 single-day and the 8 multiday participants (results not shown). The 4 male single-day volunteers had significantly lower PAA exposures compared to the 32 female single-day volunteers only for the disinfectant trials (30% lower; *P* = .0065). None of the volunteers exhibited increases in objective measures of eye and respiratory tract inflammation. We observed no increase in ocular redness or vascularity following any exposure condition. In addition, no significant elevations were detected for nasal or lung nitric oxide, and no decreases were observed for any of the spirometry end points (FEV_1_, FVC, FEV_1_/FVC). None of the 8 multiday volunteers exhibited increased serum IgE after exposure day 5 compared to their pre-study value (Supplementary Appendix 3).

**Table 1.** Summary Statistics for Monell Environmental Chamber Studies of OxyCide™ and Its Components: All Data Combined^a^

	Peracetic Acid in µg/m^3^ (ppb)^b^				Hydrogen Peroxide in µg/m^3^ (ppb)^c^				Acetic Acid in µg/m^3^ (ppb)^d^			
Chamber test solution	No.	Mean	SD	SEM	No.	Mean	SD	SEM	No.	Mean	SD	SEM
OxyCide™	219	205.9 (66.2)	71.9 (23.1)	4.8 (1.6)	219	398.6 (286.6)	168.1 (120.9)	11.3 (8.1)	219	950.1 (387)	362.8 (148)	24.4 (10)
Acetic acid only	193	13.1 (4.2)	10.0 (3.22)	0.7 (0.2)	187	121.1 (87.1)	80.2 (57.7)	5.8 (4.2)	179	967.9 (394)	471.1 (192)	34.4 (14)
Hydrogen peroxide only	71	14.8 (4.8)	14.7 (4.71)	1.7 (0.6)	71	348.6 (250.7)	154.6 (111.1)	18.1 (13)	71	190.8 (78)	159.8 (65)	18.7 (8)
Deionized water only	75	13.3 (4.3)	12.7 (4.1)	1.5 (0.5)	75	25.1 (18)	21.2 (15.3)	2.5 (1.8)	75	170.0 (69)	127.9 (52)	14.8 (6)
95^th^ percentile for OxyCide™ use in µg/m^3^ (ppb)	315 (101)				695 (500)				1,640 (667)			

Note. SD, standard deviation; SEM, standard error of the mean.

a Summary of data for the 36 single-day volunteers combined with all data from the 8 multiday volunteers; thus, a total of 75 days of testing is reflected in the data presented here for the OxyCide™, hydrogen peroxide–only, and deionized water–only trials. The first 2 weeks was designated as the pilot study, wherein it was determined that the acetic acid only trials exhibited lower-than-expected acetic acid airborne concentrations (mean, 158 ppb; SD, 59 ppb). The acetic acid cleaning solution was increased by 2.4-fold to achieve equivalent concentrations to those observed in the OxyCide™ trials (mean, 353 ppb; SD, 121 ppb). Thus, a total of 65 days of testing is reflected in the data for the acetic acid–only trials.

b The average peracetic acid concentration was significantly different between OxyCide™ and the other chamber test solutions (*P* < .001). The other pairwise comparisons were not statistically significant.

c All pairwise comparisons between solutions were statistically significantly different (*P* < .001), with the exception of OxyCide™ and hydrogen peroxide only (*P* = .1954).

d The average concentration for the OxyCide™ and acetic acid only groups was significantly higher than both hydrogen peroxide and deionized water (*P* < .001). No significant difference was detected between hydrogen peroxide only and deionized water only (*P* = .9837) or OxyCide™ and acetic acid only (*P* = .1997).

Subjective irritation scores for the disinfectant trials (PAA/AA/HP) and for the AA-only trials were similarly elevated for odor and nose, with lower scores for eye and throat (Table 2). Female participants were significantly more likely than male participants (odds ratio, 2.5; 95% confidence interval, 1.7–3.8) to rate subjective odor intensity and nasal irritation at moderate or higher levels for the disinfectant cleaning trials (Supplementary Appendix 3).

**Table 2.** Summary Statistics for Subjective Irritation Scores: Average Subjective Ratings of Intensity and Irritation

Factor	Condition							
	AA		PAA/AA/HP		HP		DI	
	Mean	SE	Mean	SE	Mean	SE	Mean	SE
Odor intensity	12.06	2.16	22.99*	2.90	8.10	1.53	7.26	1.85
Eye irritation	4.80	1.20	13.62*	2.10	2.71	0.68	3.01	0.78
Nose irritation	8.60	1.64	23.78*	2.45	6.60	1.52	5.00	1.05
Throat irritation	4.36	1.07	12.65*	2.05	3.15	0.72	2.65	0.99

Note. AA, acetic acid; PAA, peracetic acid; HP, hydrogen peroxide; DI, deionized water; SE, standard error.

* *P* < .001.

## Discussion

This study of upper-bound personal exposures during simulated hospital use of PAA-based disinfectant demonstrated that, based on mean subjective irritation scores for the disinfectant solution trials (PAA/AA/HP) and the AA-only trials, the volunteers considered their personal exposures to be significantly more irritating on average compared to HP-only or deionized water trials. Notably, eye irritation, which is typically a more sensitive locus for airborne irritants, particularly among women,^29,30^ was reported as significantly lower than nasal irritation. The similarity between the ratings of odor intensity and the nasal irritation ratings to disinfectant solution and AA-only trials suggests that the combined sensations of a strong and unusual odor with tingling sensations in the nose served to elevate the reporting of nasal irritation. Importantly, none of the volunteers had significant increases in objective markers of inflammation or allergic sensitization, and none exhibited any ocular or respiratory tract irritation. These findings demonstrate that no significant increase in objective markers of tissue injury or inflammation of the eye or respiratory tract were observed for healthy volunteers exposed during upper bound use of the PAA-based disinfectant or its components (HP only and AA only) despite the broad range of subjective irritation scores describing the intensity of unpleasant sensation for the 44 volunteers studied over 75 days of testing.

Our study design included predominantly female participants because compared to males they are significantly more prone to report odor-related irritation^21–24^ and show a lower threshold for irritant receptor-triggered cough reflex.^25,26^ This element was part of the upper-bound response analysis that was incorporated into the study design. Consistent with the published findings showing greater sensitivity to odors and inhaled irritants, female participants in the current study were significantly more likely (2.5-fold) than male participants to rate subjective odor and nasal irritation as “moderately sensed” or greater. The small subset of men (n = 4) showed a statistically significant 30% lower mean breathing zone PAA compared to the 40 women and had lower means for all other exposure metrics that were not significantly different at *P* < .05. These lower breathing-zone measurements may be attributable to greater male volunteer height (ie, source to breathing zone distance), differences in wiping technique, and/or chance related to the far fewer observations for men relative to women studied.

The current study was designed to simulate upper-bound environmental service (EVS) worker use of PAA-based disinfectant for terminal cleaning of hospital patient room and bathroom spaces. Each trial included 20 minutes of continuous wiping high-touch surfaces with wetted cloths according to the product manufacturer’s training guidelines for cleaning. Auxiliary studies (Supplementary Appendix 1) evaluated 49 EVS workers across 15 hospitals and demonstrated that the selected 20 minutes wiping period for PAA-based disinfectant exceeded the interquartile range (IQR) of 8.8–18 minutes in the field. An IQR of 3–7 cloths used per patient room or bathroom and an IQR of 1.7–4.1 minutes for cloth handling time were observed versus the current study protocol utilizing 4 cloths handled for 5 minutes each. Separate studies (Supplementary Appendix 1) evaluating the mass transfer characteristics of PAA-based disinfectant at a higher concentration (4 oz per gallon of water versus the current study at 3.0 oz per gallon) showed 24 ppb higher average breathing zone PAA concentrations (average 101 ppb PAA at 4 oz per gallon versus 66 ppb in current study at 3 oz per gallon) (Supplementary Appendix 1). Compared to longer periods of individual wetted cloth use (ie, 10 minutes per cloth vs 20 minutes per cloth), using cloths for <5 minutes led to significantly higher disinfectant film density (average, 4.6 g/m^2^) and application time (average, 9.8 g/minute versus 4.1 g/minute with 1 cloth per 20 minutes). It also led to significantly increased cleaning efficiency (average, 2.2 m^2^/minute versus 1.6 m^2^/minute with 1 cloth per 20 minutes), longer dwell time (>5 minutes), and significantly reduced PAA transfer to the user’s breathing zone (an average of 7.0% PAA transfer vs 14.9% at 1 cloth per 20 minutes). These findings suggest that applying a heavier film density of PAA-based disinfectant reduces the fraction immediately released to air, thereby increasing the PAA mass retained on surfaces to accomplish antimicrobial efficacy goals.

The current study chamber had a fresh-air exchange rate of 5.4 to 5.5 per hour, which was below the minimum recommended 6 hours for patient rooms.^31^ Perhaps most importantly, we examined disinfectant use in a chamber equipped with a condensed set of high-touch surfaces within in a small floor space (11.5 m^2^) compared to an expected floor space (patient room + bathroom) in hospitals of 24–30 m^2^.^32^ The volunteers completed 3–5 full cycles of simulated cleaning in 20 minutes, corresponding to a total surface area cleaned at 25–34 m^2^, comparable to the total floor space of typical patient room and bathroom spaces. Considered collectively, these study design elements reasonably represent upper bounds for use conditions and breathing zone exposures to PAA, HP, and AA for EVS workers during patient room terminal cleaning.

In conclusion, the current study findings provide important dose–response evidence for eye and respiratory irritation/inflammatory responses among healthy human volunteers exposed during upper-bound use conditions of PAA-based hospital surface disinfectant or its components, HP and AA. The range of breathing-zone concentrations reported for these studies demonstrate no objective effects on eye or respiratory tract tissue injury or inflammation, and, among a subset of the volunteers, did not induce allergic sensitization following 5 consecutive days of exposure to the disinfectant or its components.

Although environmental service workers have far longer-term exposure to PAA-based disinfectants than the participants in these studies, the irritation effects of PAA are known to be concentration dependent rather than concentration-times-duration dependent.^33^ With continued exposure, sensory adaptation is a common feature of human sensory systems—odor and irritation—has been demonstrated for numerous substances,^34,35^ including acetic acid.^36^ Confirmation of this shift in response is shown by the dramatic reduction in health complaints made to the manufacturer or its independent agent of the disinfectant from 2013 to 2020 (Supplementary Appendix 2). The data presented here demonstrate the range of subjective irritation responses of naïve individuals to a PAA-based disinfectant and AA-only under conditions of simulated environmental service worker use, but no significant increases in objective irritation measures of eye and respiratory irritation, inflammation, or sensitization.

## Supplementary material

For supplementary material accompanying this paper visit http://doi.org/10.1017/ash.2023.138. click here to view supplementary material
