# Supplementary material for: Environmental chamber studies of eye and respiratory irritation from use of a peracetic acid–based hospital surface disinfectant
Source: Antimicrob Steward Healthc Epidemiol. 2023 Apr 11;3(1):e71. doi: 10.1017/ash.2023.138 (PMC10127244; doi:10.1017/ash.2023.138)
Supplement: Supplementary file 1 [file ashsup.zip › S2732494X23001389sup001.pdf]

Supplemental Appendix 3: Assessments and Analysis

Materials and Methods

Participants

Forty-four healthy, volunteers (4 male), aged 39, (SEM=1.5) were recruited from the local Philadelphia, PA area to participate in this study. Exclusion criteria included pregnancy, smoking or vaping, regular exposure to second-hand smoke, current respiratory infections or history of: respiratory or cardiac conditions, chronic rhinosinusitis or polyposis, active allergies, occupational chemical exposure (past or present) and a number of psychiatric diagnoses including claustrophobia.

Screening/Data Collection Sessions

Screening Session

After consenting, pulmonary function was measured via spirometry to screen for respiratory conditions. All results were reviewed by an occupational physician prior to enrollment. Participants were deemed as screen fails if: 1) they could not achieve two 'good efforts' as determined by the KOKO Px software, or 2) they achieved three 'good efforts' but didn't qualify based on examination of results by an occupational physician. Upon verification that a subject was eligible to participate, they were scheduled for the testing session at the Monell Center.

Exposure Sessions

Figure 1 presents the study timeline of events, the details of which are expanded on in the **'Assessments'** section below.

The first half of the day began with a collection of endpoints/assessments followed by 4-20 minute cleaning sessions, separated by 20 minutes, during which participants cleaned a room outfitted with furniture found in a hospital room. Participants were handed wetted cloths containing either Oxycide™ (peroxyacetic acid [PAA]), its components (acetic acid [AA] or hydrogen peroxide [HP]), or deionized water (DI) every 5 minutes with each cleaning session dedicated to one of the four conditions. Cleaning sessions were separated by a 20-minute break during which more endpoints were collected. The first of the 4 cleaning sessions (morning or afternoon) used HP or DI with the remaining 3 sessions using PAA or AA. Endpoints and assessments were collected after the fourth session after which participants had a lunch break. The second half of the day followed the same protocol as the first half using different conditions.

The cleaning conditions and the counterbalancing are listed in Tables 1 & 2, respectively.

Cleaning Conditions	
A	Acetic Acid (AA)
B	Peroxyacetic Acid (PA)
C	Hydrogen Peroxide (HP)
D	Dionized Water (DI)

Table 1. Codes for Cleaning Conditions

Counterbalance 1a		Counterbalance 2a	
C	Hydrogen Peroxide (HP)	C	Hydrogen Peroxide (HP)
A	Acetic Acid (AA)	B	Peroxyacetic Acid (PA)
D	Dionized Water (DI)	D	Dionized Water (DI)
B	Peroxyacetic Acid (PA)	A	Acetic Acid (AA)

Counterbalance 1b		Counterbalance 2b	
D	Dionized Water (DI)	D	Dionized Water (DI)
A	Acetic Acid (AA)	B	Peroxyacetic Acid (PA)
C	Hydrogen Peroxide (HP)	C	Hydrogen Peroxide (HP)
B	Peroxyacetic Acid (PA)	A	Acetic Acid (AA)

Table 2. Counterbalancing of Cleaning Conditions

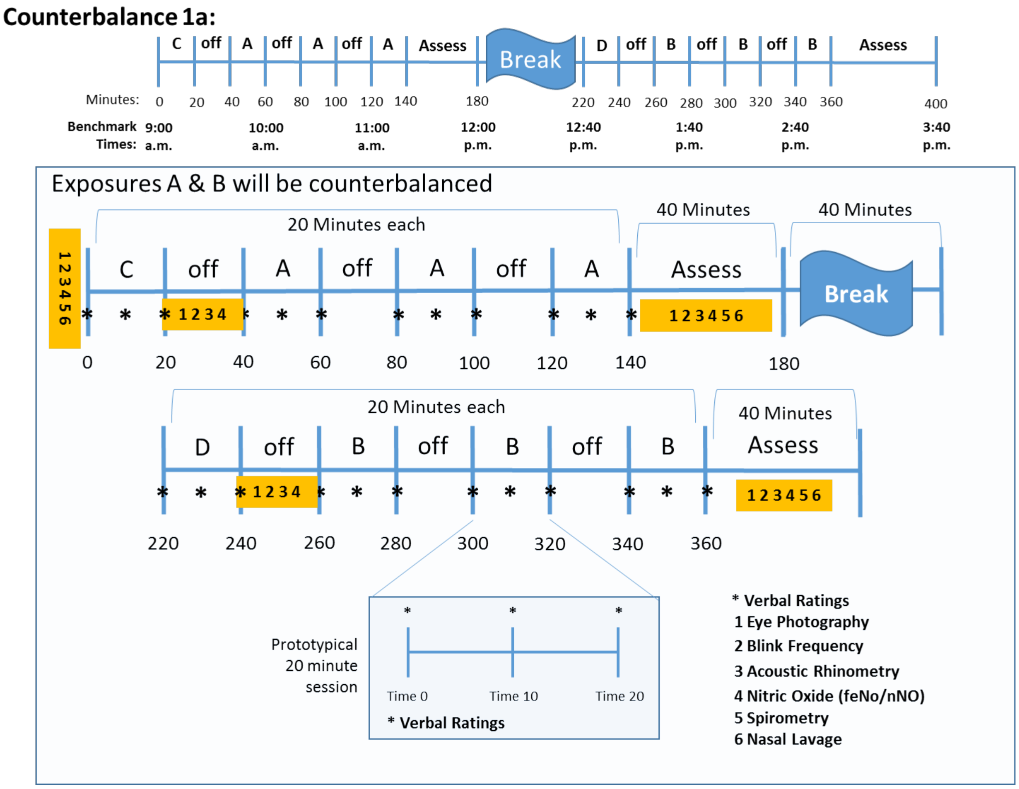

Figure 1. Timeline for Testing Session and collection of Endpoints/Assessments using Counterbalance 1a for Illustration

Exposure took place in an environmental chamber which allowed the control and monitoring of temperature and airflow. Temperature and humidity were collected at 1-min intervals using a HOBO MX1101 data logger (Onset, Bourne, MA, USA). Airflow was monitored using the Siemens control system for the chamber.

Endpoints and Assessments

Endpoints
Verbal Ratings
Eye Photography
Blink Frequency
Acoustic Rhinometry
Nitric Oxide (breath and nasal)
Spirometry
Nasal Lavage

Table 3. Subjective and Objective Endpoints and Assessments

Table 3 provides, along with the study timeline in Figure 1, the names of the endpoints collected for the study. What follows is a more detailed explanation of them and how they were collected.

Subjective Verbal Ratings of Irritation (All days)

Immediately upon entry into the exposure chamber, and at specified time periods during exposure, subjects made subjective ratings of perceived intensity of odor and irritation – as defined as the cooling, burning, tingling, tickling sensations in the eyes, nose, or throat– on a general labelled magnitude scale (gLMS).

Participants made ratings verbally using a gLMS scale they could see in the testing room with rating labels places at specific numeric anchors. Verbal ratings were collected by the researcher by entering into a spreadsheet for analysis.

Eye Photography for Ocular Hyperemia and Vascularity

Pictures (24 bit, 72 dpi; see Figure 2) were taken of participants' eyes with an Apple iPod touch (7th generation). Three pictures were taken of each eye while participants held down the corner of their eye, pointing their pupil in the opposite direction.

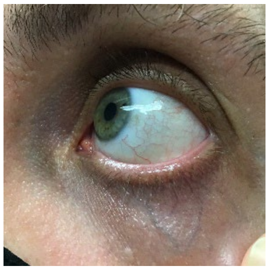

Figure 2. Photo sample of eye conjunctiva for analysis

Pictures were then uploaded to an online analysis platform run by Advanced Opthalmic Systems (AOS; https://aos-hub.com/; Croydon, UK). The AOS platform allows users to, among other things, select the conjunctival area of the eye for analysis of hyperemia (redness--scored 0-4) and vessels (0-100%) which can be summarized as a report (see Figure 3).

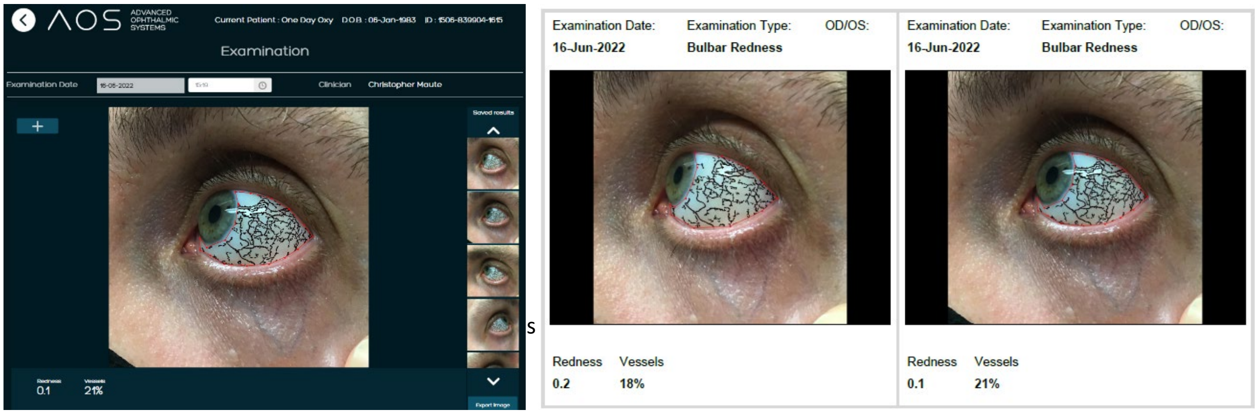

Figure 3. (Left) Screen capture of AOS online analysis platform and (Right) sample of report generated.

Blink Frequency

Blink frequency can increase as an adaptive response to dryness or mild irritation. Participants were video-recorded with a webcam (Logitech 4K Pro Webcam) while they stared at a random point in front of them and blinked naturally for one minute. The video was then independently coded for amount of blinks per minute by multiple technicians counting blinks with a tally counter.

Acoustic Rhinometry

Participants performed Acoustic Rhinometry (AR) to assess and monitor any increases or decreases in the volume of the sinus cavities. Using a GM Instruments A1 Acoustic Rhinometer (Irvine, UK), participants held a sound tube up to each nostril, one at a time, while a soundwave was emitted and reflected back from their sinus cavity. Measurements are delivered in increments of cubic centimeters (cm^3)

Nitric Oxide

Nitric oxide (NO) measurements were measured as a proxy for respiratory inflammation. Both fractional exhaled nitric oxide (FeNO) and nasal nitric oxide (nNO) measurements were made on the Eco Medics CLD88 NO_x analyzer and Spiroware Software with the DENOX 88 (ECO MEDICS AG, Duernten, Switzerland) attachment used for FeNO.

FeNO is a measure of the level of NO in an exhaled sample of breath. It is collected by having participants breathe at a specific rate and flow into a mouthpiece attached to the DENOX88, which applies resistance to exhaled breath with an NO-free air supply as it samples the exhaled breath for anlaysis. Participants completed five trials of FeNO measurement.

nNO is a measure of NO in a sample collected from the sinus cavity. It is collected by having participants hold their breath while wearing a nosepiece connected to a sampling tube attached to the CLD88. Participants completed five trials of nNO measurement. Prior to nNO collection, nasal patency was evaluated using the In-check (Clement Clarke International, Harlow, UK) peak nasal inspiratory flow (PNIF) meter, to interpret the nNO reading, as decreased nasal patency will decrease the amount of nNO recorded.

Spirometry (Screening/All days)

Pulmonary function was measured using the KOKO system used at screening. For each trial of spirometry, participants inhaled deeply and quickly through a mouthpiece attached to the handheld device then exhaled as hard as they could through the mouthpiece for 6 – 10 seconds. For testing purposes participants were allowed up to 5 trials to achieve two ‘good efforts’ as determined by the software.

Data was analyzed as a percentage of the predicted performance of participants based on their demographics (e.g., age, gender, race, height, & weight) based on NHANESIII data stored in the software.

Nasal Lavage

Mucus was collected 3 times over the test day via nasal lavage which involves the spraying of saline (0.65% Sodium Chloride in purified water (CVS Pharmacy, Inc., Woonsocket, RI, USA) into the nose using a dose-metered spray bottle and letting the saline and any loosened mucus and cells drip into a cup (Dalton, Opiekun et al. 2010). The collected liquid was then filtered through a 40 µm sterile cell strainer (Foxx Life Sciences, Salem, NH, USA) into a 50 ml conical tube (Corning Life Sciences, Corning, NY, USA), then stored at -80°C prior to analysis.
Il-8 and TNFα were analyzed using a cytokine/chemokine panel obtained from EMD Millipore, Billerica MA HCYTOMAG-60K-11. Substance P was analyzed using an elisa from Enzo Life Sciences, Farmingdale NY, kit ADI-901-018A. CGRP was analyzed using an elisa from Nordic Biosite, Taby Sweden, kit EKX-73YC16-96.

STATISTICAL ANALYSIS AND RESULTS

General Statistical Analysis Plan

Analyses of Variance (ANOVAs) were used with CONDITION (A, B, C, D) and TIME (Pre-/Post- [as needed], Hour [as needed]) as factors to unpack effects of OxyCide exposure. Any interactions revealed by analysis were explored using a Tukey HSD post hoc analysis, with Bonferroni corrections as appropriate. All analyses were done in STATISTICA (TIBCO Software, Palo Alto, CA, USA).

Results

Ocular Hyperemia and Vascularity

Eye photographs were taken at baseline (before exposure) and after the completion of each condition. Scores of eye redness and vessels showed no change of interest.

Blink Frequency

Video recordings were filmed at baseline (before exposure) and immediately after the completion of each condition. All conditions showed a small increase in blink rate compared to the averaged baseline. There was a significant CONDITION effect ($F(4, 160)=6.48$, $p<0.001$) with a slight increase in blink rate for PAA compared to baseline, but not compared to the blink rate in the Deionized water condition (see Table 4).

	Eye Blink Rate	
**Condition**	**Ave**	**SE**
**Baseline**	18.95	*1.77*
**AA**	21.60	*1.43*
**PAA**	26.48	*2.03*
**HP**	22.16	*1.66*
**DI**	23.37	*1.96*

Table 4. Average Eye Blink Rate (blinks per minute) per Condition

Acoustic Rhinometry

Acoustic rhinometry measurements were collected at baseline (before exposure) and after the completion of each condition. There was a CONDITION Main Effect ($F(4, 156)=2.49$, $p<0.05$) such that Post Hoc revealed measurements of nasal volume collected after Condition B were significantly lower than baseline (see Table 7).

	Acoustic Rhinometry Nasal Volume (cm3)	
**Condition**	**Ave**	**SE**
**Baseline**	6.41	*0.26*
**AA**	6.04	*0.24*
**PAA**	5.76	*0.24*
**HP**	6.09	*0.24*
**DI**	6.02	*0.27*

Table 5. Average Acoustic Rhinometry Measurements per Condition

Nitric Oxide

Nitric oxide measurements from both the nose and exhaled breath were collected at baseline (before exposure) and after the completion of each condition. Main Effects of CONDITION were seen in both nNO ($F(4, 160)=5.26$, $p<0.001$) and feNO ($F(4, 160)=4.54$, $p<0.01$) measurements. Post Hoc analysis revealed a decrease in NO concentration for both nasal and breath measurements in all conditions, not the hypothesized increase indicating respiratory inflammation.

	Nasal Nitric Oxide in ppb	
**Condition**	**Ave**	**SE**
**Baseline**	631.40	*32.84*
**AA**	583.72	*22.98*
**PAA**	540.43	*25.99*
**HP**	593.88	*32.22*
**DI**	599.38	*23.41*

Table 6. Average Nasal Nitric Oxide Measurements per Condition and *p* value compared to Condition B

	Breath Nitric Oxide in ppb	
**Condition**	**Ave**	**SE**
**Baseline**	17.76	*1.93*
**AA**	15.98	*1.16*
**PAA**	12.83	*1.05*
**HP**	16.99	*1.45*
**DI**	16.86	*1.29*

Table 7. Average Breath Nitric Oxide Measurements per Condition and *p* value compared to Condition B

Spirometry

Pulmonary function was measured via spirometry at baseline (before exposure) and after the completion of the target conditions A & B. Analysis revealed no significant differences between conditions for endpoints Forced Volume Capacity (FVC), Forced Exhaled Volume during first second of exhalation (FEV_1), and FEV_1/FVC. All fell within normal limits for age, gender and race.

Nasal Lavage

Nasal lavage was performed at baseline (before exposure) and after the completion of the target conditions A & B. Lavage samples were analyzed for inflammatory markers interleukin-8 (IL-8) and Tumor necrosis factor alpha (TNFa), and Substance p (SubP) and Calcitonin gene-related peptide (CGRP).

Inflammatory markers
While no significant changes were seen in levels of TNFa, IL-8 levels decreased significantly from baseline for both Conditions A and B ($F(2, 80)=12.05$, $p<0.001$) with no significant difference between conditions (see Table 8).

	IL-8 Levels	
**Condition**	**Ave**	**SE**
**Baseline**	1010.34	*148.98*
**AA**	724.87	*91.87*
**PAA**	498.04	*63.91*

Table 8. Average Interleukin 8 (IL-8) levels.

Levels of Substance P increased from baseline for both Conditions AA and PAA ($F(2, 80)=9.33$, $p<0.001$) with no significant difference between conditions (see Table 9).

	Substance P Levels	
**Condition**	**Ave**	**SE**
**Baseline**	2039.1	94.5
**AA**	2332.2	99.5
**PAA**	2349.3	80.1

Table 9. Average Substance P (SubP) levels.

Levels of CGRP decreased from baseline for both Conditions A & B ($F(2, 80)=52.54$, $p<0.001$) with no significant difference between conditions (see Table 10).

	CGRP Levels	
**Condition**	**Ave**	**SE**
**Baseline**	5419.4	398.2
**AA**	3010.3	322.4
**PAA**	2264.5	158.8

Table 10. Average Calcitonin Gene-Related Peptide (CGRP) levels.

Subjective Verbal Ratings

Subjective ratings of odor and irritation intensity were significantly higher during exposure to PAA compared to all other conditions ($F(3, 120)=52.27$, $p<0.001$). This applies to all ratings ($F(9, 360)=6.92$, $p<0.001$) as shown in Table 11.

	Condition							
	AA		PAA		HP		DI	
	Ave	SE	Ave	SE	Ave	SE	Ave	SE
Intensity	12.06	*2.16*	**22.99*** **	*2.90*	8.10	*1.53*	7.26	*1.85*
Eye Irritation	4.80	*1.20*	**13.62*** **	*2.10*	2.71	*0.68*	3.01	*0.78*
Nose Irritation	8.60	*1.64*	**23.78*** **	*2.45*	6.60	*1.52*	5.00	*1.05*
Throat Irritation	4.36	*1.07*	**12.65*** **	*2.05*	3.15	*0.72*	2.65	*0.99*

Table 11. Average Subjective Ratings of intensity and irritation. ****p*<0.001
