# Supplementary material for: Environmental chamber studies of eye and respiratory irritation from use of a peracetic acid–based hospital surface disinfectant
Source: Antimicrob Steward Healthc Epidemiol. 2023 Apr 11;3(1):e71. doi: 10.1017/ash.2023.138 (PMC10127244; doi:10.1017/ash.2023.138)
Supplement: Supplementary file 1 [file ashsup.zip › S2732494X23001389sup002.pdf]

Supplemental Appendix 1 to Dalton et al. "**Environmental Chamber Studies of Eye and Respiratory Irritation from Use of a Peracetic Acid-Based Hospital Surface Disinfectant**"

This appendix contains the following information:

1) Analytical Methods
2) Additional Exposure Characterization Data
3) Hospital Dispenser Calibration Study
4) Hospital Studies
5) Mass Transfer Studies

1. Analytical Methods

Methods for Measuring Airborne Peracetic Acid (PAA), Hydrogen Peroxide (HP), and Acetic Acid (AA)

The first modern-era method for airborne PAA determination was based on peracid oxidation of methyl p-tolyl sulfide (MTS) adsorbed to silica gel sampling media with the extract analyzed by high performance liquid chromatography (HPLC) with UV detection, as described by Di Furia et al. (1984). These investigators showed that the reaction of PAA with MTS to form methyl p-tolyl sulfoxide (MTSO) exhibited good capture efficiencies under standard flow rates (e.g., 1-2 L/min) through MTS-coated silica gel tubes ((Di Furia et al. 1984); however, the presence of other airborne oxidants such as HP could also generate MTSO and thereby interfere with PAA quantification (Christensen et al. 2000; Pinkernell, Effkemann, and Karst 1997). This method is limited by higher-than-desired detection limits for PAA and the inability to separately determine HP when concurrently present in air samples.

Hecht and Hery (2002) reported an alternative method for quantification of airborne PAA, AA, and HP using two trapping agents: titanyl sulfate on silica gel that combines with HP to form H_2TiO_4 and provides a summed quantification of total peroxides; and MTS in an impinger solution that combines with PAA to form MTSO and provides for determination by HPLC with UV detection (Hecht and Hery 2002). This method is limited by higher-than-desired detection limits for PAA and HP, inherent difficulties with handling and shipping the midget impingers/liquid samples, and the desire to use gas chromatography with flame ionization detection (GC/FID) instead of HPLC. Citing the work of Simone (1989), Hecht and Hery (2002) postulated that during the sampling of AA on a Florisil tube, the PAA present in the sample decomposed upon acid extraction to AA so that only a measurement of total acids (PAA + AA) could be made. Therefore, they measured the AA by determining the total acids in the impinger solution and subtracting the PAA as determined by the MTSO measurement.

Hecht et al. (2004) further developed the analytical approach of Hecht and Hery (2002) for simultaneous measurement of HP and PAA; they described a two-piece sampling train wherein oxygen radicals of HP are trapped with two 25 mm quartz fiber filters coated with titanyl oxysulfate, followed by a glass tube containing 600 mg of silica gel coated with sodium carbonate and MTSO (Hecht et al. 2004). The titanyl oxysulfate reaction with HP was found to be rapid and complete on the pre-filters at flow rates of 1-2 L/min, while the slower reaction rate of PAA with titanyl oxysulfate allowed it to pass through the pre-filter (Hecht et al. 2004). This method quantified PAA conversion of MTSO to methyl p-tolylsulfone (MTSOO) on silica gel, which was found to be sufficiently rapid and complete for quantitation (Hecht et al. 2004). The acetonitrile extraction of the silica gel could also be used with the GC/FID to quantify MTSOO and relate the mass to the equivalent mass of PAA. This method has been validated for industrial hygiene studies with standard flow rates of 1 to 2 L/min (Hawley et al. 2017, 2018; Hecht et al. 2004; NIOSH 2018, 2019); however, the method is limited by higher-than-desired detection

limits for PAA and HP when lower total air volumes are collected (e.g., 25 μg/m^3 (20 ppb) for HP and 31 μg/m^3 (10 ppb) for PAA at 20-liter sampling volume).

Nordling et al. (2017) presented a liquid trap HPLC method for measuring PAA with an improved detection limit down to 40 μg/m^3 (13 ppb) for a 20-min sample (Nordling et al. 2017). This method includes sample collection using midget impingers with 15 mL of acetonitrile, 20 mg/L MTS, and 2 mg/L triphenylphosphine oxide (TPPO) used as an internal standard. In this method, a 1 % sodium thiosulfate solution was added to the impinger liquid immediately after sample collection to eliminate any remaining oxidizers (e.g., HP) and stabilize the MTSO for subsequent HPLC analysis with UV spectrophotometric detection at a wavelength of 225 nm (Nordling et al. 2017). This method is limited by not having separate HP and PAA quantitation, higher-than-desired (albeit improved) detection limits for PAA, and inherent difficulties with sample collection, handling, and shipping the midget impingers/liquid samples.

In 2019, a gas chromatography measurement method for PAA was developed (OSHA Method PV2321) with 2-part sample collection system like that of Hecht et al. (2004) (OSHA 2019). OSHA Method PV2321 uses two 25 mm quartz filters impregnated with titanyl oxysulfate to trap and remove the HP in series with a midget impinger containing MTS for quantifying PAA. As in the Hecht et al. (2004) method, HP is scrubbed by titanyl oxysulfate in the pre-filter and PAA entering the midget impinger converts MTS to MTSO which is then analyzed using a DB-5 type capillary gas chromatography column with flame ionization detection using 4-chlorophenyl methyl sulfone as an internal standard. This method is limited by higher-than-desired detection limits for PAA and HP, and difficulties with impinger sampling for breathing zone samples.

Due to the lack of established sampling and analytic methods for PAA, AA, and HP measured simultaneously, along with the need to evaluate these exposures due to their ubiquity in hospital environments, the current study was initiated to fill this evidence gap. The purpose of this study was to develop analytical methods for sampling and analysis of airborne PAA, AA, and HP in an environmental chamber study of eye and respiratory irritation over a 20-minute period during use of a PAA-based hospital surface disinfectant use by human volunteers at the Monell Chemical Senses Center in Philadelphia.

MATERIALS AND METHODS

Environmental Chamber Study of PAA-Based Hospital Surface Disinfectant

This study was contracted by Ecolab, the manufacturer of OxyCide™ and associated equipment for safely dispensing and using this PAA-based chemistry for hospital surface disinfection. This human volunteer study was approved by an Institutional Review Board (Advarra Institutional Review Board; https://www.advarra.com/review-services/institutional-review-board/; protocol number Pro00055123), and written informed consent was provided by each of the healthy volunteers via signature on an Informed Consent Form (ICF), who were compensated for their participation.

Within the Monell environmental chamber, the volunteers performed 8 surface cleaning/ disinfection sessions of 20 minutes duration each throughout the study day. This scenario was designed to represent upper-bound exposures during disinfectant use for hospital surface disinfection. A 20-minute exposure duration was selected as the upper-bound time required to wipe the mattress/bed, bathroom, and other high-touch surfaces during terminal cleaning of a single patient room. The volunteers wore a vest containing a sampling harness used to collect air samples for PAA, AA, and HP for the duration of each cleaning/disinfection session. The sampling and analysis method described in the current study were designed to reliably collect and accurately analyze the air samples taken during each day of the Monell environmental chamber study, comprising up to 76 study days with 8 trials per day with measurement of PAA and HP, and an estimated measurement of AA (as total PAA + AA minus PAA) in each trial (i.e., up to 1,824 individual samples).

Since PAA is considered a more potent irritant than AA or HP (Dalton, Dilks, and Hummel 2006; Ernstgard et al. 2006; NRC 2010; Pechacek et al. 2015) and recent surveys identified hospital 8-hour work shift concentrations as low as 6 to 16 μg/m^3 (2-5 ppb) (Hawley et al. 2017, 2018), the project team considered it important to have an analytical sensitivity of at least 3 μg/m^3 (1 ppb) for PAA measurement during 20-minute exposure trials. As discussed further below, our literature review revealed that none of the methods for PAA quantification met this desired PAA sensitivity goal. This led us to develop an optimized method that would accomplish the sensitivity and selectivity goals for all three analytes (PAA, AA, and HP) for the short-term environmental chamber studies. We also carefully considered the reliability of the available and adaptable methods for sampling and analysis in light of the project parameters and the large number of samples to be collected, including the following:

1. Commercial availability of the sampling media.
2. Reliability of the air sampling pump to limit the frequency of calibration checks and sample losses due to calibration problems or battery pump failures.
3. Sufficiently long shelf life and hold time limits of the sampling media to allow for weekly transfer of sample batches without concerns for sample stability.
4. Sufficiently simple sample collection procedure to optimize the collection of the large number of samples (e.g., eight sets of samples per sampling day).
5. Sufficient robustness of the sampling tubes to avoid breakage, leakage, or other sample losses that can occur during collection, handling, and shipping to the analytical lab; and
6. Equipment and automation capability of the analytical methods to allow for batch processing of a week’s worth of samples (40 sets of samples for PAA, HP, and AA), including extraction of all samples within 48 hours after laboratory receipt and analysis within 36 hours after extraction.

Optimized Methods for the Monell Environmental Chamber Studies

NIOSH Method 1603 was chosen for airborne AA analysis in the current study (NIOSH 1994). Samples were collected on SKC charcoal tubes Anasorb CSC (Coconut Shell Charcoal; SKC

226-01; Eighty Four, PA, USA). Samples were analyzed on a HP 5890 gas chromatograph with a flame ionization detector (HewlettPackard; Palo Alto, CA, USA). The sample was injected on a 1 m x 4 mm ID glass Carbopak B 60/80 mesh/3% Carbowax 20M/0.5 % H_3PO_4 packed column (MilliporeSigma; Burlington, MA, USA). Notably, the Hawley et al. (2017) study reported workshift mean airborne AA concentrations ranging from 14.7 to 386 µg/m^3 (6-157 ppb) with 95th percentile confidence values up to 784 µg/m^3 (319 ppb) in a hospital using a PAA-based surface disinfectant. The analytical sensitivity of NIOSH Method 1603 for short-term sample collection (e.g., 123 µg/m^3 (50 ppb) for 20-liter air volume) was higher than the method detection limit goal for AA at 50 µg/m^3 (20 ppb) or less. However, the goal could be accomplished by increasing the flow rate for sample collection from the standard rate of 1 to 2 L/min up to 4L/min. Validation of this higher sampling rate is described below.

An inherent limitation of NIOSH Method 1603 in this setting was that both PAA and AA were expected to be captured on the charcoal media, and with the possible conversion of PAA to AA on the CSC tube, the resulting measurement would give the total acids (Hecht and Hery 2002) and not solely the AA, which may lead to overstated estimates of airborne AA. Hawley et al. (2017) apparently adjusted their measured AA concentrations (in ppb) using a correction factor of 1.66, although the data justifying this factor were not provided (Hawley et al. 2017, 2018). For our study we thought it reasonable to presume that as a first approximation, subtracting the separately determined airborne PAA concentration from the total AA + PAA concentration determined by NIOSH Method 1603 would give a reasonable approximation of the AA. However, there are no published data to affirm this presumption. As such, we developed a small chamber testing protocol to assess the stability of PAA, AA, and HP on all sample media to establish a minimum of a 2-week (in foil-covered refrigerated storage) hold time without degradation of the peroxide analytes (MTSOO and H_2TiO_4) and to quantitatively assess the capture of both PAA and AA on charcoal media using NIOSH Method 1603.

The selected method for sampling and quantifying airborne HP was OSHA Method 1019, which uses two 25 mm quartz filters impregnated with titanyl oxysulfate (OSHA 2016). The main advantages of this method are that it is widely used and easy to use, and the impregnated filters are commercially available through SKC (225-9030). As previously described by Hecht et al. (2004), the pre-filter can be used in conjunction with the MTSO-impregnated silica gel collection media for the PAA, so that both PAA and HP can be sampled at the same time and separately quantified. The sample is extracted from the filter with concentrated sulfuric acid (2 molar) and analyzed using a spectrophotometer at a wavelength of 410 nm. The NIOSH hospital investigation of PAA-based surface disinfectant exposures by Hawley et al. (2017) reported work shift HP concentrations averaging between 9.8 and 228 µg/m^3 (8-186 ppb) in various hospital locations with 95th percentile values up to 627 µg/m^3 (511 ppb). The project team identified a method detection limit goal of 6 µg/m^3 (5 ppb) or less for the Monell environmental chamber studies, which was not achievable using the standard sampling and analysis procedures of OSHA Method 1019 (25 µg/m^3 (20 ppb) detection limit for 20 L collected sample volume). However, the goal could be accomplished by using the same approach as described for AA and PAA, by increasing the flow rate for sample collection from the standard rate of 1 to 2 L/mine up to 4L/min. The validation of this technique is described below.

The analytical method for PAA was developed to refine certain limitations of two existing methods: OSHA Method PV2321 and the INRS BP27 method described by Hecht et al. (2004). Our objective was to adapt the existing OSHA PV2321 method and incorporate MTSO-coated basic silica gel into our method so that solid media could be used to eliminate the need for liquid impingers during sample collection. The use of a silica gel tube also allowed us to investigate using an increased flow rate to get a lower detection limit during a 20-minute task-based sampling period. As described for the other target analytes, this was done by increasing the air flow rate from 1-2 L/min up to 4 L/min and enhancing the higher flow rate reliability using an electric vacuum pump rather than standard battery-operated pumps. This modification allowed us to accomplish a reliable PAA limit of quantification below 3 $\mu g/m^3$ (1 ppb) for a 20-minute sample duration. The second problem with the OSHA Method PV2321 was that the use of midget impingers for PAA sample collection was impractical for our environmental chamber studies, which collected 8 sets of samples per study day. This was resolved by using the solid sampling media impregnated with MTSO that converts to MTSOO upon contact with PAA, as reported by Hecht et al. (2004). This alternative solid media was also amenable to the use of gas chromatography with flame ionization detection for PAA quantitation, better fitting our project goals for standardizing laboratory equipment and automation. The NIOSH hospital investigation of PAA-based surface disinfectant exposures by Hawley et al. (2017) reported work shift PAA concentrations averaging between 6 and 100 $\mu g/m^3$ (2-32 ppb) in various hospital locations, with 95th percentile values up to149 $\mu g/m^3$ (48 ppb). The project team identified a method detection limit goal of 3 $\mu g/m^3$ (1 ppb) or less for the Monell environmental chamber studies, which was not achievable using the standard sampling and analysis procedures of OSHA Method PV2321.

Laboratory Equipment for Chosen Analytical Methods:

Airborne HP was analyzed in accordance with OSHA Method 1019. This method uses two 25 mm quartz filters coated with titanyl oxysulfate hydrate and preloaded into a 2-piece polystyrene cassette (SKC 225-9030). The sample was extracted from the filter with 2M H_2SO_4 and analyzed using a UV/Visible spectrophotometer (Model 370, Sequoia-Turner, Mountain View, CA, USA) set at 410 nm. The measured oxidation product with titanyl oxysulfate quantified and related to the HP mass for calculating airborne HP concentrations.

Airborne AA was analyzed in accordance with NIOSH Method 1603 without modifications. Samples were collected on Anasorb CSC cartridges (SKC 226-01) and analyzed on a gas chromatograph with flame ionization detector (HP 5890). The cartridge was extracted with 1 mL of formic acid with 0.1 % v/v propionic acid as internal standard, and 5 µL of the sample was injected on a 1 m x 4 mm ID glass Carbopak B 60/80 mesh/3 % Carbowax 20M/0.5 % H_3PO_4 packed column. The AA was quantified directly and the measured AA mass for calculating airborne AA concentrations.

Airborne PAA was determined using a combination of the INRS BP27 method described by Hecht et al. (2004) and OSHA Method PV2321. The sample was drawn through the titanyl oxysulfate filters (SKC 225-9030), which scrubs the airborne HP that is quantified by OSHA Method 1019 described above. The HP-scrubbed air exiting the pre-filters then travels through a

silica gel tube coated with MTSO, which is commercially available from SKC (226-199-UC), where PAA interacts with MTSO to form MTSOO. The MTSOO was extracted from the silica gel tube using 5 mL of acetonitrile. The extract was analyzed using a gas chromatograph (5890, Hewlett Packard) equipped with an RTX-5 column (30 m) (Restek, Bellefonte, PA, USA) and an FID detector as described in OSHA PV 2321. The MTSOO was quantified and related to the PAA mass for calculating airborne PAA.

Acetonitrile used for extraction and preparation of internal standards or other laboratory standards and stock solutions was nanograde purity obtained from Supelco (Bellefonte, PA, USA). Internal standard and analyte chemicals included 4-chlorophenyl methyl sulfone of 98 % purity from Sigma Aldrich (St. Louis, MO, USA); MTSOO of 99 % purity from TCI; glacial acetic acid of 99 % purity, formic acid of > 95 % purity, propionic acid of > 99 % purity, and titanyl oxysulfate hydrate of 27-31 % purity were from Sigma Aldrich.

Sampling Methods and Equipment Customized to the Monell Environmental Chamber Studies

The Monell environmental chamber configuration is shown in Figure 1-1 (dimensions: 2.9 m wide x 3.6 m long x 2.2 m height). The chamber was equipped to allow for control and monitoring of supply air, exhaust air, and temperature using an air-controlling system (Siemens, Berlin, Germany). For the cleaning sessions, the temperature was set to 21 °C, the set point for supply air was 1.7 m^3/min, and exhaust was 2.0 m^3/min, which produced a negative flow. This setting equates with fresh air exchange rate of 6 air changes per hour. At the beginning of each testing day, the chamber was set to "test" mode for at least 30 min before testing to ensure that the room had reached its set points. At the end of each day the chamber was placed in "purge" mode, which flushed the room with 8.5 m^3/min of air for 20 min before placing the room in "non-testing" mode.

The chamber contained a series of hospital patient room and bathroom items considered to be "high-touch surfaces" with an estimated surface area of 8.5 m^2 to be cleaned using the OxyCide™ disinfectant (Ecolab, Saint Paul, MN, USA). All air samples were collected from the breathing zone of the individual who was conducting the cleaning. To allow for breathing zone air sample collection for individual volunteers performing disinfection/cleaning of these "high-touch surfaces" within a relatively small chamber space, a customized set of sampling equipment was developed including 1) a 4-channel sample collection manifold connected to an electric vacuum pump; 2) a customized sampling vest for holding up to 4 sampling lines; and 3) a retractable tether system to keep the Tygon tubing organized.

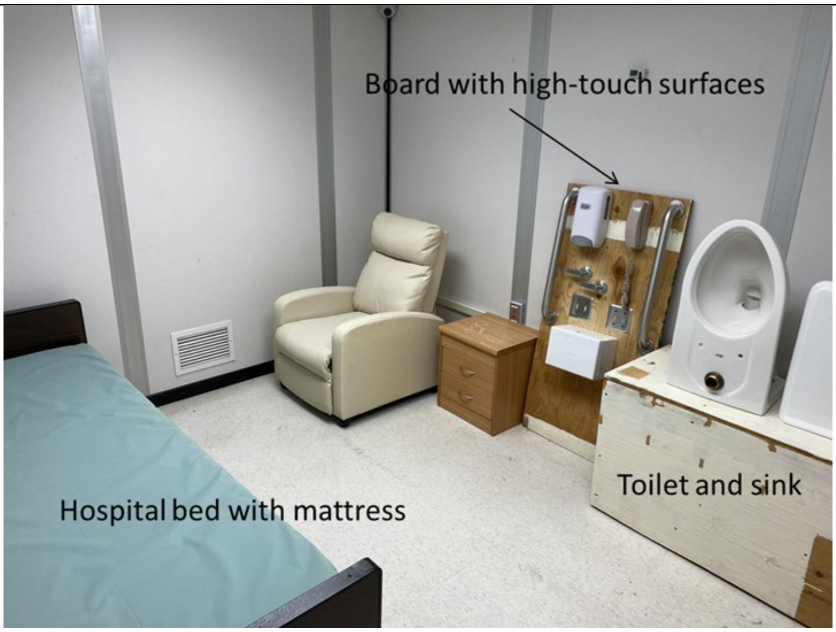

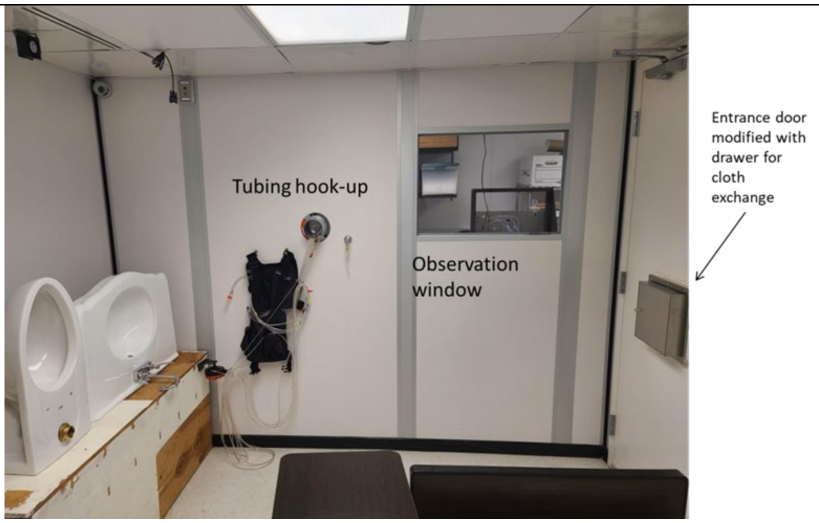

Figure 1-1. Environmental chamber configuration with furniture and high-touch surfaces, sample tubing hook-up, and chamber entrance door, which was modified with a drawer for cloth exchange.

The sample collection manifold (Figure 1-2) was constructed with four Key Instrument rotameters (Brooks Instrument; Hatfield, PA, USA) with a flow rate range of 0.4 to 5 liters per minute and a Gast DOA P707-AA vacuum pump (Gast; USA). The electric vacuum pump was rated to be capable of generating 31 liters per minute of vacuum flow and so was more than sufficient for generating 4 liters per minute across the 4 channels. The manifold was tested over a 20-minute sample period in a room chamber at EAS with the sample cartridges connected to the manifold with 7.6 m of Tygon tubing. During beta testing at EAS, the sample cartridges were attached to a stand in duplicate pairs. The flow was set to 4 liters per minute on the rotometers

and checked with a DryCal flow test meter (Lakewood, CO, USA). The manifold was able to maintain a flow of 4 L/min over the 20-minute period with no measurable change in air flow.

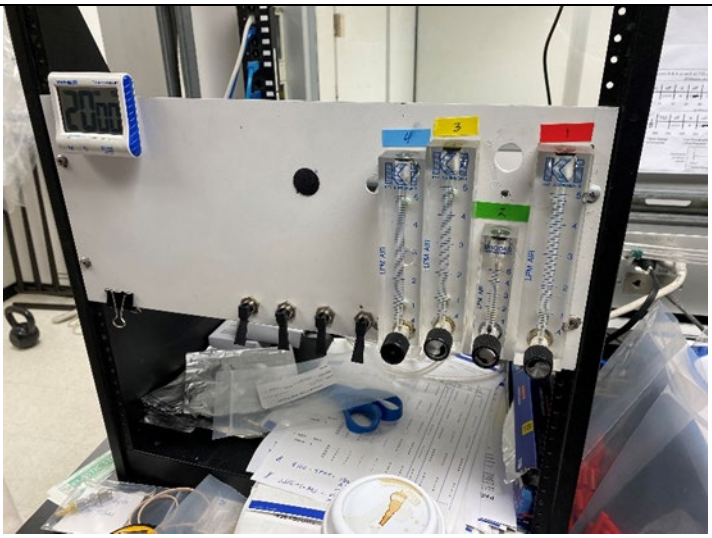

Figure 1-2. Sample collection manifold constructed with four Key Instrument Rotometers and a Gast DOA P707-AA vacuum pump.

Tygon tubing was run from the 4-channel sample collection manifold to the breathing zone of the volunteer and connected to the sample cartridges on a customized sampling vest, as shown in Figure 1-3. The sampling vest was set up to hold up to four cartridges with separate vacuum flow lines at shoulder level to obtain breathing zone air samples for each volunteer during the 20-minute disinfection/cleaning task.

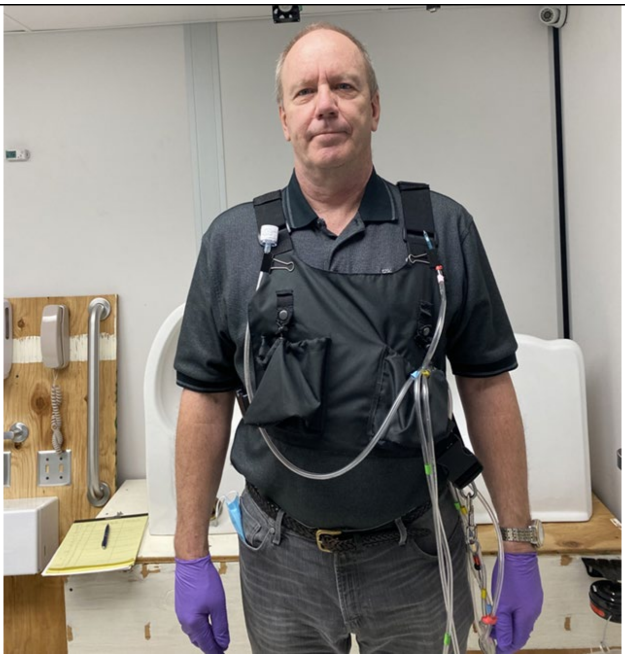

Figure 1-3. Customized sampling vest, with Tygon tubing from the 4-channel sample collection manifold to the breathing zone of the volunteer and connected to the sample cartridges.

A retractable elastic tether line was attached to a section of the Tygon tubing about 0.9 m from the waist-level attachment point on the back of the sampling vest and a spindle on the back wall of the Monell environmental chamber; this elastic tether line helped to keep the Tygon tubing lines organized and prevent volunteers from stepping on or tripping over the lines.

This equipment was beta-tested at EAS during a series of small-room and small-chamber studies and was subsequently installed at Monell for use in the environmental chamber studies.

During the cleaning sessions in the Monell chamber, study participants were instructed to wipe down the items in the room (hospital bed, toilet, sink, chair, tables, and high-touch objects), but not the floors, walls, or ceiling. The process of "wiping down" was described to the study participants as "leaving a wet layer of cleaner on the items" to evenly distribute the cleaner around the room rather than scrubbing, cleaning, or unnecessarily devoting time to a single item or part of the room.

For each 20-minute cleaning session, study participants were escorted into the chamber and provided help (if needed) placing on the sampling vest. Once the was secured, the Monell researcher left the room, closed the door, and placed a pair of nitrile gloves in the pass-through door (located on the chamber entrance door) for the participant. To simulate the typical use of the cleaning cloths, one cloth at a time was used by the volunteer, with the container of other wetted cloths located outside of the room. Each time an item (cloth) was placed in the pass-through door, researchers notified the volunteer to retrieve the new wetted cloth and to return the used one. After the participant placed the gloves on, the first pre-soaked cloth was provided via the pass-through door and the time clock was started. Participants were provided additional pre-soaked cloths after minutes 5, 10, and 15 via the pass-through door.

Validation of Measurement Method Performance at 4 Liter per minute Flow Rate

The proposed use of a 4-L/min flow rate for our sampling and analysis methods cannot be reliably obtained using standard battery-operated vacuum pumps commonly used by industrial hygienists in field studies. The use of an electric vacuum pump capable of generating multiple channels of flow at 4 L/min was both feasible for the Monell environmental chamber studies and offered greater flow reliability and easier calibration checking compared with use of battery-operated pumps. However, none of the selected methods for PAA, AA, or HP had been validated at this higher flow rate. Thus, we carried out a validation study that evaluated the lower- and upper-bound flow rates for battery-operated pumps (1 to 2L/min) in side-by-side or paired determinations at 4 L/min for air samples obtained from a static small room study at Environmental Analytical Services (EAS) laboratory of the PAA-based hospital surface disinfectant use.

To investigate the possible impact of increasing the air sampling rate from 1 or 2 L/min to 4L/min, a comparison experiment was conducted as follows. The PAA-based disinfectant was mixed at ready-to-use concentration (3 fluid ounces of concentrate per gallon of water; 23.4 mL concentrate per L of water) and applied to microfiber cloths following the product manufacturer's recommended protocol for preparation and surface disinfection. The EAS small room chamber (2.4m long x 3 m wide x 2.4 m height) with no active ventilation was set up with a 0.6 x 1.2 m stainless steel tabletop used as the disinfection target surface. Up to two stands were set up with duplicate sampling trains for side-by-side measurement of airborne PAA, AA, and HP at calibrated flow rates of 1 and 4 L/min or 2 and 4 L/min on each stand. The sampling devices were positioned adjacent to the tabletop edge at approximately 46 cm above the tabletop surface to simulate the breathing zone height of a person cleaning the target surface. Two disinfectant cloths wetted with fresh PAA-based disinfectant solution were placed simultaneously on the tabletop and moved around intermittently. This study employed fixed-location area sampling, as it was not intended to simulate personal exposure during normal product use. Samples were collected using the sample collection manifold and vacuum pump, as described above, in a series of separate sampling events until the desired number of paired samples were obtained: 6 paired samples for comparison of airborne concentration results at 2 and 4L/min, and 4 paired samples for comparison of airborne concentration results at 1 and 4L/min. Fewer samples were collected at the 1-L/min flow rate because Christensen et al. (2000) reported that flow rates at or below this rate may lead to falsely elevated HP determination due to PAA interaction with titanyl oxysulfate in OSHA Method 1019.

Determination of Detection Limit and Reporting Limit for PAA

A method detection limit (MDL) study was performed where the MDL was calculated at the 99 % confidence level from seven repetitive measurements on a sample whose concentration did not exceed 10 times the estimated MDL (Glaser et al. 1981; Long and Winefordner 1983). To calculate the MDL, a sample is prepared in the appropriate matrix with components at approximately 10 times the estimated MDL. This sample is run seven consecutive times and the standard deviation (SD) is calculated. The MDL is determined by multiplying the SD by 3.00, which is the Student t-value for n=7.

Potential Impact of Storage Hold Time Before Extraction on AA, HP and PAA Measurements

Samples for PAA, AA, and HP were collected in duplicate pairs, until 12 samples were obtained in sequential disinfection simulations where two wetted microfiber cloths were simultaneously applied to the stainless-steel table and intermittently moved across the surface. Each sample set was collected for 20 minutes at the 4-L/min flow rate, and then each sample was capped, covered with aluminum foil, and put in a sealable bag such that three designated samples were generated for refrigerated storage for 0, 3, 6, or 14 days prior to extraction and analysis. Comparison of the results obtained for samples extracted on day 0 to those stored for 3, 6, or 14 days determined whether any appreciable degradation of quantitation occurred during these storage/hold intervals.

Statistical Analysis Methods

For the analysis of air samples collected during simulated PAA-based disinfectant use in the EAS small room chamber at 0, 3, 6, or 14 days, an analysis of variance (ANOVA) model was implemented for each analyte to test for differences in the average concentrations over time. Dunnett's test was utilized to compare the mean concentrations at 3, 6, or 14 days with day zero. A simple regression model was used to determine if there was a relationship of hold time with the ratio of the mean concentration by the average at day zero. Statistical significance was assessed at an alpha of 0.05. All analyses were performed in SAS v9.4.

RESULTS

Overview of Modified Methods for PAA, HP, and AA

The methods utilized for airborne HP and AA analysis followed the standard operating procedures for OSHA Method 1019 and NIOSH Method 1603, respectively, with an increased sample flow rate of 4 liters/minute to obtain a lower air concentration detection limit. For HP determination using OSHA Method 1019 and the 4-L/min sample collection flow rate, the method detection limit was determined to be 2.76 µg/m^3 (2.25 ppb), and the laboratory reporting limit was 4.69 µg/m^3 (3.82 ppb). For AA determination using NIOSH Method 1603 and the 4-L/min sample collection flow rate, the method detection limit was determined to be 34.6 µg/m^3 (14.1 ppb), and the reporting limit was 65.1 µg/m^3 (26.5 ppb). Since PAA is also present in the chamber, this method measures the total acids (PAA + AA).

PAA and HP Determination using Simultaneous Sampling and Gas Chromatography/Flame Ionization Detection (GC/FID)

The simultaneous sampling of PAA and HP used the SKC sampling media designed for the simultaneous collection of PAA and HP, which was based on Hecht et al. (2004), who used a filter cartridge preloaded with two filters coated with titanium oxysulfate to react with the HP and a basic silica gel tube coated with MTSO. The HP was extracted and analyzed by OSHA Method 1019.

As previously discussed, modification of the method published by Hecht et al. (2004) and by OSHA (2019), Method PV2321 includes both an increase in sample collection air flow rate to 4 L/min and the determination of MTSOO by gas chromatography.

Determination of MTSOO by GC/FID

The determination of MTSOO by gas chromatography under our modified method provided well-resolved and easily quantifiable peaks (Figure 1-4) for the trapping agent (MTSO), the oxidized MTSO generated from interaction with PAA (MTSOO), and the internal standard compound that was added to the extraction solvent (4-chlorophenyl methyl sulfone. All of the data generated for standard curves and other quality control and blank samples performed appropriately within the standard operating procedures used for the method.

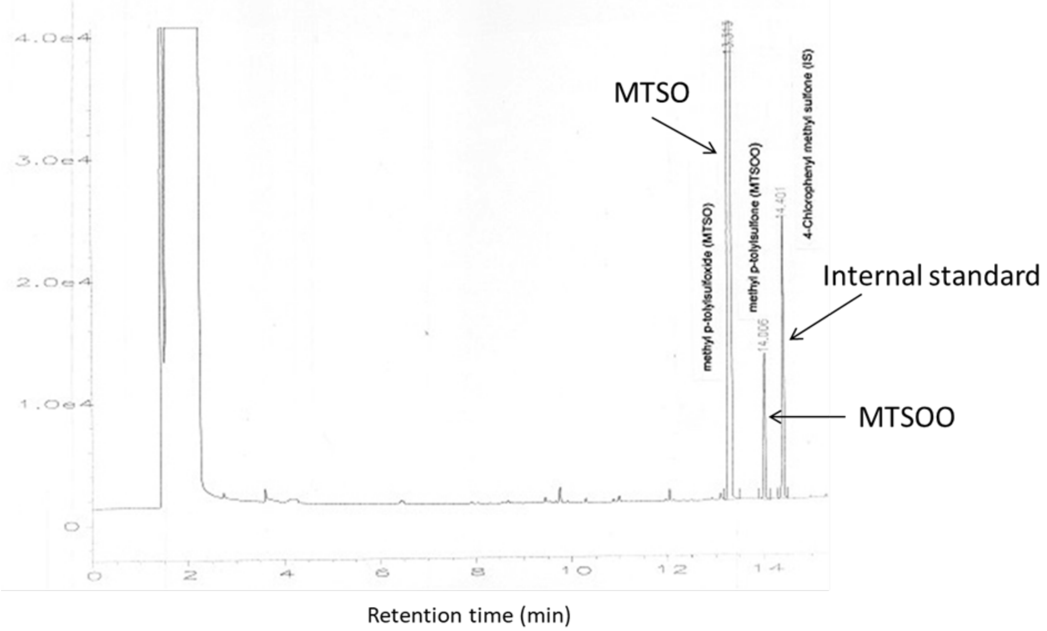

Figure 1-4. Gas chromatogram of standard solution for MTSO, MTSOO, and internal standard (4-chlorophenyl methyl sulfone). The modified methods provided well-resolved and easily quantifiable peaks.

Initial Calibration Curve for MTSOO by GC/FID

A calibration curve was prepared for the GC/FID method by diluting the stock standard solution to prepare standards for the levels shown in Table 1-1. The relative response factor (RRF) was calculated according to the following equation:

$$RRF = \frac{Concentration\ of\ Standard\ \left(\frac{\mu g}{mL}\right) * (Area\ of\ Internal\ Standard)}{Area\ of\ Standard}$$

The low calibration point on the curve is used as the value for the reporting limit (RL), and is 0.045 µg/ml or 627 $\mu g/m^3$ (0.90 ppb) with an air flow of 4L/min. The relative standard deviation for the RRF values for the curve in Table 1 was 22.7%. Several calibration curves were prepared during the study, and the variability across 3 separate calibration curve showed RSD values of 22.7 %, 33.5 %, and 29.8 % with an average RSD of 28.7 %.

Table 1-1. PAA (MTSOO) Example Calibration Curve for GC/FID

PAA Standard (µg/mL)	RRF
0.045	1.30
0.089	2.08
0.447	2.90
0.893	2.37
1.787	2.66
2.68	2.77
4.47	2.52
8.93	2.62
**Average RRF**	2.40
**RSD %**	22.7

Determination of Detection Limit and Reporting Limit for PAA

The results of the method detection limit study are shown in Table 1-2. The detection limit is reported as µg/ml and µg/sample, based on the sample extraction volume of 5 mL acetonitrile. The sampling time for the project was 20 minutes based on the amount of time the volunteer was set to perform simulated hospital disinfection of high-touch surfaces in the chamber. The detection limit is also reported in µg/m^3 and parts per billion (ppb) at three different flow rates. To make sure that measurable concentrations of PAA are obtained for all of the study cleaning conditions, a detection limit below 1 ppb was desired. To obtain this, a flow rate of 4 L/min was necessary.

Table 1-2. Detection Limits for PAA at Different Flow Rates

Air Flow (L/min)			1.0	2.0	4.0
Duration (min)			20	20	20
	Concentration		Concentration		
Sample number	µg/mL	µg/sample	µg/m^3 (ppb)	µg/m^3 (ppb)	µg/m^3 (ppb)
C04261A	0.0934	0.47	23.4 (7.51)	11.7 (3.75)	5.8 (1.88)
C04261B	0.1077	0.54	26.9 (8.66)	13.5 (4.33)	6.7 (2.16)
C04261C	0.0983	0.49	24.6 (7.9)	12.3 (3.95)	6.2 (1.98)
C04261D	0.0966	0.48	24.1 (7.76)	12.1 (3.88)	6.0 (1.94)
C04261E	0.0909	0.45	22.7 (7.31)	11.4 (3.65)	5.7 (1.83)
C04261F	0.1153	0.58	28.8 (9.27)	14.4 (4.63)	7.2 (2.32)

C04261G	0.1032	0.52	25.8 (8.29)	12.9 (4.15)	6.4 (2.07)
C04261H	0.1071	0.54	26.8 (8.61)	13.4 (4.3)	6.7 (2.15)
Average	0.102	0.509	25.4 (8.16)	12.8 (4.08)	6.3 (2.04)
SD	0.0082	0.0412	2.1 (0.66)	1.0 (0.33)	0.5 (0.17)
MDL	0.026	0.128	6.4 (2.1)	3.2 (1.03)	1.6 (0.51)

Validation of Measurement Method Performance at 4 Liter per minute Flow Rate

The methods utilized here for airborne HP and AA determination followed the standard operating procedures developed for the project, with an increased sample flow rate to 4 L/min instead of the method flow rate of 1 L/min. The simultaneous sampling of PAA with HP used a flow rate of 4 L/min instead of the 1 to 2 L/min used by Hecht et al. (2004).

Since the airborne concentrations of AA, HP, and PAA in the Monell chamber study were each expected to be well below 1 ppm, the flow rate change from 2 L/min to 4 L/min was not expected to alter the capture efficiency. As previously described, to verify this expectation, small room chamber air samples were collected during disinfectant use at flow rates of 1, 2, and 4 L/min for PAA, HP, and AA only. (The AA-only results were calculated in Table 3 by subtracting the PAA concentration in ppb from the total PAA + AA concentration in ppb.) Results for the paired sample determinations at 1 and 4 L/minor at 2 and 4 L/min are summarized in Table 1-3. Increasing the sample flow rate allowed for collection of a larger air volume and mass of chemical on the sample filter or cartridge for PAA and HP collected in series, and for AA collected in a separate tube. The results in Table 3 indicate that measured airborne concentrations of AA only, HP, or PAA showed average relative percent deviation (RPD) within the standard range of analytical variability (± 20-30 %) for pairwise comparisons of 1 vs. 4L/min, 2 vs. 4L/min, and for all data combined.

Table 1-3. Pairwise Comparison of the 4 liters per minute Sample Collection Rate to Alternative Rates of 1 or 2 liters per minute for Measured AA, HP and PAA

Sample Code	Flow Rate (L/min)	PAA + AA (μg/m³;ppb)	AA Only (μg/m³; ppb)	Pair Average (μg/m³; ppb)	Pair RPD (%)	HP (μg/m³; ppb)	Pair Average (μg/m³; ppb)	Pair RPD (%)	PAA (μg/m³; ppb)	Pair Average (μg/m³; ppb)	Pair RPD (%)
1-4A	1	2216 (398)	734 (299)	661 (269)	-18.4	63 (51.6)	58 (47.55)	-16.9	306 (98.4)	296 (95.2)	-6.7
1-4B	4	1843 (331)	587 (239)			53 (43.5)			286 (92)		
1-4C	1	2822 (507)	813 (331)	846 (345)	2.4	79 (64.5)	70 (57.3)	-25.4	548 (176)	524 (169)	-9.1
1-4D	4	2889 (519)	879 (358)			61 (50)			500 (161)		
1-4E	1	2750 (494)	769 (313)	716 (292)	-21	166 (136)	172 (140)	6.9	562 (181)	484 (156)	-32.6

1-4F	4	2227 (400)	663 (270)			178 (145)			405 (130)		
				**Average RPD (%)**	-12.3		**Average RPD (%)**	-11.8		**Average RPD (%)**	-16.1
**Sample Code**	**Flow Rate (L/min)**	**PAA + AA (µg/m³; ppb)**	**AA Only (µg/m³; ppb)**	**Pair Average (µg/m³; ppb)**	**Pair RPD (%)**	**HP (µg/m³; ppb)**	**Pair Average (µg/m³; ppb)**	**Pair RPD (%)**	**PAA (µg/m³; ppb))**	**Pair Average (µg/m³; ppb)**	**Pair RPD (%)**
2-4A	2	1119 (201)	400 (163)	484 (197)	26.3	130 (106)	122 (99.6)	-12.2	118 (37.8)	107 (34.3)	-20.6
2-4B	4	1458 (262)	567 (231)			115 (94)			96 (30.8)		
2-4C	2	952 (171)	329 (134)	319 (130)	-6.6	110 (89.4)	115 (93.5)	8.7	114 (36.5)	109 (35.2)	-7.4
2-4D	4	891 (160)	309 (126)			120 (97.6)			105 (33.9)		
2-4E	2	924 (166)	324 (132)	397 (162)	24.8	90 (73.2)	107 (87.4)	32.6	105 (33.9)	87 (28)	-40.9
2-4F	4	1186 (213)	469 (191)			125 (102)			69 (22.3)		
2-4G	2					95 (77.2)	114 (92.5)	33	100 (32.3)	87 (28)	-31.1
2-4H	4					132 (108)			73 (23.6)		
2-4I	2					95 (77.2)	102 (83.3)	14.6	97 (31.1)	90 (28.9)	-15.2
2-4J	4					110 (89.4)			83 (26.7)		
2-4K	2					115 (93.5)	117 (95.6)	4.3	76 (24.4)	88 (28.2)	27
2-4L	4					120 (97.6)			100 (32)		
				**Average RPD (%)**	14.8		**Average RPD (%)**	13.5		**Average RPD (%)**	-14.7
	**Average (all data)**	1770 (318)	570 (232)	**Average RPD (AA; %)**	-0.7		**Average RPD (HP; %)**	3.4		**Average RPD (PAA; %)**	-15.3

Evaluation of Storage/Hold Time Before Extraction

A storage/hold time study was conducted using a similar paired-sample collection approach to obtain 12 air samples during simulated PAA-based disinfectant use in the EAS small room chamber, and triplicate groups of samples were extracted at 0, 3, 6, or 14 days after collection and storage using the same refrigerated sample storage approach as designated in the Monell environmental chamber studies. The results for these triplicate samples are shown in Table 4. The triplicate sample means for samples extracted on day 3, 6, or 14 did not differ significantly from that observed for day zero samples, and there was no evidence of a significant downward trend in the ratio of triplicate mean values versus day zero mean with increasing length of storage/hold time for any of the analytes (Table 1-4). AA only was again calculated by subtracting the PAA (in ppb) from the total PAA + AA in ppb. The results in Table 4 also indicate that measured airborne concentrations for each analyte showed acceptably low RSD for

all data combined (RSD % for n = 12 was 26.2 % for AA only, 21.4 % for HP, and 17.8 % for PAA).

Table 1-4. Comparison of Airborne AA, HP and PAA from Disinfectant Use in a Small Room Chamber with Extraction After 0, 3, 6, or 14 days of Refrigerated Holding Time

Sample Code	Hold Time (days)	PAA + AA (μg/m³; ppb)	AA only (μg/m³; ppb)	HP (μg/m³; ppb)	PAA (μg/m³; ppb)
B11	0	1509 (271)	506 (206)	78 (63.2)	199 (64.1)
B12	0	1776 (319)	644 (262)	103 (84.3)	178 (57.2)
B21	0	1965 (353)	698 (284)	81 (65.9)	215 (69.1)
	Mean	1750 (314)	616 (251)	87 (71.1)	197 (63.5)
	SD	229 (41.2)	99 (40.2)	14 (11.5)	19 (6.0)
	RSD %	13.1	16.0	16.1	9.4
Sample Code	Hold Time (days)	PAA + AA (μg/m³; ppb)	AA only (μg/m³; ppb)	HP (μg/m³; ppb)	PAA (μg/m³; ppb)
B22	3	2499 (449)	928 (378)	58 (47.4)	220 (70.7)
B31	3	1737 (312)	594 (242)	71 (58)	216 (69.4)
B31	3	2182 (392)	818 (333)	103 (84.3)	185 (59.6)
	Mean	2139 (384)	780 (318)	78 (63)	207 (67)
	SD	383 (68.8)	170 (69.3)	23 (19)	19 (6.1)
	RSD %	17.9	21.8	30.0	9.1
	Ratio to Day 0 mean	1.2	1.3	0.9	1.0
Sample Code	Hold Time (days)	PAA + AA (μg/m³; ppb)	AA only (μg/m³; ppb)	HP (μg/m³; ppb)	PAA (μg/m³; ppb)
B41	6	1119 (201)	391 (159)	91 (73.8)	129 (41.5)
B42	6	1169 (210)	425 (173)	65 (52.7)	113 (36.4)
B51	6	1536 (276)	526 (214)	71 (58)	193 (62)
	Mean	1275 (229)	447 (182)	75 (62)	145 (47)
	SD	228 (41.0)	70 (28.6)	13 (11.0)	42 (13.6)
	RSD %	17.9	15.7	17.8	29.1
	Ratio to Day 0 mean	0.73	0.73	0.86	0.73
Sample Code	Hold Time (days)	PAA + AA (μg/m³;	AA only	HP (μg/m³;	PAA

		ppb)	(µg/m^3; ppb)	ppb)	(µg/m^3; ppb)
B52	14	1486 (267)	508 (207)	81 (65.9)	189 (60.8)
B61	14	1692 (304)	609 (248)	116 (94.8)	174 (56)
B62	14	1525 (274)	521 (212)	103 (84.3)	194 (62.5)
	Mean	1568 (282)	546 (222)	100 (82)	186 (60)
	SD	109 (19.7)	55 (22.4)	18 (14.6)	10 (3.4)
	RSD %	7.0	10.1	17.9	5.6
	Ratio to Day 0 mean	0.90	0.89	1.15	0.94
All data	Mean	1681 (302)	587 (243)	85 (69)	184 (59)
	SD	395 (71)	157 (63.8)	18 (14.8)	33 (10.5)
	RSD %	23.4	26.2	21.4	17.8

DISCUSSION

The sampling and analysis approach developed here overcomes key limitations in earlier published methods centered on selectivity and sensitivity for simultaneous quantitation of air samples containing the two reactive and unstable peroxides, PAA and HP. In this study, existing methods for airborne analysis of PAA, AA, and HP were evaluated and modified to enable lower detection limits to ensure quantitative results on short-term (20-minute) samples collected in controlled environmental chamber studies. Reliable quantitative results resulting in measurable numbers are important in this type of study so that short-term personal exposures to these chemicals can be accurately related to the measured responses. Increasing the sample flow rate to 4 L/min instead of the 1 to 2 L/min used in published standard methods yielded a lower detection limit for the samples, without producing a significant change in the measured concentrations. The simultaneous sampling of PAA and HP was accomplished using only solid media, thereby avoiding high risks of sample loss during shipping and handling of impinger solutions. Airborne AA was quantified by determining total acids (PAA + AA) and subtracting the concurrently measured PAA.

The data collected on the refrigerated storage of samples for up to 2 weeks before extraction showed no apparent degradation of these analytes which suggests a minimum of a 2-week storage/holding time for the project

The methods overall were determined to be sensitive and reproducible for evaluating short-term exposures to PAA (MDL =1.6 µg/m^3 or 0.5 ppb), HP MDL =2.8 µg/m^3 or 2.3 ppb) and AA (MDL =34.4 µg/m^3 or 14 ppb) during hospital use of PAA-based surface disinfectants.

These methods evaluated for the sampling and analysis were applied to a human volunteer study of ocular and respiratory tract irritation responses from use of a PAA-based solution for sanitizing nonporous surfaces commonly found in hospital patient rooms for sampling and analysis of hundreds of individual samples per week using well established commercial laboratory shipping and processing methods. This methodology may be useful for any short-term measurement scenario where airborne PAA and HP may be simultaneously present.

REFERENCES

Christensen, C.S., S. Brodsgaard, P. Mortensen, K. Egmose, and A. Linde. 2000. "Determination of Hydrogen Peroxide in Workplace Air: Interferences and Method Validation." *Journal of Environmental Monitoring* 2 (4):339-43. doi: 10.1039/b001799j.

Dalton, Pamela, Daniel Dilks, and Thomas Hummel. 2006. "Effects of Long-Term Exposure to Volatile Irritants on Sensory Thresholds, Negative Mucosal Potentials, and Event-Related Potentials." *Behavioral Neuroscience* 120 (1):180-7. doi: 10.1037/0735-7044.120.1.180.

Di Furia, Fulvio, Maurizio Prato, Ugo Quintily, Susanna Salvagno, and Gianfranco Scorrano. 1984. "Gas-Liquid Chromatographic Method for the Determination of Peracids in the Presence of a Large Excess of Hydrogen Peroxide." *Analyst* 109 (8):985-7. doi: 10.1039/AN9840900985.

Ernstgard, Lena, Anders Iregren, Bengt Sjogren, and Gunnar Johanson. 2006. "Acute Effects of Exposure to Vapours of Acetic Acid in Humans." *Toxicology Letters* 165 (1):22-30. doi: 10.1016/j.toxlet.2006.01.010.

Glaser, John, Denis Foerst, Gerald McKee, Stephan Quave, and William Budde. 1981. "Trace Analyses for Wastewaters." *Environmental Science and Technology* 15 (12):1426-35. doi: 10.1021/es00094a002.

Hawley, Brie, Megan Casey, Mohammed Abbas Virji, Kristin Cummings, Alyson Johnson, and Jean Cox-Ganser. 2017. "Respiratory Symptoms in Hospital Cleaning Staff Exposed to a Product Containing Hydrogen Peroxide, Peracetic Acid, and Acetic Acid." *Annals of Work Exposures and Health* 15 (62):28-40. doi: 10.1093/annweh/wxx087.

Hawley, Brie, Megan Casey, Mohammed Abbas Virji, Kristin Cummings, Alyson Johnson, and Jean Cox-Ganser. 2018. "Erratum to Hawley et al. 2017." *American Journal of Infection Control* 46:599. doi: 10.1016/j.ajic.2018.03.017.

Hecht, G. , and M. Hery. 2002. "Generation of Controlled Atmospheres for the Determination of the Irritant Potency of Peroxyacetic Acid." *Annals of Occupational Hygiene* 46 (1):89-96. doi: 10.1093/annhyg/mef010.

Hecht, G., M. Hery, G. Hubert, and I. Subra. 2004. "Simultaneous Sampling of Peroxyacetic Acid and Hydrogen Peroxide in Workplace Atmospheres." *Annals of Occupational Hygiene* 48 (8):715-21. doi: 10.1093/annhyg/meh067.

Long, Gary, and J. Winefordner. 1983. "Limit of Detection: A Closer Look at the IUPAC Definition." *Analytical Chemistry* 55 (7):712A-24A. doi: 10.1021/ac00258a001.

NIOSH. 1994. "NIOSH Method 1603, Issue 2." In *NIOSH Manual of Analytical Methods (NMAM), Fourth Edition*.

NIOSH. 2018. "Evaluation of Exposure to a New Cleaning and Disinfection Product and Symptoms in Hospital Employees; Report No. 2015-0053-3269." In.

NIOSH. 2019. "Evaluation of Exposure to a Hydrogen Peroxide, Peracetic Acid, and Acetic Acid-Containing Cleaning and Disinfection Product and Symptoms in Hospital Employees; Report No. 2017-0114-3357." In.

Nordling, John, Owen Kinsky, Magdalena Osorio, and Nathan Pechacek. 2017. "Description and Evaluation of a Peracetic Acid Air Sampling and Analysis Method." *Toxicology and Industrial Health* 33 (12):922-9. doi: 10.1177/0748233717739165.

NRC. 2010. "Acute Exposure Guideline Levels for Selected Airborne Chemicals: Volume 8." In. Washington DC: National Research Council.

OSHA. 2016. "OSHA Method 1019: Hydrogen Peroxide." In.

OSHA. 2019. "OSHA Method PV2321: Peracetic Acid." In.

Pechacek, Nathan, Magdalena Osorio, Jeff Caudill, and Bridget Peterson. 2015. "Evaluation of the Toxicity Data for Peracetic Acid in Deriving Occupational Exposure Limits: A Minireview." *Toxicology Letters* 233 (1):45-57. doi: 10.1016/j.toxlet.2014.12.014.

Pinkernell, Ulrich, Stefan Effkemann, and Uwe Karst. 1997. "Simultaneous HPLC Determination of Peroxyacetic Acid and Hydrogen Peroxide." *Analytical Chemistry* 69 (17):3623-7. doi: 10.1021/ac9701750.

2. Additional Exposure Characterization Data

Table 2-1. Summary of Measurements for Concentrates A, B, C, and D Prepared and Validated by Ecolab. Ecolab staff produced each of the 4 concentrates (OxyCide^{TM}, AA only, HP only, and deionized water) and validated the final mixture concentrations of PAA, AA, and HP

Concentrations of Solutions	Density (g/mL)		Titration 5/25/2021			Titration results 5/28/2021		Titration results 6/15/2021		Final Accepted Values	
	Lot 1	Lot 2	Lot 1	Lot 2	Target	Lot 1	Lot 2	Lot 1	Lot 2	Lot 1	Lot 2
			Mass %	Mass %	Mass %	Mass %	Mass %	Mass %	Mass %	Mass %	Mass %
**Solution B Concentrate: Peracetic Acid (PAA), Hydrogen Peroxide (HP), Acetic Acid (AA)**	**1.127**	**1.128**									
**PAA**			**5.40**	**5.30**	**5.73**	**5.73**	**5.76**	**5.733**	**5.759**	**5.73**	**5.76**
**HP**			**26.8**	**26.9**	**27**	**27.3**	**27.1**			**27.3**	**27.1**
**AA**			**6.8**	**6.8**	**6.8**					**6.8**	**6.8**
**Solution A Concentrate: Acetic Acid Only**	**1.011**	**1.009**									
**PAA**			**NA**	**NA**	**0**	**<0.001**	**<0.001**			**0**	**0**
**HP**			**NA**	**NA**	**0**	**NA**	**NA**			**0**	**0**
**AA**			**6.8**	**6.8**	**6.8**	**6.8**	**6.8**			**6.8**	**6.8**
**Solution C Concentrate: Hydrogen Peroxide Only**	**1.107**	**1.103**									
**PAA**			**NA**	**NA**	**0**	**<0.001**	**<0.001**			**0**	**0**
**HP**			**27.0**	**27.0**	**27.0**	**27.0**	**27.0**			**27.0**	**27.0**
**AA**			**NA**	**NA**	**0**	**NA**	**NA**			**0**	**0**
**Method QATM 317 was used for the titrations performed on 5/25/21 and 5/28/21. On 6/15/21 a potentiometric titration was used.**											

Table 2-2. Studies to Determine Cloth Saturation for OxyCide™ Use Solution. The Ecolab training for OxyCide™ use indicated that the wetted cloths should be saturated but not dripping and/or accumulating liquid at the bottom of the bucket. Preliminary studies at EAS laboratories identified a cloth saturation point at approximately 125-135 mL per cloth to achieve the Ecolab training guideline of "saturated but not dripping". The cloth saturation level of 125 mL/cloth was selected for use in the environmental chamber studies

Trial #	Solution Volume Applied (mL/cloth)	Cloths were completely wetted?	Volume of Residual Drippage (mL)
Trial 1	175	YES	30
Trial 2	150	YES	5
Trial 3	145	YES	2
Trial 4	140	YES	2
Trial 5	135	YES	0
Trial 6	125	YES	0
Trial 7	100	YES	0
[1]OxyCide™ solution at 3 oz/gal applied to Ecolab Microfiiber Wipes 35 cm x 40 cm			

Table 2-3. Solution Preparation for Monell Environmental Chamber Studies During the Pilot Phase[1]. Monell staff were provided with specific instructions for weighing out a designated mass of each concentrate to achieve the designated chemical concentrations in the proper volume of deionized water.

Solution	Wetted Cloths Used (cloths/day)	Solution Volume @125 mL/cloth (mL/day)	Volume of Concentrate [2] Used (mL)	Mass of Concentrate [2] Used (g)	Mixing Ratio (oz. concentrate/gal)
A: Acetic Acid Only	12	1500	33.72	33.73	3.0
B: OxyCide™	12	1500	33.72	38.02	3.0
C: Hydrogen Peroxide Only	4	500	11.32	12.51	3.0
D: De-ionized Water Only	4	500	11.24	11.24	3.0

[1]The mass of concentrate was weighed out into a tared 1-gallon plastic container and the residual volume of deionized water (solution volume minus volume of concentrate) was added to the container and mixed well. All solutions were prepared fresh each morning for use in chamber study trials on the same day.

[2]The volume of concentrate used was set at 3 oz/gal, corresponding to the dilution rate designated for the use solution of OxyCide™ on the label and corresponding safety data sheet. The mass corresponding to the volume of concentrate used was estimated based on solution density values from the Ecolab chemist overseeing the production of all the solutions: A (1.000 g/mL); B (1.128 g/mL); C (1.105 g/mL); D (1.000 g/mL).

Table 2-4. Solution Preparation for Monell Environmental Chamber Studies Post-Pilot Phase[1]. Monell staff were provided with specific instructions for weighing out a designated mass of each concentrate to achieve the designated chemical concentrations in the proper volume of deionized water

Solution	Wetted Cloths Used (cloths/day)	Solution Volume @125 mL/cloth (mL/day)	Volume of Concentrate[2] Used (mL)	Mass of Concentrate[2] Used (g)	Mixing Ratio (oz. concentrate/gal)
A: Acetic Acid Only	12	1500	80.94	81.74[3]	7.2
B: OxyCide™	12	1500	33.72	38.02	3.0
C: Hydrogen Peroxide Only	4	500	11.32	12.51	3.0
D: De-ionized Water Only	4	500	11.24	11.24	3.0

[1]The mass of concentrate was weighed out into a tared 1-gallon plastic container and the residual volume of deionized water (Solution volume minus volume of concentrate) was added to the container and mixed well. All solutions were prepared fresh each morning for use in chamber study trials on the same day.

[2]The volume of concentrate used was set at 3 oz/gal, corresponding to the dilution rate designated for the use solution of OxyCide™ on the label and corresponding safety data sheet. The mass corresponding to the volume of concentrate used was estimated based on solution density values from the Ecolab chemist overseeing the production of all the solutions: A (1.000 g/mL); B (1.128 g/mL); C (1.105 g/mL); D (1.000 g/mL).

[3] To accomplish similar airborne AA concentrations to the OxyCide™ solution, the mass of AA-only concentrate was increased by 2.4-fold starting with the third multi-day volunteer on testing week 3.

Table 2-5. Chamber Conditions for Multi-Day Study Subjects[1]

Study Days	Temperature (F; average, minimum, and maximum)	Relative Humidity (%)	Supply Air Flow (ft^3/min)	Exhaust Air Flow (ft^3/min)	Air changes per hour (ACH)[2]
7/21/2021 - 7/25/2021[3]	70.5 (69.1, 77.4)	55.5	58.7	68.8	5.4
7/26/2021 - 7/30/2021	70.3 (69.1, 72.2)	57.3	60.0	69.8	5.5
8/2/2021 - 8/6/2021	70.5 (68.7, 74.1)	57.0	59.5	69.4	5.4
8/9/2021 - 8/13/2021	70.3 (69.1, 71.4)	58.1	59.8	69.6	5.5
8/16/2021 - 8/20/2021[4]	70.4 (69.1, 72.0)	59.6	59.5	69.3	5.4
8/23/2021 - 8/27/2021	70.2 (69.1, 72.2)	56.8	60.2	70.0	5.5
9/20/2021 - 9/24/2021	70.0 (68.7, 71.0)	50.6	59.0	69.0	5.4
9/27/2021 - 10/1/2021	71.7 (68.6, 80)	49.3	59.9	69.7	5.5
Average	70.5	55.5	59.6	69.4	5.4
SD	0.52	3.7	0.51	0.42	0.03

[1] Data were recorded every minute during the 8-hour cleaning sessions and then averaged over the entire 5 days of consecutive cleaning

[2] The ACH is calculated by the following formula: Exhaust air flow (ft^3/min)*60 min/chamber volume (ft^3). The chamber is 11.5 ft x 9.5 ft x 7 ft; total volume is 764.75 ft^3.

[3] Chamber data on this volunteer was only available for 3 days of cleaning

[4] Chamber data on this volunteer was only available for 4 days of cleaning

Table 2-6. Log of Samples Lost or Omitted for Likely Contamination*

Multi-Day Volunteer Subset:				
Sample date code	Solution	Volunteer Week	Analyte	Comments
0727B1	B	2	AA	Tube lost or broken at Monell
0809B1	B	4	ALL	Sample train fell off vest; contaminated
0809B2	B	4	ALL	Sample train fell off vest; contaminated
0809B3	B	4	ALL	Sample train fell off vest; contaminated
0819	A,B,C,D	5	ALL	Volunteer absent for Thursday of test week
0719A1 to 0730A3	A	1, 2	ALL	Pilot phase low AA; adjusted by 2.4x forward
0806A1	A	3	AA	Tube lost or broken at Monell
0810!3	A	3	AA	Tube lost or broken at Monell
0921A2	A	7	AA	Tube lost or broken at Monell
0930A3	A	8	AA	Tube lost or broken at Monell
Single Day Volunteer Subset				
1117A2	A,B,C,D		ALL	Pump shut off time failure
0627C1	C		AA	High outlier for AA concentration
* Several samples, especially for PAA in groups C and D, were below the reporting limit and were not considered exceptions (lost or excluded).				

Table 2-7. Chamber Conditions for Single-Day Study Subjects[1]

Study Day	Temperature (F; average, minimum, and maximum)	Relative Humidity (%)	Supply Air Flow (ft^3/min)	Exhaust Air Flow (ft^3/min)	Air changes per hour (ACH)[2]
7/21/2021	71.0 (70, 77.4)	54.9	58.4	68.4	5.4
7/26/2021	70.2 (69.3, 71.4)	57.7	59.9	69.8	5.5
8/2/2021	70.3 (68.8, 71.4)	57.2	59.1	69.0	5.4
8/9/2021	70.4 (69.5, 71.4)	57.9	59.5	69.2	5.4
8/16/2021	70.3 (69.1, 71.4)	59.5	59.0	68.8	5.4
8/23/2021	70.4 (69.5, 71.7)	57.3	59.6	69.4	5.4
9/20/2021	70.0 (68.4, 70.8)	57.1	58.4	68.4	5.4
9/27/2021	77.1 (72.9, 80)	39.3	60.3	70.0	5.5
10/4/2021	70.4 (69.2, 71.4)	60.6	59.2	69.0	5.4
10/21/2021	69.9 (68.2, 70.9)	48.4	59.7	69.9	5.5
10/25/2021	70.0 (69.9, 71.1)	52.8	59.7	70.0	5.5
10/28/2021	69.7 (67.8, 71.5)	42.4	59.8	69.7	5.5
11/11/2021	69.8 (67.6, 72.4)	34.4	60.3	70.3	5.5
11/17/2021	69.6 (67.6, 70.8)	33.2	60.5	70.4	5.5
11/19/2021	75.8 (72.0, 77.0)	20.3	60.1	69.6	5.5
11/22/2021	77.9 (74.5, 78.8)	28.1	60.1	70.1	5.5
12/1/2021	77.0 (73.9, 77.9)	23.5	60.3	70.3	5.5
12/6/2021	75.3 (74.2, 76.7)	40.1	59.9	70.0	5.5
12/16/2021	75.5 (73.9, 77.1)	34.9	59.3	69.4	5.4
12/20/2021	77.3 (74.4, 78.1)	16.1	59.8	69.7	5.5
1/25/2022	73.6 (71.4, 75.6)	20.7	59.5	69.8	5.5
2/2/2022	70.6 (69.6, 72.6)	27.1	60.2	69.9	5.5
3/23/2022	68.9 (66.3, 70.9)	27.8	59.7	70.1	5.5
3/28/2022	70.0 (69.0, 71.0)	15.1	60.2	70.2	5.5
3/30/2022	70.1 (68.6, 72.1)	14.6	59.7	70.1	5.5
4/4/2022	70.2 (68.3, 72.0)	26.5	60.3	70.1	5.5
4/6/2022	71.7 (70.4, 72.5)	43.5	60.2	69.7	5.5
4/8/2022	69.8 (68.5, 71.0)	30.1	59.6	70.2	5.5
4/13/2022	73.9 (69.5, 78.1)	47.4	59.7	69.8	5.5
5/3/2022	70.1 (67.9, 73.0)	46.5	60.0	70.1	5.5
5/6/2022	69.9 (68.2, 71.7)	48.3	57.9	68.2	5.4
5/9/2022	69.9 (67.1, 71.8)	22.0	60.1	70.5	5.5
5/13/2022	69.9 (68.4, 71.5)	51.1	60.1	70.0	5.5
5/18/2022	69.8 (67.4, 71.0)	35.9	60.1	70.2	5.5
5/23/2022	70.0 (68.6, 71.5)	42.9	60.1	70.0	5.5
6/6/2022	69.8 (67.6, 71.5)	39.1	59.5	69.8	5.5
6/10/2022	69.8 (67.9, 71.8)	60.2	60.2	69.9	5.5

6/20/2022	69.5 (66.9, 71.2)	32.3	61.6	71.9	5.6
6/27/2022	69.9 (68.1, 71.9)	62.2	59.7	69.9	5.5
Average (all)	71.4	40.2	59.8	69.8	5.5
SD (all)	2.6	14.6	0.64	0.66	0.052
Average (single-day)	71.5	36.4	59.9	70.0	5.5
SD (single-day)	2.7	13.6	0.57	0.57	0.050
[1] Data were recorded every minute during the 8-hour cleaning sessions and then averaged over the entire day for single-day volunteers. The first 8 rows are the chamber data for the first study day for the multi-day volunteers					
[2] The ACH is calculated by the following: Exhaust air flow (ft^3/min)*60 min/chamber volume (ft^3). The chamber is 11.5 ft x 9.5 ft x 7 ft; total volume is 764.75 ft^3.					

Table 2-8. Summary Statistics for Monell Environmental Chamber Studies of OxyCide™ and Its Components in Single-Day Female Volunteers*

	Peracetic Acid (µg/m³; ppb)[a]				Hydrogen Peroxide (µg/m³; ppb)[b]				Acetic Acid (µg/m³; ppb)[c]			
Chamber Test Solution	n	Mean	S.D.	S.E.M.	n	Mean	S.D.	S.E.M.	n	Mean	S.D.	S.E.M.
OxyCide™	114	208.6 (67.0	76.4 (24.5)	7.1 (2.3)	114	454.4 (326.7)	168.0 (120.8)	15.5 (11.2)	114	1,011.7 (412)	370.7 (151)	34.3 (14.0)
Acetic Acid Only	112	13.7 (4.4)	9.5 (3.1)	0.9 (0.3)	106	127.8 (91.9)	82.6 (59.4)	7.9 (5.7)	106	944.7 (384)	486.9 (198)	46.4 (19.0)
Hydrogen Peroxide Only	38	14.0 (4.5)	13.2 (4.2)	2.1 (0.7)	38	391.8 (281.7)	177.5 (127.6)	28.4 (20.4)	38	169.1 (69.0)	116.5 (47.0)	18.7 (8.0)
Deionized Water Only	40	14.1 (4.5)	13.9 (4.5)	2.2 (0.7)	40	23.5 (16.9)	21.4 (15.4)	3.4 (2.4)	40	175.2 (71.0)	133.7 (54.0)	21.1 (9.0)

* Summary of data for 32 single-day volunteers combined with the first sampling day of 8 multi-day volunteers; thus, a total of 40 days of testing is reflected in the data presented here.

[a] The average peracetic acid concentration was significantly different between OxyCide™ and the other chamber test solutions ($p<0.001$). The other pairwise comparisons were not statistically significant.

[b] All pairwise comparisons between solutions were statistically significantly different ($p<0.001$) with the exception of OxyCide™ and hydrogen peroxide only ($p=0.0514$).

[c] The average concentration for the OxyCide™ and acetic acid only groups was significantly higher than both hydrogen peroxide and deionized water ($p<0.001$). No significant difference was found between hydrogen peroxide only and deionized water only ($p = 0.9999$) or OxyCide™ and acetic acid ($p = 0.5398$).

Table 2-9. Summary Statistics for Monell Environmental Chamber Studies of OxyCide™ and Its Components in Single-Day Male Volunteers*

	Peracetic Acid (µg/m³; ppb)[a]				Hydrogen Peroxide (µg/m³; ppb)[b]				Acetic Acid (µg/m³; ppb)[c]			
Chamber Test Solution	n	Mean	S.D.	S.E.M.	n	Mean	S.D.	S.E.M.	n	Mean	S.D.	S.E.M.
OxyCide™	12	146.7 (47.1)	35.9 (11.5)	10.4 (3.3)	12	369.8 (266)	93.5 (67.3)	27.0 (19.4)	12	1,055.4 (429)	407.3 (166)	117.6 (48.0)
Acetic Acid Only	12	15.3 (4.9)	10.4 (3.3)	3.0 (1.0)	12	133.4 (95.9)	51.1 (36.7)	14.7 (10.6)	12	1,085.2 (441)	249.0 (101)	71.9 (29.0)
Hydrogen Peroxide Only	4	20.3 (6.5)	18.6 (6.0)	9.3 (3.0)	4	320.8 (230.7)	42.9 (30.9)	21.5 (15.4)	4	460.8 (187)	450.2 (183)	225.1 (92.0)
Deionized Water Only	4	9.3 (3.0)	6.6 (2.1)	3.3 (1.1)	4	8.9 (6.4)	7.8 (5.6)	3.9 (2.8)	4	304.6 (124)	143.8 (58.0)	71.9 (29.0)

* Summary of data for the 4 single-day male volunteers.

[a] The average peracetic acid concentration was significantly different between OxyCide™ and the other chamber test solutions ($p<0.001$). The other pairwise comparisons were not statistically significant.

[b] All pairwise comparisons between solutions were statistically significantly different ($p<0.001$) with the exception of OxyCide™ and hydrogen peroxide only ($p=0.6055$).

[c] The average concentration for the OxyCide™ and acetic acid only groups was significantly higher than both hydrogen peroxide and deionized water ($p<0.05$). No significant difference was found between hydrogen peroxide only and deionized water only ($p = 0.9127$) or OxyCide™ and acetic acid ($p = 0.9963$).

Table 2-10. Correlation Analysis for Selected Parameters in the Mass Transfer Studies of OxyCide™ Use at 1, 2, and 4 Wetted Cloths per 20-min Trial

Parameter X	Parameter Y	Fit Type	Slope	Intercept	R^2 value
# of Cloths Used	Cleaning Rate (m^2/min)	Linear	0.2x	1.4	1
# of Cloths Used	Soln. Mass Loss from Cloths (g)	Logarithmic	82.16 ln(x)	82.78	0.9999
		Linear	36.53x	54.5	0.9598
# of Cloths Used	Soln. Mass Loss per Time (g/min)	Logarithmic	4.10 ln(x)	4.16	0.9995
		Linear	1.82x	2.75	0.9555
# of Cloths Used	Soln. Mass per Area Wiped (g/m^2)	Logarithmic	1.44 ln(x)	2.67	0.9636
		Linear	0.616x	2.23	0.861
# of Cloths Used	Airborne PAA (mg/m^3)	Exponential	$263e^{0.0432}$		0.9727
		Linear	12.79x	261.5	0.9673
Soln. Mass Loss from Cloths (g)	Soln. Mass Loss per Time (g/min)	Linear	0.05x	0.028	0.9999
Soln. Mass Loss from Cloths (g)	Soln. Mass per Area Wiped (g/m^2)	Logarithmic	2.31 ln(X)	-7.61	0.9964
		Linear	0.018x	1.22	0.9678
Soln. Mass Loss from Cloths (g)	Airborne PAA (mg/m^3)	Exponential	$249.6e^{0.0011x}$		0.8722
		Linear	0.323x	246.2	0.8599
Soln. Mass per Area Wiped (g/m^2)	Airborne PAA (mg/m3)	Exponential	$236.7e^{0.0562x}$		0.7268
		Linear	16.6x	230.6	0.7143
Soln. Mass Loss per Time (g/min)	Airborne PAA (mg/m3)	Exponential	$249.6e^{0.0219x}$		0.8649
		Linear	6.45x	246	0.8524

Figure 2-1. Scatter Graphs of Airborne PAA, HP, and AA Concentrations for Multi-Day Volunteers

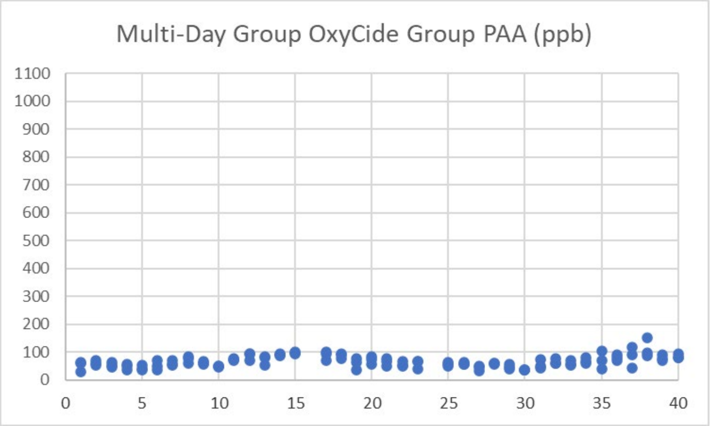

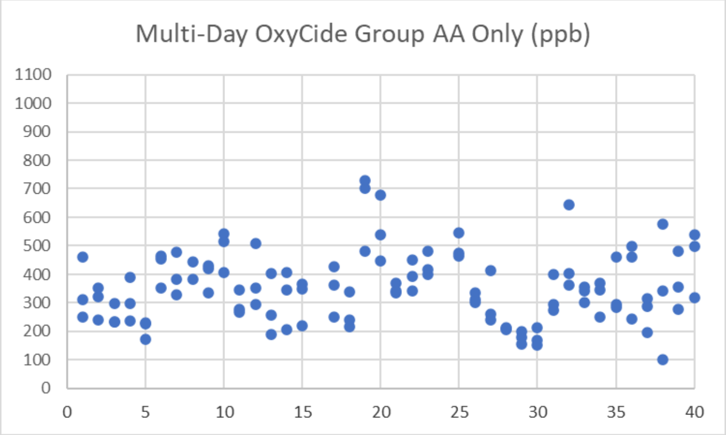

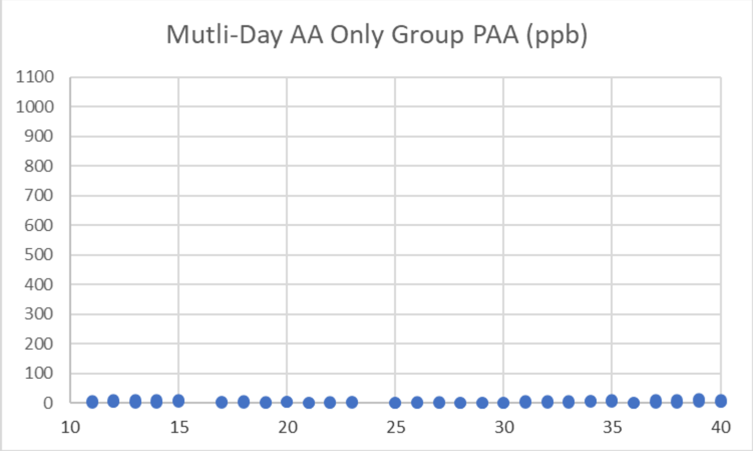

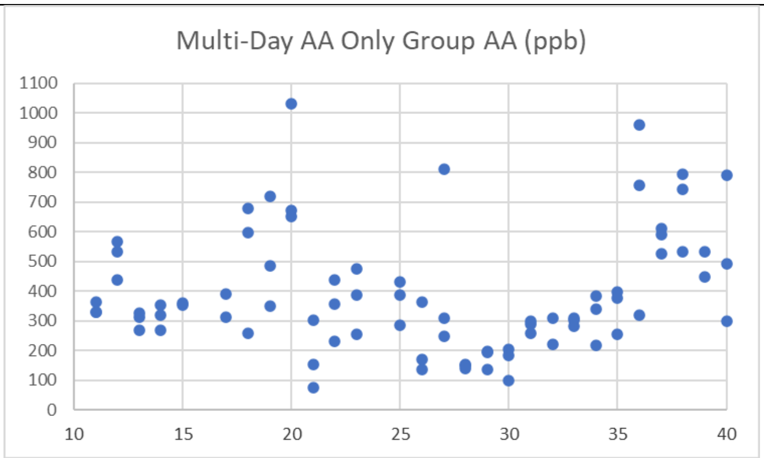

Multi-Day AA Only Group AA (ppb)
1100
1000
900
800
700
600
500
400
300
200
100
0
10
15
20
25
30
35
40

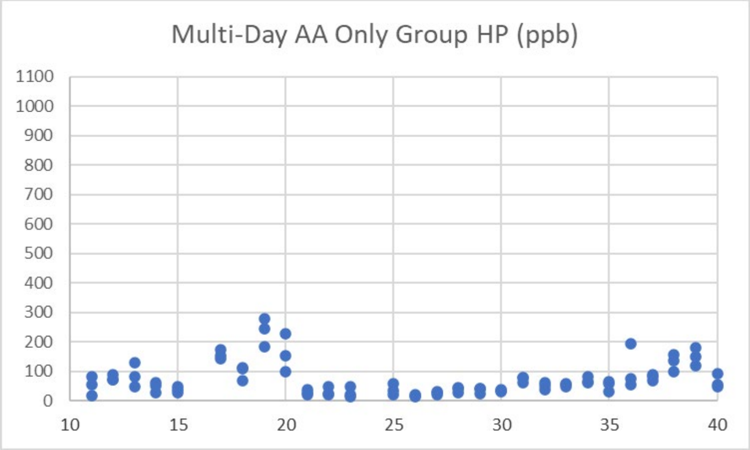

Multi-Day AA Only Group HP (ppb)
1100
1000
900
800
700
600
500
400
300
200
100
0
10
15
20
25
30
35
40

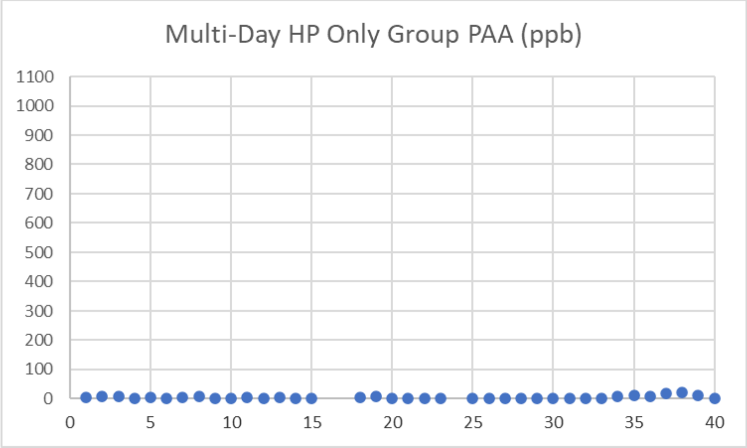

Multi-Day HP Only Group PAA (ppb)
1100
1000
900
800
700
600
500
400
300
200
100
0
0
5
10
15
20
25
30
35
40

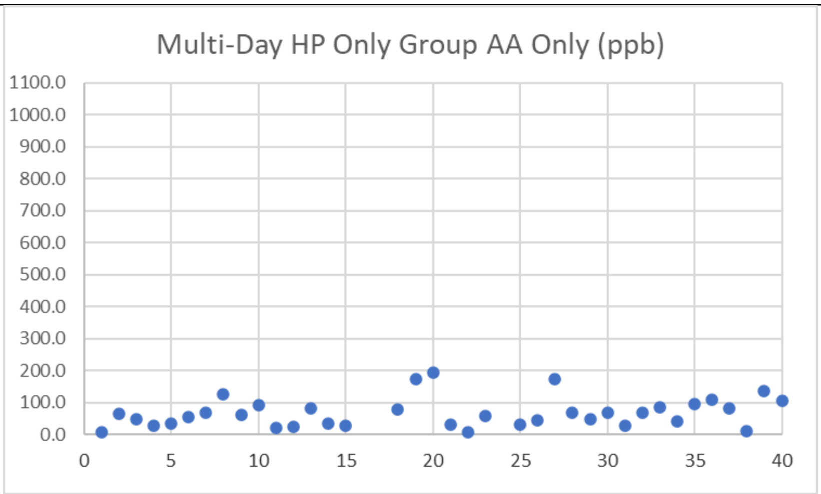
Multi-Day HP Only Group AA Only (ppb)
1100.0
1000.0
900.0
800.0
700.0
600.0
500.0
400.0
300.0
200.0
100.0
0.0
0
5
10
15
20
25
30
35
40

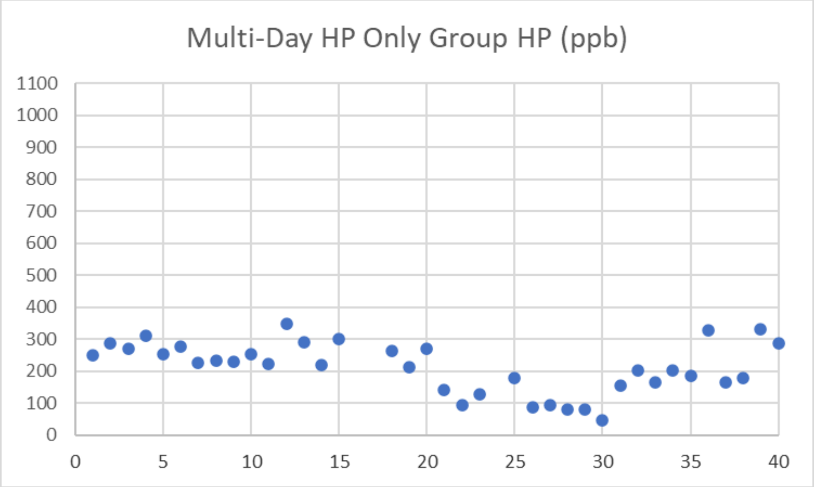
Multi-Day HP Only Group HP (ppb)
1100
1000
900
800
700
600
500
400
300
200
100
0
0
5
10
15
20
25
30
35
40

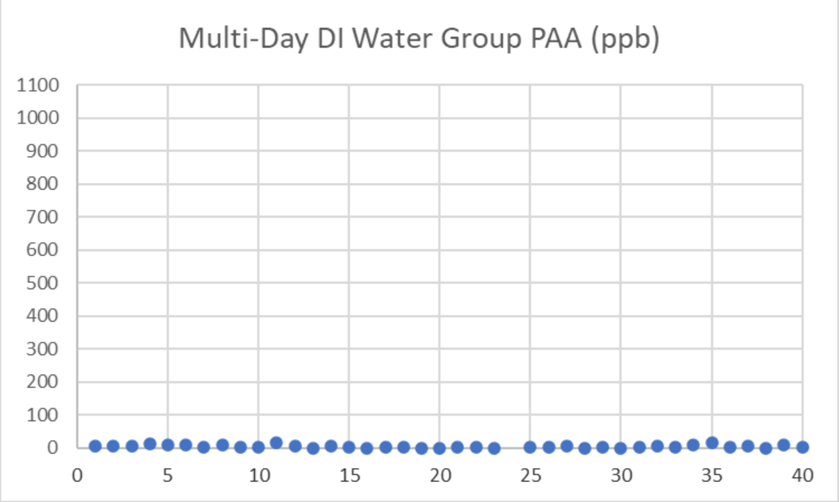
Multi-Day DI Water Group PAA (ppb)
1100
1000
900
800
700
600
500
400
300
200
100
0
0
5
10
15
20
25
30
35
40

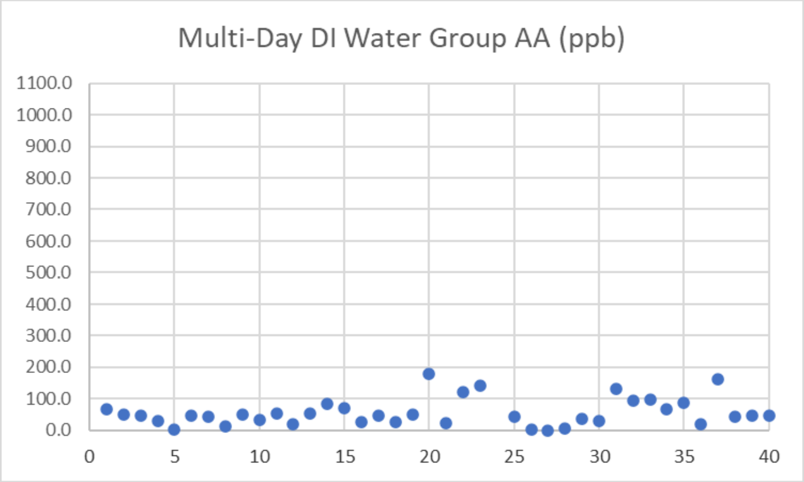
Multi-Day DI Water Group AA (ppb)
1100.0
1000.0
900.0
800.0
700.0
600.0
500.0
400.0
300.0
200.0
100.0
0.0
0
5
10
15
20
25
30
35
40

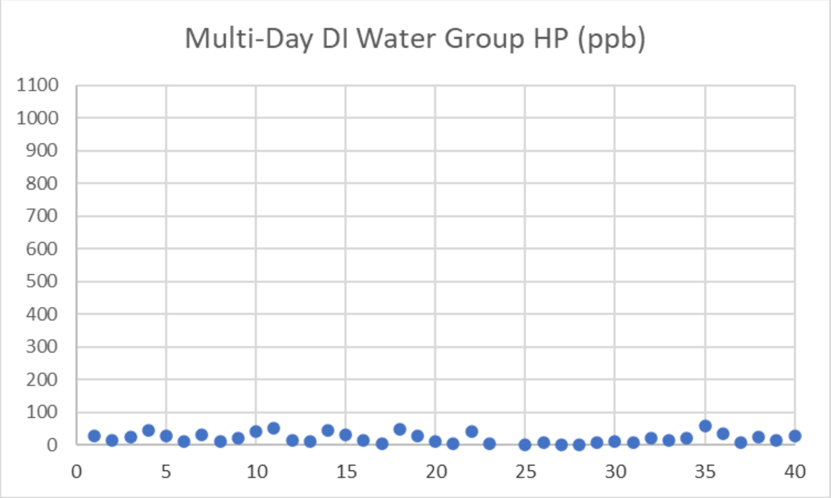
Multi-Day DI Water Group HP (ppb)
1100
1000
900
800
700
600
500
400
300
200
100
0
0
5
10
15
20
25
30
35
40

Figure 2-2. Scatter Graphs of Airborne PAA, HP, and AA Concentrations for Single-Day Volunteers

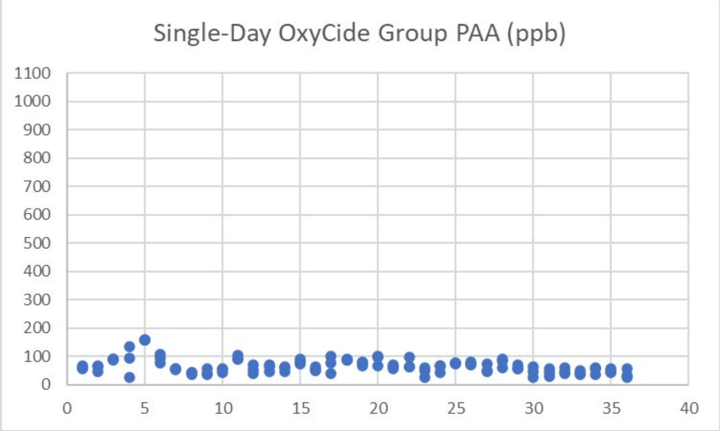

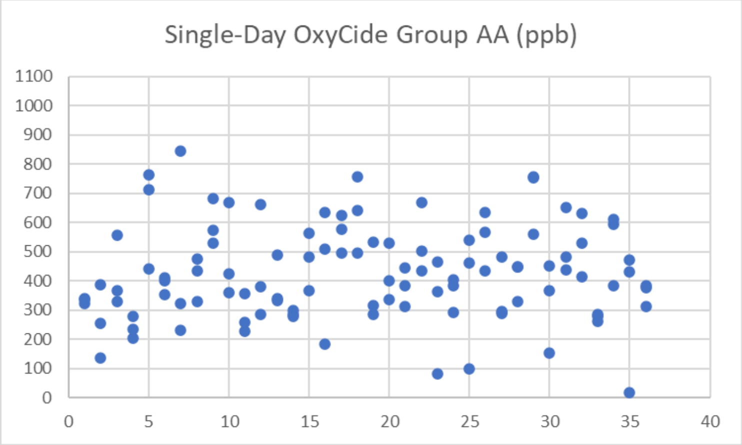

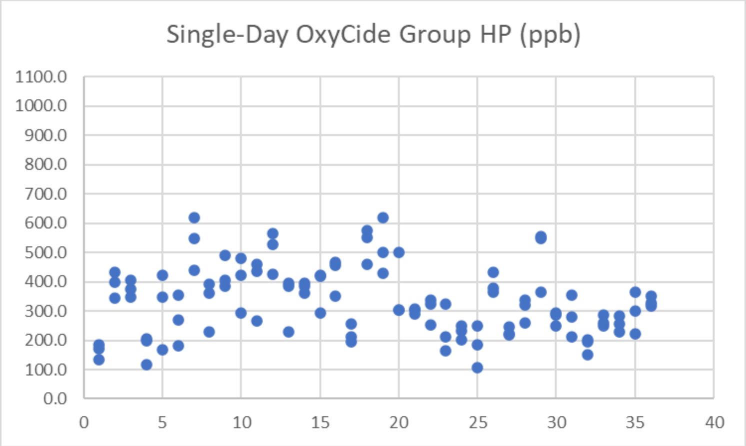

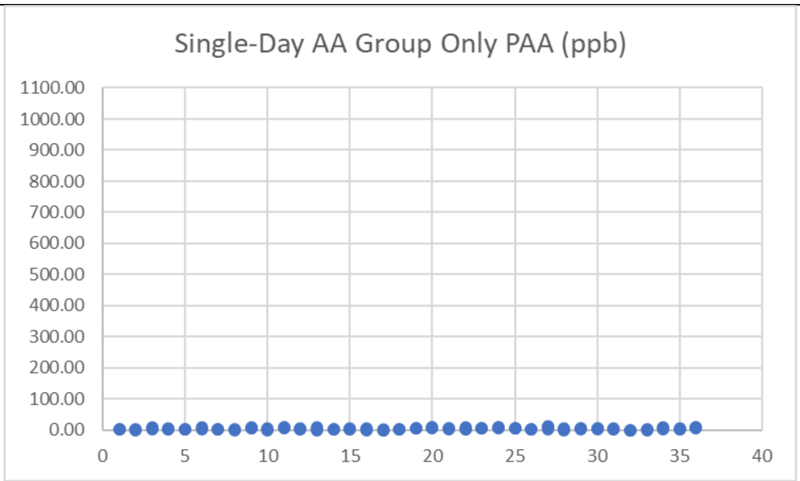
Single-Day AA Group Only PAA (ppb)
1100.00
1000.00
900.00
800.00
700.00
600.00
500.00
400.00
300.00
200.00
100.00
0.00
0
5
10
15
20
25
30
35
40

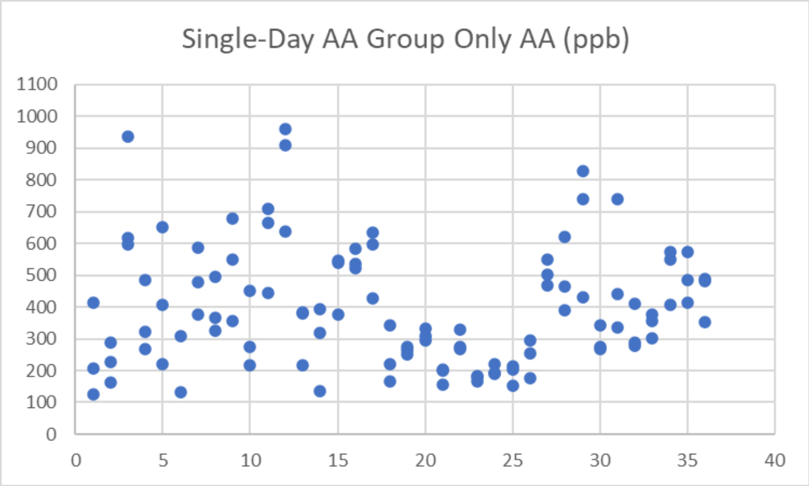
Single-Day AA Group Only AA (ppb)
1100
1000
900
800
700
600
500
400
300
200
100
0
0
5
10
15
20
25
30
35
40

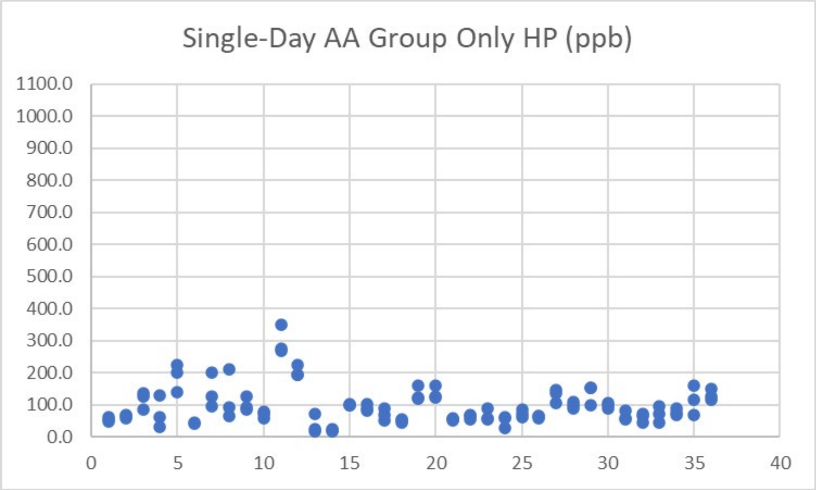
Single-Day AA Group Only HP (ppb)
1100.0
1000.0
900.0
800.0
700.0
600.0
500.0
400.0
300.0
200.0
100.0
0.0
0
5
10
15
20
25
30
35
40

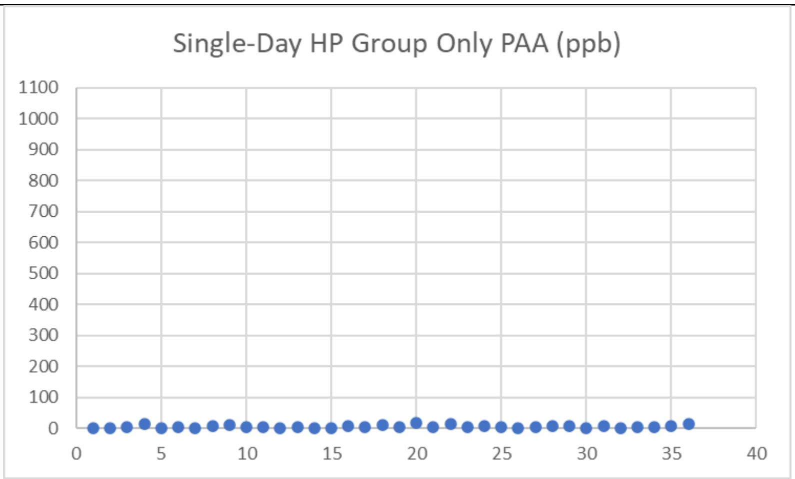
Single-Day HP Group Only PAA (ppb)
1100
1000
900
800
700
600
500
400
300
200
100
0
0
5
10
15
20
25
30
35
40

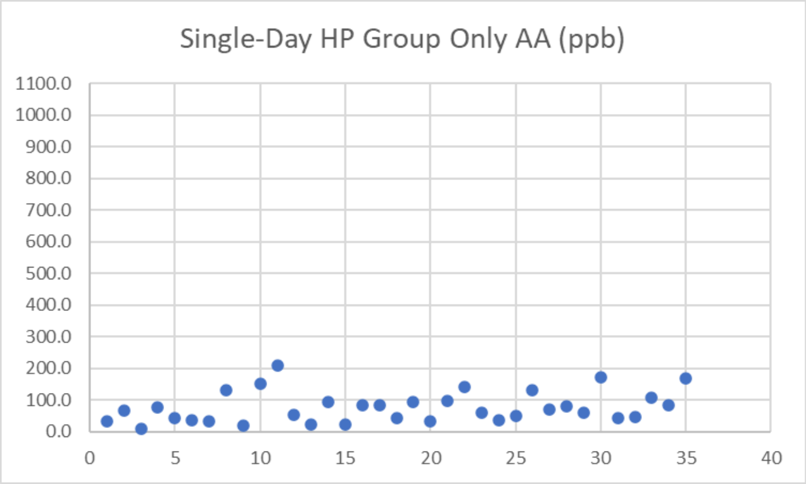
Single-Day HP Group Only AA (ppb)
1100.0
1000.0
900.0
800.0
700.0
600.0
500.0
400.0
300.0
200.0
100.0
0.0
0
5
10
15
20
25
30
35
40

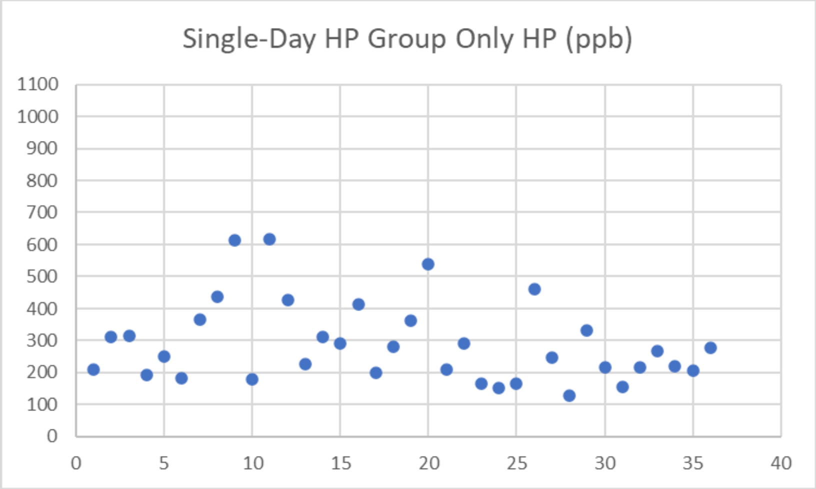
Single-Day HP Group Only HP (ppb)
1100
1000
900
800
700
600
500
400
300
200
100
0
0
5
10
15
20
25
30
35
40

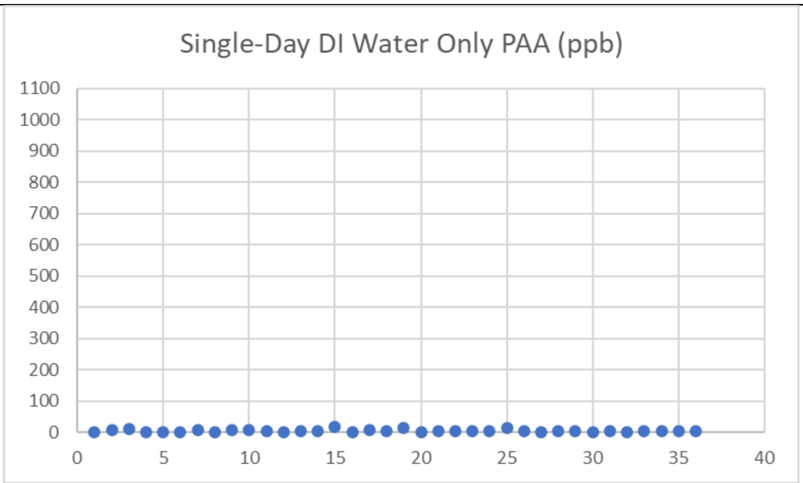
Single-Day DI Water Only PAA (ppb)
1100
1000
900
800
700
600
500
400
300
200
100
0
0
5
10
15
20
25
30
35
40

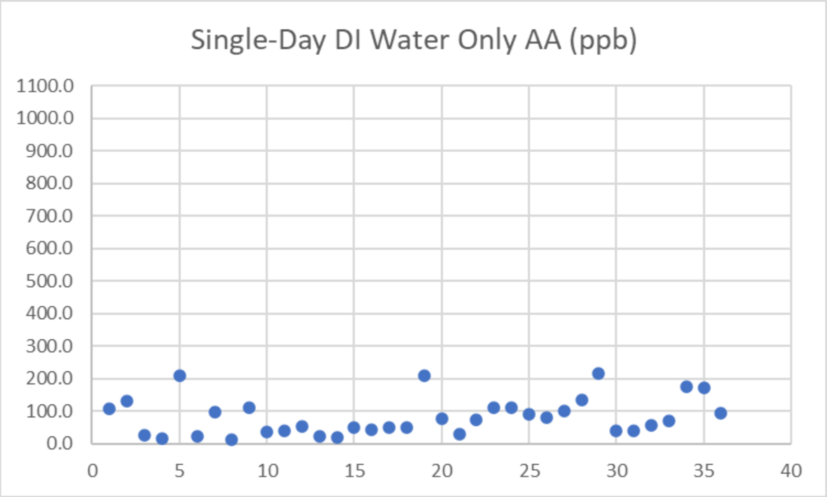
Single-Day DI Water Only AA (ppb)
1100.0
1000.0
900.0
800.0
700.0
600.0
500.0
400.0
300.0
200.0
100.0
0.0
0
5
10
15
20
25
30
35
40

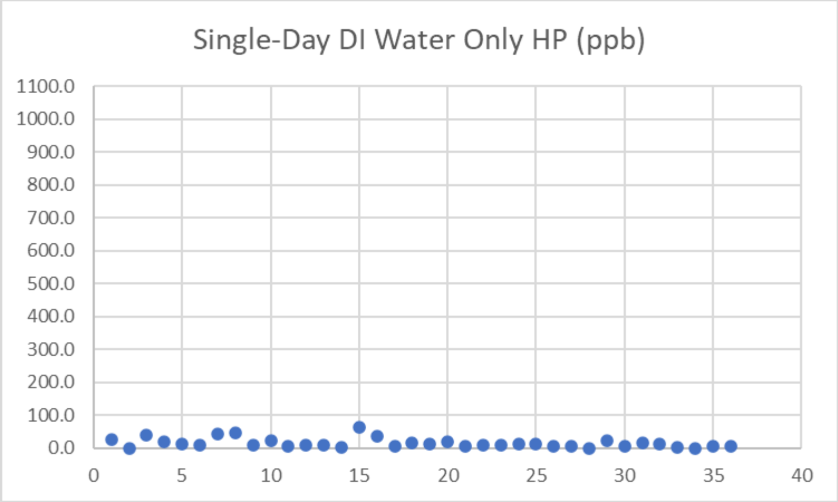
Single-Day DI Water Only HP (ppb)
1100.0
1000.0
900.0
800.0
700.0
600.0
500.0
400.0
300.0
200.0
100.0
0.0
0
5
10
15
20
25
30
35
40

3. Hospital Dispenser Calibration Study

Calibration Data on OxyCide™ Dispensers in Hospital Facilities

The Ecolab field representatives are tasked with performing dispenser calibration testing at least quarterly to assure that the proper concentration of OxyCide™ is being dispensed, with the EPA label-designated effective sporicidal concentration range of approximately 3.0 to 3.3 ounces of concentrate per gallon of cold tap water. The dispenser is calibrated by adjusting input water pressure that hydraulically draws a fixed stream of concentrate into solution as it passes a nozzle attached to a tube in the concentrate bottle. The dilution rate is determined by measuring the weight difference for a fixed volume of tap water and for the OxyCide™ solution as dispensed; the mass difference is then related to the dilution rate in oz/gal.

An internal Ecolab study was conducted from December 2013 through December 31, 2015 to evaluate dispenser performance at 381 hospital facilities concurrently using OxyCide™. A total of 11,220 dispensers were included in the study, each having at least 2 calibration tests: one taken at dispenser installation and a second taken at approximately 3 months later. Adjustments were recorded at the 3-month check for 1,240 of the dispensers for a total of 23,680 calibration test results.

Initial calibration studies of OxyCide™ dispensers in hospitals (Figure 3-1) indicated that mean dilution was within 10% of the minimum level identified on the USEPA-approved labeling for sporicidal activity (3 oz/gal) and even the 95th percentile values were within 21% of this target dilution.

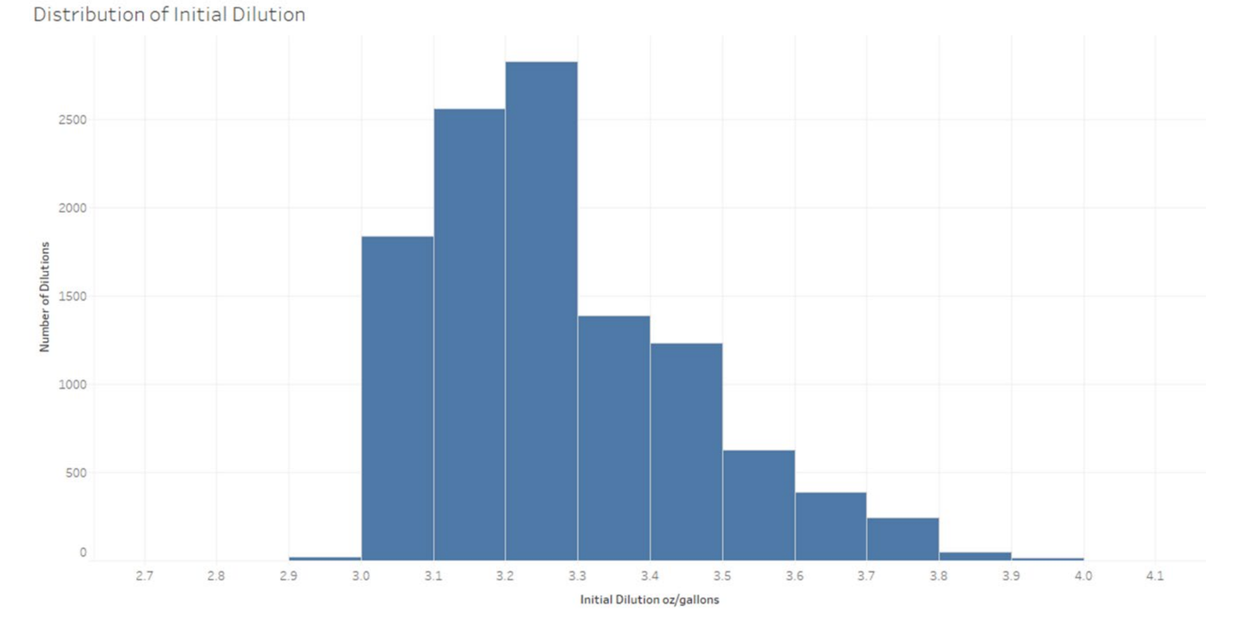

Figure 3-1. Distribution of Initial Dilution Rate Measurements for the Ecolab Dispenser Used for OxyCide™. Technicians measured the mass of two sequential 32 oz bottles from the dispenser and subtracted the mass of the source tap water to determine the mass of

OxyCide™, which was then related to the dilution rate in fluid ounces of concentrate per gallon of tap water.

A total of 11,220 dispensers were counted across 381 facilities; some facilities were noted to have over 100 dispensers present (Figure 3-2).

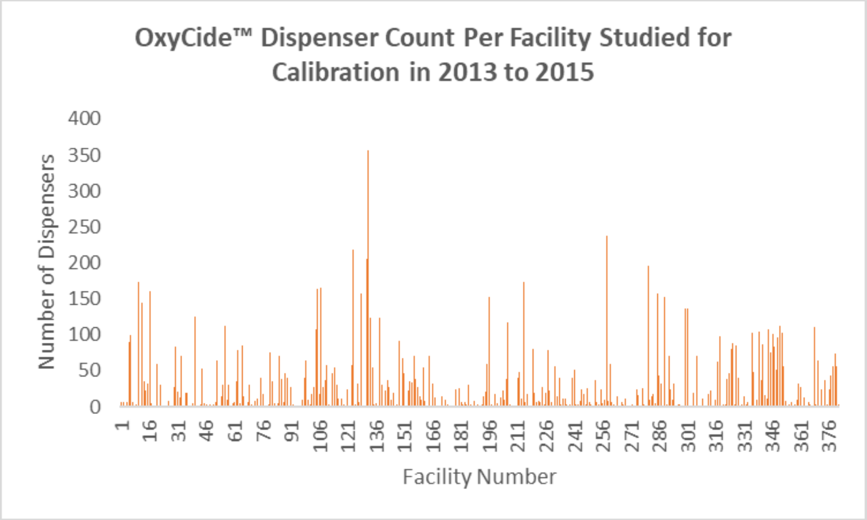

Figure 3-2. Number of dispensers across various hospital facilities. Technicians collected calibration data from 11,220 dispensers across 381 facilities. These measurements were taken from 2013 through 2015; however, most were taken in 2014 and 2015 (10 dispenser readings were taken in December 2013; those were counted in with the readings from 2014). Of the 11,220 readings, 24% were taken in 2014 and 76% were taken in 2015.

The OxyCide™ dilution rate showed a mean value of 3.35 oz/gal at installation and at the initial 3-month check, and dispensers subject to adjustment at the 3-month check showed a slightly lower mean value of 3.20 oz/gal (Figure 3-3).

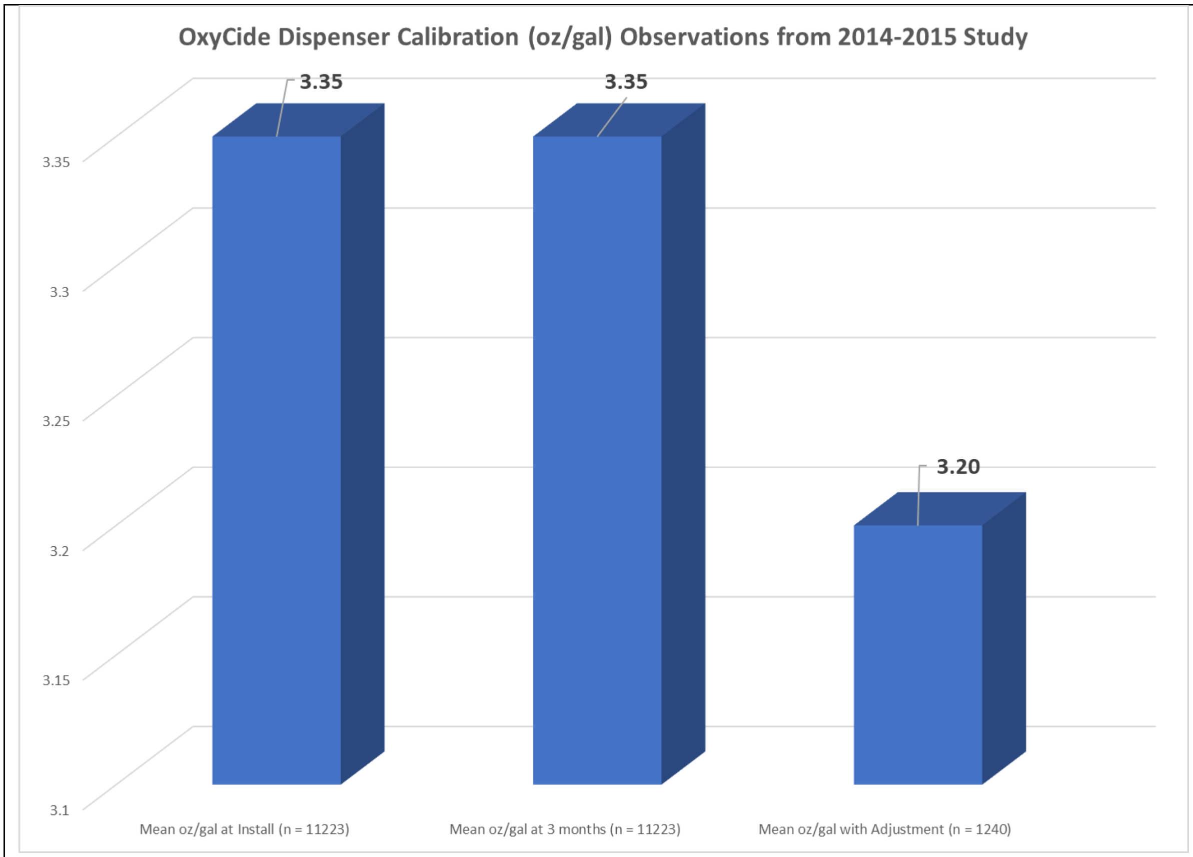

Figure 3-3. OxyCide™ dispenser calibration observations. A total of 11,220 dispensers were included in the study, each having at least 2 calibration tests: one taken at dispenser installation and a second taken at approximately 3 months later. Adjustments were recorded at the 3-month check for 1,240 of the dispensers for a total of 23,680 calibration test results. The OxyCide™ dilution rate showed a mean value of 3.35 oz/gal at installation and at the initial 3-month check, and dispensers subject to adjustment at the 3-month check showed a slightly lower mean value of 3.20 oz/gal.

The variability in dispenser calibration was assessed by difference in calibration (i.e., subtracting the measurement at installation from the measurement taken at the 3-month check) which showed that 98.8% of the values were within approximately 10% of each other (i.e., less than 0.3 oz/gal difference). The frequency of readings that increased more than 0.3 oz/gal between installation and the 3-month check was 0.74% (0.45% between 0.3 oz/gal, and 0.5 and 0.29% > 0.5 oz/gal). The frequency of readings that decreased more than 0.3 oz/gal between installation and the 3-month check was 0.42% (0.37% between 0.3 and 0.5 oz/gal, and 0.07% > 0.5 oz/gal; Figure 3-4). Summary statistics and figures illustrating the dispenser calibration study results are provided below.

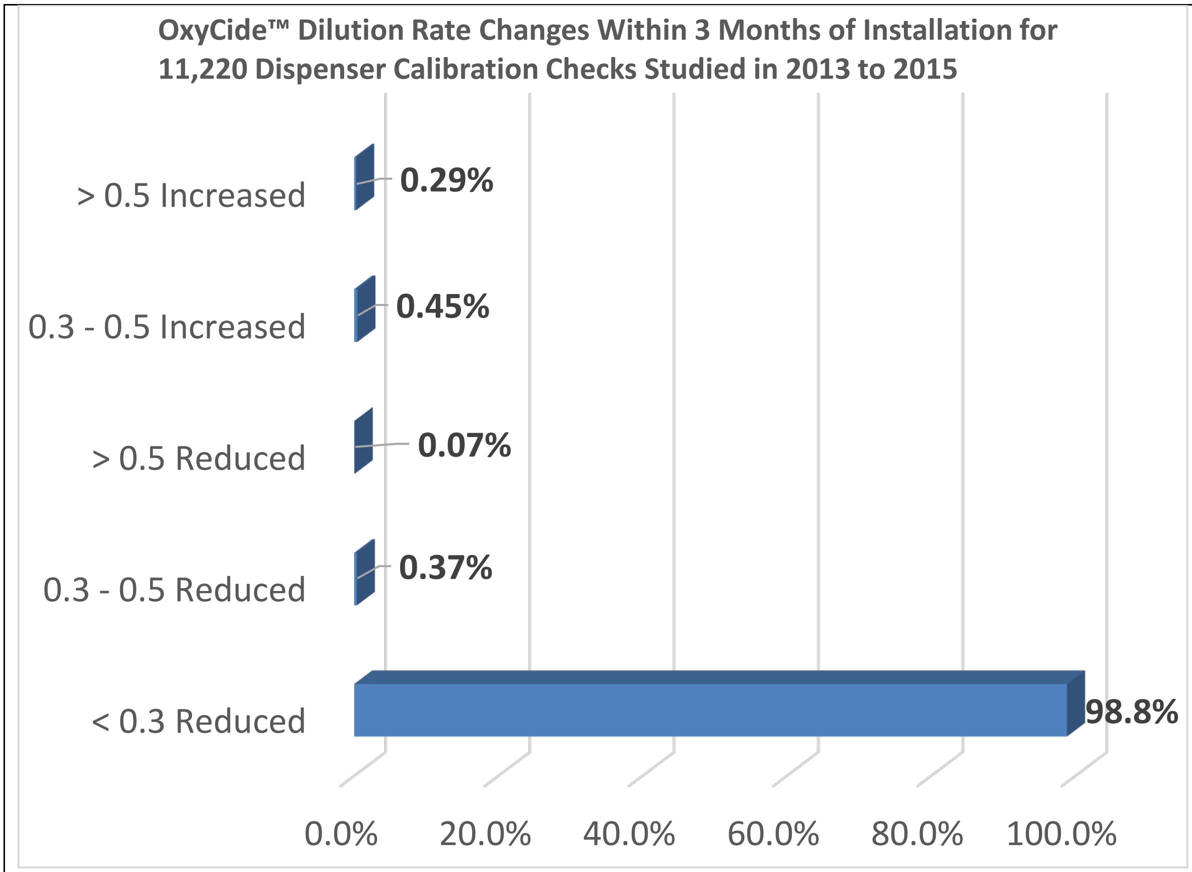

Figure 3-4. OxyCide™ Dilution Rate Changes Within 3 Months of Installation. The variability in dispenser calibration was assessed by difference in calibration (i.e., subtracting the measurement at installation from the measurement taken at the 3-month check) which showed that 98.8% of the values were within approximately 10% of each other (i.e., less than 0.3 oz/gal difference). The frequency of readings that increased more than 0.3 oz/gal between installation and the 3-month check was 0.74% (0.45% between 0.3 oz/gal, and 0.5 and 0.29% > 0.5 oz/gal). The frequency of readings that decreased more than 0.3 oz/gal between installation and the 3-month check was 0.42% (0.37% between 0.3 and 0.5 oz/gal, and 0.07% > 0.5 oz/gal).

4. Hospital Time-Activity Studies

Hospital Time-Activity Studies with Use of the OxyCide™Product

A two-phase study was conducted to obtain quantitative data on time-activity patterns for trained environmental services staff performing patient room/bathroom discharge cleaning. The Phase 1 study conducted in 2021 included observations on 9 environmental services staff who worked at one of four hospitals that participated. Ecolab field representatives were provided with a protocol for recording the timing of activities during room cleaning with particular focus on characterizing the duration and items cleaned when handling OxyCide™ wetted cloths. Each of the shadowed environmental service staff members was requested to perform their normal cleaning activities and the wetted cloth handling observations were recorded on a log that was submitted to the authors (BK and AL) for analysis. Phase 2 study conducted in 2022 included observations on 40 additional environmental service staff who worked at 11 hospitals other than those studied in Phase 1. The same shadowing technique was applied in Phase 2 using a smartphone application to record the start and stop time for room cleaning and OxyCide™ cloth handling. The primary difference between Phase 1 and Phase 2 of the study was that no start and stop times for each cloth was recorded in Phase 2. Also, Phase 2 included standard patient rooms (N=30), isolation rooms (N=5), and some rooms that did not have a bathroom (N=5).

The data from these studies is outlined in this Appendix.

To evaluate the patterns of OxyCide™ use for patient room discharge cleaning, the authors developed a protocol for an observer to report specifically on the time that trained EVS workers in hospitals spend handling wetted cloths. The steps outlined in the protocol were as follows:

1. In each hospital, different EVS workers were observed performing their standard discharge cleaning procedure using OxyCide™ in a patient room. EVS workers who regularly cleaned patient rooms were selected to participate. EVS workers were told that the observer would simply be writing down the timing and location of their tasks while they followed their normal procedure for room cleaning and they were informed that this was not a test of efficiency or cleaning techniques, but that this study was designed to collect data on the normal timing of OxyCide™ wetted cloth handling during the standard procedures the EVS worker has been trained to follow for discharge cleaning of the single patient room.

2. The observer collected one log (see the end of this appendix for the time-activity log form) for each of the selected EVS workers during discharge cleaning of the selected patient room and bathroom.

3. The observer documented the total elapsed time in the room from start to finish (from the time the worker walked into the room until they walked out).

4. For tasks NOT involving OxyCide™ wetted cloth use: the observer documented elapsed time from the start of a task in minutes/seconds to the nearest quarter minute of time (e.g. pulling trash, restocking supplies, making the bed).

5. For tasks that did involve OxyCide™ wetted cloth use: the observer documented the exact amount of time, what the worker cleaned, and how they disposed of each individual wetted OxyCide™ cloth used.
   - For tasks involving OxyCide™ wetted cloth use for items that took > 1 minute to clean (e.g. large items like bed, chair, shower stall), the observer was told to record the exact start and finish time to the point where the worker discarded the cloth or moved to the next task.
   - For tasks involving OxyCide™ wetted cloth use that took < 1 minute to clean, the observer was told to combine cleaning time for all objects until the worker discarded the cloth.
   - If the worker was NOT mopping floors/walls with OxyCide™, the observer was told to time to nearest 15 seconds.
   - If the worker was mopping floors/walls with OxyCide™, the observer was told to document exact time of mopping[1].
   - The observer was told to record the time when each wetted cloth was picked up and when it was considered spent and identify the exact location where each spent cloth was stored.

6. The observer was told to document how the shower was cleaned (OxyCide™ or list other product if known, wiped vs sprayed, rinsed).

7. The observer was told to measure the dimensions (length, width, height) of the shower stall and to do this regardless of whether the stall was cleaned as part of the discharge cleaning process.

8. The observer was asked to provide notes on any hospital-specific procedures that might impact the nature and extent of OxyCide™ exposures to EVS workers during patient room discharge cleaning (e.g., if they didn't have the OxyCide™ bucket covered, or the cloths they were using were oversaturated)

The full study protocol can be found at the end of this Appendix. Table 4-1 contains the summary data from the hospital time-activity studies; Tables 4-2 through 4-10 contain the raw data from each of the 9 EVS workers observed across the 4 hospitals. Table 4-11 contains the raw data from the Phase 2 portion of the study.

[1] Although Ecolab does not recommend mopping use with OxyCide™, a small percentage of hospitals may choose to use the product for mopping. Thus, in this study, if a worker was mopping floors/walls, the observer was asked to document the time of mopping.

Table 4-1. Summary of OxyCide^{TM} Cloth Use Patterns for EVS Workers in Phase 1 and Phase 2 Hospital Studies

Study Phase, Room Type & Sample Size	Total Cloths Used	Time Using Cloths in Patient Room (min)	Time Using Cloths in Bathroom (min)	Total Time Using Cloths (min)	Average Time per Cloth (min)	Total Cleaning Time (min)	Percent of Cleaning Time Using Cloths in Patient Room	Percent of Cleaning Time Using Cloths in Bathroom	Percent of Cleaning Time Using Cloths
Phase 1: Median for Standard Room Clean (n = 9)	5	12	4.1	16.0	4.1	35.4	34.0%	10.7%	46.7%
IQR (25th - 75th percentile)	3 - 7	11.4-12.3	3.2-5.1	13.9-18.0	3.0-4.3	35.2-41.3	31.1%-40.3%	8.6%-15.5%	44.8%-48.9%
Phase 2: Median Standard Room Clean (n = 30)	6	9.2	2.0	10.9	2.9	30.8	33.0%	9.8%	42.1%
IQR (25th - 75th percentile)	3 - 7	7.3-14.1	1.2-5.7	8.8-19.7	1.5-3.8	18.7-42.1	24.4%-53.0%	4.7%-18.9%	30.0%-73.1%
Phase 2: Median for Isolation Room Clean (n = 5)	4	16.6	1.3	17.5	2.1	45.7	23.2%	7.3%	30.1%
IQR (25th - 75th percentile)	3 - 6	7.1-16.8	1.0-2.1	8.4-22.9	1.8-7.6	26.1-94.3	21.9%-36.3%	2.1%-7.9%	24.2%-38.3%
Phase 2: Median for Specialty Room Clean (n = 5)	7	7.2		7.8	1.8	24.9	33.7%		34.9%
IQR (25th - 75th percentile)	4 - 7	4.1-13.0		4.1-14.0	1.0-2.0	8.5-45.2	30.4%-47.2%		32.2%-47.5%
Combined Data (n = 49 or 45)	6	11.3	2.3	12.5	2.8	35.2	33.9%	9.1%	42.1%
IQR (25th - 75th percentile)	3 - 7	7.3-14.2	1.2-5.4	8.8-18.0	1.7-4.1	25.4-45.7	25.3%-43.3%	4.7%-15.5%	31.7%-60.7%

Using Wilcoxon rank sum tests to compare Phase I and 2 for the Standard Room Clean found no significant differences in the medians between phases across each of the outcomes provided in the table ($p>0.05$). Differences were marginally significant at the 10% significance level for Time Using Cloths in Bathroom ($p=0.0923$), Time Using Clothes in Patient Room ($p=0.0596$), and Total Time Using Cloths ($p=0.0553$).

Furthermore, comparing each Phase and Room Clean type (Phase 1 Standard, Phase 2 Standard, Phase 2 Isolation, Phase 2 Specialty) found no significant differences in the medians between groups across each outcome reported ($p>0.05$) except for the bathroom. There was no significant difference in medians between Phase I Standard Room Clean, Phase 2 Standard Room Clean, and Phase 2 Isolation Room Clean for Time Using Cloths in Bathroom or Percent of Cleaning Time Using Cloths in Bathroom ($p>0.05$).

In a second analytical approach, a multivariate regression analysis was performed with Phase/Room Clean type as a factor. No significant difference in average outcome was found except for Total Cleaning Time where Phase 2 Isolation Room Clean was significantly higher than Phase 2 Standard Room Clean ($p<0.05$).

Table 4-2. EVS Worker 1

Hospital #	EVS Worker #	Approximate room and bathroom area cleaned (sq. ft) and location	Total time spent cleaning (min:ss)	Total # OxyCide™ cloths used
1	1	S776/ICU	33:04:00	5
**Cloth #**	**Time grabbed**	**Time of discard**	**Total time used**	**Objects cleaned and observations**
1	4:43	8:01	3:18	Counter<br>hand hygiene dispenser<br>overbed table (5:15-5:38)<br>cabinet handles and fronts<br>Chair (6:59-7:40)<br>phone<br>light switches<br><br>This worker discarded used cloths outside of the room
2	8:00	14:46	6:46	Electrical cords/monitor cords<br>call button<br>blood pressure cuff<br>hand hygiene dispenser<br>nurses call button<br>keyboard/mouse, computer shelf. This worker also replaced trash bags as she went around cleaning
3	14:48	17:02	2:14	bed controls<br>pillows (three)<br>mattress top<br><br>Did not clean underside of mattress or bed frame
4	17:33	20:55	3:22	NA
5	21:10	22:55	1:45	sink bowl<br>faucets<br>toilet<br><br>During this time, she cleaned the toilet bowl for 10 s with non-OxyCide™ cleaner
		total time using cloths	17:25	
		% time using cloths	53	

Table 4-3. EVS Worker 2

Hospital #	EVS Worker #	Approximate room and bathroom area cleaned (sq. ft) and location	Total time spent cleaning (min:ss)	Total # OxyCide™ cloths used
1	2	NA	41:15:00	3
**Cloth #**	**Time grabbed**	**Time of discard**	**Total time used**	**Objects cleaned and observations**
1	7:17	10:31	3:14	hand hygiene dispenser<br>outside room door<br>knobs<br>counter<br>cabinet handles/front<br>light switches<br>barcode scanner<br>computer shelf, keyboard, mouse, monitor. This worker discarded used cloths outside the room<br><br>This guy left the room from 10:31-12:46. Went to EVS closet to put more OxyCide™ on cloths as they were too dry (which is 2 min 15 s)
2	12:56	17:56	5:00	Pillows<br>light switches<br>Bed (13:50-16:50)<br>BR sink (17:10-17:20)<br>toilet plumbing<br>toilet seat
3	18:36	24:18:00	5:42	equipment on room walls<br>white board<br>window sill/counter<br>HVAC top<br>bedside table<br>pillows<br>overbed table (19:50-21:49)<br>chair (22:19-22:46)<br>walker<br>small folding chair seat<br>Wall outlets<br>Cleaned portable commode from 23:40 - 24:09
		total time using cloths	13:56	
		% time using cloths	34	

Table 4-4. EVS Worker 3

Hospital #	EVS Worker #	Approximate room and bathroom area cleaned (sq. ft) and location	Total time spent cleaning (min:ss)	Total # OxyCide™ cloths used
1	3	NA	26:32:00	3
**Cloth #**	**Time grabbed**	**Time of discard**	**Total time used**	**Objects cleaned and observations**
1	2:00	10:16	8:16	hand hygiene dispenser<br>light switches<br>door knobs<br>computer<br>tray/mouse/keyboard<br>Thermometer<br>overbed table<br>bedside stand top<br>blood pressure cuff and cords<br>2nd overbed table<br>window sill<br>2nd vital sign machine and cords. This worker discarded used cloths outside the room<br><br>There was some time spent waiting for patient to get into next bed
2	10:18	13:26	3:08	pillows<br>telephone<br>call button<br>mattress<br>rails<br>footboard<br>bed frame (partial)
3	13:54	15:05	1:11	bathroom door knobs<br>bathroom light switches<br>Bathroom grab bar<br>sink<br>toilet handle<br>bedpan cleaner<br>toilet seat<br>toilet rim and exterior
		total time using cloths	12:35	
		% time using cloths	47	

Table 4-5. EVS Worker 4

Hospital #	EVS Worker #	Approximate room and bathroom area cleaned (sq. ft) and location	Total time spent cleaning (min:ss)	Total # OxyCide™ cloths used
1	4	NA	35:25:00	3
**Cloth #**	**Time grabbed**	**Time of discard**	**Total time used**	**Objects cleaned and observations**
NA	4:12	NA	NA	Used a squirt bottle of OxyCide™ and squirted extra OxyCide™ on table, bed, window sill, chair<br><br>It was unclear from the observation logs if this worker then wiped these areas
1	4:51	15:04	10:13	Bed/mattress top and bottom/frame under mattress (7:50-11:35)<br>Bedrail 10:10-10:32 and 10:40-11:00)<br>Chair (11:36-12:04)<br>overbed table including base (12:05-15:04)<br><br>sharps container<br>door knobs<br><br>phone and cord<br>call button<br>pillows<br>Blood pressure cuff<br><br>This worker discarded used cloths outside of the room<br><br>Cloth was noted to be very saturated
2	15:20	17:10	1:50	closet handles<br>Counter<br>room sink<br>light switches<br>paper towel dispenser<br>wall equipment<br>Vital signs machine<br>scanner<br>drawer handles<br>glove dispenser<br>window sill/HVAC top<br><br>Cloth was noted to be very saturated

3	18:06	21:54	3:48	door knobs<br>shelf under mirror<br>handrail<br>Toilet paper dispenser<br>hand hygiene dispenser<br>mirror<br>paper towel dispenser<br>sink (19:04-19:23<br>Toilet (19:30-20:45)<br>included use of non-OxyCide™ Johnny Mop
		total time using cloths	15:51	
		% time using cloths	45	

Table 4-6. EVS Worker 5

Hospital #	EVS Worker #	Approximate room and bathroom area cleaned (sq. ft) and location	Total time spent cleaning (min:ss)	Total # OxyCide™ cloths used
2	5	NA	38:35:00	6
**Cloth #**	**Time grabbed**	**Time of discard**	**Total time used**	**Objects cleaned and observations**
1,2,3	6:00	12:00	6:00	She grabbed three cloths for the mattress and base
4	12:00	18:00	6:00	Fourth OxyCide™ cloth retrieved and start of other high touch surface cleaning in the patient room: door handle, bedside dresser, telephone, tray table, call button, IV pole, light switches (each task takes < 1 minute so combined together). Cleaning of bedside recliner (takes > 1 minute); sheet indicates 17:00 - 18:00<br><br>Fourth OxyCide™ cloth discarded into plastic bag on cleaning cart at doorway of room: 18:00 - 22:00
5	24:00:00	27:00:00	3:00	Fifth OxyCide™ cloth retrieved for bathroom cleaning<br><br>Cleaned shower from 26:00 - 27:00
6	27:00:00	30:00:00	3:00	Sixth OxyCide™ cloth used to clean sink, other high touch items, and toilet in bathroom
		total time using cloths	18:00	
		% time using cloths	47	

Table 4-7. EVS Worker 6

Hospital #	EVS Worker #	Approximate room and bathroom area cleaned (sq. ft) and location	Total time spent cleaning (min:ss)	Total # OxyCide™ cloths used
3	6	NA	35:09:00	7
**Cloth #**	**Time grabbed**	**Time of discard**	**Total time used**	**Objects cleaned and observations**
1,2	3:23	12:30	9:07	Grabbed 2 cloths at once and brought into room<br><br>Door Knobs<br>Light switch<br>medical devices<br>Bed<br>Table<br>Chair<br><br>This worker was noted to discard used cloths at their cleaning cart in the hallway
3	12:40	14:45	2:05	Room sink<br>Counter<br>mirror<br>storage cabinets
4	14:55	15:34	0:39	Cabinets<br>light switches<br>linen hamper
5	15:40	16:05	0:25	Bathroom
6	16:20	18:24	2:04	Bathroom
7	19:35	21:12	1:37	Floor mat
		total time using cloths	15:57	
		% time using cloths	45	

Table 4-8. EVS Worker 7

Hospital #	EVS Worker #	Approximate room and bathroom area cleaned (sq. ft) and location	Total time spent cleaning (min:ss)	Total # OxyCide™ cloths used
3	7	NA	35:21:00	3
**Cloth #**	**Time grabbed**	**Time of discard**	**Total time used**	**Objects cleaned and observations**
1	3:15	10:24	7:09	medical devices<br>bed<br>table<br>chair<br><br>This worker was noted to discard used clothes at their cleaning cart in the hallway
2	10:35	12:22	1:47	room sink<br>window<br>mirror
3	12:36	15:50	3:14	Bathroom--sink<br>mirror<br>shower<br>toilet<br><br>Used OxyCide™ and another product during this time
		total time using cloths	12:10	
		% time using cloths	34	

Table 4-9. EVS Worker 8

Hospital #	EVS Worker #	Approximate room and bathroom area cleaned (sq. ft) and location	Total time spent cleaning (min:ss)	Total # OxyCide™ cloths used
4	8	NA	56:43:00	13
**Cloth #**	**Time grabbed**	**Time of discard**	**Total time used**	**Objects cleaned and observations**
1	3:55	6:25	2:30	Grabbed 3 cloths all at one time and placed them all on the mattress<br><br>Bed mattress<br><br>This worker was noted to discard used cloths on the floor of the room
2	6:25	7:50	1:25	bed frame top
3	7:50	9:40	1:50	bed frame bottom
4	9:56	12:20	2:24	Grabbed 4 more cloths and cleaned sink, cabinets<br>linen hamper
5	12:20	13:40	1:20	Bench<br>Strap<br>pillows<br>TV
6	13:40	16:11	2:31	Drawers<br>mobile equipment<br>shelves<br>white board
7	16:11	17:20	1:09	Couch inside and out
8	17:20	20:32	3:12	Chair<br>Equipment<br>overbed table
9	20:32	22:56	2:24	computer<br>trashcan<br>desk<br>wall equipment<br>nurse call button<br>cabinets
10	22:56	27:34:00	4:38	IV pole<br>window<br>shelves<br>Got new gloves, saturated more cloths, squirted OxyCide™ into toilet

11	30:57:00	33:12:00	2:15	Grabbed 3 more cloths<br><br>Bathroom walls including shower<br>Grab bars<br>Shower measures 36" x 60" x 84"
12	33:12:00	34:31:00	1:19	toilet
13	34:31:00	35:48:00	1:17	cleaned walls
		total time using cloths	28:14:00	
		% time using cloths	50	

Table 4-10. EVS Worker 9

Hospital #	EVS Worker #	Approximate room and bathroom area cleaned (sq. ft) and location	Total time spent cleaning (min:ss)	Total # OxyCide™ cloths used
4	9	NA	59:21:00	10
**Cloth #**	**Time grabbed**	**Time of discard**	**Total time used**	**Objects cleaned and observations**
1	0:00	2:32	2:32	Grabbed 8 cloths and placed them around the patient room<br><br>bench, drawers<br><br>This worker placed used cloths in a covered bag on their cleaning cart
2	2:32	4:02	1:30	Pillows, overbed table
3	4:02	5:02	1:00	Big, expandable couch
4	5:02	7:05	2:03	Used to clean: couch cushions, inside couch, couch drawer<br><br>Cleaned with both cloth 3 and 4 in hand - one in each hand
5	7:05	12:03	4:58	chair/recliner<br><br>Prep/organize cords, equipment, adjust bed
6	12:03	17:24	5:21	bed frame and mattress
7	17:24	23:33	6:09	call button, bedside table, cabinets, cords, hoses, equipment
8	23:33	27:55:00	4:22	bedside cabinets, desk, computer, walker<br><br>main cabinets, doors, closet<br><br>He emptied the trash after cleaning the walker and then continued cleaning with the cloth. After cleaned the other objects, he squirted OxyCide™ into the toilet bowl

9	27:55:00	33:50:00	5:55	Bathroom commode, handrails, shower faucet, hose, sink, checked and refilled paper towels, mopped out the inside of the toilet, wiped outside of toilet<br><br>Shower measures 36" x 60" x 84" but they only cleaned the faucet and hose.
10	33:50:00	43:14:00	9:24	toilet seat, sink, mirror<br><br>During this time, he retrieved the mop and mopped the bathroom floor. He then went back and cleaned the bathroom door handle, the room sink, equipment, another pillow, and hand hygiene dispensers
		total time using cloths	19:14	
		% time using cloths	73	

Protocol for Hospital Time-Activity Studies

**The focus is on understanding how long EVS workers are physically handling an OxyCide wetted cloth.

Does the hospital have a room cleaning process flow other than our PRP flow chart that they follow? If so, see if you can obtain an SOP or graphic that explains it.

In each hospital, observe 4 different EVS workers performing their standard discharge cleaning procedure using OxyCide in the same type of single patient room.

Select 4 EVS workers who regularly clean the selected type of single patient room to participate. Let them know that the observer will simply be writing down the timing and location of their tasks while they follow their normal procedure for room cleaning.

**Make it clear that this is not test of their efficiency or their cleaning technique, but rather is a study designed to collect data on the normal timing of OxyCide™ wetted cloth handling during the standard procedures the EVS worker has been trained to follow for discharge cleaning of the single patient room.

Collect one Log (see example and Log below) for each of the 4 selected EVS workers during discharge cleaning of the selected single patient room and bathroom.

Document total elapsed time in the room from start to finish – from the time they walk into the room until they walk out.

For tasks NOT involving OxyCide wetted cloth use: Document elapsed time from start of a task in minutes/seconds to the nearest quarter minute of time (e.g. pulling trash, restocking supplies, making the bed).

For tasks that DO involve OxyCide wetted cloth use: Document the exact amount of time, what they cleaned and how they disposed of each individual wetted OxyCide cloth used.

- For tasks involving OxyCide wetted cloth use for items that take > 1 minute to clean (e.g. large items like bed, chair, shower stall), please record the exact start and finish time to the point where they discard the cloth or move to the next task.
- For tasks involving OxyCide wetted cloth use that take < 1 minute to clean, combine cleaning time for all objects until they discard the cloth. See example at the 8:00 minute mark below.
- If they are NOT mopping floors/walls with OxyCide, time to nearest 15 seconds
- If they ARE mopping floors/walls with OxyCide, document exact time of mopping.
- Record the time when each wetted cloth is picked up and when it is considered spent, and identify the exact location where each spent cloth is stored.

Please document how the shower was cleaned (OxyCide or list other product, wiped vs sprayed, rinsed).

Measure the LxWxH of the shower stall here __________________. Do this regardless of whether the stall was cleaned as part of the discharge cleaning process

Please provide notes on any hospital-specific procedures that might impact the nature and extent of OxyCide™ exposures to EVS workers during single patient room discharge cleaning. (e.g. they didn't have the OxyCide bucket covered, or the cloths they were using were over saturated).

TIME/ACTIVITY LOG

Elapsed Time (MM:SS)	Location (Room/Bathroom)	Activity Description<br>**Record exact MM:SS for objects that take > 1 min to clean**
00:00		Removal of trash, linen and other items from patient room to prepare for cleaning
		1st OxyCide™ cloth retrieved and used to clean<br>List items cleaned with first cloth here:
		1st OxyCide™ cloth discarded<br>Describe where discarded cloths are stored here:
		2nd OxyCide™ cloth retrieved and used to clean<br>List items cleaned with second cloth here:
		2nd OxyCide™ cloth discarded<br>Describe where stored if different than first cloth:
		3rd OxyCide™ cloth retrieved and used to clean<br>List items cleaned with third cloth here:
		3rd OxyCide™ cloth discarded
		4th cloth retrieved and used to clean<br>List items cleaned with 4th cloth here:
		4th OxyCide™ cloth discarded
		5th OxyCide™ cloth retrieved and used to clean<br>List items cleaned with 5th cloth here:
		5th OxyCide™ cloth discarded
		Perform Hand hygiene
		Retrieve linens and make the bed
		Retrieve supplies and replace trash/linen liners, restock bathroom supplies
		Retrieve mop cleaning supplies from cart
		Final cleaning checks in room and mop floor in room
		Final cleaning checks in bathroom and mop floor in bathroom
		Room cleaning completed and EVS worker exits room<br>**End of last step = Total Elapsed Time**

EXAMPLE: TIME/ACTIVITY LOG

Time	Location	Task/Activity Description
00:00	Room	Removal of trash and other items from patient room to set for cleaning
1:15	Room	Removal of bed linens
3:30	Room	First OxyCide™ cloth retrieved and start of bed and mattress cleaning
7:45	Room	First OxyCide™ cloth discarded into plastic bag on cleaning cart at doorway
8:00	Room	Second OxyCide™ cloth retrieved and start of other high touch surface cleaning in the patient room: door handle, bedside dresser, telephone, tray table, call button, IV pole, light switches (each task takes < 1 minute so combined together)
10:45	Room	Cleaning of bedside recliner (takes > 1 minute)
12:00	Room	Second OxyCide™ cloth discarded into plastic bag on cleaning cart at doorway
12:15	Bathroom	Perform preliminary cleaning and restocking inside bathroom
14:30	Room	Third OxyCide™ cloth retrieved for bathroom cleaning
14:45	Bathroom	Shower cleaning with third OxyCide™ cloth
16:30	Room	Third OxyCide™ cloth discarded into plastic bag on cleaning cart at doorway and fourth OxyCide™ cloth retrieved for continued bathroom cleaning
16:45	Bathroom	Fourth OxyCide™ cloth used to clean sink, other high touch items, and toilet in bathroom
18:45	Room	Fourth OxyCide™ cloth discarded into plastic bag on cleaning cart at doorway
19:00	Room	Retrieve linens, make the bed, and complete room preparation tasks
22:45	Room	Retrieve mop cleaning supplies from cart for bathroom floor cleaning
23:00	Bathroom	Final cleaning checks in bathroom and mop floor in bathroom
24:45	Room	Final cleaning checks in room and mop floor in room
28:15	Room	Room cleaning completed and EVS worker exits room

Table 4-11. Raw Data from Phase 2 of Hospital Time-Activity Studies

ID	Total # of Cloths Used	Average Time per Cloth (min)	of Cleaning Time Using Cloths	Total Cleaning Time (min)	Total Time Using Cloths (min)	Percent of Cleaning Time in Patient Room	Using Cloths in Patient Room (min)	Percent of Cleaning Time in Bathroom	Time Using Cloths in Bathroom (min)	Isolation room?	In-room shower ?	Is a disinfection solution being used on the floor?	Timer	Time in Room (min)	Bed	Bed (min)	Bedside Table	Bedside Table (min)	Call Button	Call Button (min)	Chair	Chair (min)	Door Handle - Patient Room	Door Handle Pt Rm (min)	IV Pole	IV Pole (min)	Light Switch - Patient Room	Light switch Pt Rm (min)	Sink - Patient Room	Sink Pt Rm (min)	Telephone	Phone (min)	Tray Table	Tray Table (min)	Bedpan Cleaner	Bedpan Cleaner (min)	Door Handle - Bathroom	Door Handle Bathrm (min)	Flush Handle	Flush Handle (min)	Light Switch - Bathroom	Light Switch Bath (min)	Shower	Shower (min)	Sink-Bathrm (min)	Toilet Seat (min)	Comments
Standard Patient Room Cleaning:																Individual Item Cleaning Times in Patient Room:																				Individual Item Cleaning Times in Bathroom:											
S149	7	1.30	24.4%	37.43	9.12	21.2%	7.94	3.2%	1.18	No	Yes	No	37:25.45	37.43	5:27.07	5.45	0:14.62	0.25	0:01.67	0.03	1:04.37	1.07	0:00.78	0.01	0:22.40	0.37	0:00.95	0.01	0:21.98	0.37			0:23.02	0.38			0:01.40	0.02	0:06.16	0.1	0:02.32	0.04	0:09.40	0.16	0.69	0.17	No phone in room
S146	9	1.16	20.3%	51.33	10.43	15.0%	7.7	5.3%	2.73	No	Yes	No	51:19.98	51.33	3:40.64	3.68	0:51.09	0.85	0:00.54	0.01	1:06.70	1.12			0:32.32	0.54	0:01.51	0.03	0:50.14	0.84			0:37.57	0.63			0:00.42	0.01			0:00.45	0.01			1.38	1.33	No phone, tech missed a few HTO's & shower.
S140	5	5.92		25.38	29.62		27.34	9.0%	2.28	No	Yes	No	25:22.54	25.38	9:38.61	9.65	0:52.93	0.88	1:03.24	1.05	3:02.73	3.05			5:25.09	5.42			2:58.11	2.97	3:14.17	3.25	1:04.36	1.07	0:22.01	0.37			0:23.60	0.39			1:12.53	1.21		0.31	
S137	3	3.85	79.8%	14.48	11.56	57.4%	8.31	22.4%	3.25	No	Yes	No	14:29.08	14.48	2:43.36	2.72	0:41.51	0.7	0:16.31	0.27	2:04.53	2.08	0:06.74	0.11	0:12.15	0.2	0:05.75	0.1	0:35.80	0.6	0:27.71	0.47	1:03.60	1.06	0:39.62	0.66	0:08.38	0.14	0:05.71	0.1	0:08.44	0.14	1:12.08	1.2	0.57	0.44	
S134	3	2.97	65.0%	13.71	8.91	40.7%	5.58	24.3%	3.33	No	Yes	No	13:42.43	13.71	1:38.06	1.63	0:48.09	0.8	0:07.60	0.13	0:34.27	0.57	0:05.36	0.11	0:22.84	0.38	0:03.08	0.05	0:44.86	0.75	0:19.65	0.33	0:49.48	0.83	0:26.48	0.44	0:06.35	0.11	0:13.02	0.22	0:05.00	0.08	0:55.52	0.93	0.79	0.76	
S131	2	3.80	91.8%	8.27	7.59	73.5%	6.08	18.3%	1.51	No	Yes	No	8:15.84	8.27	2:56.19	2.93	0:58.78	0.98	0:18.18	0.32	0:24.66	0.42	0:05.66	0.11			0:03.67	0.05	0:32.03	0.53	0:16.05	0.27	0:28.00	0.47	0:08.21	0.14	0:04.96	0.08	0:04.10	0.07	0:01.71	0.03	0:31.62	0.53	0.3	0.36	Non patient prepatient clean
S128	6	1.06	22.0%	28.77	6.33	20.8%	5.97	1.3%	0.36	No	No	No	28:46.07	28.77	4:10.46	4.18			0:01.82	0.03	0:25.35	0.42	0:05.13	0.11	0:01.79	0.03	0:01.87	0.03	0:41.40	0.69			0:28.50	0.48	0:03.86	0.07	0:02.94	0.05	0:02.61	0.04	0:02.83	0.05			0.07	0.08	No shower no telephone bed table same as tray
S125	11	0.78	20.9%	41.15	8.6	18.9%	7.76	2.0%	0.84	No	Yes	No	41:08.83	41.15	5:06.61	5.12	0:19.48	0.33	0:14.02	0.23	1:06.62	1.12	0:03.16	0.05	0:03.68	0.06	0:01.43	0.03	0:42.66	0.71	0:06.73	0.11			0:01.22	0.02	0:03.61	0.06	0:01.72	0.03	0:02.09	0.04	0:14.10	0.23	0.29	0.17	Bed table same as tray.
S119	4	2.82	39.6%	28.49	11.29	36.2%	10.31	3.4%	0.98	No	Yes	No	28:29.17	28.49	9:35.29	7.08	0:00.77	0.01	0:03.46	0.07	1:11.21	1.18	0:01.39	0.02	0:17.70	0.3	0:02.92	0.05	0:27.93	0.47	0:08.27	0.14	0:59.34	0.99	0:03.90	0.07	0:09.58	0.16	0:03.35	0.06	0:01.49	0.03	0:11.00	0.18	0.25	0.23	2.5 miunte delay on bed. No bedside table
S113	6	3.76	73.1%	30.82	22.53	54.2%	16.7	18.9%	5.83	No	Yes	No	30:48.89	30.82	4:38.12	4.63	1:24.29	1.42			7:48.69	7.82			0:50.22	0.84	1:59.32	1.99											4:14.49	4.24					0.73	0.86	Thin disposable cloth
S107	11	1.55	44.2%	38.57	17.03	29.3%	11.3	14.9%	5.73	No	Yes	Yes	38:34.31	38.57	3:04.00	3.07	5:34.36	5.58	0:00.52	0.01	1:09.63	1.17	0:02.74	0.05	0:02.34	0.04	0:01.10	0.02	0:05.21	0.09	0:00.48	0.01	1:15.34	1.26			0:00.58	0.01			0:01.69	0.03	2:37.41	2.62	0.22	2.85	Spray bathroom down. They used alot wipes
S104	10	1.97	46.8%	42.11	19.69	31.7%	13.35	15.1%	6.34	No	Yes		42:06.39	42.11	5:56.37	5.93	0:59.27	0.98	0:04.42	0.07	0:27.52	0.47	0:00.48	0.01			0:00.87	0.01	0:02.54	0.04	0:00.99	0.01	5:49.93	5.83			0:00.80	0.01	0:02.44	0.04	0:01.95	0.03	3:27.76	3.46	0.08	2.72	1.Pull trash, strip room. 2. Stage room for wipes
S101	5	9.63	93.5%	51.47	48.15	53.0%	27.3	40.5%	20.85	No	Yes	No	51:28.39	51.47	10:22.00	10.37	5:50.50	5.85	0:29.42	0.49	4:23.21	4.38	0:19.60	0.33	0:51.52	0.86	0:08.58	0.14			0:51.52	0.86	4:01.41	4.02	0:43.85	0.73	0:31.15	0.52	0:36.12	0.6	0:19.02	0.32	11:19.00	11.32	5.95	1.41	
S98	5	0.68	12.9%	26.52	3.42	11.3%	3.01	1.5%	0.41	No	Yes	Yes	26:31.05	26.52			0:11.56	0.2	0:06.03	0.1	0:02.92	0.05	0:06.33	0.11			0:01.60	0.03	0:02.46	0.04	0:01.57	1.95	0:31.76	0.53	0:09.30	0.16			0:00.55	0.01	0:01.30	0.02	0:02.22	0.04	0.14	0.04	extremely thorough and wiping down everything
S95	6	7.19	89.9%	47.98	43.13	65.4%	31.38	24.5%	11.75	No	Yes	No	47:58.81	47.98	8:37.50	8.63	4:07.23	4.12	0:40.17	0.67	8:38.93	8.65	0:18.22	0.3	1:05.89	1.1	0:12.77	0.21	5:16.38	5.27	0:46.01	0.77	1:39.62	1.66	0:13.12	0.22	0:12.45	0.21	0:11.99	0.2	0:10.94	0.18	7:27.20	7.45	3	0.49	Chair and sofa in room. Used extra blue cloth
S92	5	9.72	95.1%	51.06	48.58	57.8%	29.51	37.3%	19.07	No	Yes	No	51:03.66	51.06	8:41.03	8.68	3:55.40	3.92	0:35.20	0.58	4:49.73	4.83	0:24.90	0.42	2:08.56	2.14	0:23.62	0.39	5:04.95	5.08	1:02.44	1.04	2:25.99	2.43	0:40.56	0.68	0:20.30	0.34	0:20.54	0.34	0:19.73	0.33	9:23.11	9.39	5.96	2.03	
S83	7	2.35	33.3%	49.48	16.47	28.6%	14.14	4.7%	2.33	No	Yes	No	49:28.67	49.48	7:49.36	7.82	2:12.58	2.22	1:02.46	1.05	1:19.85	1.33	0:21.19	0.35	1:22.28	1.37									0:23.53	0.39	0:13.17	0.22	0:10.28	0.17	0:07.08	0.12	0:23.80	0.4	0.43	0.6	
S80	8	3.54	61.5%	46.03	28.33	46.4%	21.37	15.1%	6.96	No	Yes	No	46:01.29	46.03	11:37.17	11.62	3:49.31	3.82	0:24.59	0.42	1:41.61	1.7	0:18.91	0.32	1:19.46	1.33	1:17.91	1.3	0:21.69	0.37			0:29.16	0.49	1:13.09	1.22	2:09.90	2.17	0:09.01	0.15	0:13.96	0.23	2:02.81	2.05	1.14	0.7	Avg. clothes per discharge = 5 blue + 2 orange
S71	6	2.39	76.5%	18.72	14.32	62.4%	11.68	14.1%	2.64	No	Yes	No	18:43.12	18.72	8:54.65	8.92	0:40.58	0.68	0:07.58	0.14			0:07.43	0.12	0:28.50	0.48	0:10.05	0.17	0:13.70	0.23	0:13.63	0.23	0:42.77	0.71	0:08.95	0.15	0:05.77	0.1	0:04.93	0.08	0:05.22	0.08	1:27.78	1.46	0.51	0.26	EVS Tech has been here for 1.5 years
S68	6	1.47	52.2%	16.9	8.83	42.9%	7.25	9.3%	1.58	No	Yes	No	16:53.36	16.9	4:05.60	4.1	0:34.47	0.58	0:18.22	0.3	1:00.66	1.01	0:04.88	0.08	0:17.25	0.29	0:08.55	0.14	0:16.32	0.27	0:06.60	0.11	0:22.15	0.37	0:08.43	0.15	0:08.62	0.14	0:02.87	0.05	0:02.57	0.04	0:46.39	0.77	0.24	0.19	EVS tech has been here for 1 year
S65	6	0.87	36.8%	14.23	5.23	22.3%	3.18	14.4%	2.05	No	Yes	No	14:14.06	14.23	1:18.31	1.31	0:33.49	0.57	0:06.27	0.11			0:11.03	0.18			0:05.65	0.1	0:17.65	0.3	0:06.08	0.1	0:30.32	0.51	0:19.89	0.33	0:05.55	0.09	0:06.83	0.11	0:09.37	0.16	0:37.51	0.63	0.41	0.32	No IV pole in the room. EVS Tech since 2017
S62	9	0.91	40.0%	20.57	8.23	30.2%	6.22	9.8%	2.01	No	Yes	Yes	20:34.05	20.57	2:46.66	2.78	0:02.93	0.05	0:19.92	0.33	0:16.28	0.27	0:07.35	0.12	1:48.34	1.81	0:05.97	0.1	0:18.18	0.3	0:08.53	0.14	0:19.23	0.32	0:02.68	0.05	0:04.42	0.07	0:05.68	0.1	0:02.22	0.04	0:54.40	0.91	0.53	0.31	
S59	3	3.69	30.0%	36.88	11.07	25.3%	9.33	4.7%	1.74	No	Yes	No	36:53.18	36.88	5:05.37	5.08	1:37.66	1.63			0:27.91	0.47	0:07.17	0.12	0:28.77	0.48			1:01.63	1.03			0:30.98	0.52	0:03.77	0.06	0:07.40	0.12	0:09.75	0.16	0:02.57	0.04	0:25.61	0.43		0.93	Oxycide above floor, neutral floor cleaner 3 mops
S53	3	3.55			10.65		9.49		1.16	No	Yes	No			6:00.71	6.01	0:46.57	0.78	0:12.21	0.2	0:17.49	0.3	0:03.34	0.06					0:20.48	0.34	0:05.79	0.1	1:42.14	1.7			0:02.45	0.04	0:05.18	0.09			0:35.12	0.59	0:45	0.44	
S50	2	6.25			12.5		11.27		1.23	No	Yes	No			6:11.05	6.18	1:02.05	1.03	0:28.95	0.48	1:08.95	1.15			1:43.53	1.73			0:05.92	0.1	0:03.42	0.05	0:32.76	0.55			0:01.99	0.03	0:18.93	0.32	0:03.59	0.06	0:23.88	0.4	0.07	0.35	
S44	6	1.74			10.44		8.66		1.78	No	No	No			4:22.02	4.37	1:02.62	1.03	0:17.98	0.3	0:54.01	0.9			0:18.18	0.3					0:23.50	0.39	1:22.33	1.37			0:10.68	0.18	0:08.57	0.14					0.63	0.83	Some hto s were missed
S41	3	3.13	27.2%	34.58	9.4	24.4%	8.43	2.8%	0.97	No	Yes	No	34:35:00	34.58	5:02.56	5.05	0:18.14	0.3	0:14.49	0.25	0:27.80	0.47	0:04.54	0.08	0:31.50	0.53	0:03.38	0.06	0:27.95	0.57	0:02.20	0.04	1:05.11	1.08	0:01.91	0.03	0:06.60	0.11	0:02.36	0.04	0:02.99	0.05	0:23.77	0.4	0.21	0.13	Total time 34:35
S35	6	1.78	37.4%	28.5	10.65	32.1%	9.14	5.3%	1.51	No	Yes	No	28:30:00	28.5	3:45.53	3.77	0:26.66	0.45	0:06.12	0.1	1:53.83	1.9	0:03.60	0.06	0:41.65	0.7	0:03.30	0.06	0:27.64	0.57	0:05.90	0.1	1:25.51	1.43	0:04.04	0.07	0:02.83	0.05	0:10.40	0.17	0:02.43	0.04	0:43.17	0.72	0.37	0.09	Total time28:30
S32	4	5.11	62.8%	32.55	20.45	43.7%	14.21	19.2%	6.24	No	Yes	No	32:33:00	32.55	8:56.70	8.95	0:55.76	0.93	0:13.29	0.22	0:28.26	0.47	0:10.00	0.17	0:48.32	0.81	0:05.55	0.09	0:34.55	0.58	0:14.94	0.25	1:44.20	1.74	0:22.35	0.37	0:10.75	0.18	0:10.00	0.17	0:07.24	0.12	4:45.39	4.76	0.37	0.27	Total time 32:33
S8	2	3.61	39.9%	18.08	7.22	34.0%	6.15	5.9%	1.07	No	No	Yes	18:05.27	18.08	2:17.33	2.28	0:14.62	0.25	0:43.21	0.72	0:09.84	0.16	0:01.17	0.02	0:23.37	0.37	0:00.96	0.01					2:20.52	2.34	0:05.65	0.1	0:06.90	0.12	0:01.43	0.02	0:01.70				0.49	0.34	
Average	5.6	3.29	50.7%	31.63	15.99	37.6%	12.00	13.0%	3.99							5.59	#DIV/0!	1.42	#DIV/0!	0.31	#DIV/0!	1.73	#DIV/0!	0.14	#DIV/0!	0.90	#DIV/0!	0.22	#DIV/0!	0.92	#DIV/0!	0.49	#DIV/0!	1.29	#DIV/0!	0.29	#DIV/0!	0.20	#DIV/0!	0.29	#DIV/0!	0.09	#DIV/0!	2.09	0.96	0.67	
Std Dev	2.6	2.42	25.6%	13.18	12.13	16.8%	7.77	10.6%	5.02							2.76	#DIV/0!	1.62	#DIV/0!	0.29	#DIV/0!	2.17	#DIV/0!	0.12	#DIV/0!	1.10	#DIV/0!	0.46	#DIV/0!	1.40	#DIV/0!	0.77	#DIV/0!	1.23	#DIV/0!	0.30	#DIV/0!	0.41	#DIV/0!	0.78	#DIV/0!	0.09	#DIV/0!	3.01	1.55	0.74	
Isolation Room Cleaning:																																															
S62	6	1.31	30.1%	26.08	7.84	21.9%	5.72	8.1%	2.12	Yes	Yes	Yes	26:04.82	26.08	2:59.06	2.98	0:26.17	0.43	0:00.45	0.01	0:28.79	0.48	0:04.13	0.07	0:58.32	0.97	0:04.50	0.08	0:29.20	0.49	0:09.00	0.15	0:03.67	0.06	0:01.67	0.03	0:03.67	0.06	0:06.63	0.11	0:00.90	0.01	1:04.53	1.08	0.51	0.32	Oxy on floor
S62	4	2.11	50.2%	16.78	8.43	42.3%	7.1	7.9%	1.33	Yes	Yes	Yes	16:46.30	16.78	5:23.63	5.4	0:01.32	0.02	0:02.72	0.05	0:39.10	0.65	0:01.62	0.03			0:04.85	0.08	0:11.60	0.19	0:07.92	0.13	0:32.70	0.55	0:01.95	0.03	0:08.58	0.14	0:02.13	0.04			0:33.04	0.55	0.28	0.29	Covid room
S59	3	7.62	24.2%	94.32	22.86	23.2%	21.91	1.0%	0.95	Yes	Yes	Yes	94:19.34	94.32	16:53.32	16.88	0:03.89	0.07	0:08.09	0.14	0:45.76	0.77	0:02.75	0.05	2:41.27	2.69	0:01.98	0.03	1:08.30	1.14	0:08.76	0.14			0:02.00	0.03	0:03.09	0.05	0:04.72	0.08			0:10.74	0.18	0.43	0.18	Covid room, wait on mattress and xenex 5 mops
S59	3	8.00	24.1%	99.58	23.99	16.8%	16.77	7.3%	7.22	Yes	Yes	Yes	99:34.87	99.58	5:59.92	6	0:16.77	0.28	0:04.44	0.07	9:13.29	9.23	0:04.47	0.08	0:05.13	0.09	0:20.82	0.35	0:21.62	0.36	0:01.26	0.02	0:17.50	0.29	0:13.50	0.23	0:03.01	0.05	0:01.97	0.03	0:01.03	0.02	2:13.87	2.23	3.59	1.07	Water running all discharge, 3 mops, h20 stains
S56	10	1.75	38.3%	45.73	17.52	36.3%	16.58	2.1%	0.94	Yes	No	No	45:44.03	45.73	9:13.29	9.22	1:58.40	1.98	0:25.09	0.42	1:57.99	1.97	0:03.49	0.06	1:50.03	1.83	0:08.87	0.15	0:39.36	0.66			0:17.83	0.29	0:06.10	0.1	0:04.44	0.07	0:02.02	0.03	0:02.77	0.04	0:02.97	0.05	0.37	0.28	Medical ICU Rooms - long term COVID patient.
Average	5.2	4.16	33.4%	56.50	16.13	28.1%	13.62	5.3%	2.51							8.10	#DIV/0!	0.56	#DIV/0!	0.14	#DIV/0!	2.62	#DIV/0!	0.06	#DIV/0!	1.40	#DIV/0!	0.14	#DIV/0!	0.57	#DIV/0!	0.11	#DIV/0!	0.30	#DIV/0!	0.08	#DIV/0!	0.07	#DIV/0!	0.06	#DIV/0!	0.02	#DIV/0!	0.82	1.04	0.43	
Std Dev	3	3.35	11.1%	38.42	7.70	10.7%	6.93	3.5%	2.68							5.39	#DIV/0!	0.81	#DIV/0!	0.16	#DIV/0!	3.74	#DIV/0!	0.02	#DIV/0!	1.12	#DIV/0!	0.13	#DIV/0!	0.36	#DIV/0!	0.06	#DIV/0!	0.20	#DIV/0!	0.09	#DIV/0!	0.04	#DIV/0!	0.04	#DIV/0!	0.02	#DIV/0!	0.88	1.43	0.36	
Apparent Touch-Up Cleaning:																																															
S5	1	6.23		0.87	6.23		3.45		2.78	No	Yes	No	0:52.72	0.87	0:26.79	0.45	0:30.15	0.5	0:17.27	0.29	0:21.98	0.37	0:32.71	0.55	0:14.85	0.25	0:19.56	0.33	0:21.84	0.36	0:09.63	0.16	0:11.40	0.19	0:31.55	0.53	0:30.36	0.51	0:29.63	0.49	0:10.44		0:31.47	0.53	0.21	0.51	
S2	1	6.67		0.85	6.67		4.38		2.29	No	Yes	Yes	0:51.28	0.85	0:27.08	0.45	0:25.92	0.43	0:23.14	0.39	0:25.93	0.43	0:23.63	0.39	0:24.54	0.41	0:24.89	0.42	0:22.14	0.37	0:32.16	0.54	0:32.74	0.55	0:22.64	0.38	0:20.27	0.34	0:10.25	0.17	0:26.45		0:28.10	0.47	0.46	0.47	More comments
Average	1	6.45																																													
Specialty Rooms Without Bathroom Cleaning:																																															
S86	7	1.99	35.2%	39.68	13.95	32.8%	13.03			No	No	No	39:41.17	39.68	5:32.07	5.53	2:15.77	2.27			1:27.62	1.47			2:25.91	2.43	0:11.00	0.18	0:34.02	0.57			0:34.73	0.58					0:13.46	0.23						0.69	Neurology patient room - no bathroom
S26	8	0.97			7.76		7.15			No	No	No			5:02.70	5.05	0:37.47	0.63	0:03.17	0.05	0:16.39	0.27	0:05.56	0.09	0:48.21	0.81	0:04.44	0.07	0:03.01	0.05			0:07.89	0.13											0.41	0.2	Neuro ICU Room, toilet and sink were in the oper
S17	4	1.02	59.8%	6.82	4.08	59.8%	4.08			No	No		6:49.37	6.82	1:06.80	1.12			1:38.75	1.65	0:07.48	0.13			0:19.09	0.32	0:01.05	0.02	0:11.64	0.19			0:39.14	0.65													
S14	2	1.76	34.6%	10.15	3.51	34.6%	3.51			No	No	Yes	10:09.21	10.15	2:23.00	2.38	0:10.71	0.18	0:14.75	0.25	0:12.25	0.21			0:08.88	0.15			0:18.09	0.3	0:02.06	0.04															
S11	7	2.16	29.7%	50.75	15.09	28.0%	14.19			No	No	Yes	50:44.79	50.75	7:44.07	7.73	0:51.79	0.87	0:38.85	0.65	2:18.64	2.32	0:13.12	0.22	0:36.55	0.61	0:02.31	0.04			0:12.70	0.21	1:32.05	1.54			0:04.76	0.08	0:00.48	0.01	0:00.74				0.46	0.35	
Average	5.6	1.58	39.8%	26.85	8.88	38.8%	8.39									4.36	#DIV/0!	0.99	#DIV/0!	0.65	#DIV/0!	0.88	#DIV/0!	0.16	#DIV/0!	0.86	#DIV/0!	0.08	#DIV/0!	0.28	#DIV/0!	0.13	#DIV/0!	0.73	#DIV/0!		#DIV/0!	0.08	#DIV/0!	0.12	#DIV/0!		#DIV/0!		0.44	0.41	
Std Dev	2.51	0.55	13.6%	21.72	5.42	14.3%	4.98									2.63	#DIV/0!	0.90	#DIV/0!	0.71	#DIV/0!	0.98	#DIV/0!	0.09	#DIV/0!	0.91	#DIV/0!	0.07	#DIV/0!	0.22	#DIV/0!	0.12	#DIV/0!	0.59	#DIV/0!		#DIV/0!		#DIV/0!		#DIV/0!		#DIV/0!			0.25	

5. Mass Transfer Study

Mass Transfer Study with Use of the OxyCide™Product in the Monell Chamber

A PAA mass transfer protocol was developed and implemented for assessing airborne PAA exposures to OxyCide™ at an elevated use solution dilution of 4 ounces of concentrate per gallon, 33.3% higher than the antimicrobial target dilution rate for antimicrobial efficacy of 3 ounces per gallon used in the main study at Monell. Varied conditions for wetted cloth use frequency were examined in sets of 4 trials of 20-minutes each using the same application protocol in the Monell environmental chamber:

1) use of one wetted cloth for 20 minutes of continual cleaning;

2) use of two wetted cloths (10 minutes each) for 20 minutes of continual cleaning; and

3) use of 4 wetted cloths (5 minutes each for 20 minutes of continual cleaning which mimicked the main study upper bound use pattern).

The goals of this study were to: 1) determine the magnitude of change in airborne PAA, AA, and HP concentrations in the Monell chamber using the standard protocol for the human chamber studies, but increasing the OxyCide™ dilution rate to 4 oz concentrate per gallon water, and 2) to estimate surface areas for each object sanitized and measure the mass of solution transferred to the sanitized surface areas for 3 use scenarios (1 cloth used for 20 minutes, 2 cloths used for 10 minutes each, and 4 cloths used for 5 minutes each). The steps of the study protocol are outlined below:

1. Two solutions with an OxyCide™ content of 4 ounces of concentrate per gallon were mixed within 1 hour prior to start of cloth usage.

2. Prior to start of the trials, measurements were taken of the surface area of sanitation for all surfaces to be wiped in series during this testing (this was done by one of the authors, BK).

3. For each trial, a top-loader balance was used to weigh each wetted cloth before and after it was used by BK in sanitizing the designated surfaces within the Monell chamber. The cloth weights for each trial were recorded in a log for that day. All chamber operating conditions were set to be identical to the standard protocol and a minimum of 10 minutes was allowed between trials for clearing prior trial residual vapors. The sequence of trials was from the lowest to highest number of wetted cloths used per trial: 1) one cloth per 20-minute trial; 2) two cloths for 10 minutes each per trial; and 3) four cloths for 5 minutes each per trial. Four trials of each condition were completed.

4. For each trial, BK recorded the number of times that the full sequence of surface cleaning was completed and the stopping point in the sequence for each cloth used, which was used to estimate the total surface area wiped per cloth in each trial.

5. BK wore the sampling vest for collecting the air samples while sanitizing surfaces during each trial in accordance with the standard Monell protocol (except that no subjective ratings or biological samples were collected); video recordings were taken for the first trial of each set.

6. Air sampling tubes for each trial were capped and placed in pre-labeled bags by Monell staff for each of the three test conditions per the standard protocol. The samples were stored refrigerated under the standard protocol and shipped to Environmental Analytical Services (EAS) labs within 24-hours after completion of testing. EAS analyzed each sample for PAA, AA, and HP in accordance with the standard protocol.

The full study protocol can be found at the end of this Appendix. Table 5-1 summarizes the study results for use of 1, 2, or 4 wetted clothes for simulated disinfecting in the Monell chamber. The following tables show the raw data collected from the study.

Table 5-1. Summary of Mass Transfer Study Results for Use of 1, 2, or 4 Wetted OxyCide™ Cloths for Simulated Disinfection in the Monell Chamber*

# of Cloths	Statistic	Soln. mass loss from cloth(s) (g)	Soln. mass loss per time (g/min)	Total area wiped (m^2)	Number of full cycles wiped	Soln. mass loss per area wiped (g/m^2)	Cleaning rate (m^2/min)	PAA Mass in Chamber (g)	Breathing Zone PAA (mg/m^3)	Estimated Airborne PAA with Complete Evaporation mg/m^3)[1]	Breathing Zone PAA Fraction (%)
1	Mean	82.4	4.1	32.2	3.8	2.56	1.6	0.14	277	1876	14.9%
	S.D.	4.0	0.2	1.8	0.2	0.10	0.09	0.007	86	90	5.0%
2	Mean	141	7.1	36.2	4.2	3.89	1.8	0.25	283	3200	8.9%
	S.D.	18	1.1	4.1	0.5	0.22	0.20	0.030	31	399	0.4%
4	Mean	196	9.8	43.6	5.1	4.55	2.2	0.34	314	4470	7.0%
	S.D.	14	1.0	4.5	0.5	0.76	0.35	0.030	38	323	0.4%
All Data	Mean	140	8.2	37.3	4.4	3.70	2.0	0.24	291	3182	10.2%
	S.D.	50	2.3	5.9	0.7	1.0	0.37	0.090	55	1139	4.4%

*OxyCide™ use solution mixed at 4 oz/gal for 20-min wiping period. Estimated Breathing Zone PAA with Complete Vaporization was estimated as Cave = (G/Q) * [1 + (1/(ACM*T) * (EXP(-ACM*T)$^{-1}$)] with G = grams of PAA lost per min; Q = air flow at 2.06 m^3/min; ACM = air changes per minute at 0.09; and T = exposure time of 20 min. Breathing Zone PAA Fraction = Breathing Zone PAA in mg/m^3 divided by Estimated Airborne PAA with Complete Vaporization in mg/m^3.

There is a significant difference in average Solution Mass Loss, Solution Mass Loss per Time, Total Area Wiped, Full Cycles Wiped, and Cleaning Rate between the three groups using either 1, 2, or 4 cloths per trial ($p<0.01$).

There is no significant difference in average Breathing Zone PAA between the three groups using either 1, 2, or 4 cloths per trial ($p=0.641$).

There is a significant difference in average Solution Mass in Chamber between the three groups using either 1, 2, or 4 cloths per trial ($p<0.001$).

There is a significant difference in average Estimated Breathing Zone PAA with Complete Vaporization between the three groups using either 1, 2, or 4 cloths per trial (p=<0.001).
There is a significant difference in average Breathing Zone PAA Fraction between the three groups using either 1, 2, or 4 cloths per trial (p=0.010).
[1] This was calculated using standard methods (American Industrial Hygiene Association (AIHA). Mathematical Models for Estimating Occupational Exposure to Chemicals, 2nd Edition. Eds.: Keil, C. B., Simmons, C. E., Anthony, T.R. AIHA, 2009.)

Table 5-2. Correlation Analysis for Selected Parameters in the Mass Transfer Studies of OxyCide™ Use at 1, 2, and 4 Wetted Cloths per 20-min Trial

Parameter X	Parameter Y	Fit Type	Slope	Intercept	R^2 value
# of Cloths Used	Cleaning Rate (m^2/min)	Linear	0.2x	1.4	1
# of Cloths Used	Soln. Mass Loss from Cloths (g)	Logarithmic	82.16 ln(x)	82.78	0.9999
		Linear	36.53x	54.5	0.9598
# of Cloths Used	Soln. Mass Loss per Time (g/min)	Logarithmic	4.10 ln(x)	4.16	0.9995
		Linear	1.82x	2.75	0.9555
# of Cloths Used	Soln. Mass per Area Wiped (g/m^2)	Logarithmic	1.44 ln(x)	2.67	0.9636
		Linear	0.616x	2.23	0.861
# of Cloths Used	Airborne PAA (mg/m^3)	Exponential	$263e^{0.0432}$		0.9727
		Linear	12.79x	261.5	0.9673
Soln. Mass Loss from Cloths (g)	Soln. Mass Loss per Time (g/min)	Linear	0.05x	0.028	0.9999
Soln. Mass Loss from Cloths (g)	Soln. Mass per Area Wiped (g/m^2)	Logarithmic	2.31 ln(X)	-7.61	0.9964
		Linear	0.018x	1.22	0.9678
Soln. Mass Loss from Cloths (g)	Airborne PAA (mg/m^3)	Exponential	$249.6e^{0.0011x}$		0.8722
		Linear	0.323x	246.2	0.8599
Soln. Mass per Area Wiped (g/m^2)	Airborne PAA (mg/m3)	Exponential	$236.7e^{0.0562x}$		0.7268
		Linear	16.6x	230.6	0.7143
Soln. Mass Loss per Time (g/min)	Airborne PAA (mg/m3)	Exponential	$249.6e^{0.0219x}$		0.8649
		Linear	6.45x	246	0.8524

Table 5-3. Mass Transfer Studies Data Log

Trial #	# of Cloths	Time of cloth use (min)	Cloth weight in (g)	Cloth weight out (g)	Mass loss from cloth (g)	Start wipe item	# full cycles	Stop wipe item
1	1	20	174.03	90.78	83.25	Bed tray	3	Headboard
2	1	20	163.16	84.72	78.44	Bed tray	3	Mattress
3	1	20	167.74	87.49	80.25	Bed tray	3	Mattress
4	1	20	180.76	91.43	89.33	Bed tray	4	1/2 of sink
5	2	12	165.98	99.93	66.05	Bed tray	2	1/2 of toilet
		8	190.24	131.99	58.25	1/2 of toilet	1	Headboard
6	2	10	172.84	110.03	62.81	Bed tray	2	Sink
		10	173.24	107.82	65.42	Toilet	2	Toilet
7	2	10	176.33	108.01	68.32	Bed tray	2	Soap Dispenser
		10	189.2	108.87	80.33	Soap Dispenser	2	1/2 recliner
8	2	10	178.88	106.47	72.41	Bed tray	2	Phone
		10	200.28	111.5	88.78	Recliner	2	1/2 Mattress
9	4	5	184.92	133.56	51.36	Bed tray	1	Sink
		5	178.62	126.26	52.36	Toilet	1	Toilet
		5	183.32	131.46	51.86	Pull bars	1	Phone
		5	199.72	138.72	61	Recliner	1	2/3 Mattress
10	4	5	169.08	122.18	46.9	Bed tray	1	1/2 Toilet
		5	189.7	142.42	47.28	1/2 Toilet	1	Phone
		5	176.51	123.1	53.41	Recliner	1	1/2 Mattress
		5	163.34	118.9	44.44	1/2 Mattress	1	1/2 Toilet
11	4	5	170.05	125.61	44.44	Bed tray	1	Pull bars
		5	167.32	123.5	43.82	Soap Dispenser	1	Recliner
		5	178.41	124.3	54.11	Headboard	1	1/2 Mattress
		5	175.92	127.18	48.74	1/2 Mattress	1	Bed tray
12	4	5	161.98	115.35	46.63	Bed tray	1	1/3 Mattress
		5	175.85	122.92	52.93	2/3 Mattress	1	Phone
		7.5	180.46	116.55	63.91	Recliner	1	Bed tray
		2.5	169.53	147.75	21.78	Sink	0	Mattress

Table 5-4. Summary of Dimensions and Surface Areas for High Touch Non-Porous Surfaces Used in the Monell Environmental Chamber Studies Simulating Hospital Environmental Service Worker Disinfection of Patient Rooms

Items Wiped in Sequence	Dimensions (inches)	Surface Area (sq. in.)	Surface Area (sq. ft.)	Surface Area (sq. m.)
Bed Tray Table	30 x 15 x 0.75	518	3.6	0.334
Bathroom Sink	34 x 20 x 4	724	5.0	0.467
Bathroom Sink Faucet	6 x 2 x 0.75 base; 2 handles 5 x 1.5; spout stem 13	63	0.44	0.041
Bathroom Sink Bowl	18 x 14 x 7 deep	402	2.8	0.259
Toilet Top Side	14 x 26 x 13	134	0.9	0.086
Toilet Bowl	15 x 10.5 x 8 deep	402	2.8	0.259
Toilet Base & Underside	Complex	442	3.1	0.285
High Touch Surfaces Wall:				
Stainless Steel Pull Bars (2)	28 x 1.5 diam. Tube; 3 round end plates	146	1.0	0.094
Soap Dispenser	6 x 11 x 4	178	1.2	0.115
Stainless Steel Door Handles (2)	4 x 1 x 0.25 lever; 1 x 2 cylinder; 2.5 round end plates	32	0.22	0.021
Stainless Steel Swich Plates (2)	4.5 x 4.5	41	0.28	0.026
Paper Towel dispenser	10.5 x 7.5 x 6 (5 faces)	326	2.3	0.210
Slimline Corded Telephone	8 x 2.25 x 2; 8 x 2.25 x 3; 54L coiled cord 0.3W	166	1.2	0.107
Bedside Chest Top Surface	17 x 15.5 x 1	329	2.3	0.212
Vinyl Recliner Seat Top/Upright	31 x 20 x 7	1054	7.3	0.680
Vinyl Recliner Seat	25 x 19	475	3.3	0.306
Vinyl Recliner Arms & Sides (2)	Top 48 x 4; inner sides 6.5 x 19; outer sides 20 x 28	1335	9.3	0.861
Bed Headboard	Top 66 x 15 x 2; Inner side 36 x 15; outer side 36 x 15	1212	8.4	0.782
Vinyl Mattress Sides	Ends 36 x 6; Sides 80 x 6	1392	9.7	0.898
Vinyl Mattress Top	36 x 80	2880	20.0	1.858
Bed Footboard	Top 60 x 12 x 2; Inner side 36 x 12; outer side 36 x 12	984	6.8	0.635
All Disinfected Surfaces		13235	91.9	8.54
All Disinfected Surfaces in Bathroom		2818	19.6	1.82
All Disinfected Surfaces--Chair		2864	20	1.85
All Disinfected Surfaces--Bed		6468.0	44.9	4.17

Table 5-5. Summary of High Touch Non-Porous Surfaces Wiped and Mass Deposited During Continuous 20-min Wiping with OxyCide™ in the Monell Environmental Chamber Studies Simulating Hospital Environmental Service Worker Disinfection of Patient Rooms Using 1, 2, or 4 Cloths per Trial

Trial #	Cloth #	Full Cycles Completed	Starting Point in Cycle	Stopping Point in Cycle	Timing to Complete (min)	Mass Loss from Cloth (g)	Rate Applied (g/min)	Surface Area (sq. m.)	Cleaning Rate (sq. m./min)	Equiv. cycles wiped	Rate Applied (g/sq. m.)
1 Cloth for 20-min											
1	1	3	Bed Tray	Headboard	20	83.25	4.2	30.7	1.5	3.6	2.7
2	1	3	Bed Tray	Mattress	20	78.44	3.9	31.6	1.6	3.7	2.5
3	1	3	Bed Tray	Mattress	20	80.25	4.0	31.6	1.6	3.7	2.5
4	1	4	Bed Tray	1/2 of Sink	20	87.49	4.4	34.9	1.7	4.1	2.5
					Average per Trial	82.4	4.1	32.2	1.6	3.8	2.6
2 Cloths for 10-min Each					S.D.	4.0	0.2	1.8	0.09	0.2	0.1
5	1	2	Bed Tray	1/2 of Toilet	12	66.05	5.5	18.5	1.5		3.6
	2	1	1/2 of Toilet	Headboard	8	58.25	7.3	12.2	1.5		4.8
					Total Per 20-min	124		30.7		3.6	4.0
6	1	2	Bed Tray	Sink	10	62.81	6.3	18.2	1.8		3.5
	2	2	Toilet	Toilet	10	65.42	6.5	17.7	1.8		3.7
					Total Per 20-min	128		35.9		4.2	3.6
7	1	2	Bed Tray	Soap Dispenser	10	68.32	6.8	19.0	1.9		3.6
	2	2	Soap Dispenser	1/2 Recliner	10	80.33	8.0	18.7	1.9		4.3
					Total Per 20-min	149		37.7		4.4	3.9
8	1	2	Bed Tray	Phone	10	72.41	7.2	19.4	1.9		3.7
	2	2	Recliner	1/2 Mattress	10	88.78	8.9	21.0	2.1		4.2
					Total Per 20-min	161		40.4		4.7	4.0
					Average per Trial	141	7.1	36.2	1.8	4.2	3.9
4 Cloths for 5-min Each					S.D.	17.4	1.0	4.1	0.20	0.5	0.2
9	1	1	Bed Tray	Sink	5	51.36	10.3	9.6	1.9		5.4
	2	1	Toilet	Toilet	5	52.36	10.5	9.2	1.8		5.7
	3	1	Pull Bars	Phone	5	51.86	10.4	9.0	1.8		5.8
	4	1	Recliner	2/3 Mattress	5	61	12.2	11.1	2.2		5.5
					Total Per 20-min	217		38.9		4.6	5.6

10	1	1	Bed Tray	1/2 Toilet	5	46.9	9.4	9.9	2.0		4.7
	2	1	1/2 Toilet	Phone	5	47.28	9.5	9.4	1.9		5.0
	3	1	Recliner	2/3 Mattress	5	53.41	10.7	13.0	2.6		4.1
	4	1	1/2 Mattress	1/2 Toilet	5	44.44	8.9	11.3	2.3		3.9
					Total Per 20-min	192		43.7		5.1	4.4
11	1	1	Bed Tray	Pull Bars	5	44.44	8.9	10.4	2.1		4.3
	2	1	Soap Dispenser	Recliner	5	43.82	8.8	11.1	2.2		3.9
	3	1	Headboard	1/2 Mattress	5	54.11	10.8	10.7	2.1		5.1
	4	1	1/2 Mattress	Bed Tray	5	48.74	9.7	10.3	2.1		4.7
					Total Per 20-min	191		42.3		5.0	4.5
12	1	1	Bed Tray	1/3 Mattress	5	46.63	9.3	14.6	2.9		3.2
	2	1	2/3 Mattress	Phone	5	52.93	10.6	13.1	2.6		4.0
	3	1	Recliner	Bed Tray	7.5	63.91	8.5	14.9	2.0		4.3
	4	1	Sink	Mattress	2.5	21.78	8.7	7.1	2.8		3.1
					Total Per 20-min	185		49.6		5.8	3.7
					Average per Trial	196	9.8	43.6	2.2	5.1	4.6
					S.D.	13.9	1.0	4.5	0.35	0.5	0.8
					Overall Average	140	8.2	37.3	2.0	4.4	3.7
					S.D.	50.0	2.3	5.9	0.37	0.7	1.0
There is a significant difference in average Mass Loss from Cloth, Rate Applied, Surface Area, Cleaning Rate, Equiv. cycles wiped, and Rate Applied between the three groups using either 1, 2, or 4 cloths per trial ($p<0.01$).											

Table 5-6. Measured Peracetic Acid (PAA) in Air and Total Mass Transferred into Monell Chamber Using 1, 2, or 4 Cloths per Trial*

Trial #	# of Cloths	Breathing Zone PAA (ppb)	Breathing Zone PAA (mg/m³)	Solution Mass in Chamber (g)	Total PAA in Chamber (g)	Estimated Airborne PAA with Complete Vaporization (mg/m³)	Breathing Zone PAA Fraction (%)
1	1	53.3	166	83.25	0.146	1896	0.0874
2	1	99.8	310	78.44	0.137	1787	0.1736
3	1	119	370	80.25	0.140	1828	0.2022
4	1	84.5	263	87.49	0.153	1993	0.1318
	Average	89.1	277	82.4	0.144	1876	0.1487
	S.D.	27.7	86	4.0	0.007	90	0.0501
5	2	84.6	263	124	0.217	2825	0.0931
6	2	80.4	250	128	0.224	2916	0.0857
7	2	98.8	307	149	0.261	3394	0.0905
8	2	100	312	161	0.282	3667	0.0850
	Average	91.0	283	140.5	0.246	3200	0.0886
	S.D.	10.0	31	17.5	0.031	399	0.0039
9	4	118.6	369	217	0.380	4943	0.0746
10	4	91.1	283	192	0.336	4374	0.0647
11	4	99.7	310	191	0.334	4351	0.0712
12	4	94.1	292	185	0.324	4214	0.0694
	Average	100.9	314	196.3	0.343	4470	0.0700
	S.D.	12.3	38	14.2	0.025	323	0.0041
	Overall Average	93.7	291.1	139.7	0.244	3182	0.1024
	S.D.	17.5	54.5	50.0	0.088	1139	0.0439

*OxyCide™ use solution mixed at 4 oz/gal for 20-min wiping period. Estimated Breathing Zone PAA with Complete Vaporization was estimated as Cave = (G/Q) * [1 + (1/(ACM*T) * (EXP(-ACM*T)-1)] with G = grams of PAA lost per min; Q = air flow at 2.06 m^3/min; ACM = air changes per minute at 0.09; T = exposure time of 20 min. Breathing Zone PAA Fraction = Breathing Zone PAA in mg/m3 divided by Estimated Airborne PAA with Complete Vaporization in mg/m^3.

There is no significant difference in average Breathing Zone PAA between the three groups using either 1, 2, or 4 cloths per trial (p=0.641).

There is a significant difference in average Solution Mass in Chamber between the three groups using either 1, 2, or 4 cloths per trial (p<0.001).

There is a significant difference in average Estimated Breathing Zone PAA with Complete Vaporization between the three groups using either 1, 2, or 4 cloths per trial (p=<0.001).

There is a significant difference in average Breathing Zone PAA Fraction between the three groups using either 1, 2, or 4 cloths per trial (p=0.010).

PAA Exposure Concentrations

The PPA exposure concentrations were both measured and modeled using data from emission studies (see below) and standard industrial hygiene modeling techniques. The modeling results are discussed below.

Measured PAA Exposure Concentrations

As described above, PAA exposure studies were conducted in a controlled environment with a known volume and air flow rate. One, two, and four rags were soaked in a 4% OxCide™ (5.6% PAA) solution and used to clean surfaces over a 20-minute period. The mass lost over the 20 minutes was measured as well as the averaged PAA concentration in the breathing zone. Each experiment was conducted four times.

Modeled PAA Exposure Concentrations

The following procedure was used to model the average PAA concentrations for each of the twelve exposure studies. The average PAA emission rate was derived from the total mass lost over the 20-minute interval and the fraction of PAA in the solution (0.13%).

The average concentration in the chamber for each test was calculated using the following approach.[2]

1. For a constant emission rate, the vapor concentration, C, in the chamber at any time, t, during the application is:

$$C = (E/Q) * (1-\exp(-ACM*t)$$

Where, Q=V*ACM

   a. C = Vapor concentration in the breathing zone, $\mu g/m^3$.
   b. E = Emission rate of PAA, g/min
   c. Q = Air flow rate through the area, m^3/min
   d. V = Volume of test chamber
   e. ACM = Air exchange rate of the test chamber, 1/min = ACH/60

[2] American Industrial Hygiene Association (AIHA). Mathematical Models for Estimating Occupational Exposure to Chemicals, 2nd Edition. Eds.: Keil, C. B., Simmons, C. E., Anthony, T.R. AIHA, 2009.

2. Integrating the above equation over time T yields the modeled average chamber concentration, Cave:

$$C = (E^*/Q) * [1 + (1/(ACM*T) * (\exp(-ACM*t)-1]$$

Table 5-7 shows the calculated Cave values.

Table 5-7. Calculated Average Concentration in Room from Mass Lost

# cloths	E (g/min)[1]	Q (m^3/min)	ACM (1/min)	T (min)	Cave (µg/m^3)	Airborne PAA Fraction (%)[2]
1	0.007284	2.06	0.09	20	1896	9%
1	0.006864	2.06	0.09	20	1787	17%
1	0.007022	2.06	0.09	20	1828	20%
1	0.007655	2.06	0.09	20	1993	13%
2	0.01085	2.06	0.09	20	2825	9%
2	0.0112	2.06	0.09	20	2916	9%
2	0.013038	2.06	0.09	20	3394	9%
2	0.014088	2.06	0.09	20	3667	9%
4	0.018988	2.06	0.09	20	4943	7%
4	0.0168	2.06	0.09	20	4374	6%
4	0.016713	2.06	0.09	20	4351	7%
4	0.016188	2.06	0.09	20	4214	7%
[1] E is calculated by dividing the total PAA mass (g) by 20 min						
[2] This is calculated by dividing the breathing zone PAA (µg/m^3) by Cave (µg/m^3)						

Testing Protocol for Exposure Model Calibration of Monell Chamber

Scope: The first goal is to determine the magnitude of change in airborne PAA, AA, and HP concentrations in the Monell chamber using the standard protocol but increasing the OxyCide™ mixing rate to 4 ounces of concentrate per gallon. The second goal is to estimate surface areas for each object sanitized and measure the mass of solution transferred to the sanitized surface area under three use scenarios: 1 cloth used for 20 minutes, 2 cloths used for 10 minutes each, and 4 cloths used for 5 minutes each.

Procedure:

1. Two solutions with an OxyCide™ content of 4 ounces of concentrate per gallon will be mixed within 1 hour prior to start of cloth usage: 1) For Trials 1 through 8 in the morning sessions, an OxyCide™ concentrate mass of 51.25 grams will be added to 1455 ml deionized water and used to evenly wet 12 cloths; 2) For Trials 9 through 12 in the afternoon sessions, an OxyCide™ concentrate mass of 68.33 grams will be added to 1940 ml of deionized water and used to wet 16 cloths.
2. Prior to start of the trials, Brent Kerger will take measurements of the surface area of sanitation for all surfaces to be wiped in series during this testing.
3. For each trial, a top-loader balance will be used to weigh each wetted cloth before and after it has been used by Brent Kerger in sanitizing the designated surfaces within the Monell chamber. The cloth weights for each trial will be recorded in a log for that day. All chamber operating conditions will be identical to the standard protocol and a minimum of 10 minutes will be allowed between trials for clearing prior trial residual vapors. The sequence of trials will be from the lowest to highest number of wetted cloths used per trial: 1) one cloth per 20-minute trial; 2) two cloths for 10 minutes each per trial; and 3) four cloths for 5 minutes each per trial. Four trials of each condition will be completed.
4. For each trial, Brent Kerger will record the number of times that the full sequence of surface cleaning was completed and the stopping point in the sequence for each cloth used; this information will be used to estimate the total surface area wiped per cloth in each trial.
5. With assistance from Monell staff for sampling per the standard protocol, Brent Kerger will wear the sampling vest for collecting the air samples while sanitizing surfaces during each trial in accordance with the standard Monell protocol, except that no subjective ratings or biological samples will be collected; video recordings will be taken only for the first trial of each set. This protocol is designed solely for collecting quantitative information on surface area treated and mass of the solution transferred into the Monell chamber, which can be combined with the air concentration data to estimate PAA, AA, and HP emission rates under varied use conditions of the OxyCide™ wetted cloths.
6. Air sampling tubes for each trial will be capped and placed in pre-labeled bags by Monell staff for each of the three test conditions per the standard protocol. The samples will be stored refrigerated under the standard protocol and shipped to EAS within 24-hours after completion of this testing protocol.
7. EAS will extract and analyze each sample for PAA, AA, and HP in accordance with the standard protocol.

Laboratory Mass-Loss Studies Use of the OxyCide™Product

The study authors developed a testing protocol that was carried out by Environmental Analytical Services (EAS) laboratory to provide quantitative information on mass emission rates. The goal of this study was to determine the mass emission rate over one hour for undisturbed OxyCide™ solution and an equivalent volume of solution adhered to a microfiber cloth.

The procedure for conducting these studies is outlined and the results obtained from the EAS testing are presented in below. Mass emission rates of a 4 oz/gallon OxyCide™ solution were studied for both 125 mL of the solution and microfiber cloths wetted with 125 mL of the solution. A total of six 5 x 8-inch plastic trays (2 inches in depth) were set up so that there were 3 trays of 125 mL of the OxyCide™ solution only and 3 trays with microfiber cloths wetted with 125 mL of the OxyCide™ solution. The study protocol can be found at the end of this section of the Appendix.

1. The OxyCide™ solution was prepared by the following steps: A fresh bottle of OxyCide™ (provided by Ecolab) was opened and 27.83 g of the concentrate were mixed with 787.92 g deionized water in a 1 liter bottle. This was done within 1 hour prior to the start of testing. This corresponded to a 4 oz/gallon mixture. The Ecolab-provided bucket (including the sieve) for wetting the microfiber cloths was set up and three 125 mL portions of the OxyCide™ solution was poured in to wet 3 cloths.

2. The studies were completed in a laboratory hood at EAS (interior size of 3 feet wide and 2 feet deep) with a fixed sash height (open 14 inches). Air velocity measurements were obtained before and after the mass loss studies were performed in order to allow for estimation of the air exchange rate through the hood. The temperature was also recorded before and after each set of trials. A Cole-Parmer PBL 2002 top loader scale with a maximum range of 2000 g and a sensitivity of 0.01 g was used for the mass measurements over time.

3. Six labeled shallow plastic pans of fixed dimensions (5 x 8 inches and 2 inches in depth) were used for the mass loss studies. The initial weight of the six trays was recorded. Three trays were then filled with 125 mL OxyCide™ solution and the other three trays had a microfiber cloth wetted with 125 mL of the OxyCide™ solution. All 6 trays were then placed in the fume hood. The weights of each of the trays was recorded at T = 0, and then at 5-minute intervals until T = 60 min. Trays were arranged in the hood in two columns of 3:

	Back of hood	
Tray A		Tray E
Tray B		Tray F
Tray D		Tray C
	Front of hood	

4. The above series of experiments was completed with only deionized water in the open liquid and cloth-adsorbed conditions as a control comparison.

Table 5-8. Results from Deionized Water Trials

Air Flow Rate (m/s)					Temp (°C)					
Position	T_o	T_{30}	T_{60}		T_o	T_{15}	T_{30}	T_{45}	T_{60}	
POS 1	0.83	0.89	1.06		19.6	19.9	19.7	19.6	19.7	
POS 2	0.95	0.97	1.05							
POS 3	1.06	1.04	1.08		Mass of Dry Rags (g)					
POS 4	1.03	1.05	1.13		RAG D	52.17				
POS 5	1.23	1.31	1.36		RAG E	45.42				
POS 6	1.05	1.21	1.26		RAG F	45.38				
Mass (g) over time (min)										
	Dry	T_o	T_5	T_{10}	T_{15}	T_{20}	T_{30}	T_{40}	T_{50}	T_{60}
Tray A	67.91	191.51	190.97	190.56	190.22	189.75	188.97	188.23	187.58	187.01
Tray B	67.96	191.26	190.69	190.29	189.93	189.51	188.80	188.09	187.45	186.81
Tray C	68.10	191.36	190.68	190.23	189.81	189.40	188.60	187.80	187.09	186.29
Tray D	120.26	242.75	242.21	241.75	241.33	240.87	240.01	239.18	238.45	237.72
Tray E	112.90	241.40	240.88	240.40	240.01	239.58	238.85	238.12	237.46	236.63
Tray F	113.36	244.43	243.81	243.34	242.94	242.51	241.67	240.93	240.21	239.45

Table 5-9. Results from OxyCide™ Use Dilution Solution Trial

Air Flow Rate (m/s)					Temp (°C)					
Position	T_o	T_{30}	T_{60}		T_o	T_{15}	T_{30}	T_{45}	T_{60}	
POS 1	0.80	0.95	0.89		20.50	20.50	20.70	20.40	20.60	
POS 2	1.09	0.97	0.97							
POS 3	1.26	1.05	1.16		Mass of Dry Rags (g)					
POS 4	1.10	1.09	1.08		RAG D	51.79				
POS 5	1.28	1.23	1.26		RAG E	48.53				
POS 6	1.21	1.16	1.24		RAG F	52.25				
Mass (g) over time (min)										
	Dry	T_o	T_5	T_{10}	T_{15}	T_{20}	T_{30}	T_{40}	T_{50}	T_{60}
Tray A	67.94	190.59	190.06	189.63	189.25	188.88	188.21	187.57	186.87	186.29
Tray B	68.01	190.54	189.99	189.56	189.20	188.82	188.18	187.55	186.89	186.31
Tray C	68.12	191.88	191.25	190.69	190.30	189.86	189.07	188.30	187.57	186.86
Tray D	119.94	242.97	242.30	241.86	241.43	241.02	240.18	239.43	238.67	237.95
Tray E	116.10	242.19	241.34	240.77	240.30	239.83	238.93	238.04	237.25	236.45
Tray F	120.30	242.95	242.24	241.74	241.35	240.94	240.15	239.35	238.65	237.97

EAS Study of PAA Emission Rates from Saturated Rags not in Use

The following procedure was used to calculate the PAA emission rate from the EAS mass loss versus time study. Six trays containing microfiber cloths saturated with a 4 oz/gal OxyCide™ solution were placed in a well-ventilated area (fume hood). The trays were weighed over time to determine the mass of material lost per unit time. There was no significant difference between the emission rate slopes calculate for the 3 trays of OxyCide™ solution adhered to microfiber cloths when compared to the 3 trays of deionized water adhered to microfiber cloths, so the data

were combined. The emission rate was calculated by averaging the slopes of the lines of the mass loss versus time for each of the 6 trays. As shown below in Figures 1 and 2 and in the attached spreadsheet the results were all linear with an average emission rate of 79 mg/min for the solution and the deionized water. Based on a 2.8% (4 oz/gal dilution) OxyCide™ solution containing 5.8% PAA, this results in a PAA emission rate of 0.14 mg/min.

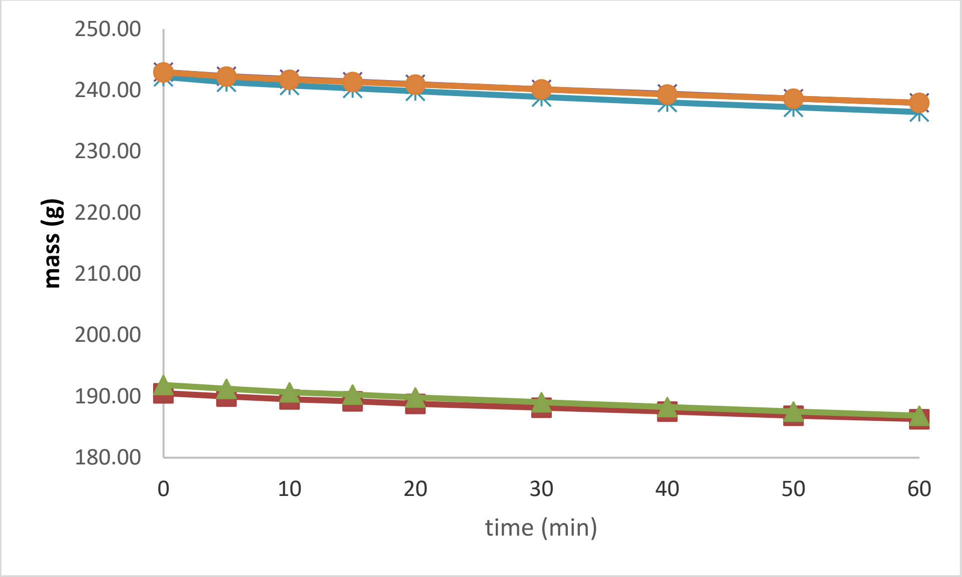

Figure 5-1. Mass loss over time from the six OxyCide™ trials in the EAS study

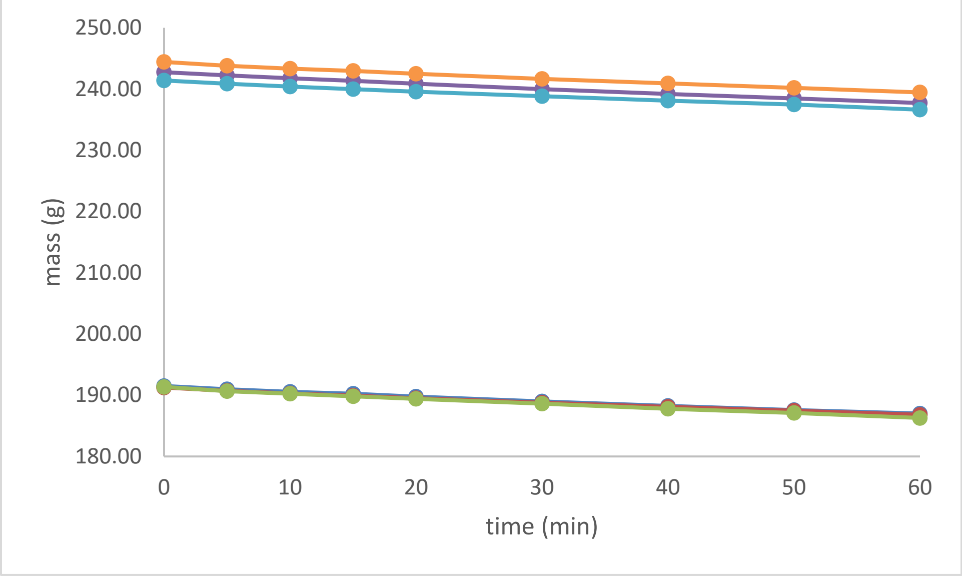

Figure 5-2. Mass loss over time from the six deionized water trials in the EAS study

Testing Protocol for OxyCide™ Mass Emission Rate from Liquid and Wetted Cloth

Scope: The goal is to determine the mass emission rate over one hour for undisturbed OxyCide™ solution and an equivalent volume of solution adhered to a microfiber cloth.

Procedure:

1. 800 ml of OxyCide use solution at 4 ounces of concentrate per gallon will be mixed within 1 hour prior to start of testing. The studies will be completed in a laboratory hood with a fixed sash height with air velocity measurements obtained before and after the mass loss studies are performed in order to allow for estimation of air exchange rate through the hood. A top-loader electronic balance will be placed inside the hood for measuring mass loss over time.
2. Six labeled shallow plastic pans of fixed dimensions (approximately 4 x 8 inches) will be used for the mass loss studies. Three pans will contain 125 ml of OxyCide use solution in the open liquid form and three other pans will contain 125 ml of OxyCide use solution adsorbed to a microfiber cloth that is folded to fit into the pan with the same top surface area as for the liquid solution.
3. Mass measurements for each pan will be recorded at time zero, 5, 10, 15, 20, 30, 40, 50 and 60 minutes. The temperature will be recorded before and after each set of trials.
4. The above series of experiments will be completed with only deionized water in the open liquid and cloth-adsorbed conditions as a control comparison.
