# Supplementary material for: Environmental chamber studies of eye and respiratory irritation from use of a peracetic acid–based hospital surface disinfectant
Source: Antimicrob Steward Healthc Epidemiol. 2023 Apr 11;3(1):e71. doi: 10.1017/ash.2023.138 (PMC10127244; doi:10.1017/ash.2023.138)
Supplement: Supplementary file 1 [file ashsup.zip › S2732494X23001389sup003.pdf]

Supplemental Appendix 2: Product Training Materials

Ecolab Training Materials for Use of the OxyCide™Product

The Ecolab training materials for OxyCide^{TM} use in hospital surface disinfection were reviewed and interviews were conducted to clarify their procedures in field training of environmental service staff. Selected written training documents are provided in this Appendix. Key considerations in the training include:

1. Assuring that the dispensed solution bottles contain usual amounts of visible bubbles indicating the presence of active ingredients PAA and HP needed for antimicrobial efficacy.
2. The EPA label-designated effective sporicidal concentration of OxyCide™ is 3 ounces per gallon of cold water.
3. Assuring that proper volume of dispensed solution is used to wet the cloths to a point of saturation but not dripping (the site-specific number of wetted cloths per bottle is listed on the Ecolab wall chart), and that the solution or cloths are used within an 8-hour shift to assure antimicrobial efficacy.
4. Assuring that the wetted cloths are used only for keeping target surfaces continually wetted for 5 minutes (as recommended by the OxyCide™ label for optimal sporicidal activity), and typically using 1 to 2 wetted cloths each for the patient room and bathroom spaces. The Ecolab wall chart states that bottles of liquid and buckets of wetted cloths should be kept sealed/covered, and the saturated cloths should not be wrung out or compressed causing drippage, as these activities can remove PAA and HP from solution and reduce antimicrobial efficacy of the use solution.
5. The Center for Disease Control (CDC) patient room cleaning guidelines indicate to proceed cleaning from cleaner areas to dirtier areas (https://www.cdc.gov/hai/prevent/resource-limited/cleaning-procedures.html). Terminal cleaning should start with shared equipment, then proceed to surfaces and items touched during patient care that are outside of the patient zone, and finally to surfaces and items directly touched by the patient. The CDC advises to clean from high surfaces to lower surfaces to prevent dirt and microorganisms from falling and contaminating already cleaned areas. In addition, cleaning should be performed in a systematic manner (i.e., left to right or clockwise). Ecolab's training and recommended cleaning steps follow the CDC guidance.
6. Aside from the wetted microfiber cloth application by hand for typical surface disinfection uses, Ecolab also sold microfiber cloths with elastic edges to act as mophead covers for floor and/or nonporous wall surfaces (e.g., tiled walls). These wetted mophead covers were designated for surface disinfectant use in unique spaces like operating rooms that typically have much higher air exchange rates compared to patient rooms or bathrooms. Based on field studies conducted in 2013 by Ecolab researchers (Nordling et al., 2015) showing higher airborne PAA concentrations with floor mopping use in patient

rooms and bathrooms, Ecolab training discontinued floor mopping use of OxyCide™ in patient rooms and bathrooms.

Interviews of Ecolab training supervisors and field staff were used to assess a reasonable upper bound use pattern for patient room/bathroom discharge cleaning by environmental services staff. It was agreed that in general the total room cleaning time target was between 30 and 40 minutes; that typical duration of wetted cloth handling was up to 10 minutes in the patient room space and up to 10 minutes in the patient bathroom space; and that typically 1 to 2 wetted cloths were used in each space. Based on these estimates, it was decided that an upper bound value for continuous handling of the wetted cloths would be 20 minutes per room, with a total of 4 cloths utilized (i.e., changing to a fresh wetted cloth every 5 minutes over a 20-minute period of continuous cleaning).

As part their product stewardship program, Ecolab tracked and monitored complaints of adverse health effects regarding the OxyCide™ product via two systems: 1) an internal monitoring system called CATSWeb, which is a software system that was used for tracking complaints about the product, including those of adverse health effects, and 2) an external monitoring system called SafetyCall, which is a third party who employs medical and health professionals who review and categorize complaints and then send them to the USEPA. Data from both monitoring services demonstrates that complaints of respiratory irritation decreased from 2013 to 2020. The figures at the end of this supplemental appendix summarize the complaints of respiratory irritation regarding OxyCide™ recorded in the CATSWeb system from 2013 through 2020 and the complaints of respiratory irritation regarding OxyCide™ recorded by SafetyCall from 2013 through 2021.

Materials included in this Appendix include the following:

--Ecolab Safety Data Sheet (SDS) for OxyCide™

--USEPA Label for OxyCide™

--Ecolab Wall Chart

--CDC Guidelines for Environmental Cleaning

--CDC Examples of High-Touch Surfaces

--Ecolab OxyCide™ Product Specification Sheet

--Ecolab Training Presentation: EVS Staff In-Service Training

-- CATSWeb and SafetyCall data

SAFETY DATA SHEET

OXYCIDE DAILY DISINFECTANT CLEANER

SECTION 1. PRODUCT AND COMPANY IDENTIFICATION

Product name	:	OXYCIDE DAILY DISINFECTANT CLEANER
Other means of identification	:	Not applicable
Recommended use	:	Disinfectant
Restrictions on use	:	Reserved for industrial and professional use.
Product dilution information	:	2.34 %
Company	:	Ecolab Inc.<br>1 Ecolab Place<br>St. Paul, Minnesota USA 55102<br>1-800-352-5326
Emergency health information	:	1-800-328-0026 (US/Canada), 1-651-222-5352 (outside US)
Issuing date	:	05/07/2019

SECTION 2. HAZARDS IDENTIFICATION

GHS Classification

Product AS SOLD

Oxidizing liquids	:	Category 2
Organic peroxides	:	Type F
Acute toxicity (Oral)	:	Category 4
Acute toxicity	:	Category 3
Skin corrosion	:	Category 1A
Serious eye damage	:	Category 1

Product AT USE DILUTION

Acute toxicity (Oral) : Category 4

GHS label elements

Product AS SOLD

Hazard pictograms :

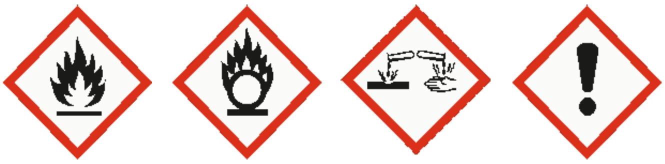

Signal Word : Danger

Hazard Statements : Heating may cause a fire.
May intensify fire; oxidizer.
Harmful if swallowed.
Causes severe skin burns and eye damage.
Toxic if inhaled.

Precautionary Statements : **Prevention:**
Keep away from heat/sparks/open flames/hot surfaces. No smoking. Keep/Store away from clothing/ combustible materials. Take any precaution to avoid mixing with combustibles. Keep only in original container. Keep cool. Avoid breathing dust/ fume/ gas/ mist/ vapors/

spray. Wash skin thoroughly after handling. Do not eat, drink or smoke when using this product. Use only outdoors or in a well-ventilated area. Wear protective gloves/ protective clothing/ eye protection/ face protection.
Response:
IF SWALLOWED: Call a POISON CENTER/doctor if you feel unwell. Rinse mouth. IF SWALLOWED: Rinse mouth. Do NOT induce vomiting. IF ON SKIN (or hair): Take off immediately all contaminated clothing. Rinse skin with water/shower. IF INHALED: Remove person to fresh air and keep comfortable for breathing. Immediately call a POISON CENTER/doctor. IF IN EYES: Rinse cautiously with water for several minutes. Remove contact lenses, if present and easy to do. Continue rinsing. Immediately call a POISON CENTER/doctor. Wash contaminated clothing before reuse. In case of fire: Use dry sand, dry chemical or alcohol-resistant foam to extinguish.
Storage:
Store in a well-ventilated place. Keep container tightly closed. Store locked up. Protect from sunlight. Store away from other materials.
Disposal:
Dispose of contents/ container to an approved waste disposal plant.

Product AT USE DILUTION

Hazard pictograms :

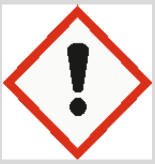

Hazard Statements : Harmful if swallowed.

Precautionary Statements : **Prevention:**
Wash skin thoroughly after handling. Do not eat, drink or smoke when using this product.
Response:
IF SWALLOWED: Call a POISON CENTER/doctor if you feel unwell. Rinse mouth.
Disposal:
Dispose of contents/ container to an approved waste disposal plant.

Product AS SOLD

Other hazards : Do not mix with bleach or other chlorinated products – will cause chlorine gas.

SECTION 3. COMPOSITION/INFORMATION ON INGREDIENTS

Product AS SOLD

Pure substance/mixture : Mixture

**Chemical name**	**CAS-No.**	**Concentration (%)**
Hydrogen peroxide	7722-84-1	27.5
Acetic acid	64-19-7	5 - 10
Peroxyacetic acid, type F, stabilized	79-21-0	5.8

Product AT USE DILUTION

**Chemical name**	**CAS-No.**	**Concentration (%)**
Hydrogen peroxide	7722-84-1	0.64
Peroxyacetic acid	79-21-0	0.14

SECTION 4. FIRST AID MEASURES

Product AS SOLD

In case of eye contact	:	Rinse immediately with plenty of water, also under the eyelids, for at least 15 minutes. Remove contact lenses, if present and easy to do. Continue rinsing. Get medical attention immediately.
In case of skin contact	:	Wash off immediately with plenty of water for at least 15 minutes. Use a mild soap if available. Wash clothing before reuse. Thoroughly clean shoes before reuse. Get medical attention immediately.
If swallowed	:	Rinse mouth with water. Do NOT induce vomiting. Never give anything by mouth to an unconscious person. Get medical attention immediately.
If inhaled	:	Remove to fresh air. Treat symptomatically. Get medical attention immediately.
Protection of first-aiders	:	If potential for exposure exists refer to Section 8 for specific personal protective equipment.
Notes to physician	:	Treat symptomatically.
Most important symptoms and effects, both acute and delayed	:	See Section 11 for more detailed information on health effects and symptoms.

Product AT USE DILUTION

In case of eye contact	:	Rinse with plenty of water.
In case of skin contact	:	Rinse with plenty of water.
If swallowed	:	Rinse mouth. Get medical attention if symptoms occur.
If inhaled	:	Get medical attention if symptoms occur.

SECTION 5. FIRE-FIGHTING MEASURES

Product AS SOLD

Suitable extinguishing media	:	Use extinguishing measures that are appropriate to local circumstances and the surrounding environment.
Unsuitable extinguishing media	:	None known.
Specific hazards during fire fighting	:	Special protective equipment for fire-fighters<br>Oxidizer. Contact with other material may cause fire.<br>Oxidizer; material is an oxidizer which may readily react with other materials, especially upon heating.
Hazardous combustion products	:	Decomposition products may include the following materials:<br>Carbon oxides<br>Oxides of phosphorus
Special protective equipment for fire-fighters	:	In case of fire, wear a full face positive-pressure self contained breathing apparatus and protective suit.
Specific extinguishing	:	Fire residues and contaminated fire extinguishing water must be

methods		disposed of in accordance with local regulations. In the event of fire and/or explosion do not breathe fumes.

SECTION 6. ACCIDENTAL RELEASE MEASURES

Product AS SOLD

Personal precautions, protective equipment and emergency procedures	:	Ensure adequate ventilation. Keep people away from and upwind of spill/leak. Avoid inhalation, ingestion and contact with skin and eyes. When workers are facing concentrations above the exposure limit they must use appropriate certified respirators. Ensure clean-up is conducted by trained personnel only. Refer to protective measures listed in sections 7 and 8.
Environmental precautions	:	Do not allow contact with soil, surface or ground water.
Methods and materials for containment and cleaning up	:	Stop leak if safe to do so. Isolate the waste do not allow it to come into contact with incompatible materials. For small spills contain with sand or vermiculite and dilute the contained product at least 10 times with water. Transfer to an open topped container and remove to a safe place for neutralization* / disposal. For large spills contain spill and evacuate the area, leave until the reaction subsides, then collect up for disposal. Obtain consent from the local water company / authority if considering discharge to sewer. *NEUTRALIZATION : once diluted, neutralize with a suitable alkali such as sodium bicarbonate. Combustible materials exposed to this product should be rinsed immediately with large amounts of water to ensure that all product is removed. Residual product which is allowed to dry on organic materials such as rags, cloths, paper, fabrics, cotton, leather, wood, or other combustibles may spontaneously ignite and result in a fire.

Product AT USE DILUTION

Personal precautions, protective equipment and emergency procedures	:	Ensure clean-up is conducted by trained personnel only. Refer to protective measures listed in sections 7 and 8.
Environmental precautions	:	Do not allow contact with soil, surface or ground water.
Methods and materials for containment and cleaning up	:	Stop leak if safe to do so. Contain spillage, and then collect with non-combustible absorbent material, (e.g. sand, earth, diatomaceous earth, vermiculite) and place in container for disposal according to local / national regulations (see section 13). For large spills, dike spilled material or otherwise contain material to ensure runoff does not reach a waterway.

SECTION 7. HANDLING AND STORAGE

Product AS SOLD

Advice on safe handling	:	Do not ingest. Do not get in eyes, on skin, or on clothing. Do not breathe dust/ fume/ gas/ mist/ vapors/ spray. Use only with adequate ventilation. Wash hands thoroughly after handling. Do not mix with bleach or other chlorinated products – will cause chlorine gas.
Conditions for safe storage	:	Do not store on wooden pallets. Keep in a cool, well-ventilated place. Keep away from reducing agents. Keep away from strong bases. Keep away from combustible material. Keep out of reach of children. Keep container tightly closed. Store in suitable labeled containers. Pressure bursts may occur due to gas evolution if the container is not adequately vented.

Storage temperature : -10 °C to 40 °C

Product AT USE DILUTION

Advice on safe handling : Do not ingest. Wash hands thoroughly after handling.

Conditions for safe storage : Keep out of reach of children. Store in suitable labeled containers.

SECTION 8. EXPOSURE CONTROLS/PERSONAL PROTECTION

Product AS SOLD
Ingredients with workplace control parameters

Components	CAS-No.	Form of exposure	Permissible concentration	Basis
Hydrogen peroxide	7722-84-1	TWA	1 ppm	ACGIH
		TWA	1 ppm<br>1.4 mg/m3	NIOSH REL
		TWA	1 ppm<br>1.4 mg/m3	OSHA Z1
Acetic acid	64-19-7	TWA	10 ppm	ACGIH
		STEL	15 ppm	ACGIH
		STEL	15 ppm<br>37 mg/m3	NIOSH REL
		TWA	10 ppm<br>25 mg/m3	NIOSH REL
		TWA	10 ppm<br>25 mg/m3	OSHA Z1
Peroxyacetic acid	79-21-0	STEL	0.4 ppm	ACGIH

Engineering measures : Effective exhaust ventilation system. Maintain air concentrations below occupational exposure standards.

Personal protective equipment

Eye protection : Wear eye protection/ face protection.

Hand protection : Wear the following personal protective equipment:
Standard glove type.
Gloves should be discarded and replaced if there is any indication of degradation or chemical breakthrough.

Skin protection : Personal protective equipment comprising: suitable protective gloves, safety goggles and protective clothing

Respiratory protection : When workers are facing concentrations above the exposure limit they must use appropriate certified respirators.

Hygiene measures : Handle in accordance with good industrial hygiene and safety practice. Remove and wash contaminated clothing before re-use. Wash face, hands and any exposed skin thoroughly after handling. Provide suitable facilities for quick drenching or flushing of the eyes and body in case of contact or splash hazard.

Product AT USE DILUTION

Engineering measures : Good general ventilation should be sufficient to control worker exposure to airborne contaminants.

Personal protective equipment

Eye protection	:	No special protective equipment required.
Hand protection	:	No special protective equipment required.
Skin protection	:	No special protective equipment required.
Respiratory protection	:	No personal respiratory protective equipment normally required.

SECTION 9. PHYSICAL AND CHEMICAL PROPERTIES

		Product AS SOLD	Product AT USE DILUTION
Appearance	:	liquid	liquid
Color	:	colorless	colorless
Odor	:	pungent	vinegar-like
pH	:	1.0, (100 %)	2.7 - 4.0
Flash point	:	Not applicable	
Odor Threshold	:	No data available	
Melting point/freezing point	:	No data available	
Initial boiling point and boiling range	:	No data available	
Evaporation rate	:	No data available	
Flammability (solid, gas)	:	No data available	
Upper explosion limit	:	No data available	
Lower explosion limit	:	No data available	
Vapor pressure	:	No data available	
Relative vapor density	:	No data available	
Relative density	:	1.1 - 1.14	
Water solubility	:	No data available	
Solubility in other solvents	:	No data available	
Partition coefficient: n-octanol/water	:	No data available	
Autoignition temperature	:	No data available	
Thermal decomposition	:	No data available	
Viscosity, kinematic	:	No data available	
Explosive properties	:	No data available	
Oxidizing properties	:	No data available	
Molecular weight	:	No data available	
VOC	:	No data available	

SECTION 10. STABILITY AND REACTIVITY

Product AS SOLD

Reactivity	:	No dangerous reaction known under conditions of normal use.
Chemical stability	:	pressure build-up

		Contamination may result in dangerous pressure increases - closed containers may rupture.
Possibility of hazardous reactions	:	Do not mix with bleach or other chlorinated products – will cause chlorine gas.
Conditions to avoid	:	Direct sources of heat.<br>Exposure to sunlight.
Incompatible materials	:	Bases<br>Metals<br>Organic materials
Hazardous decomposition products	:	Decomposition products may include the following materials:<br>Carbon oxides<br>Oxides of phosphorus

SECTION 11. TOXICOLOGICAL INFORMATION

Information on likely routes of exposure	:	Inhalation, Eye contact, Skin contact

Potential Health Effects

Product AS SOLD

Eyes	:	Causes serious eye damage.
Skin	:	Causes severe skin burns.
Ingestion	:	Harmful if swallowed. Causes digestive tract burns.
Inhalation	:	Toxic if inhaled. May cause respiratory tract irritation.
Chronic Exposure	:	Health injuries are not known or expected under normal use.

Product AT USE DILUTION

Eyes	:	Health injuries are not known or expected under normal use.
Skin	:	Health injuries are not known or expected under normal use.
Ingestion	:	Harmful if swallowed.
Inhalation	:	Health injuries are not known or expected under normal use.
Chronic Exposure	:	Health injuries are not known or expected under normal use.

Experience with human exposure

Product AS SOLD

Eye contact	:	Redness, Pain, Corrosion
Skin contact	:	Redness, Pain, Corrosion
Ingestion	:	Corrosion, Abdominal pain
Inhalation	:	Respiratory irritation, Cough

Product AT USE DILUTION

Eye contact	:	No symptoms known or expected.

Skin contact	:	No symptoms known or expected.
Ingestion	:	Corrosion, Abdominal pain
Inhalation	:	No symptoms known or expected.

Toxicity

Product AS SOLD
Product

Acute oral toxicity	:	Acute toxicity estimate : 1,599 mg/kg
Acute inhalation toxicity	:	4 h Acute toxicity estimate : 33.03 mg/l<br>Test atmosphere: vapor
Acute dermal toxicity	:	Acute toxicity estimate : > 5,000 mg/kg
Skin corrosion/irritation	:	No data available
Serious eye damage/eye irritation	:	No data available
Respiratory or skin sensitization	:	No data available
Carcinogenicity	:	No data available
Reproductive effects	:	No data available
Germ cell mutagenicity	:	No data available
Teratogenicity	:	No data available
STOT-single exposure	:	No data available
STOT-repeated exposure	:	No data available
Aspiration toxicity	:	No data available

SECTION 12. ECOLOGICAL INFORMATION

Product AS SOLD
Ecotoxicity

Environmental Effects	:	Harmful to aquatic life.

Product

Toxicity to fish	:	96 h LC50: 17.8 mg/l
Toxicity to daphnia and other aquatic invertebrates	:	96 h LC50: 21.2 mg/l
Toxicity to algae	:	No data available
Toxicity to daphnia and other aquatic invertebrates (Chronic toxicity)	:	96 h NOEC: 15 mg/l

Components

Toxicity to algae	:	Hydrogen peroxide<br>72 h EC50: 1.38 mg/l<br><br>Acetic acid<br>72 h EC50 Skeletonema costatum (marine diatom): > 1,000 mg/l

Peroxyacetic acid, type F, stabilized
72 h EC50: 0.7 mg/l

Persistence and degradability

Product AS SOLD
Readily biodegradable.

Product AT USE DILUTION
Not applicable - Biocide

Bioaccumulative potential

No data available

Mobility in soil

No data available

Other adverse effects

No data available

SECTION 13. DISPOSAL CONSIDERATIONS

Product AS SOLD

Disposal methods	:	Do not contaminate ponds, waterways or ditches with chemical or used container. Where possible recycling is preferred to disposal or incineration. If recycling is not practicable, dispose of in compliance with local regulations. Dispose of wastes in an approved waste disposal facility.
Disposal considerations	:	Dispose of as unused product. Empty containers should be taken to an approved waste handling site for recycling or disposal. Do not re-use empty containers. Dispose of in accordance with local, state, and federal regulations.
RCRA - Resource Conservation and Recovery Authorization Act Hazardous waste	:	D002 (Corrosive)<br>D001 (Ignitable)

Product AT USE DILUTION

Disposal methods	:	Do not contaminate ponds, waterways or ditches with chemical or used container. Where possible recycling is preferred to disposal or incineration. If recycling is not practicable, dispose of in compliance with local regulations. Dispose of wastes in an approved waste disposal facility.
Disposal considerations	:	Dispose of as unused product. Empty containers should be taken to an approved waste handling site for recycling or disposal. Dispose of in accordance with local, state, and federal regulations.<br>Dispose of as unused product. Empty containers should be taken to an approved waste handling site for recycling or disposal. Do not re-use empty containers. Dispose of in accordance with local, state, and federal regulations.

SECTION 14. TRANSPORT INFORMATION

Product AS SOLD

OXYCIDE DAILY DISINFECTANT CLEANER

The shipper/consignor/sender is responsible to ensure that the packaging, labeling, and markings are in compliance with the selected mode of transport.

Land transport (DOT)

UN number : 3109
Description of the goods : ORGANIC PEROXIDE TYPE F, LIQUID (Peroxyacetic acid, type F, stabilized)
Class : 5.2 (8)
Environmentally hazardous : no

Sea transport (IMDG/IMO)

UN number : 3109
Description of the goods : ORGANIC PEROXIDE TYPE F, LIQUID (Peroxyacetic acid, type F, stabilized)
Class : 5.2 (8)
Marine pollutant : no

SECTION 15. REGULATORY INFORMATION

Product AS SOLD

EPA Registration number : 1677-237

EPCRA - Emergency Planning and Community Right-to-Know

CERCLA Reportable Quantity

Components	CAS-No.	Component RQ (lbs)	Calculated product RQ (lbs)
Acetic acid	64-19-7	5000	62500

SARA 304 Extremely Hazardous Substances Reportable Quantity

Components	CAS-No.	Component RQ (lbs)	Calculated product RQ (lbs)
Peroxyacetic acid	79-21-0	500	8620

SARA 311/312 Hazards : Oxidizer (liquid, solid or gas)
Organic peroxides
Acute toxicity (any route of exposure)
Skin corrosion or irritation
Serious eye damage or eye irritation
Specific target organ toxicity (single or repeated exposure)

SARA 302 : The following components are subject to reporting levels established by SARA Title III, Section 302:

Hydrogen peroxide	7722-84-1	27.5 %
Peroxyacetic acid	79-21-0	5.8 %

SARA 313 : This material does not contain any chemical components with known CAS numbers that exceed the threshold (De Minimis) reporting levels established by SARA Title III, Section 313.

Peroxyacetic acid	79-21-0	5.8 %

California Prop. 65
This product does not contain any chemicals known to the State of California to cause cancer, birth, or any other reproductive defects.

California Cleaning Product Right to Know Act of 2017 (SB 258)

This regulation applies to this product.

Chemical Name	CAS-No.	Function	List(s)
water	7732-18-5	Diluent	Not Applicable
Hydrogen peroxide	7722-84-1	Brightener	Not Applicable
Acetic acid	64-19-7	Buffer	Not Applicable
Peroxyacetic acid, type F, stabilized	79-21-0	Brightener	Not Applicable
Chelating agent	Withheld	Stabilizer	Not Applicable

*refer to ecolab.com/sds for electronic links to designated lists

The ingredients of this product are reported in the following inventories:

United States TSCA Inventory :
On the inventory, or in compliance with the inventory

Canadian Domestic Substances List (DSL) :
All components of this product are on the Canadian DSL

Australia Inventory of Chemical Substances (AICS) :
On the inventory, or in compliance with the inventory

New Zealand. Inventory of Chemical Substances :
On the inventory, or in compliance with the inventory

Japan. ENCS - Existing and New Chemical Substances Inventory :
On the inventory, or in compliance with the inventory

Korea. Korean Existing Chemicals Inventory (KECI) :
On the inventory, or in compliance with the inventory

Philippines Inventory of Chemicals and Chemical Substances (PICCS) :
On the inventory, or in compliance with the inventory

China. Inventory of Existing Chemical Substances in China (IECSC) :
On the inventory, or in compliance with the inventory

Taiwan Chemical Substance Inventory (TCSI) :
On the inventory, or in compliance with the inventory

SECTION 16. OTHER INFORMATION

Product AS SOLD

SAFETY DATA SHEET

OXYCIDE DAILY DISINFECTANT CLEANER

NFPA:

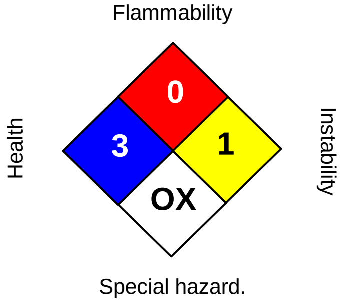

HMIS III:

HEALTH	3
FLAMMABILITY	0
PHYSICAL HAZARD	1

0 = not significant, 1 =Slight,
2 = Moderate, 3 = High
4 = Extreme, * = Chronic

Product AT USE DILUTION

NFPA:

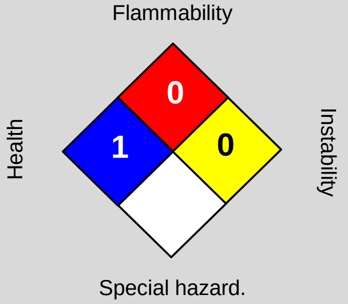

HMIS III:

HEALTH	1
FLAMMABILITY	0
PHYSICAL HAZARD	0

0 = not significant, 1 =Slight,
2 = Moderate, 3 = High
4 = Extreme, * = Chronic

Issuing date	:	05/07/2019
Version	:	2.6
Prepared by	:	Regulatory Affairs

REVISED INFORMATION: Significant changes to regulatory or health information for this revision is indicated by a bar in the left-hand margin of the SDS.

The information provided in this Material Safety Data Sheet is correct to the best of our knowledge, information and belief at the date of its publication. The information given is designed only as a guidance for safe handling, use, processing, storage, transportation, disposal and release and is not to be considered a warranty or quality specification. The information relates only to the specific material designated and may not be valid for such material used in combination with any other materials or in any process, unless specified in the text.

UNITED STATES ENVIRONMENTAL PROTECTION AGENCY
WASHINGTON, DC 20460

OFFICE OF CHEMICAL SAFETY
AND POLLUTION PREVENTION

November 23, 2020

Mandy Sunde
Senior Regulatory Specialist I
Ecolab Inc.
1 Ecolab Place
St. Paul, MN 55102

Subject: PRIA Label Amendment – Acceptable Addition to Add SARS-COV-2 Claims
Product Name: Oxycide Daily Disinfectant Cleaner
EPA Registration Number: 1677-237
Application Date: 08/18/2020
Decision Number: 565625

Dear Ms. Sunde:

The amended label referred to above, submitted in connection with registration under the Federal Insecticide, Fungicide and Rodenticide Act, as amended, is acceptable. This approval does not affect any conditions that were previously imposed on this registration. You continue to be subject to existing conditions on your registration and any deadlines connected with them.

A stamped copy of your labeling is enclosed for your records. This labeling supersedes all previously accepted labeling. You must submit one copy of the final printed labeling before you release the product for shipment with the new labeling. In accordance with 40 CFR 152.130(c), you may distribute or sell this product under the previously approved labeling for 18 months from the date of this letter. After 18 months, you may only distribute or sell this product if it bears this new revised labeling or subsequently approved labeling. "To distribute or sell" is defined under FIFRA section 2(gg) and its implementing regulation at 40 CFR 152.3.

Should you wish to add/retain a reference to the company's website on your label, then please be aware that the website becomes labeling under the Federal Insecticide Fungicide and Rodenticide Act and is subject to review by the Agency. If the website is false or misleading, the product would be misbranded and unlawful to sell or distribute under FIFRA section 12(a)(1)(E). 40 CFR 156.10(a)(5) list examples of statements EPA may consider false or misleading. In addition, regardless of whether a website is referenced on your product's label, claims made on the website may not substantially differ from those claims approved through the registration process. Therefore, should the Agency find or if it is brought to our attention that a website contains false or misleading statements or claims substantially differing from the EPA approved registration, the website will be referred to the EPA's Office of Enforcement and Compliance.

Your release for shipment of the product constitutes acceptance of these conditions. If these conditions are not complied with, the registration will be subject to cancellation in accordance

with FIFRA section 6. If you have any questions, please contact Alex Horansky by phone at 703-347-0128, or via email at Horansky.alex@epa.gov.

Sincerely,

John Hebert, Chief
Regulatory Management Branch 1
Antimicrobials Division (7510P)
Office of Pesticide Programs

Enclosure

OxyCide™ Daily Disinfectant Cleaner

ONE-STEP† DISINFECTANT WITH SPORICIDAL ACTIVITY/ CLEANER / VIRUCIDE* / DEODORIZER / KILLS GERMS* / KILLS *CLOSTRIDIUM DIFFICILE* ENDOSPORES

Healthcare Facilities, Academic Facilities, Dietary Areas, Office Buildings, Recreational Facilities, Retail and Wholesale Establishments, Institutional and Industrial Use

ACCEPTED
11/23/2020
Under the Federal Insecticide, Fungicide and Rodenticide Act as amended, for the pesticide registered under EPA Reg. No. 1677-237

(Contains no alkyl phenol ethoxylate detergents)

**Active Ingredients:**	
Hydrogen Peroxide	27.5%
Peroxyacetic Acid	5.8%
**Other Ingredients:**	66.7%
**Total:**	100.0%

KEEP OUT OF REACH OF CHILDREN

DANGER

PELIGRO

(See [back], [side], [inside], [other] [fold-out] [booklet] [hang tag] [product container] [label(s)] [panel(s)] [container] for [complete] [additional] [information] [directions for use] [precautionary statements]) [and] [storage and disposal] [container handling and disposal])

[**Note to Reviewer**: In accordance with 40 CFR 156.68(d), all first aid statements, as prescribed, will appear on the front panel of the product label.]

PRECAUTIONARY STATEMENTS

HAZARDS TO HUMANS AND DOMESTIC ANIMALS

DANGER: Corrosive. Causes irreversible eye damage and skin burns. May be fatal if inhaled. Harmful if swallowed. Do not get in eyes, on skin or on clothing. Do not breathe vapor or spray mist. Wash thoroughly with soap and water after handling and before eating, drinking, chewing gum, using tobacco, or using the toilet. Remove and wash contaminated clothing before reuse. Wear coveralls over long-sleeved shirt and long pants, socks and chemical-resistant footwear, goggles or face shield, chemical-resistant gloves (such as rubber or made out of any waterproof material), and chemical-resistant apron. Wear a minimum of a NIOSH-approved elastomeric half mask respirator with organic vapor (OV) cartridges and combination N1 , R, or P filters; OR a NIOSH-approved gas mask with OV canisters; OR a NIOSH-approved powered air purifying respirator with OV cartridges and combination HE filters.

FIRST AID

IF IN EYES: Hold eye open and rinse slowly and gently with water for 15-20 minutes. Remove contact lenses, if present, after the first 5 minutes, then continue rinsing eye. Call a poison control center or doctor for treatment advice.

IF ON SKIN OR CLOTHING: Take off contaminated clothing. Rinse skin immediately with plenty of water for 15 –20 minutes. Call a poison control center or doctor for treatment advice.

IF INHALED: Move person to fresh air. If person is not breathing, call 911 or an ambulance and then give artificial respiration, preferably mouth-to-mouth, if possible. Call a poison control center or doctor for further treatment advice.

IF SWALLOWED: Call a poison control center or doctor immediately for treatment advice. Have person sip a glass of water if able to swallow. Do not induce vomiting unless told to do so by the poison control center or doctor. Do not give anything by mouth to an unconscious person.

[**Note to Reviewer**: In accordance with 40 CFR 156.68(d), all first aid statements, as prescribed, will appear on the front panel of the product label.]

Have the product container or label with you when calling a poison control center or going for treatment.

FOR EMERGENCY MEDICAL INFORMATION CALL TOLL FREE: 1-800-328-0026
OUTSIDE NORTH AMERICA, CALL 1-651-222-5352
NOTE TO PHYSICIAN: Probable mucosal damage may contraindicate the use of gastric lavage.

PHYSICAL AND CHEMICAL HAZARDS: Strong oxidizing agent. Corrosive. Do not use in concentrated form. Mix only with water according to label instructions. Never bring concentrate in contact with other sanitizers, cleaners or organic substances.

(only required for containers 5 gallons or larger)
ENVIRONMENTAL HAZARDS: This pesticide is toxic to birds, fish, and aquatic invertebrates. Do not discharge effluent containing this product into lakes, streams, ponds, estuaries, oceans, or other waters unless in accordance with the requirements of a National Pollutant Discharge Elimination System (NPDES) permit and permitting authority has been notified in writing prior to discharge. Do not discharge effluent containing this product to sewer systems without previously notifying the local sewage treatment plant authority. For guidance contact your State Water Board or Regional Office of the EPA.

† One-step claims do not apply to *Clostridioides difficile* (formerly known as] [*Clostridium difficile*] [*C. difficile*] [C. diff] spores, which require[s] a pre-cleaning step before disinfection.
Note to reviewer. Wording in parentheses or brackets is interchangeable.

DIRECTIONS FOR USE

It is a violation of Federal law to use this product in a manner inconsistent with its labeling.

Combination Disinfection and Cleaning: This product is effective against the labeled organisms¥ at (3 fl. oz. per 1 gallon of water) (3 fl. oz. per 128 fl. oz. of water) (23 milliliters per 1 liter of water) in hard water (400 ppm as CaCO3) and 5% blood serum on hard non-porous surfaces. For visibly soiled areas a pre-cleaning step is required. Apply solution with mop, cloth, sponge, brush, scrubber, disposable wipes, or coarse spray device or by soaking so as to wet all surfaces thoroughly. Allow to remain wet for required contact time and then allow to air dry or if desired remove solution and entrapped soil with a clean wet mop, cloth, or wet vacuum pickup. Prepare a fresh solution daily or when it becomes soiled or diluted.

¥ Follow Sporicidal instructions for disinfection of [*Clostridioides difficile* (formerly known as] *Clostridium difficile*) spores

To Clean Hard, Non-Porous Surfaces: Apply this product diluted (3 fl. oz. per 1 gallon of water) (3 fl. oz. per 128 fl. oz. of water) (23 milliliters per 1 liter of water) onto soils and wipe clean [with a dry paper towel or lint-free cloth or microfiber cloth or sponge]. No rinsing necessary. [For best results, use a dry paper towel or lint-free cloth or microfiber cloth or sponge.] Repeat for visibly soiled areas. For stubborn stains or visibly soiled areas or tougher jobs, allow product to penetrate [dirt and/or soap scum] before wiping.

To Clean/Remove Soap Scum: Apply this product diluted (3 fl. oz. per 1 gallon of water) (3 fl. oz. per 128 fl. oz. of water) (23 milliliters per 1 liter of water) onto soils and wipe clean [with a dry paper towel or lint-free cloth or microfiber cloth or sponge]. No rinsing necessary. [For best results, use a dry paper towel or lint-free cloth or microfiber cloth or sponge.] Repeat for visibly soiled areas. For stubborn stains or visibly soiled areas or tougher jobs, allow product to penetrate [dirt and/or soap scum] before wiping. For best results, use regularly to prevent dirt and soap scum build up.

To Deodorize: Apply this product use solution to completely wet all surfaces. Let stand for 3 minutes to kill odor causing bacteria then wipe or allow to air dry. For visibly soiled areas, a pre-cleaning step is required.

To Clean and Deodorize Toilets: To clean and deodorize toilet bowl, squirt liberally (1/2 cup) product use solution on toilet sides and upper toilet bowl rim. Swab or brush all surfaces let stand for 3 minutes and flush.

Dilution: Disinfection (3 fl. oz. per 1 gallon of water) (3 fl. oz. per 128 fl. oz. of water) (23 milliliters per 1 liter of water).

This product is not to be used as a terminal sterilant/high level disinfectant on any surface or instrument that (1) is introduced directly into the human body, either into or in contact with the bloodstream, or normally sterile areas of the body, or (2) contacts intact mucous membranes, but which does not ordinarily penetrate the blood barrier or otherwise enter normally sterile areas of the body. This product may be used to pre-clean or decontaminate critical or semi-critical medical devices prior to sterilization or high level disinfection.

To Pre-clean Instruments Prior to Terminal Sterilization/High Level Disinfection: Apply this product diluted (3 fl. oz. per 1 gallon of water) (3 fl. oz. per 128 fl. oz. of water) (23 milliliters per 1 liter of water) and wipe clean [with a dry paper towel or lint-free cloth or microfiber cloth or sponge]. No rinsing necessary. [For best results, use a dry paper towel or lint-free cloth or microfiber cloth or sponge.] For stubborn stains or visibly soiled areas or tougher jobs, allow product to penetrate [dirt and/or soap scum] before wiping.

To Disinfect Non-Critical, Pre-cleaned Instruments: Instruments must be thoroughly pre-cleaned to remove excess organic debris, rinsed, and rough dried. Clean and rinse lumens of hollow instruments before filling with this product. [Spray] [submerge] all surfaces of instruments with this product use solution until thoroughly wet. Allow to remain wet for listed contact time. [To kill TB and Poliovirus, let stand for 10 minutes at room temperature.] Wipe with a clean, damp cloth or paper towel and allow to air dry.

Animal housing facilities [and poultry and swine premises]: This product use solution removes dirt, grime, fungus, blood, urine, fecal matter and other common soils found in animal housing facilities, grooming facilities, kennels, pet stores, veterinary clinics, laboratories or other small animal facilities. It [also] eliminates odors leaving surfaces smelling clean and fresh.

[This product] cleans, disinfects and deodorizes hard, non-porous inanimate surfaces in one-step† when used according to disinfection directions. Its non-abrasive formula is designed [for use on] [for daily use on] [for daily use to clean and disinfect] hard, non-porous inanimate surfaces found in animal housing facilities.

Disinfection of animal quarters and kennels: For disinfection of pre-cleaned animal quarters and kennels, apply this product use solution. Remove all animals and feed from premises. Remove all litter and droppings from floors, walls and surfaces of facilities occupied or traversed by animals. Empty all troughs, racks, and other feeding and watering appliances. Thoroughly clean all surfaces with soap or detergent and rinse with water. Saturate the surfaces with the disinfecting solution for required contact times. Ventilate building and other closed spaces. Do not house animals or employ equipment until treatment has been absorbed, set or dried. All treated equipment that will contact feed or drinking water must be scrubbed with soap or detergent and rinsed with potable water before reuse.

To Clean and Disinfect in a Veterinary Application: Use to clean and disinfect hard, non-porous surfaces such as feeding and watering equipment, cages, utensils, instruments, kennels, stables, catteries, etc. Remove all animals and feed from premises, animal transportation vehicles, crates, etc. Remove all litter, droppings, and manure from walls, floors, and surfaces of facilities occupied or traversed by animals. Empty all feeding and watering equipment. Pre-clean all surfaces with soap or detergent and rinse with water. Saturate surfaces with this product's use solution and let stand for required contact times. Ventilate buildings and other closed spaces. Do not house animals or employ equipment until treated surfaces have been thoroughly rinsed with water and allowed to dry. Thoroughly scrub all treating, feeding, and watering appliances with soap or detergent, and rinse with potable water before re-use.

Hospitals/Healthcare facilities:
This product use solution cleans, disinfects and deodorizes hard, non-porous hospital/medical surfaces in one-step† when used according to disinfection directions with no rinsing required.

This product use solution is a one-step† when used according to disinfection directions [hospital use] germicidal [disinfectant] cleaner and deodorant [odor counteractant] [odor neutralizer] designed for general cleaning, [and] disinfecting [deodorizing] [of] hard, non-porous inanimate surfaces. Quickly removes dirt, grime, food residue, blood and other organic matter commonly found in hospitals [healthcare facilities] [on medical surfaces]. It [also] eliminates odors leaving [restroom] surfaces smelling clean and fresh. Use where odors are a problem.

This product use solution is a [broad spectrum] [germicidal] [disinfectant] [disinfectant with sporicidal activity] cleaner and deodorizer designed for general cleaning [and] disinfecting [deodorizing] [of] hard, non-porous surfaces [and is efficacious against [*Clostridioides difficile* (formerly known as] [*Clostridium difficile*] [C. difficile] [C. diff] endospores after a pre-cleaning step]. Quickly removes dirt, grime, blood and other organic matter commonly found in hospitals [in healthcare facilities] [on medical surfaces].

Use where housekeeping is of prime importance in controlling the hazard of cross-contamination between treated hard, non-porous surfaces.

<u>DISINFECTANT WITH SPORICIDAL ACTIVITY AGAINST *Clostridium difficile*</u>
When applied to pre-cleaned surfaces, [this product] [OxyCide Daily Disinfectant Cleaner] kills and/or inactivates spores of [*Clostridioides difficile* (formerly known as] *Clostridium difficile*) on hard, non-porous surfaces. This product is effective against *C. difficile* endospores after a 5 minute exposure time.

SPECIAL INSTRUCTIONS FOR CLEANING PRIOR TO DISINFECTION AGAINST [*Clostridioides difficile* (formerly known as] ***Clostridium difficile*) SPORES**
Personal Protection: Wear appropriate barrier protection such as gloves, gowns, masks or eye covering.
Cleaning Procedure: Fecal matter/waste must be thoroughly cleaned from surfaces/objects before disinfection by application with a clean cloth, mop, and/or sponge saturated with the disinfectant product. This cleaning may be accomplished with any cleaning solution, including this product. Cleaning is to include vigorous wiping and/or scrubbing, until all visible soil is removed. Special attention is needed for high-touch surfaces. Surfaces in patient rooms are to be cleaned in an appropriate manner, such as from right to left or left to right, on horizontal surfaces, and top to bottom, on vertical surfaces, to minimize spreading of the spores. Restrooms are to be cleaned last. Do not reuse soiled cloths.
Contact time: Leave surface wet for 5 minutes with 3 fl. oz. per gallon use solution.
Infectious Materials Disposal: Materials used in the cleaning process that may contain feces/wastes are to be disposed of immediately in accordance with local regulations for infectious materials disposal.

<u>VIRUCIDAL*</u>
OxyCide Daily Disinfectant Cleaner inactivates the listed viruses at their corresponding contact times as listed [the presence of 5% organic soil load]. Apply as directed in the Disinfection / Cleaning Deodorizing directions for use.

This product kills HIV and HBV and HCV on pre-cleaned environmental surfaces/objects previously soiled with blood/body fluids in healthcare settings or other settings in which there is an expected likelihood of soiling of inanimate surfaces / objects with blood or body fluids, and in which the surfaces / objects likely to be soiled with blood or body fluids can be associated with the potential for transmission of Human Immunodeficiency Virus Type 1 (HIV-1) (associated with AIDS) or Hepatitis B Virus (HBV) or Hepatitis C Virus (HCV)

SPECIAL INSTRUCTIONS FOR CLEANING AND DECONTAMINATION AGAINST HIV-1 OR HBV OR HCV ON SURFACES / OBJECTS SOILED WITH BLOOD / BODY FLUIDS.
Personal protection: Clean-up must always be done wearing protective gloves, gowns, masks and eye protection.
Cleaning procedure: Blood and other body fluids containing HIV or HBV or HCV must be thoroughly cleaned from surfaces and objects before application of this product. This cleaning may be accomplished with any cleaning solution, including this product.

Contact time: Leave surface wet for 3 minutes for HIV-1 and 5 minutes for HBV and HCV with 3 fl. oz. per gallon use-solution.
Disposal of infectious material: Blood and other body fluids must be autoclaved and disposed of according to local regulations for infectious waste disposal.

FUNGICIDAL
This product is a one-step fungicide when diluted at 3 fl. oz. per gallon of water. This product kills *Candida auris* and *Candida albicans* after a contact time of 3 minutes, and *Trichophyton interdigitale* (formerly *Trichophyton mentagrophytes*) (the athlete's foot fungus) after a contact time of 10 minutes. OxyCide can be used in areas such as locker rooms, dressing rooms, shower and bath areas and exercise facilities.

SPECIAL LABEL INSTRUCTIONS FOR CLEANING PRIOR TO DISENFECTION AGAINST *Candida auris*

Personal Protection: Wear appropriate barrier protection such as gloves, gowns, masks, or eye covering.
Cleaning Procedure: Fecal matter/waste must be thoroughly cleaned from surfaces/objects before disinfection by application with a clean cloth, mop, and/or sponge saturated with the product. Pre-cleaning is to include vigorous wiping and/or scrubbing and all visible soil is removed. Surfaces in patient rooms are to be cleaned in an appropriate manner, such as from right to left or left to right, on horizontal surfaces, and top to bottom, on vertical surfaces, to minimize spreading the organism. Restrooms are to be cleaned last. Do not reuse soiled cloths.
Contact Time: Leave surface wet for 3 minutes with 3 fl. oz. per gallon use-solution.
Infectious Waste Disposal: Materials used in the cleaning process that may contain feces/wastes are to be disposed of immediately in accordance with local regulations for infectious materials disposal.

TABLE 1 General Use Sites:

This product is designed specifically as a general [non-abrasive] cleaner and disinfectant for use on hard, non-porous surfaces in:

Ambulatory Care Centers	ICU Areas	Pet Shops
Animal Life Science Laboratories	Locker Rooms	Public Restrooms
Athlete/Recreational Facilities	Lodging Establishments	Retail Businesses
Colleges	Long Term Care Centers	Schools
Cruise Ships	Manufacturing Facilities	Shower Rooms
Dental Offices	Nursing Homes	Surgical Centers
Food Service Establishments [Restaurants] [Commercial Kitchens]	Office Building	Transportation Terminals
Examination Rooms	Operating Rooms	Universities
Hospitals	Patient Rooms	Veterinary Clinics

MATERIAL COMPATABILITY

Baked enamel surfaces	[Finished] sealed floors	Stainless steel
Acrylic (plastic)	Flexible, non-porous surfaces	Chrome
Glazed ceramic tile	Glass	Vinyl [linoleum] [tile]
Glazed porcelain	Plastic and painted surfaces	Polished nickel finish
Laminated surfaces	Plastic surfaces	Shower stalls

NOTE: This product is compatible with the listed materials. It is recommended that you apply product to a smaller test area to determine compatibility before proceeding with its use.

This product is specifically designed to disinfect, deodorize and clean inanimate hard non-porous surfaces such as walls, floors, sink tops, furniture, patient beds, [and] operating tables, [kennel runs, cages and feeding and watering equipment]. In addition this product will deodorize those areas that are generally hard to keep fresh smelling such as garbage storage areas, empty garbage bins and cans, and any other areas that are prone to odors caused by microorganisms.

TABLE 2: Medical Use Sites

This product is designed for use on hard, non-porous surfaces in:

Ambulances or [Emergency Medical] Transport Vehicles	Eye Surgical Centers	Pharmacies
Ambulatory Care Centers	Hospitals	Physical Therapy Rooms or Patient Areas
Ambulatory Surgical Centers (ASC)	Intensive Care Units or ICU[s] [areas]	Physicians' Offices
Anesthesia Rooms or Areas	Isolation Areas	Physical therapy (PT) equipment surfaces
[Assisted Living or Full Care] Nursing Homes	Laboratories	Psychiatric Facilities
CAT Lab[oratories]	Laundry Rooms	Public [Care] Areas
Central Service Areas	Long Term Care Facilities	PVC tubing
Central Supply Rooms [Areas]	[Medical] Clinics	Radiology or X-Ray Rooms or Areas
Critical Care Units [CCUs]	Medical Facilities	Recovery Rooms
Dialysis Clinics [Facilities]	[Medical] [Physician's] [Doctor's] Offices	Rehabilitation Centers
Doctor's Offices	MRI or Magnetic Resonance Imaging equipment	Respiratory Centers
Donation Centers [blood] [plasma] [semen] [milk] [apheresis]	Non-porous hospital mattresses	Respiratory Therapy Rooms or Areas
Examination Rooms or Areas	Nursing Homes	Restrooms
Emergency Rooms [ERs]	Nursing or Nurses' Stations	Out-Patient [Surgical Centers (OPSC)] [Clinics] [Facilities]
Healthcare Settings or Facilities	Operating Rooms	[Surgery Rooms] [Operating Rooms] [Ors]
Home Healthcare Settings	Ophthalmic Offices	Waiting Rooms or Waiting Areas
Hospices	Orthopedics Facilities	

TABLE 3: Medical Use Surfaces

This product is designed for use on the following hard, non-porous surfaces and cannot be applied to any untreated wood surfaces:

anesthesia machines	[exam or examination] tables	overbed tables
apheresis machines	exterior surfaces of air vents or air vent exteriors	paddles
autoclaves	external surfaces of [medical] equipment or [medical] equipment surfaces	patient chairs
bathroom doorknob	[external] [surfaces of] ultrasound transducers [and/or probes]	patient monitoring equipment
bathroom surfaces	exterior of pipes	patient support and delivery equipment

bedpans	footboards	phlebotomy trays
bedrails	gurneys	phone cradle
[bedside] commodes	handheld [electronic] devices	Physical therapy (PT) equipment surfaces
bedside tables	[flexible, non-porous] edges of privacy curtains	plastic mattress covers
blood pressure cuffs	pagers	power cords
blood pressure (BP) monitors	hard, non-porous [environmental] hospital or medical surfaces	PVC tubing
cabinet handles	headboards	reception [counter] [desks] [areas]
call boxes	[Hospital] headboards, [external] [surfaces of] ultrasound transducers [and/or probes]	remote controls
CAT or Computerized Axial Tomography equipment	[hospital or patient] bed[s] [springs] [railings] [frames] [linings]	respirators
carts	[inner] [inside of] drawers	respiratory therapy equipment
[cellular] phones	isolettes	scales
chairs	IV [stands] [pumps] [poles]	sequential compression devices
charging stations	keyboards	shower fixtures
closet handles	laptops	side rails
coated mattresses	loupes	slit lamps
coated pillows	mammography equipment	spine backboards
computer mouse	[Mayo] [instrument] stands	stethoscopes
computer peripherals	medication carts	stools
computer screens	mobile devices	stretchers
computer tables	mobile electronic equipment	support bars
cords	mobile workstations	tablet PCs
counters	mouse pads	toilet handholds
[crash] [emergency] carts	MRI or Magnetic Resonance Imaging equipment	toilet surfaces
desktops	non-porous hospital mattresses	traction devices
diagnostic equipment	nurse-call [devices] [buttons] [and cords]	walls [around toilet] [in patient rooms]
dialysis machines	Nursing or Nurses' Stations	wash basins
docking stations	operating room tables and lights	wheelchairs
environmental surfaces	operatory light switches	x-ray equipment

Unless otherwise noted, using AOAC approved test methods (under Good Laboratory Practices, [GLPs]), in the presence of 5% blood serum and hard water up to 400 ppm hardness (calculated as $CaCO_3$), this product kills the following organisms on hard, non-porous inanimate surfaces.

TABLE 4: Listed Organisms and Contact Times

Organism	**ATCC Number**
**3 minute contact time**	
Pseudomonas aeruginosa	ATCC 15442
Salmonella enterica	ATCC 10708
Staphylococcus aureus	ATCC 6538
Staphylococcus aureus, (Methicillin Resistant [MRSA]	ATCC 33592

Staphylococcus aureus, (Genotype USA300) (Community Associated Methicillin Resistant) [CA-MRSA]	ATCC BAA-1556
Staphylococcus aureus, (Genotype USA400) (Community Associated Methicillin Resistant) [CA-MRSA]	ATCC BAA-1683
Staphylococcus aureus, (Intermediate Vancomycin Resistance) [VISA]	ATCC 700788
Enterococcus faecalis (Vancomycin Resistant) [VRE]	ATCC 51299
Staphylococcus epidermidis (Methicillin Resistant) [MRSE]	ATCC 51625
Streptococcus pneumoniae	ATCC 6303
Streptococcus pyogenes	ATCC 19615
Bordetella pertussis [Whooping Cough Virus]	ATCC12743
Escherichia coli	ATCC 11229
Escherichia coli (Extended-Spectrum Beta Lactamase producing) [ESBL]	ATCC BAA-196
Klebsiella pneumoniae	ATCC 4352
Klebsiella pneumoniae (Carbapenemase producer) [KPC]	ATCC BAA-1705
Acinetobacter baumannii	ATCC 19606
Acinetobacter baumannii (Multi-drug Resistant) [MDR]- gentamicin, imipenem, ceftazidime	ATCC BAA-1605
Proteus mirabilis	ATCC 7002
*Human Immunodeficiency Virus Type 1 (Strain HTLV-III$_B$) [HIV-1] [AIDS virus]	
*Human Coronavirus (Strain 229E)	ATCC VR-740
*Herpes Simplex Type I virus (F strain)	ATCC VR-733
*Herpes Simplex Type II virus (G strain)	ATCC VR-734
*Influenza A virus (Strain Hong Kong)	ATCC VR-544
*Respiratory Syncytial Virus (Strain Long) [RSV]	ATCC VR-26
*Vaccinia Virus (Strain WR) [Pox Virus]	ATCC VR-119
*Norovirus (feline calicivirus tested surrogate)	ATCC VR-782
*Rhinovirus (Type 37, Strain 151-1)	ATCC VR-1147
*Rotavirus (Strain WA)	
*Adenovirus Type 5	ATCC VR-5
Candida albicans	ATCC 10231
Candida auris	AR-BANK #3081
*SARS-Related Coronavirus 2 [SARS-CoV-2]	(BEI Resources NR-52281) (Strain Isolate USA-WA 1/2020)
**5 minute contact time**	**ATCC Number**
Clostridioides difficile (Clostridium difficile)	ATCC 43598
*Hepatitis B virus (as duck hepatitis B virus)	
*Hepatitis C virus (as bovine viral diarrhea virus)	
**10 minute contact time**	**ATCC Number**
Mycobacterium bovis BCG [TB]	
*Poliovirus (Type 1, Chat strain)	ATCC VR-1562
Trichophyton interdigitale (formerly *mentagrophytes*)	ATCC 9533

Optional Marketing Claims:

- Effective in 5 minutes against [*Clostridioides difficile* (formerly known as] [*Clostridium difficile] [C. difficile]* [C. diff] spores
- Kills [*Clostridioides difficile* (formerly known as] [*Clostridium difficile] [C. difficile]* [C. diff] spores in 5 minutes.
- Compliant with new test method [for developing efficacy data supporting *C. difficile* claims] [Quantitative Method for Testing Antimicrobial Products Against Spores of *Clostridium difficile* (ATCC 43598) on Inanimate, Hard, Non-porous Surfaces]
- Tested under the [insert current test method] as of [date of test method]
- Concentrated broad-spectrum disinfectant with efficacy against [*Clostridioides difficile* (formerly known as] [*Clostridium difficile] [C. difficile]* [C. diff] spores
- Effective one-step† disinfectant-cleaner when used according to disinfection directions [with sporicidal activity against *Clostridium difficile* when used with a pre-cleaning step] for use in hospitals [ambulatory care centers, long term care facilities, and other healthcare settings]
- Proven "one-step" † used according to disinfection directions disinfectant – virucide which is effective in water up to 400ppm hardness in the presence of 5% serum contamination
- Proven "one-step" † when used according to disinfection directions disinfectant – virucide.
- May be used as part of a comprehensive approach to *Clostridium difficile* spore control
- Is designed for killing [*Clostridioides difficile* (formerly known as] [*Clostridium difficile*] [*C. difficile*] [C. diff] spores [on surfaces] [on pre-cleaned, hard non-porous surfaces] in hospitals
- Effective against [**insert any organism from list of organisms**] and [*Clostridium difficile*] [*C. difficile*] [C. diff] spores [in hospitals]
- Effective for daily use against [**insert any organism from list of organisms**] [and] [*Clostridioides difficile* (formerly known as] [*Clostridium difficile*] [*C. difficile*] [C. diff] spores [in hospitals]
- Kills [*Clostridioides difficile* (formerly known as] [*Clostridium difficile*] [*C. difficile*] [C. diff] spores in 5 minutes.
- Daily use product with [*Clostridioides difficile* (formerly known as] [*Clostridium difficile*] [*C. difficile*] [C. diff] spore efficacy [allows for product standardization] [eliminates need for separate disinfectant with sporicidal activity] [bleach]
- Proactive daily defense against [*Clostridioides difficile* (formerly known as] [*Clostridium difficile*] [*C. difficile*] [C. diff] spores on treated hard, non-porous surfaces
- Tough on [*Clostridioides difficile* (formerly known as] [Clostridium difficile] [C. difficile] [C. diff] spores but easy on surfaces and designed for daily use.
- Effective in 3 minutes against *Candida auris*
- Economical disinfectant with sporicidal activity designed for daily cleaning and easy on surfaces.
- Effective against Multidrug Resistant Organisms [MDROs] (*Staphylococcus aureus,* (Resistant to Methicillin [MRSA], *Staphylococcus aureus*, (Genotype USA300) (Community Associated Methicillin Resistant) [CA-MRSA], *Staphylococcus aureus*, (Genotype USA400) (Community Associated Methicillin Resistant) [CA-MRSA], *Staphylococcus aureus*, (Intermediate Vancomycin Resistance) [VISA], *Enterococcus faecalis* (Resistant to Vancomycin) [VRE], *Staphylococcus epidermidis* (Resistant to Methicillin) [MRSE], *Escherichia coli* (Extended-Spectrum Beta Lactamase producing) [ESBL], *Klebsiella pneumoniae* (Carbapenemase producer) [KPC].
- [Effective against] [Disinfects] [Kills] [99.9% when used according to label directions] SARS-CoV-2 virus on hard, non-porous surfaces

- [Effective against] [Disinfects] [Kills] [99.9% when used according to label directions] SARS-CoV-2 virus, the cause of COVID-19 on hard, non-porous surfaces
- Kills [Eliminates] 99.9% SARS-Related Coronavirus 2 when used according to label directions [SARS-CoV-2] [USA-WA1/2020] [causative agent of COVID-19][the virus that causes COVID-19] on hard, non-porous surfaces
- Effective against SARS-Related Coronavirus 2 [SARS-CoV-2] [USA-WA1/2020] [in 3 minutes] on hard, non-porous surfaces
- Disinfects hard, non-porous surfaces by killing [99.9% when used according to label directions] SARS-Related Coronavirus 2 [SARS-CoV- 2] [USA-WA1/2020] [in One Step]
- Kills [99.9% of] SARS-CoV-2, which causes COVID-19 on hard, non-porous surfaces when used according to label directions
- [Kills] [Effective against] the virus* that causes COVID-19 (**SARS-CoV-2)* on hard, non-porous surfaces
- Cuts cleaning time
- [Is a disinfectant cleaner that] cleans, disinfects and deodorizes in one labor saving step.
- Cross-contamination is of major housekeeping concern. This product has been formulated to aid in the reduction of cross-contamination between treated hard, non-porous surfaces not only in hospitals, but in schools, institutions and industry.
- This product kills, removes and destroys germs, bacteria and viruses*(SARS-CoV-2) on hard, non-porous surfaces.
- Contains hydrogen peroxide
- Daily cleaning
- Designed for non-critical, hard, non-porous surfaces in healthcare
- Makes cleaning easier
- The smell of clean
- Evaporates completely
- Clear drying formula
- Leaves no visible residue
- Will not leave grit or soap scum.
- No rinsing
- Removes and/or cleans [insert stains(s)/soils(s) from list below]

Bathtub ring	Grime
Blood	Laboratory stains
Body oils	Other soils and/or stains
Dirt	Other organic matter
Fecal matter	Urine

- Cleans
- Cleans everyday messes
- Cleans to a shine
- Fast strong cleaning
- For discharge cleaning
- Fragrance free.
- No added [perfumes] [fragrances] [and] [or] [dyes]
- Good for use with microfiber cloths
- May be used to clean and disinfect hard, non-porous finished floors. Cleans and disinfects without dulling gloss
- May be used to clean and disinfect floor areas, sinks, faucets, bathrooms and tubs
- Do not use on marble or un-sealed/un-coated terrazzo floors
- Suitable for use on flexible, non-porous surfaces

- Compatible with [sites] [surfaces] [material compatibility chart]
- Multi surface cleaner disinfectant on hard, non-porous surfaces
- Non-abrasive
- For non-scratch cleaning of showers and tubs, shower doors and curtains, fixtures and toilet bowls.
- Is a complete, chemically balanced disinfectant that provides clear use solutions even in the presence of hard water.
- [Chlorine] bleach free, does not contain [chlorine] bleach, non [chlorine] bleach
- Will not [stain] [discolor] [bleach] uniforms or fabrics
- Color safe
- This product does not damage furnishings, equipment or clothing.
- Tough on germs, easy on surfaces
- Economical concentrate
- Is effective yet economical.
- Is an economical concentrate [that can be diluted for use] [with a mop and bucket, cloth, microfiber cloth, sponge, disposable wipe, coarse spray device or by soaking].
- Intended for use with the Ecolab [insert name of appropriate dispenser]
- Closed loop automated dispensing reduces employee exposure to concentrate product.
- Closed loop automated dispensing reduces the risk of spills.
- Ecolab [insert name of appropriate dispenser] controls dilution to reduce waste of concentrate.
- Ecolab [insert name of appropriate dispenser] ensures appropriate ppm levels of actives in use solution.
- Ecolab [insert name of appropriate dispenser] makes accurate dispensing quick and easy.
- Non-flammable
- Concentrate [Concentrated].
- Disinfects [Disinfectant] on hard, non-porous surfaces.
- Cleans [Cleaner]
- Designed for daily use on common materials found in hospitals.
- Degradable active ingredients
- After product has been diluted according to label directions, PPE is not required.

DEODORIZATION

Claims:

- Deodorizes [Deodorant] [Deodorizer]
- Deodorizes by killing microorganisms that causes offensive odors.
- Kills odor causing bacteria
- No harsh alcohol smell
- No harsh bleach smell
- This product will deodorize hard, non-porous surfaces [where obnoxious odors may develop] [sites] [surfaces] [material compatibility chart]
- This product will deodorize surfaces and other places bacteria growth can cause malodors [sites] [surfaces] [material compatibility chart]
- This product deodorizes areas that are hard to keep fresh smelling.
- Counteracts [common] hospital malodors
- Will disinfect, clean and deodorize hard, non-porous surfaces in rest rooms and toilet areas, behind and under sinks and counters, garbage cans and garbage storage areas, and other hard, non-porous surfaces where bacterial growth can cause malodors.

† One-step claims do not apply to *Clostridioides difficile* (formerly known as] [*Clostridium difficile*] [*C. difficile*] [C. diff] spores, which require[s] a pre-cleaning step before disinfection.

STORAGE & DISPOSAL

DO NOT CONTAMINATE WATER, FOOD OR FEED BY STORAGE OR DISPOSAL

PESTICIDE STORAGE: Product must be kept cool and in a vented container to avoid any explosion hazard. [Do not store in [direct] sunlight]. [Vented container, store upright].

PESTICIDE DISPOSAL: Pesticide wastes are acutely hazardous. Improper disposal of excess pesticide, spray mixture, or rinsate is a violation of Federal Law. If these wastes cannot be disposed of by use according to label instructions, contact your State Pesticide or Environmental Control Agency, or the Hazardous Waste representative at the nearest EPA Regional Office for guidance.

(*Non-refillable sealed container is designed to reduce worker exposure to the concentrate. This container cannot be triple rinsed because it is a closed container. The following text will be used on this container.)*

CONTAINER DISPOSAL: Non-refillable sealed container. Do not reuse or refill this container. Wrap empty container and put in trash.

((INTENDED) FOR INSTITUTIONAL USE)
STRONG OXIDIZING AGENT

EPA Reg. No. 1677-237

EPA Est. 60156-IL-1(SI), 72806-OK-1(AD), 1677-IL-2(J), 1677-TX-1(D), 1677-GA-1(M), 1677-CA-2(R), 1677-MN-1(P), 1677-WV-1(V), 70271-CA-2(A), 303-IN-1(L).
The superscript refers to first letter of date code.

Net Contents: 96 U.S. fl oz (2.84 L)
1 U.S. Gal. (3.78 L)

Ecolab Inc.
1 Ecolab Place
St. Paul, MN 55102

(Made in United States of America) (Made in USA)

This product may be patented│ Ce produit peut être breveté │ Este producto puede ser patentado:
www.ecolab.com/patents
© 20XX Ecolab USA Inc • All rights reserved

SECONDARY/USE DILUTION CONTAINER LABEL

(Note to reviewer: This secondary/use dilution container label will be used only when the product is diluted at 3 fl. oz. per gallon of water) When this product is diluted in accordance with the directions on this label, the dilutions container must bear the following statements:

OxyCide™ Daily Disinfectant Cleaner

(Use Solution Ingredient Statement)

Active Ingredients:

Hydrogen Peroxide 0.63%
Peroxyacetic Acid 0.13%
Other Ingredients: 99.24%
Total: 100.0%

The product in this container is diluted as directed on the pesticide product label.

Diluted product in this container is 3 fl. oz. per gallon water.

KEEP OUT OF REACH OF CHILDREN

PRECAUTIONARY STATEMENTS
HAZARDS TO HUMANS AND DOMESTIC ANIMALS
Harmful if swallowed. Wash thoroughly with soap and water after handling and before eating, drinking, chewing gum, using tobacco or using the toilet.

(FIRST AID
IF SWALLOWED: Call a poison control center or doctor immediately for treatment advice. Have a person sip a glass of water if able to swallow. Do not induce vomiting unless told to do so by the poison control center or doctor. Do not give anything by mouth to an unconscious person.)

After product has been diluted according to label directions, PPE is not required.

Follow the directions for use listed on the pesticide label when applying this product.

Use solution prepared by end-user
Not for sale or distribution
(Do Not Drink)

EPA Reg. No. 1677-237

EMERGING VIRAL PATHOGENS

This product qualifies for emerging viral pathogen claims per the EPA's "Guidance to Registrants: Process for Making Claims Against Emerging Viral Pathogens not on EPA-Registered Disinfectant Labels" when used in accordance with the appropriate use directions indicated below.

(Note to the reviewer: The statements shall be made only through the following communications outlets: technical literature distributed exclusively to long term care professionals, food safety professionals, environmental services professionals, health care facilities, physicians, nurses, veterinarians and public health officials, "1-800" consumer information services, social media sites and company websites (non-label related). *These statements shall not appear on marketed (final print) product labels.)*

This product meets the criteria to make claims against certain emerging viral pathogens from the following viral category[ies]:

- Enveloped Viruses
- Large Non-Enveloped Viruses
- Small Non-Enveloped Viruses

For an emerging viral pathogen that is a/an…	*…follow the directions for use for the following organisms on the label*
Enveloped virus	Feline calicivirus, Rhinovirus
Large, non-enveloped virus	Feline calicivirus, Rhinovirus
Small, non-enveloped virus	Feline calicivirus, Rhinovirus

[OxyCide Daily Disinfectant Cleaner] has demonstrated effectiveness against viruses similar to [name of emerging virus] on hard, non-porous surfaces. Therefore, [OxyCide Daily Disinfectant Cleaner] can be used against [name of emerging virus] when used in accordance with the directions for use against [name of supporting virus(es)] on hard, non-porous surfaces. Refer to the [CDC or OIE] website at [pathogen-specific website address] for additional information.

OxyCide™ Daily Preparation
OxyCide Preparación diaria

Personal Protective Equipment*
*Equipo de Protection Personal**

Gloves
Guantes

Glasses
Gafas

Work Bottle Filling
Llenado de la botella de trabajo

1
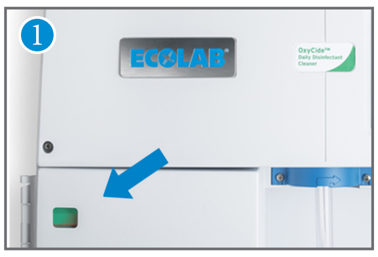
Confirm low product indicator shows mostly **GREEN**
Confirmar indicador de producto bajo muestra principalmente VERDE

2
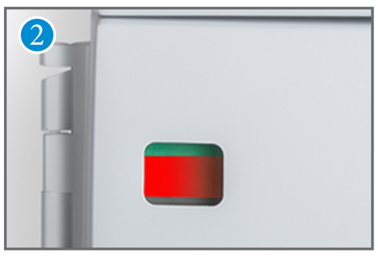
When low product indicator shows 3/4 **RED** notify supervisor to refill
Si el indicador de producto bajo muestra 3/4 ROJO, notifique al supervisor que rellene

3
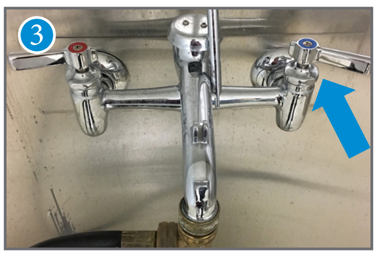
Turn **COLD** water on fully
Abra por complete el flujo de agua FRIA

4
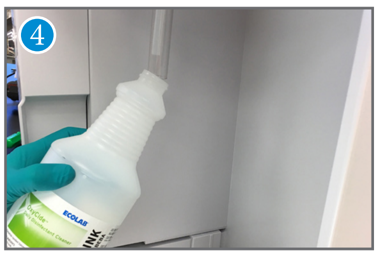
Insert fill tube into empty work bottle
Inserte el tubo de llenado en la botella de trabajo vacía

5
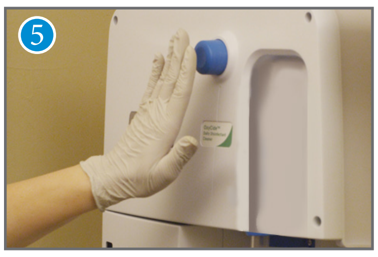
Press and hold button until bottle is full
Presione y mantenga presionado el botón hasta que la botella esté llena

6
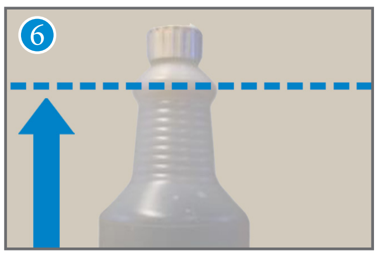
Stop at the top
Detener en la parte superior

7
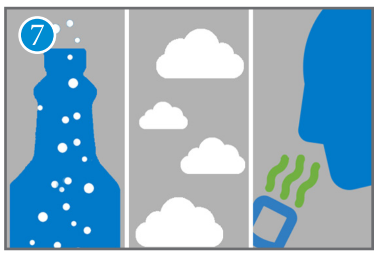
Look for signs that product is being dispensed
Fizzy/Cloudy/Vinegar Odor
If no signs observed, notify supervisor
Busque señales de que el producto se está dispensando
Burbuja/Nublado/Olor a vinagre
Si no hay señales observadas, notifique al supervisor

8
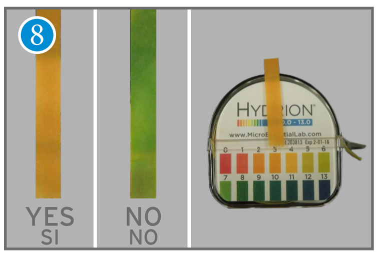

Optional Dispenser Check (Supervisor Only)
Place pH strip in dispensed solution to verify dispenser is working
ORANGE = Proper operation
GREEN = Call for service
Comprobación del dispensador opcional (Supervisor solamente)
Coloque la tira de pH en solución para verificar que el dispensador esté funcionando
NARANJA = producto dispensado
VERDE = llamar al servicio

9
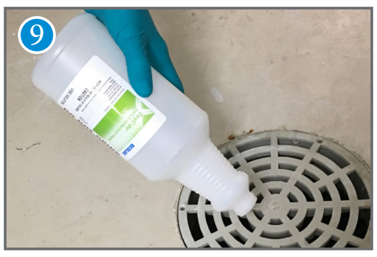
Empty work bottle or bucket into sink, toilet, or mop drain and rinse at end of shift
Vacíe la botella de trabajo o el cubo en el fregadero, el inodoro o el desagüe del trapeador y enjuague al final del turno

Cloth Bucket Filling
Llenado de la cubeta para el trapo

Your Ecolab representative will determine the proper number of cloths per bucket during installation and training. Cloths should be damp, but not dripping.
Su representante de Ecolab determinará el número adecuado de paños por cubo durante la instalación y el entrenamiento. Los paños deben estar húmedos, pero no gotear.

______ 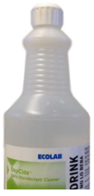 + ______ 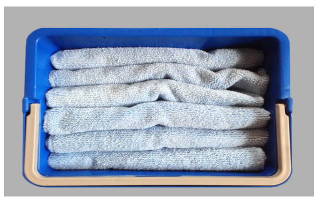

Number of 32 oz work bottles
Númer de botellas de 32 oz

Number of cloths
Númer de paños

1
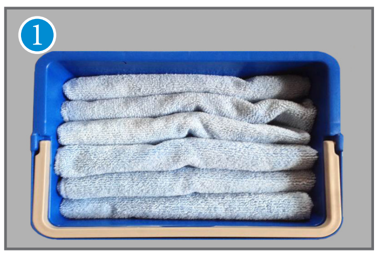
Prepare cloths in bucket
Preparar los paños en un cubo

2
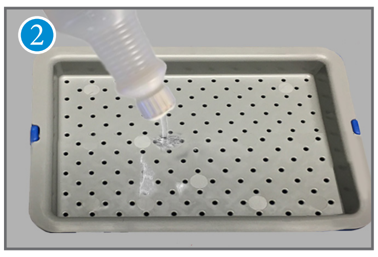
Pour OxyCide over sieve onto cloths
Vierta OxyCide sobre el colador ubicado encima de los paños

3
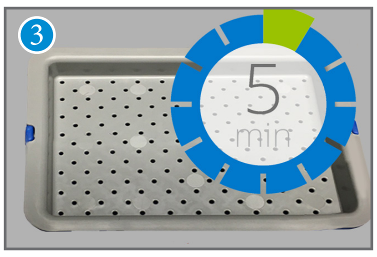

Allow cloths to soak for 5 minutes before use
Deje que los paños se sumerjan 5 minutos antes del uso

4
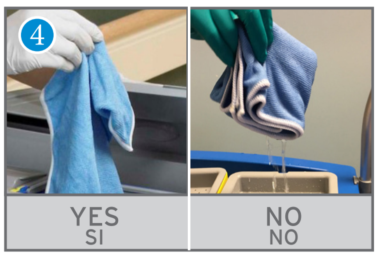

Cloths should be DAMP, but NOT DRIPPING
Los paños deben estar HÚMEDOS, pero NO GOTEAR

5 MINUTE Overall Contact Time
MINUTO en general Contacto Hora

1 DAY SHELF LIFE
Empty Bottle or Bucket After Shift or within 24 hours
Botella vacía o cubo
Después de cambiar o en 24 horas

Optional
Opcional

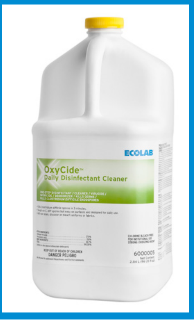

Supervisor:
Place concentrate bottle expiration date sticker here

Coloque la etiqueta de fecha de caducidad del concentrado de OxyCide aquí

World Headquarters
1 Ecolab Place, St. Paul, MN 55102
www.ecolab.com 866 781 8787
©2017 Ecolab Inc. All rights reserved.
44542/8502/0517 92023079

* The SDS for OxyCide indicates that no personal protective equipment is required for handling the diluted product. As best practice when dispensing any cleaning proudcts, however, we recommend using gloves and glasses.

* *El SDS para OxyCide indica que no se requiere equipo de protección personal para el manejo del producto diluido. Sin embargo, como mejor práctica al dispensar cualquier producto de limpieza, recomendamos usar guantes y gafas.*

ECOLAB®

OxyCide™ Daily Preparation
OxyCide Preparación diaria

Personal Protective Equipment*
*Equipo de Protection Personal**

Gloves *Guantes* Glasses *Gafas*

Work Bottle Filling
Llenado de la botella de trabajo

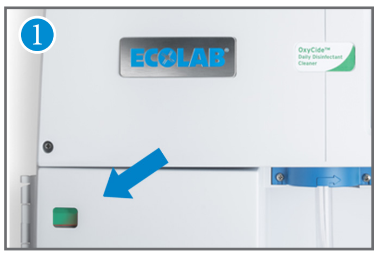

Confirm low product indicator shows mostly **GREEN**
Confirmar indicador de producto bajo muestra principalmente VERDE

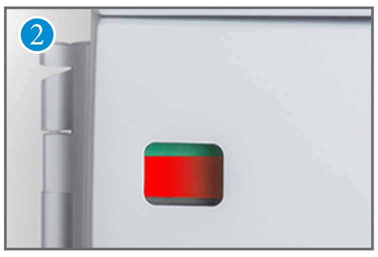

When low product indicator shows 3/4 **RED** notify supervisor to refill
Si el indicador de producto bajo muestra 3/4 ROJO, notifique al supervisor que rellene

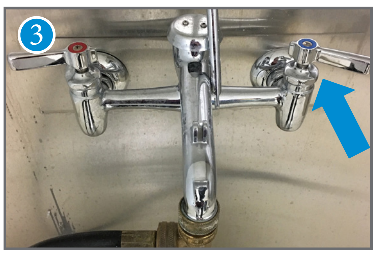

Turn **COLD** water on fully
Abra por complete el flujo de agua FRIA

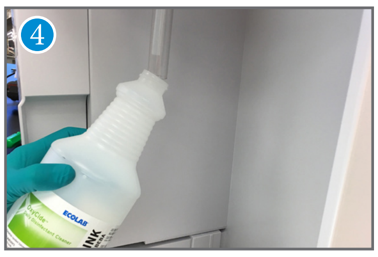

Insert fill tube into empty work bottle
Inserte el tubo de llenado en la botella de trabajo vacía

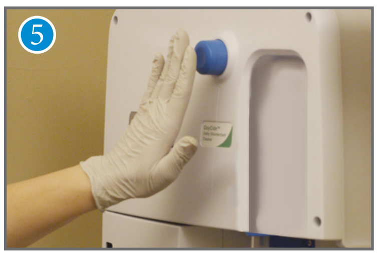

Press and hold button until bottle is full
Presione y mantenga presionado el botón hasta que la botella esté llena

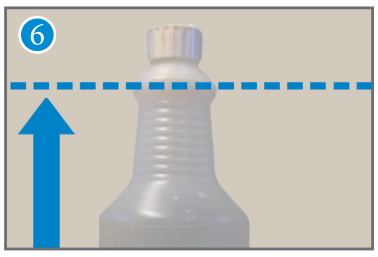

Stop at the top
Detener en la parte superior

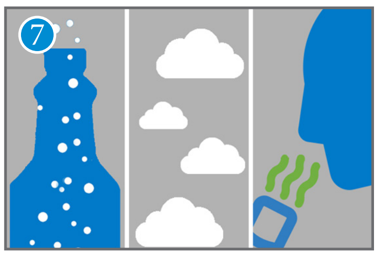

Look for signs that product is being dispensed
Fizzy/Cloudy/Vinegar Odor
If no signs observed, notify supervisor
Busque señales de que el producto se está dispensando
Burbuja/Nublado/Olor a vinagre
Si no hay señales observadas, notifique al supervisor

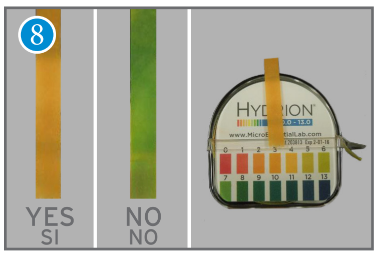

Optional Dispenser Check (Supervisor Only)
Place pH strip in dispensed solution to verify dispenser is working
ORANGE = Proper operation
GREEN = Call for service
Comprobación del dispensador opcional (Supervisor solamente)
Coloque la tira de pH en solución para verificar que el dispensador esté funcionando
NARANJA = producto dispensado
VERDE = llamar al servicio

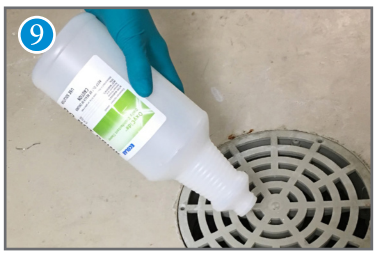

Empty work bottle or bucket into sink, toilet, or mop drain and rinse at end of shift
Vacíe la botella de trabajo o el cubo en el fregadero, el inodoro o el desagüe del trapeador y enjuague al final del turno

Dry Wipe Instructions
Instrucciones para el paño seco

Your Ecolab representative will determine the proper amount of OxyCide to use for each wipe container during installation and training.
Su representante de Ecolab determinará la cantidad adecuada de OxyCide que se debe usar para cada recipiente de limpieza durante la instalación y el entrenamiento.

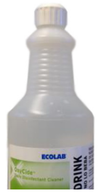

\+

Number of 32 oz work bottles
Númer de botellas de 32 oz

Container and Size
(35 count, 90 count, 64 oz, etc.)
Cubo y tamaño

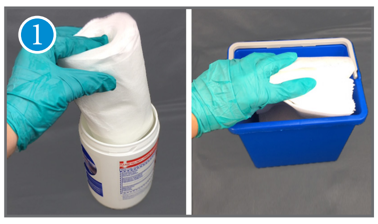

Place dry wipes in container or bucket
Coloque los paños seco en un cubo o cubeat

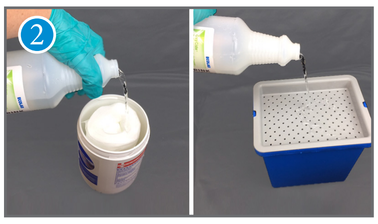

Pour OxyCide over wipes in a circular pattern
Vierta el OxyCide sobre los paños en un patrón circular

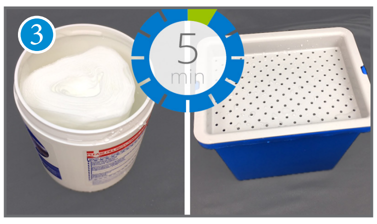

Allow wipes to soak for 5 minutes before use
Deje que los paños se sumerjan 5 minutos antes del uso

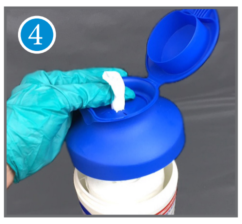

Place cap on container and pull first wipe through cap
Coloque la tapa sobre el recipiente y tire de la primera tela a través de la tapa

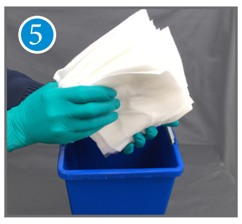

Wipes should be DAMP, but NOT DRIPPING
Los paños deben estar HÚMEDOS, pero NO GOTEAR

5 MINUTE Overall Contact Time
MINUTO en general Contacto Hora

1 DAY SHELF LIFE
Empty Bottle or Bucket
After Shift or within 24 hours
Botella vacía o cubo
Después de cambiar o en 24 horas

Optional
Opcional

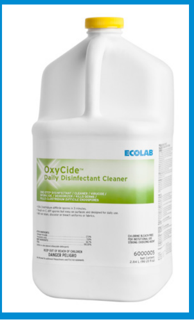

Supervisor:
Place concentrate bottle expiration date sticker here

Coloque la etiqueta de fecha de caducidad del concentrado de OxyCide aquí

World Headquarters
1 Ecolab Place, St. Paul, MN 55102
www.ecolab.com 866 781 8787
©2017 Ecolab Inc. All rights reserved.
44542/8502/0517 92023079

* The SDS for OxyCide indicates that no personal protective equipment is required for handling the diluted product. As best practice when dispensing any cleaning proudcts, however, we recommend using gloves and glasses.

* *El SDS para OxyCide indica que no se requiere equipo de protección personal para el manejo del producto diluido. Sin embargo, como mejor práctica al dispensar cualquier producto de limpieza, recomendamos usar guantes y gafas.*

ECOLAB®

CDC Guidance on Environmental Cleaning Techniques

https://www.cdc.gov/hai/prevent/resource-limited/cleaning-procedures.html

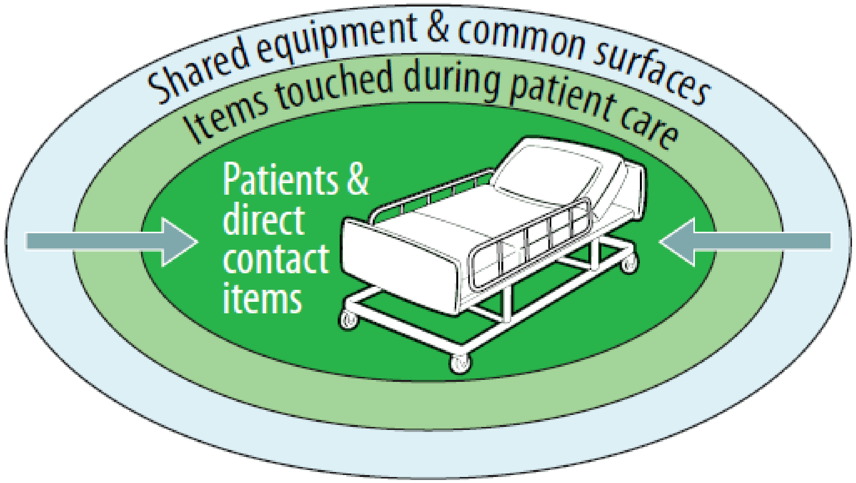

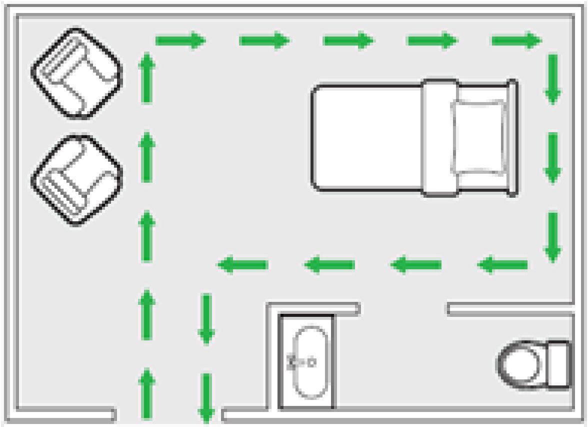

--Proceed **from cleaner to dirtier** areas to avoid spreading dirt and microorganisms

--Within a specified patient room, terminal cleaning should start with **shared equipment and common surfaces**, then proceed **to surfaces and items touched during patient care** that are outside of the patient zone, and finally to **surfaces and items directly touched by the patient** inside the patient zone

--Proceed from **high to low** to prevent dirt and microorganisms from dripping or falling and contaminating already cleaned areas.

--Proceed in a **systematic manner** to avoid missing areas—for example, left to right or clockwise

CDC Examples of High-Touch Surfaces in the Patient Area

https://www.cdc.gov/hai/prevent/resource-limited/high-touch-surfaces.html

High touch surfaces include,
but are not limited to:

- bed rails
- bed frames
- moveable lamps
- tray table
- bedside table
- handles
- IV poles
- blood-pressure cuff

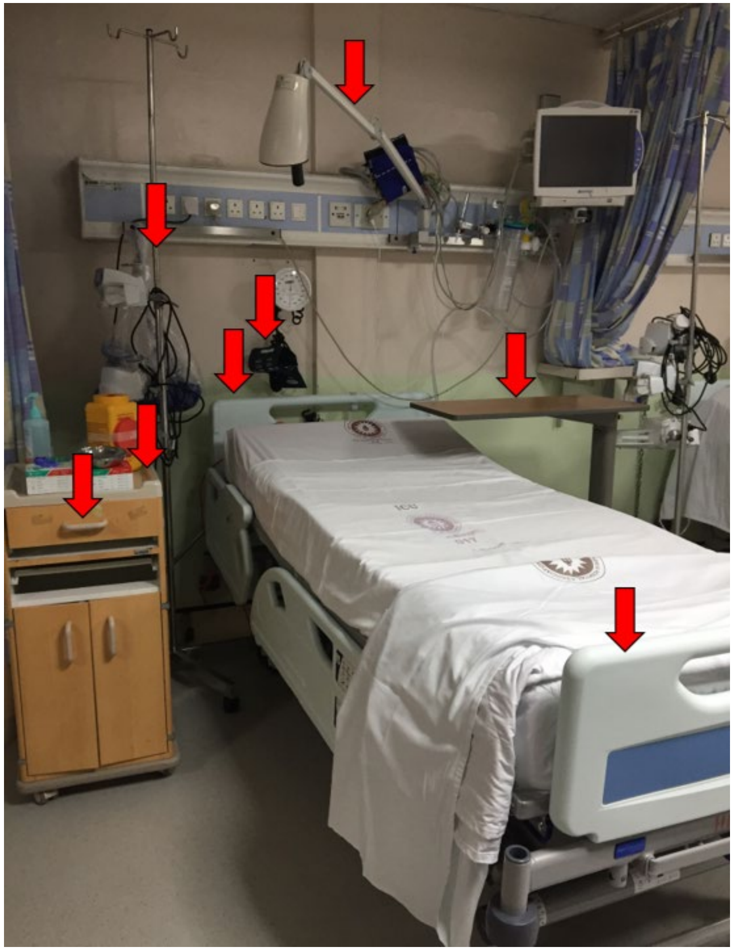

OXYCIDE DAILY DISINFECTANT CLEANER

CLASSIFICATION

- One–Step Disinfectant, Cleaner, *Virucide, Sporicide, Deodorizer

PRODUCT DESCRIPTION

- ***OxyCide Daily Disinfectant Cleaner*** is effective in three minutes against *Clostridium difficile* spores.
- ***OxyCide Daily Disinfectant Cleaner*** is a daily use product with C. diff efficacy allows for product standardization and eliminates the need for a separate sporicide.
- ***OxyCide Daily Disinfectant Cleaner*** This product use solution is a one step hospital use disinfectant cleaner and deodorant designed for general cleaning, disinfecting and deodorizing of hard, nonporous inanimate surfaces. Quickly removes dirt, grime, fungus, food residue, blood and other organic matter commonly found in health care facilities. It eliminates odors leaving surfaces smelling clean and fresh. Use where odors are a problem.
- ***OxyCide Daily Disinfectant Cleaner*** does not damage furnishings, equipment, or clothing.

FEATURES AND BENEFITS

Feature	Benefit
**Speed:** 3 min contact time for 31 of 33 organisms including *C. difficile.*	Dwell time compliance with JCAHO audits.
**Broad spectrum:** 33 organisms including *C. difficile.*	Daily defense against *C. difficile.* from proactive sporicide use. Improvement in environmental outcomes due to reduction in bioburden.
**Material compatibility:** Good compatibility with most surfaces and furnishings in a hospital including finished floors and uniforms. Zero residue or film buildup.	Allows for daily use in patient rooms, ORs and common areas. Reduced risk and cost of damaged goods requiring replacement. Surfaces remain looking clean without requiring post-disinfection wiping to remove residue.
**Safety:** Category III for use dilution.	No PPE required when handling diluted chemistry (gloves recommended in a healthcare setting).
**Speed, broad spectrum, material compatibility and safety**	Combined features allow for product standardization and simplification. Reduces complexity, confusion and error compared to using and training on multiple products. No need to think about which product to use, at which dilution based on the room type or patient condition. Eliminates the time needed for secondary cleaning/disinfection or residue removal. Reduces error in reporting to surveyors.

OxyCide Daily Disinfectant Cleaner is specifically designed to disinfect, deodorize and clean inanimate hard non-porous surfaces such as walls, floors, sink tops, furniture, patient beds, operating tables, kennel runs, cages and feeding and watering equipment. In addition this product will deodorize those areas that are generally hard to keep fresh smelling such as garbage storage areas, empty garbage bins and cans, and any other areas that are prone to odors caused by microorganisms.

Areas of Use:

- ***OxyCide Daily Disinfectant Cleaner*** is for use in Healthcare Settings or Facilities including Hospitals, Ambulances, Ambulatory Care Centers Ambulatory Surgical Centers, Eye Surgical Centers, Anesthesia Rooms or Areas, Pharmacies, Assisted Living or Full Care Nursing Homes, Physical Therapy Rooms or Areas, Patient Areas, Physicians' Offices, Psychiatric Facilities, Home Health Care Settings, Public Areas, Hospices Radiology or X-Ray Rooms or Areas, CAT Laboratories, Recovery Rooms, Rehabilitation Centers, Intensive Care Units or ICUs, Central Service Areas, Isolation Areas, Central Supply Areas, Laboratories, Respiratory Centers, Respiratory Therapy Rooms or Areas, Laundry Rooms Restrooms, Long Term Care Facilities, Medical Facilities, Medical Offices, Medical Clinics, Critical Care Units, Operating Rooms, Dialysis Facilities, Nursing Homes, Waiting Areas, Nurses' Stations, Donation Centers, Examination Rooms, Emergency Rooms, Ophthalmic Offices, Out-Patient Surgical Centers and Facilities.
- ***OxyCide Daily Disinfectant Cleaner*** is for use on hard, nonporous hospital or medical surfaces, bedpans, environmental surfaces, high touch surfaces, patient chairs, overbed tables, phone cradles, hard, nonporous edges of privacy curtains, patient beds, bedrails, side rails, headboards, footboards, surfaces, plastic mattress covers, counters, bedside tables, IV stands, remote controls, cabinet handles, inside of drawers, closet handles, coated mattresses, coated pillows, wash basins, bathroom doorknob, commodes, shower fixtures, toilet handholds, walls around toilet, nurse-call devices and cords, call boxes, reception areas, chairs, scales, exterior surfaces of air vents, PVC tubing, exterior of pipes, pagers, cellular phones, mobile devices, charging stations, desktops, mobile workstations, laptops, docking stations, computer screens, keyboards, computer mouse, mouse pads, tablet PCs, mobile electronic equipment, computer tables, computer peripherals, external surfaces of medical equipment, ultrasound transducers and probes, patient monitoring equipment, patient support and delivery equipment, anesthesia machines, phlebotomy trays, apheresis machines, gurneys, handheld electronic devices, autoclaves, Physical therapy (PT) equipment, power cords, blood pressure cuffs blood pressure (BP) monitors, pulse oximeters, CAT or Computerized Axial Tomography equipment, MRI or Magnetic Resonance Imaging equipment, isolettes, carts, respirators, respiratory therapy equipment, loupes, sequential compression devices, mammography equipment, instrument stands, medication carts, slit lamps, spine backboards, cords, stethoscopes, stools, emergency carts, stretchers, support bars, diagnostic equipment, dialysis machines, traction devices, operating room tables and lights, operatory light switches, wheelchairs, x-ray equipment, paddles, examination tables, Orthopedics.
- ***OxyCide Daily Disinfectant Cleaner*** is not to be used as a terminal sterilant / high level disinfectant on any surface or instrument that (1) is introduced into the human body, either into or in contact with the bloodstream or normally sterile areas of the body, or (2) contacts intact mucous membranes but which does not ordinarily penetrate the blood barrier or otherwise enter normally sterile area of the body. This product may be used to pre-clean or decontaminate critical or semi-critical medical devices prior to sterilization of high-level disinfection

CONCENTRATE PRECAUTIONARY STATEMENTS

- **KEEP OUT OF REACH OF CHILDREN**
- **DANGER.** Causes digestive tract, eye and skin burns. May be fatal if inhaledor swallowed. Oxidizer. Contact with other material may cause fire. Causes respiratory tract irritation. Harmful if absorbed through the skin. Do not ingest. Do not get in eyes, on skin, or on clothing. Do not breathe vapor spray. Keep away from heat, sparks and flame. Store only in the original, properly sealed vented container. Avoid contact with combustible materials. Keep away from heat and direct sunlight. Decomposes on heating. Use only with adequate ventilation. Wash thoroughly after handling.
- The following Personal Protective Equipment (PPE) should be used when handling the concentrated product: Due to the form and packaging of the product, no protective equipment is needed under normal use conditions. Chemical splash goggles, impervious gloves and apron should be used when there is a likelihood of exposure to concentrated product.
- After product has been diluted according to label directions PPE is not required.

USE DILUTION PRECATIONARY STATEMENTS

- **WARNING**
- Harmful if swallowed. Do not ingest. Wash thoroughly after handling.
- **IF SWALLOWED**: Rinse mouth; then drink one or two large glasses of water. Do not induce vomiting. Never give anything by mouth to an unconscious person. Get medical attention immediately.
- After product has been diluted according to label directions PPE is not required.

DIRECTIONS FOR USE

- It is a violation of Federal law to use this product in a manner inconsistent with its labeling.

COMBINATION DISINFECTION AND CLEANING:

- **This product** is effective against the labeled organisms at (3.0oz. per 1 gallon of water) (3 oz per 128 oz of water) (23 milliliters per 1 liter of water) in hard water (400 ppm as $CaCO3$) and 5% blood serum on hard nonporous surfaces. For heavily soiled areas a precleaning step is required.
- Apply solution with mop, cloth, sponge, brush, scrubber, or coarse spray device or by soaking so as to wet all surfaces thoroughly.
- Allow to remain wet for required contact of 3, 5 or 10 minutes and then either allow to air dry or if desired remove solution and entrapped soil with a clean wet mop, cloth, or wet vacuum pickup.
- Prepare a fresh solution daily or when it becomes soiled or diluted

To Clean/Remove Soap Scum:

- Apply this product onto soils and wipe clean with a dry paper towel or lint-free cloth or microfiber cloth or sponge.
- No rinsing necessary
- Repeat for heavily soiled areas.
- For stubborn stains or heavily soiled areas or tougher jobs, allow product to penetrate dirt and/or soap scum before wiping.
- For best results, use regularly to prevent dirt and soap scum build up.

To Deodorize:

- Apply this product use solution to completely wet all surfaces.
- Let stand for 3 minutes to kill odor causing bacteria then wipe or allow to air dry.
- For heavily soiled areas, a precleaning is required.

To Clean and Deodorize Toilets:

- To clean and deodorize toilet bowl, squirt liberally (1/2 cup) on toilet sides and upper toilet bowl rim.
- Swab or brush all surfaces let stand for 3 minutes and flush.

BACTERICIDAL

- ***OxyCide Daily Disinfectant Cleaner*** is effective against the following pathogenic bacteria at a contact time of 3 minutes in the presence of 5% organic soil load. Apply as directed in the Disinfection / Cleaning / Deodorizing directions for use.

Pseudomonas aeruginosa
Salmonella enterica
Staphylococcus aureus
Staphylococcus aureus, Methicillin Resistant [MRSA]
Staphylococcus aureus, (Genotype USA300) (Community Associated Methicillin Resistant) [CA-MRSA]
Staphylococcus aureus, (Genotype USA400) (Community Associated Methicillin Resistant) [CA-MRSA]
Staphylococcus aureus, (Intermediate Vancomycin Resistance) [VISA]
Enterococcus faecalis (Resistant to Vancomycin) [VRE]
Staphylococcus epidermidis (Resistant to Methicillin) [MRSE]
Streptococcus pneumoniae
Streptococcus pyogenes
Bordetella pertussis [Whooping Cough]
Escherichia coli
Escherichia coli (Extended-Spectrum Beta Lactamase producing) [ESBL]
Klebsiella pneumoniae
Klebsiella pneumoniae (Carbapenemase producer) [KPC]
Acinetobacter baumannii
Acinetobacter baumannii (Multi-drug Resistant) [MDR]
Clostridium difficile (Endospores)
Proteus mirabilis
Candida albicans

SPORICIDAL
Clostridium difficile

- ***OxyCide Daily Disinfectant Cleaner*** kills and/or inactivates spores of *Clostridium difficile* on hard, non-porous surfaces.
- ***OxyCide Daily Disinfectant Cleaner*** is effective against *Clostridium difficile* endospores after a 3 minute exposure time.

SPECIAL INSTRUCTIONS FOR CLEANING PRIOR TO DISINFECTION AGAINST *Clostridium difficile* SPORES

- **Personal Protection:** Wear appropriate barrier protection such as gloves, gowns, masks or eye covering.
- **Contact time:** Leave surface wet for 3 minutes with 3oz. per gallon use solution.
- **Cleaning Procedure:** Fecal matter/waste must be thoroughly cleaned from surfaces/objects before disinfection by application with a clean cloth, mop, and/or sponge saturated with the disinfectant product. This cleaning may be accomplished with any cleaning solution, including this product. Cleaning is to include vigorous wiping and/or scrubbing, until all visible soil is removed. Special attention is needed for high-touch surfaces. Surfaces in patient rooms are to be cleaned in an appropriate manner, such as from right to left or left to right, on horizontal surfaces, and top to bottom, on vertical surfaces, to minimize spreading of the spores. Restrooms are to be cleaned last. Do not reuse soiled cloths.
- **Infectious Materials Disposal:** Materials used in the cleaning process that may contain feces/wastes are to be disposed of immediately in accordance with local regulations for infectious materials disposal.

FOR ADDITIONAL LABEL CLAIMS PLEASE REFER TO THE PRODUCT LABEL.

VIRUCIDAL*

- ***OxyCide Daily Disinfectant Cleaner*** is effective against the following viruses after the listed contact time in the presence of 5% organic soil load. Apply as directed in the Disinfection / Cleaning / Deodorizing directions for use.

3 minute contact time
*Human Immunodeficiency Virus Type 1 (StrainHTLV-IIIB) [HIV-1] [AIDS virus]
*Herpes Simplex Type I virus (F strain)
*Herpes Simplex Type II virus (G strain
*Human Coronavirus (Strain 229E)
*Influenza A virus (Strain Hong Kong)
*Respiratory Syncytial Virus (Strain Long) [RSV]
*Vaccinia Virus (Strain WR) [Pox Virus]
*Norovirus (feline calicivirus tested surrogate)
*Rhinovirus (Type 37, Strain 151-1)
*Rotavirus (Strain WA)

5 minute contact time
*Hepatitis B virus (as duck hepatitis B virus)
*Hepatitis C virus (as bovine viral diarrhea virus)

*Additional claims see product label.

****OxyCide Daily Disinfectant Cleaner*** kills HIV and HBV and HCV on pre-cleaned environmental surfaces/objects previously soiled with blood/body fluids in health care settings or other settings in which there is an expected likelihood of soiling of inanimate surfaces / objects with blood or body fluids, and in which the surfaces / objects likely to be soiled with blood or body fluids can be associated with the potential for transmission of Human Immunodeficiency Virus Type 1 (HIV-1)(associated with AIDS) or Hepatitis B Virus (HBV) or Hepatitis C Virus (HCV).

- **SPECIAL INSTRUCTIONS FOR CLEANING AND DECONTAMINATION AGAINST HIV-1 (AIDS VIRUS) AND HBV (HEPATITIS B VIRUS) OF SURFACES/ OBJECTS SOILED WITH BLOOD/BODY FLUIDS.**
- **Personal protection:** Disposable latex or vinyl gloves, gowns, face masks, or eye coverings must be worn during all cleaning of body fluids, blood, and decontamination procedures.
- **Cleaning procedure:** Blood and other body fluids containing HIV or HBV or HCV must be thoroughly cleaned from surfaces and objects before application of this product. This cleaning may be accomplished with any cleaning solution, including this product.
- **Contact time:** Leave surface wet for 3 minutes for HIV-1 and 5 minutes for HBV and HCV with 3oz. per gallon use-solution.
- **DISPOSAL OF INFECTIOUS MATERIALS**: Blood and other body fluids should be autoclaved and disposed of according to local regulations for infectious waste disposal.

SPECIFICATIONS

**Active Ingredient**	- Peroxyacetic Acid<br>- Hydrogen Peroxide	5.8%<br>27.5%
Physical state	Liquid	
Color	Colorless	
Odor	Pungent	
pH (Concentrate)	0.49 [Conc. (%w/w): 100%]	
pH (Use dilution)	3.06	
Relative density	1.12 (Water = 1)	
Shelf life	270 days from date of manufacture	

REGULATORY REFERENCES

- EPA Reg. No. 1677-237

ENVIRONMENT & DISPOSAL

- Concentrate Hazard Rating: Health – 3; Fire – 1; Physical Hazards – 1
- Use Dilution Hazard Rating: Health – 1; Fire – 0; Physical Hazards – 0
- Pesticide disposal: Pesticide wastes are acutely hazardous. Improper disposal of excess pesticide, spray mixture, or rinsate is a violation of Federal Law. If these wastes cannot be disposed of by use according to label instructions, contact your State Pesticide

or Environmental Control Agency, or the Hazardous Waste representative at the nearest EPA Regional Office for guidance.
- CONTAINER DISPOSAL: Non-refillable container. Do not reuse this container to hold materials other than pesticides or diluted pesticide rinsate. Offer for recycling if available or puncture and dispose in a sanitary landfill, or by other procedures approved by state and local authorities.

STABILITY & STORAGE
- DO NOT CONTAMINATE WATER, FOOD OR FEED BY STORAGE OR DISPOSAL
- PESTICIDE STORAGE: Product should be kept cool and in a vented container to avoid any explosion hazard.
- Do not store above 35ºC.

CONCENTRATE FIRST AID OTHER INFORMATION
- **SKIN CONTACT**: In case of contact, immediately flush skin with plenty of water for at least 15 minutes while removing contaminated clothing and shoes. Get medical attention immediately. Wash clothing before reuse. Clean shoes thoroughly before reuse.
- **EYE CONTACT**: In case of contact, immediately flush eyes with cool running water. Remove contact lenses and continue flushing with plenty of water for at least 15 minutes. Get medical attention immediately.
- **INHALATION:** If inhaled, remove to fresh air. If exposed person is not breathing, give artificial respiration or oxygen applied by trained personnel. Get medical attention immediately.
- **INGESTION**: If material has been swallowed and the exposed person is conscious, give small quantities of water to drink. Do not induce vomiting. Never give anything by mouth to an unconscious person. Get medical attention immediately.
- Have the product container or label with you when calling a poison control center or doctor, or going for treatment.
- For emergency medical information worldwide, call toll free: 1-651-328-0026.

USE DILUTION FIRST AID OTHER INFORMATION
- **SKIN CONTACT**: No known effect after skin contact. Rinse with water for a few minutes.
- **EYE CONTACT**: No known effect after eye contact. Rinse with water for a few minutes.
- **INHALATION:** No special measures required. Treat symptomatically.
- **INGESTION**: Rinse mouth; then drink one or two large glasses of water. Do not induce vomiting. Never give anything by mouth to an unconscious person. Get medical attention immediately.
- Have the product container or label with you when calling a poison control center or doctor, or going for treatment.
- For emergency medical information worldwide, call toll free: 1-651-328-0026.

PACKAGING & PRODUCT NUMBERS

Size Available	Product Number
1 gallon	6000005
Not for sale in Canada	

Worldwide Headquarters
370 Wabasha Street N St. Paul, MN 55102
www.ecolab.com 1.866.781.8787

© 2015 Ecolab Inc. All rights reserved. 979252 0915

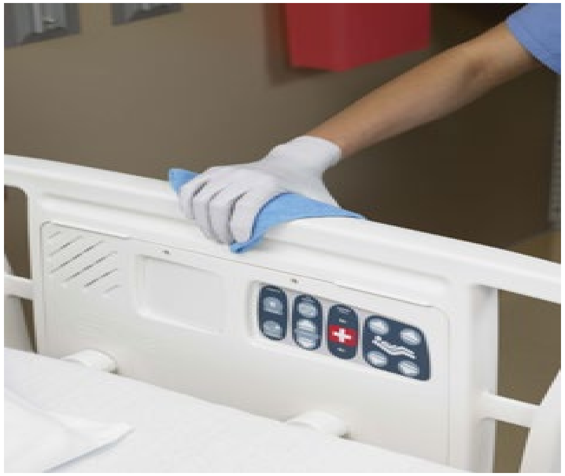

EVS STAFF IN-SERVICE TRAINING

2017

ECOLAB®

Objectives

- Introduce OxyCide
- Explain Product and Dispenser Features
- Review Key Safety Points
- **Reduce the Spread of Infection**

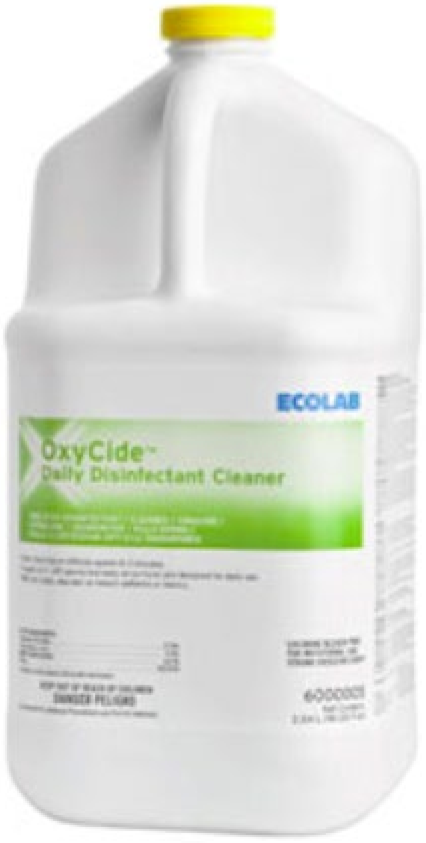

ECOLAB

HEALTHCARE-ASSOCIATED INFECTIONS

Healthcare-Associated Infections

"Healthcare-Associated Infections (HAIs) are the most common complication in hospitalized patients in the U.S."

- **1 in every 25** hospital patients has an HAI.[1]
- 75,000 die every year in the US from HAIs.[2]
- *C. diff* is linked to **14,000** deaths per year in the US.[2]

CDC Centers for Disease Control and Prevention
CDC 24/7: Saving Lives, Protecting People™

ECOLAB®

1 http://www.cdc.gov/HAI/surveillance/index.html
2 cdc.gov/VitalSigns/Hai/StoppingCdifficile

Microorganisms/Germs Survival on Environmental Surfaces

Pathogen	Approx. Lifespan on Hard Surfaces
MRSA	9 months
Staphylococci	7 months
C. Diff	> 5 months
VRE	4 months
Norovirus	3 weeks
SARS, HIV, etc.	Days to week
H1N1	Few days

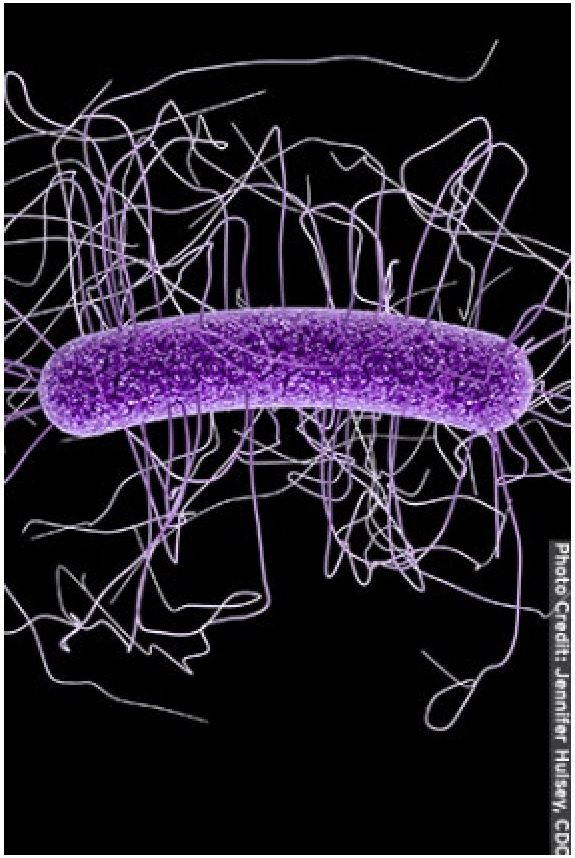

ECOLAB®

Source: Science Digest Article/Improving Cleaning Carling

Clostridium Difficile ("C diff") Video

- https://www.ecolab.com/expertise-and-innovation/resources/microbial-risks/clostridium-difficile

ECOLAB®

What You Do Matters!

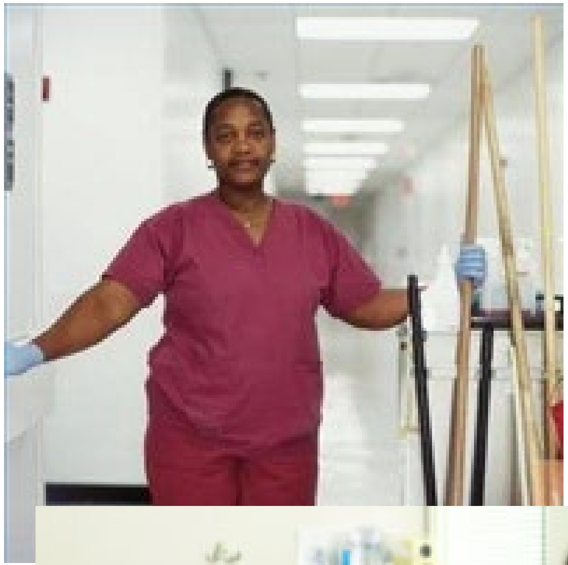

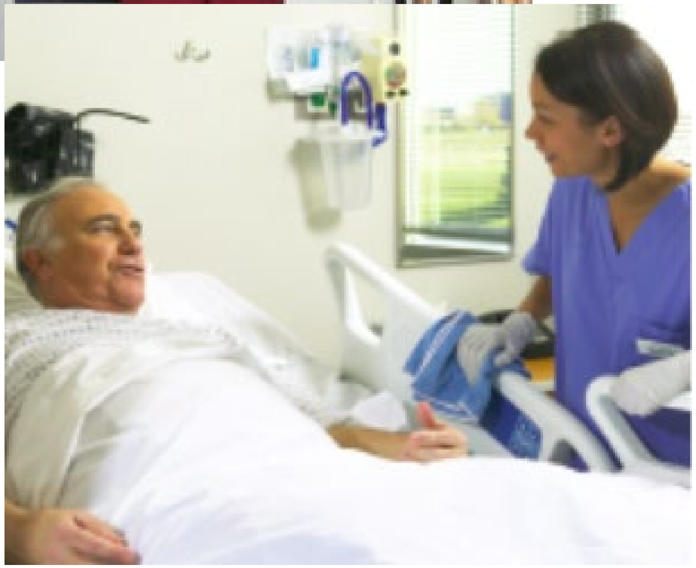

YOU help prevent the spread of germs

YOU increase patient safety

YOU keep staff & families healthy

ECOLAB

WHAT IS OXYCIDE?

What is OxyCide?

- One-step cleaner, disinfectant and **sporicide**
- **Contact time is 5 min.**
- Two active ingredients:
  - PEROXYACETIC ACID breaks down outer membrane of the spore, bacteria or virus
  - HYDROGEN PEROXIDE destroys the inner component (DNA, proteins)
- Chemistry EPA-registered since 1977 & used in many industries

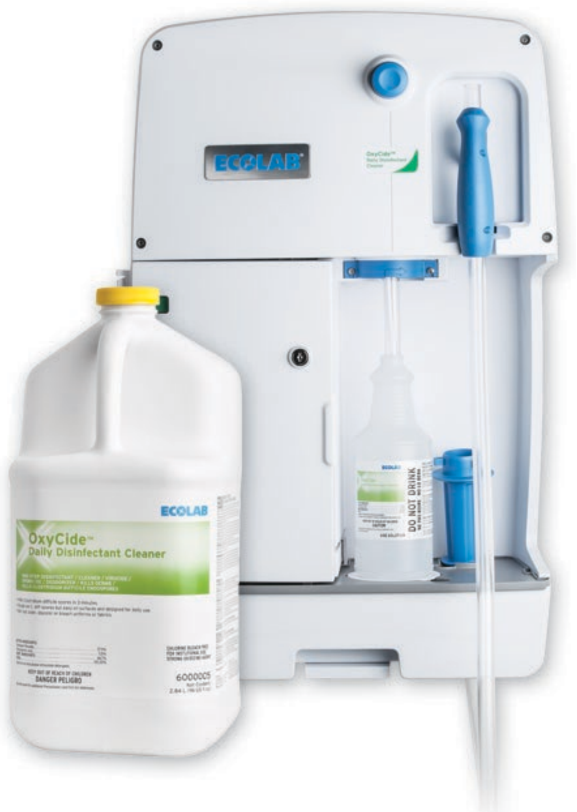

Why OxyCide?

- To ***proactively kill C. difficile*** without harmful effects of bleach
- To ***increase patient safety*** & improve patient outcomes
- To create ***efficiency***
  - 5 minute contact time
  - 1 product for all surfaces
- To ***simplify*** the cleaning process
  - One product, everyday use
  - Reduces rework
- To ***reduce damage*** to work surfaces & uniforms

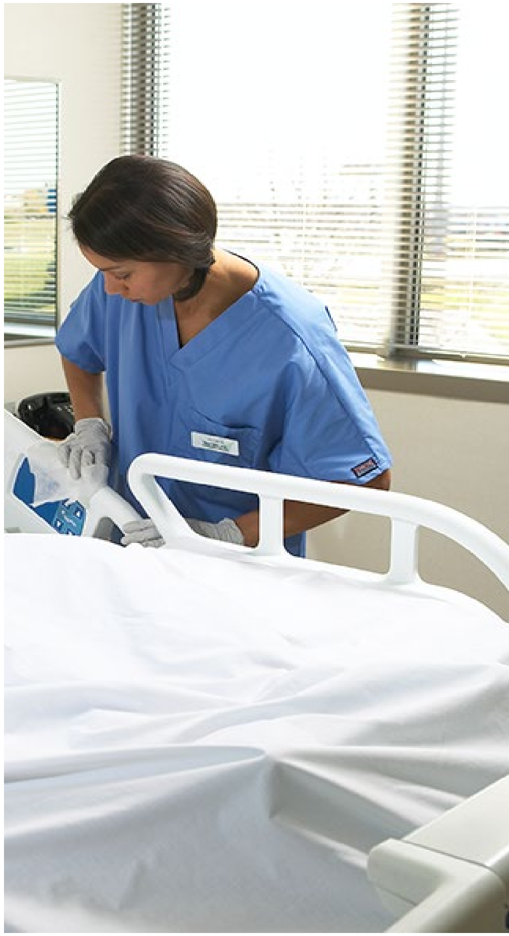

OxyCide Acceptance

- EPA Registered & Approved
- EPA Safer Choice Partner of the Year Award 2016
  - Recognizes organizations that advance chemical safety by developing products that perform well and are safer for human health and the environment
- Safety Data Sheet (SDS)
  - Health rating of 1 in use-solution (scale of 1-4 with 4 being highest)
  - Quaternary disinfectants also typically rated as 1

Where Will OxyCide be Used ?

- Patient Rooms: Daily, Terminal and Isolation Cleaning
- Operating Room: Terminal Cleaning only
- Common Areas: Lobbies, Public Rest Rooms, Nurse Stations
- Focus on High Touch Objects (HTOs)

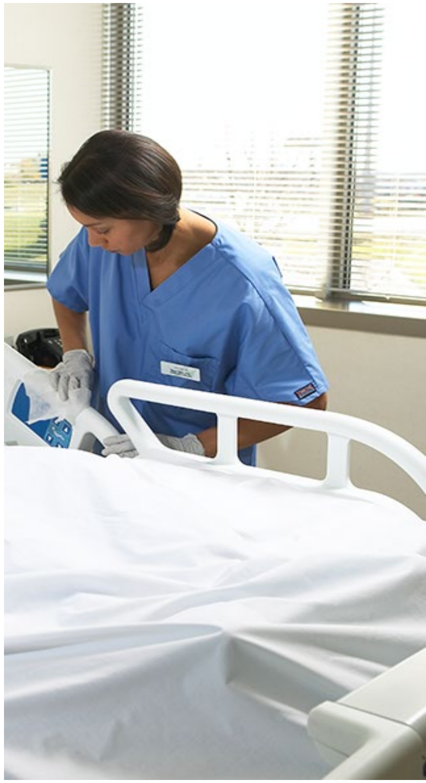

A Room is a Room is a Room

- Follow your facility's policy for cleaning of electronics (Computer screens, TV screens, X-ray viewer boxes)
- Nursing staff will continue using their current products

ECOLAB

What is NOT Changing?

- **Cleaning policies**
- **Bloodborne** Pathogens Protocol
- **Isolation policies**
- **Hand Hygiene**
- Handling of **linen**
- Handling of **waste**

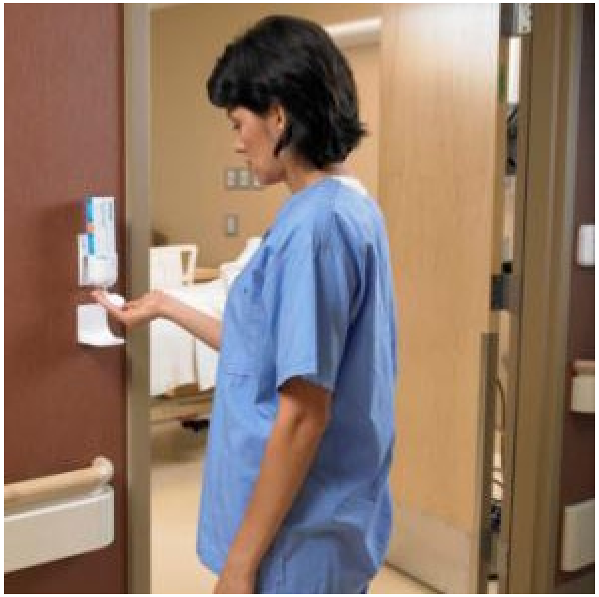

How to Use OxyCide

- Dispenser creates OxyCide **use solution**

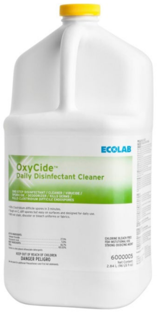

Concentrate

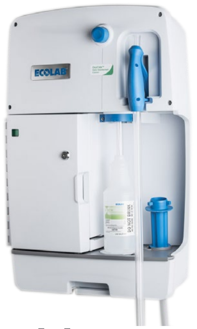

Mixed with water through OxyCide dispenser

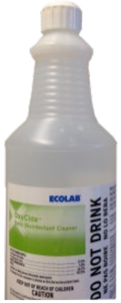

Diluted use solution

- Physical State: Liquid
- Odor: Vinegar-like
- Color: Clear

Once diluted, shelf life is 24 hours (dump at end of shift)

How to Use OxyCide (MICROFIBER)

- Always wear **gloves** and **glasses** when dispensing any cleaning products
- Wear **gloves** when cleaning environmental surfaces as best practice
- Start of shift:
  - **Prepare fresh OxyCide use solution**
  - 10 Microfiber cloths to 1 - 32oz. work bottle of OxyCide
  - Prepare 1 – 32 oz. work bottle of OxyCide for your cart
  - Prepare Johnny mop caddy per facility practice
- End of Shift:
  - **Use any remaining OxyCide use solution (wipe down cart, tools, etc.). Pour balance down toilet or mop sink drain and rinse with water.**
  - Place any unused pre-saturated cloths in soiled linen bag
  - Rinse work bottle, bucket and Johnny mop caddy

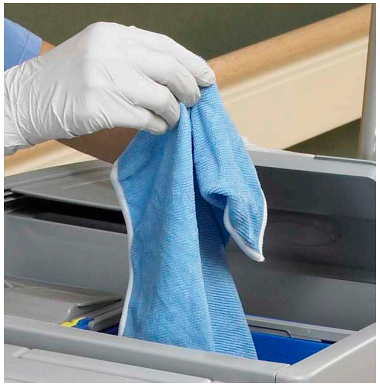

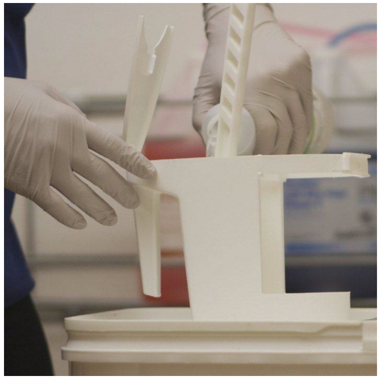

OXYCIDE DISPENSER

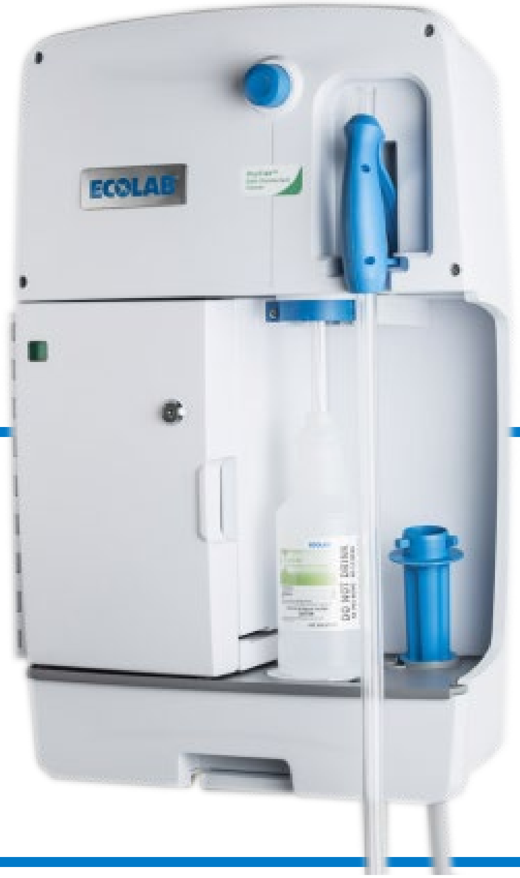

OxyCide™ Dispenser

Low Product Indicator

Shows when the concentrate bottle is nearly empty. When ¼ **GREEN** and ¾ **RED,** let your supervisor know the concentrate bottle needs to be changed.

Lock Product Cabinet

Prevents unauthorized access to concentrate product and Overflow Tray.

Overflow Tray

Collects any drips from concentrate and use solution. Only supervisors will empty this tray.

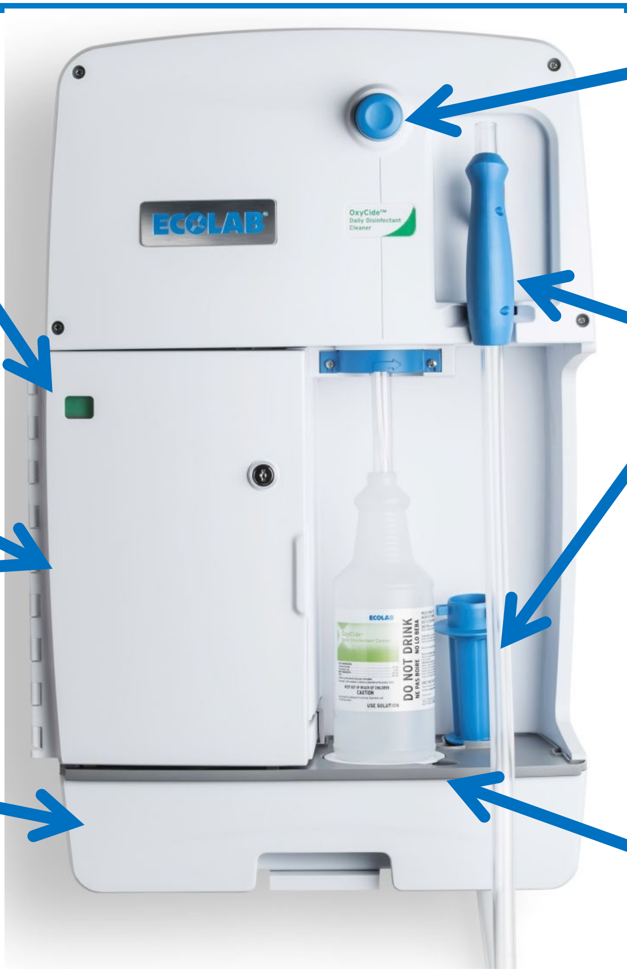

Dispensing Button

Press to start chemical flow. Let go to stop chemical flow. No hands-free dispensing option.

Bucket Fill Hose Docking Station and Holder

Long hose for filling buckets. Use to hold the bucket fill hose adaptor when not in use. Place handle into docking station. Place fill adaptor into resting area.

Bottle Fill Ledge

Short hose for filling work bottles. Ledge holds work bottle when filling. Empty drip tray when needed.

Remember: Turn **COLD** water fully on and **fill an EMPTY bottle to the top** **WITHOUT STOPPING** the chemical flow.

Low Product Indicator (LPI)

- Look at **EACH AND EVERY TIME** before filling a work bottle
- If the low product indicator is ¼ GREEN and ¾ RED, notify your supervisor to change out the concentrate bottle
    - About 2 - 4 work bottles remain

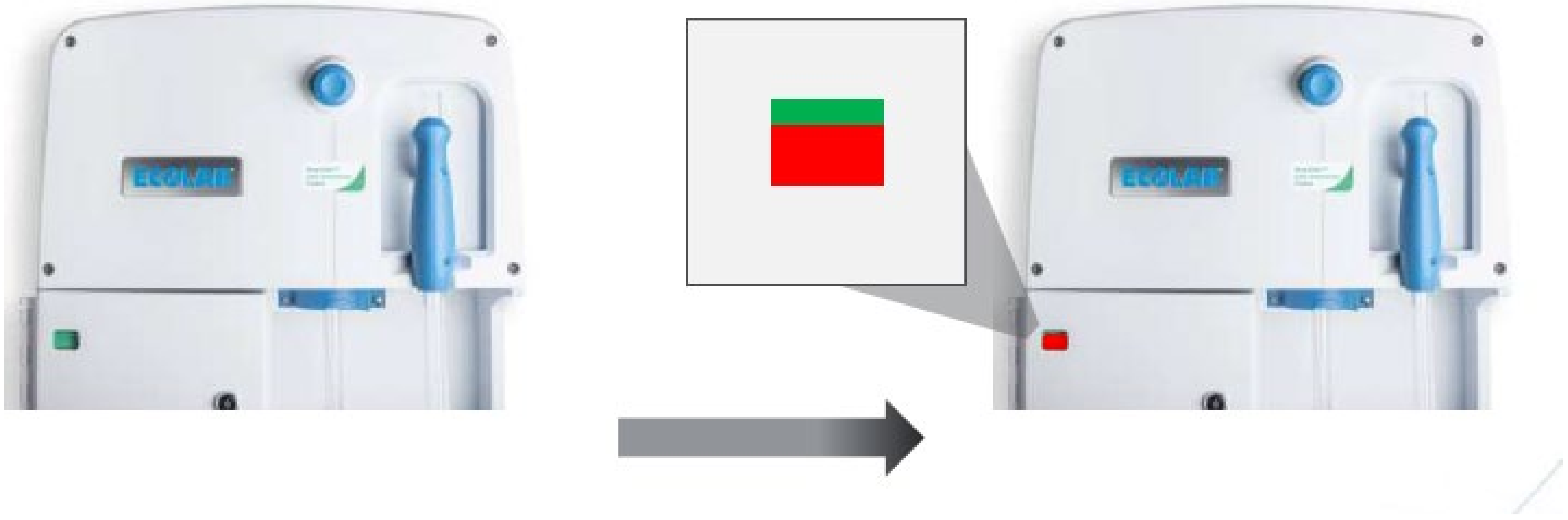

Dispenser Wall Card

- Posted near dispenser
- Reminder of **PPE** to use when dispensing
- Step-by-Step instructions for preparing work bottles and cloths
- **5 minute** contact/dwell time
- Shelf Life 1 day
  - Make fresh solution at beginning of shift
  - Discard solution in bottles/buckets at end of shift

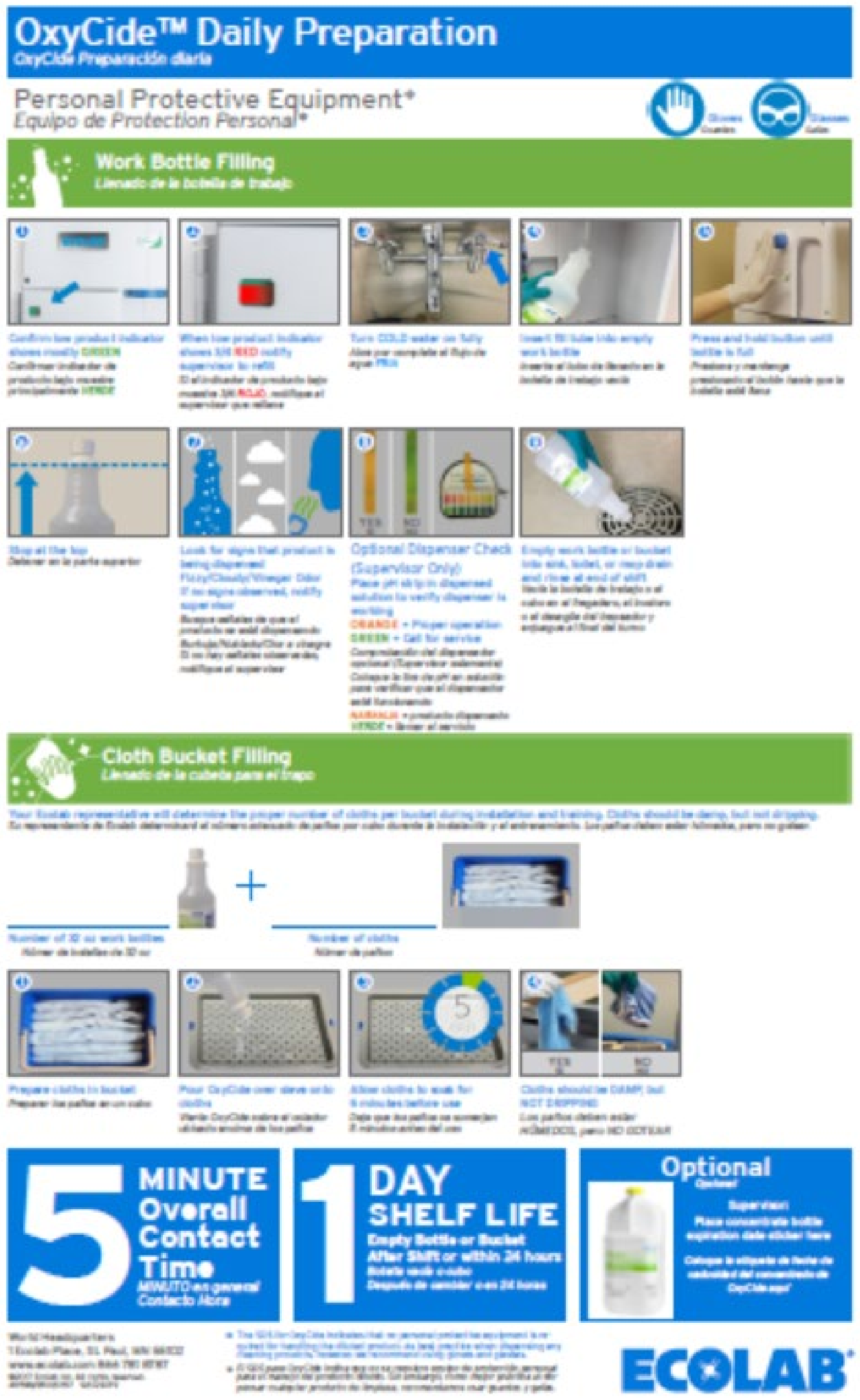

ECOLAB

How Do You Use the Oxycide Dispenser?

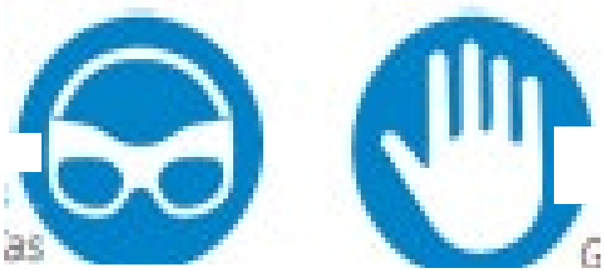

- Put on gloves and eye protection glasses as best practice for dispensing any chemistry

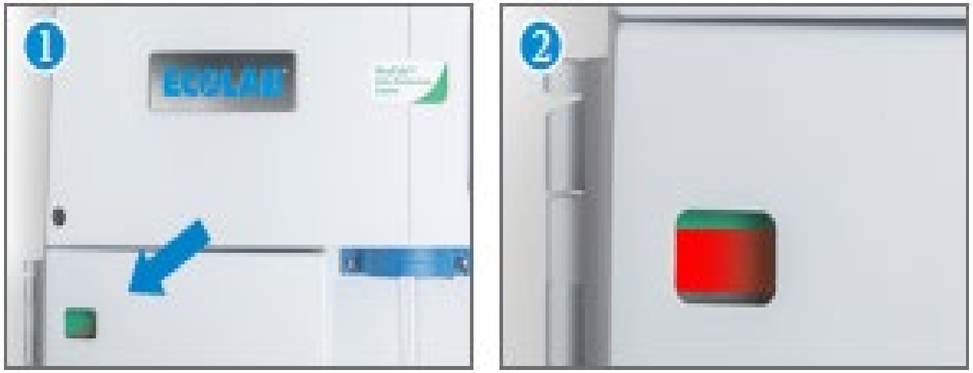

- **LOOK before you push <u>EACH AND EVERY TIME</u>**
  - Low Product Indicator is mostly **GREEN.** If **¾ RED**, tell you supervisor

How Do You Use the Oxycide Dispenser?

- Turn **COLD** water fully on (if needed)

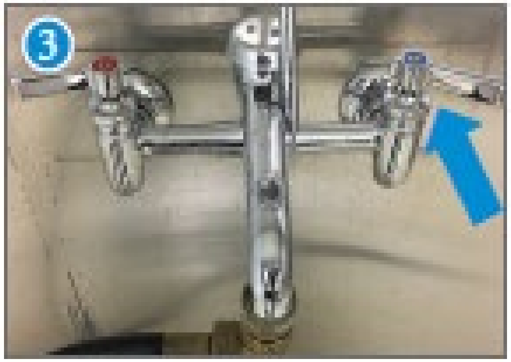

- Insert short tube into an **EMPTY** work bottle

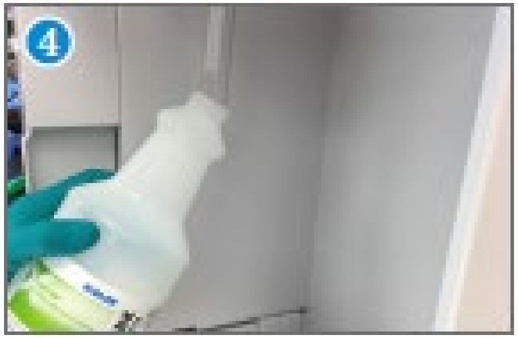

ECOLAB®

How Do You Use the OxyCide™ Dispenser?

- Chemical only in concentrate…the chemical line/tube drains back into the concentrate bottle after each dispense, therefore the chemical line is not primed with chemical
- So, when the **BLUE** dispensing button is pushed, only water comes out as it take 3-5 seconds until the chemical to be pulled from the concentrate bottle
  - Chemical lags behind
- THEREFORE…

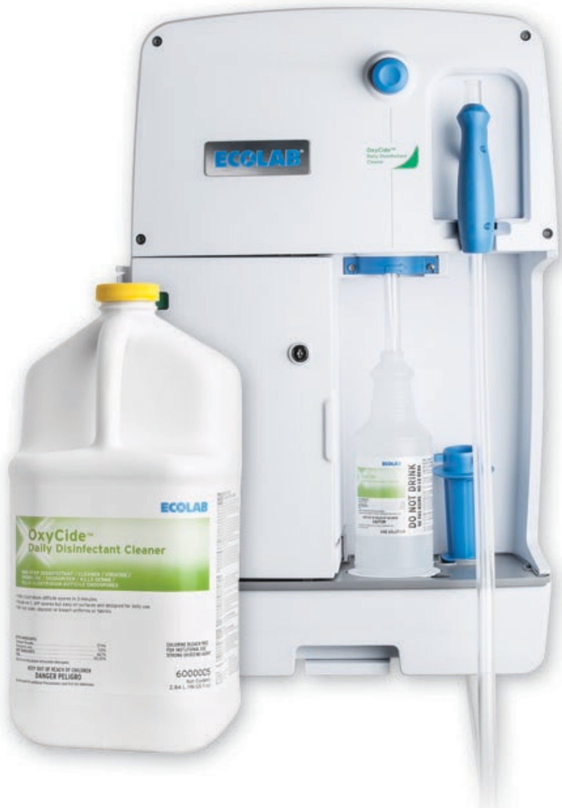

ECOLAB

How Do You Use the OxyCide Dispenser?

To get the right mixture of water and chemical (dilution rate) to kill germs, you must **ALWAYS…**

- Start with an **EMPTY 32 oz. bottle**
- **PUSH AND HOLD down the BLUE operating button…**
  - One non-stop push
- **Until the bottle is filled to the top; STOP AT THE TOP**

STOP AT THE TOP

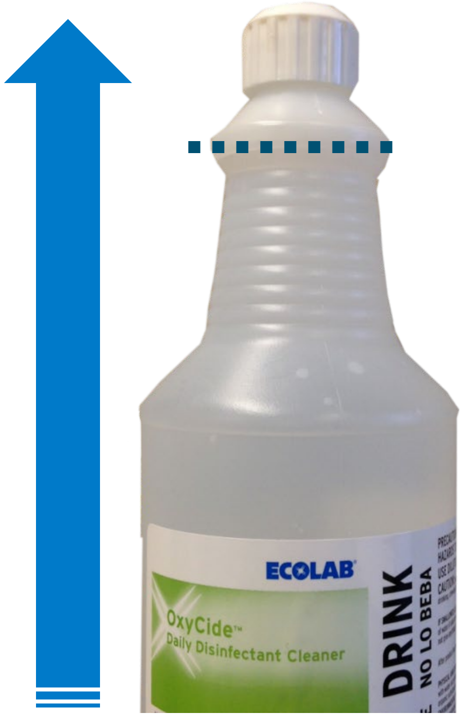

ECOLAB

How Do You Use the OxyCide™ Dispenser?

When filling a Work Bottle you CANNOT…

- Start and stop, start and stop
- Partially fill a bottle
- "Top Off" (add to solution already in a bottle)
    - Wrong dilution rate won't kill germs
    - Cross-contamination of solution
    - Mixing different shelf lives

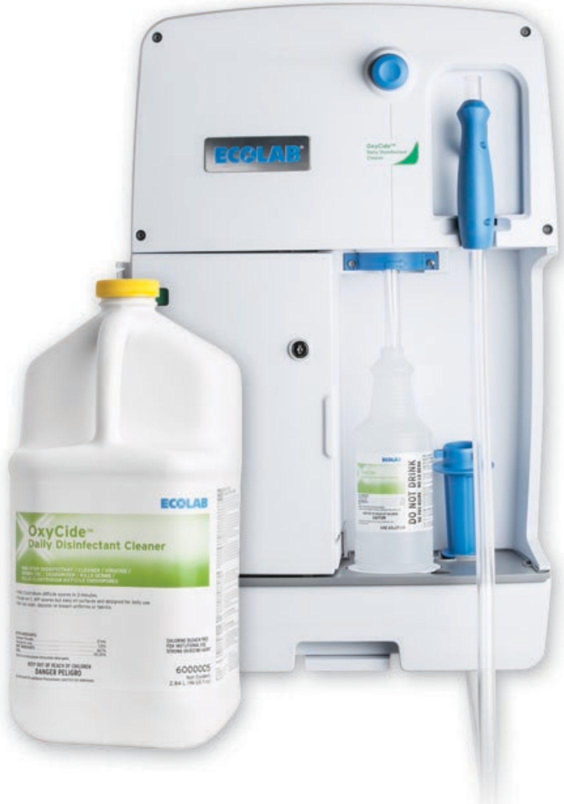

ECOLAB

How Do You Use the Oxycide Dispenser?

- **PRESS <u>AND</u> HOLD BLUE** dispensing until the bottle is full
  - One non-stop push
  - No starting and stopping

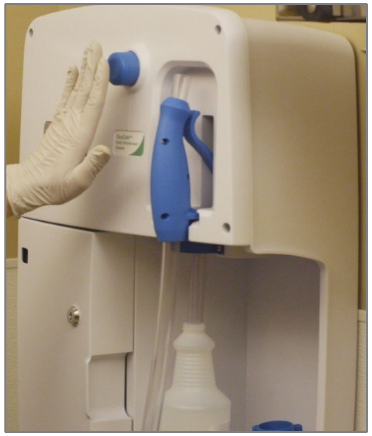

- **STOP AT THE TOP**
  - Flow stops as soon as button is released

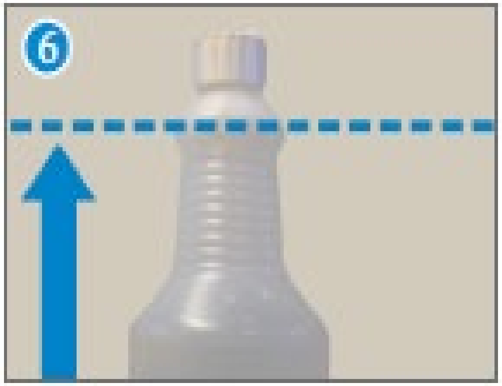

ECOLAB

How Do You Use the Dispenser?

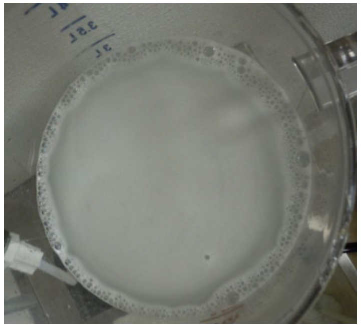

- **AFTER** filling a work bottle **AND BEFORE** pouring OxyCide over the cloths or putting on the flip top, look into the bottle **to make sure chemical and not just water is being dispensed** (dispenser working)
- Look for **3 signs** that product is being dispensed (Quality Check)
  1. Fizzy/tiny bubbles
  2. Cloudy (goes from cloudy to clear)
  3. Vinegar smell

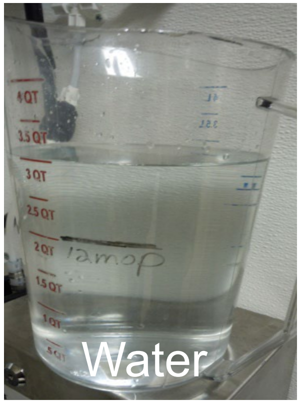

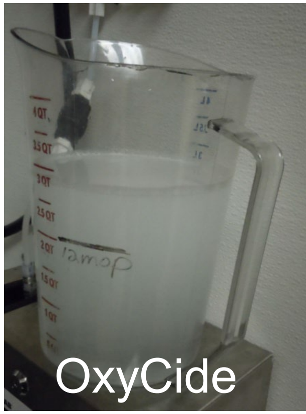

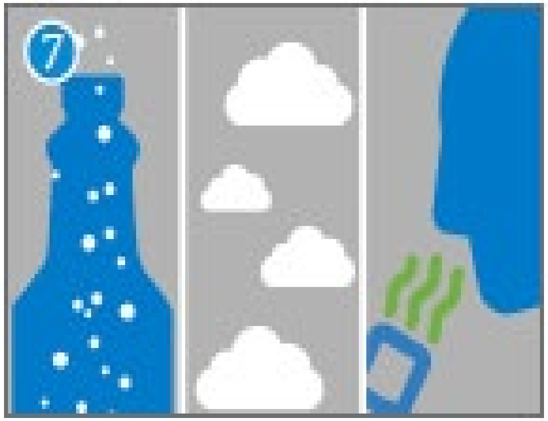

How Do you Use the Dispenser?

- Supervisor may also perform additional quality check using pH strips
  - After changing out the concentrate bottle
  - When question if a dispenser is not working properly
  - When question if use solution is over cleaning cloths

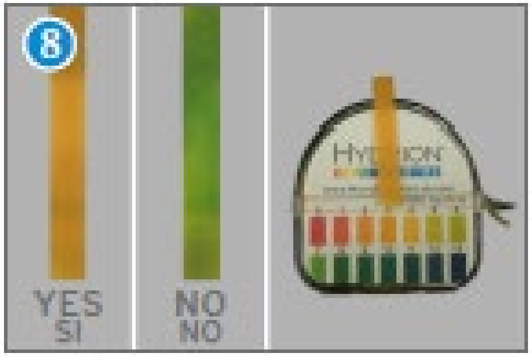

How Do You Use the OxyCide Dispenser?

- After done dispensing use solution:
  - Turn **COLD** water fully off if needed
  - Remove gloves then chemical goggles
  - Wash hands with soap and water before eating, drinking, chewing gum, using tobacco, using restroom

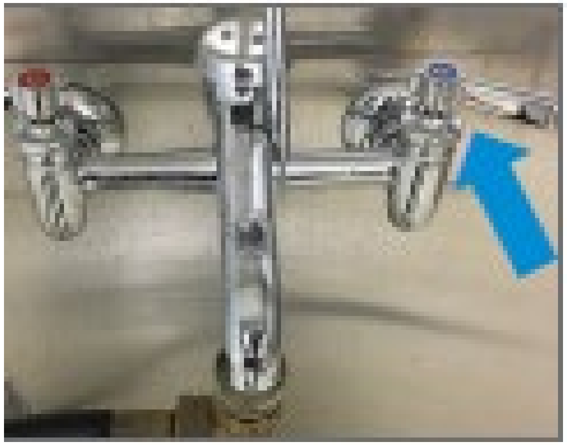

ECOLAB

Using OxyCide Safely

- Do NOT “top off” work bottles
- Do NOT mix with BLEACH or other chemicals (no “wet-on-wet”)
- Do NOT mix toilet bowl acid with OxyCide on Johnny Mop
- Do NOT mix Cream cleanser with cloth pre-soaked in OxyCide
- Do NOT use on soft metals: Copper, Aluminum, Brass

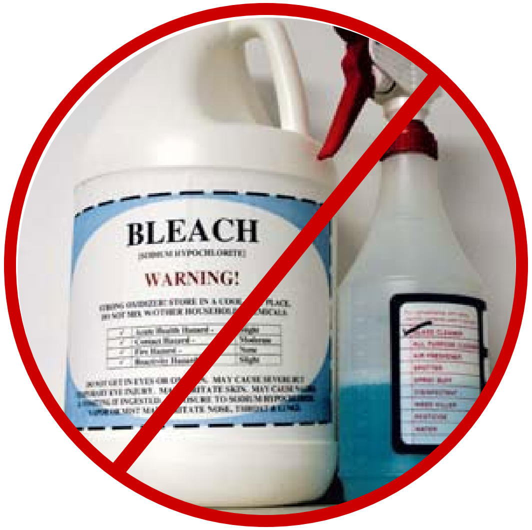

No “wet-on-wet” contact between chemistries

Right-to-Understand Information

- Safety Data Sheet (SDS) provides information on chemicals
- OSHA "Right to Understand"
- Provides information for product:
  - As Sold = Concentrated product
  - Use Solution = Diluted product

ECOLAB

ECOLAB SAFETY DATA SHEET

OXYCIDE DAILY DISINFECTANT CLEANER

SECTION 1. PRODUCT AND COMPANY IDENTIFICATION

Product name	:	OXYCIDE DAILY DISINFECTANT CLEANER
Other means of identification	:	Not applicable
Recommended use	:	Disinfectant
Restrictions on use	:	Reserved for industrial and professional use.
Product dilution information	:	0.0 % - 2.34 %
Company	:	Ecolab Inc.<br>370 N. Wabasha Street<br>St. Paul, Minnesota USA 55102<br>1-800-352-5326
Emergency health information	:	1-800-328-0026 (US/Canada), 1-651-222-5352 (outside US)
Issuing date	:	05/31/2016

SECTION 2. HAZARDS IDENTIFICATION

GHS Classification

Product AS SOLD

Oxidizing liquids	:	Category 2
Organic peroxides	:	Type F
Acute toxicity (Oral)	:	Category 4
Acute toxicity (Inhalation)	:	Category 3
Skin corrosion	:	Category 1A
Serious eye damage	:	Category 1

Product AT USE DILUTION

Acute toxicity (Oral)	:	Category 4

GHS label elements

Product AS SOLD

Hazard pictograms :

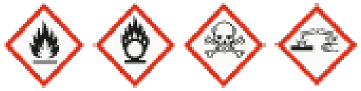

Signal Word	:	Danger
Hazard Statements	:	Heating may cause a fire.<br>May intensify fire; oxidizer.<br>Harmful if swallowed.<br>Causes severe skin burns and eye damage.<br>Toxic if inhaled.
Precautionary Statements	:	**Prevention:**<br>Keep away from heat/sparks/open flames/hot surfaces. No smoking. Keep/Store away from clothing/ combustible materials. Take any precaution to avoid mixing with combustibles. Keep only in original container. Keep cool. Avoid breathing dust/ fume/ gas/ mist/ vapors/

ECOLAB®

Using OxyCide Safely

- Make sure bottles and buckets are properly labeled

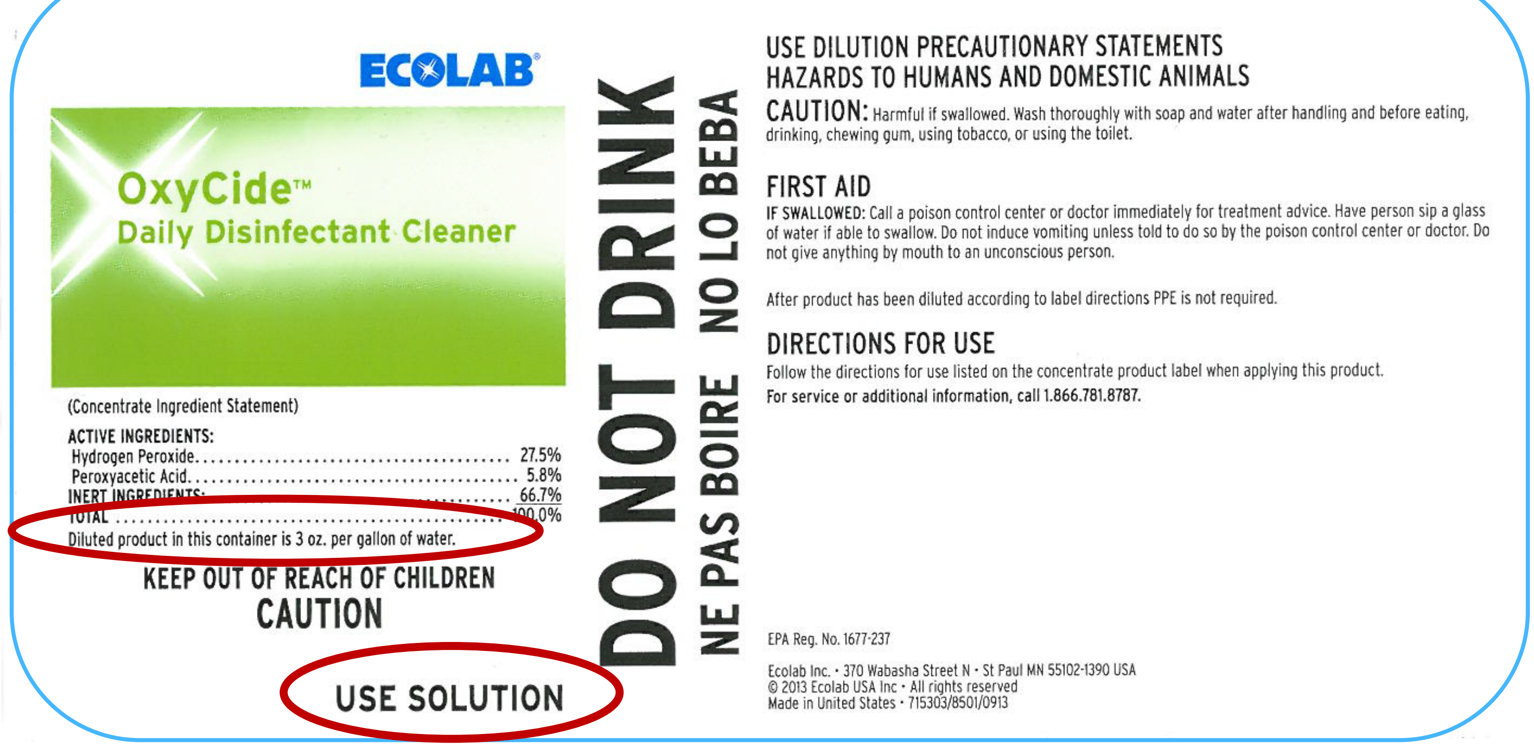

Next Steps

- Post-training quiz
- Demonstrate use of dispenser
- Begin using OxyCide
- Ecolab staff will be rounding to provide assistance and answer questions

QUESTIONS

OxyCide Training Notes

- THIS SLIDE CONTAINS KEY POINTS TO COMMUNICATE IF THE ENTIRE TRAINING PRESENTATON CANNOT BE CONDUCTED IN A CLASSROOM SETTING AND WILL BE DONE IN SMALL GROUPS IN CLOSET
- 1 in every 25 hospital patients gets an HAI[1]
  - 14,000 people die every year in the US because of C. diff
- Your Job Matters
  - EVS plays a very important role in preventing the spread of infection and increasing patient safety.
- OxyCide Overview
  - Use and show wall card as training aid
  - 5 min. contact time; PPE; cart set-up; 1-shift shelf life
  - Used on all hard surfaces except the floor in any type of room
- How to use the dispenser
  - TIP, turn cold water fully on; 3 signs: fizzy, cloudy, vinegar smell; stop at the top, continuous flow
  - Low Product Indicator: ¾ red, ¼ green = notify your manager

ECOLAB®

1 http://www.cdc.gov/HAI/surveillance/index.html

CATSWeb and SafetyCall Data for OxyCide™

As part their product stewardship program, Ecolab tracked and monitored complaints of adverse health effects regarding the OxyCide™ product via two systems: 1) an internal monitoring system called CATSWeb, which is a software system that was used for tracking complaints about the product, including those of adverse health effects, and 2) an external monitoring system called SafetyCall, which is a third party who employs medical and health professionals who review and categorize complaints and then send them to the USEPA. Data from both monitoring services demonstrates that complaints of respiratory irritation decreased from 2013 to 2020 (Figures 1a and 1b, below). Figure 1a summarizes the complaints of respiratory irritation regarding OxyCide™ recorded in the CATSWeb system from 2013 through 2020. Figure 1b summarizes the complaints of respiratory irritation regarding OxyCide™ recorded by SafetyCall from 2013 through 2021.

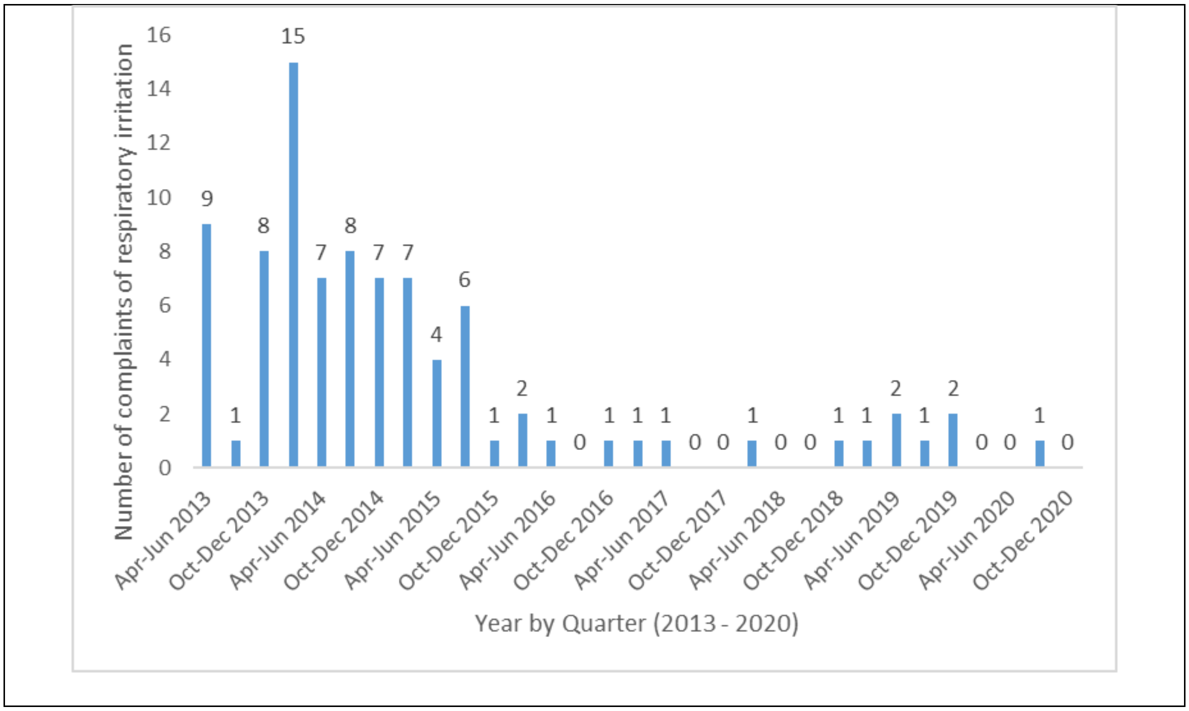

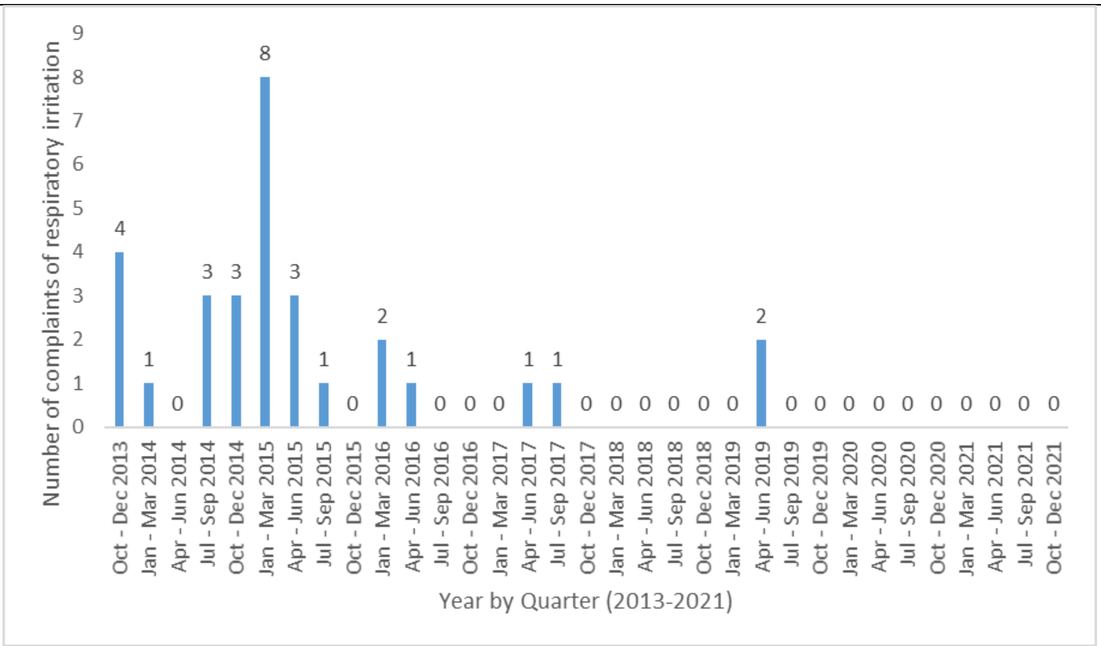

Figure 1a (top). Number of complaints of respiratory irritation regarding OxyCide™ recorded in the CATSWeb system quarterly from 2013 through 2020. Figure 1b (bottom). Number of complaints of respiratory irritation regarding OxyCide™ recorded by SafetyCall quarterly from 2013-2021.
